# Supplementary material for: Asymmetric three-component olefin dicarbofunctionalization enabled by photoredox and copper dual catalysis
Source: Nat Commun. 2021 Mar 22;12:1815. doi: 10.1038/s41467-021-22127-x (PMC7985521; doi:10.1038/s41467-021-22127-x)
Supplement: Supplementary file 1 — Supplementary Information [file 41467_2021_22127_MOESM1_ESM.pdf]

Asymmetric Three-Component Olefin Dicarbofunctionalization Enabled by Photoredox and Copper Dual Catalysis

Peng-Zi Wang[+], Yuan Gao[+], Jun Chen, Xiao-Die Huan, Wen-Jing Xiao & Jia-Rong Chen**

Supporting Information

1. General Information 1

2. Preparation of the Radical Precursors 2

2.1 Preparation of the Acyl Radical Precursors **3a-3p** 2

2.2 Preparation of the Cyclobutanone Oxime Esters **4a-4g** 3

3. Detailed Optimization of Reaction Conditions 4

3.1 Optimization of Reaction Conditions And Control Experiments for Synthesis of **6aa** 4

3.2 Optimization of Reaction Conditions And Control Experiments for Synthesis of **7ia** 11

4. General Procedure and Spectral Data of Products 15

4.1 General Procedure for Synthesis of **6aa** 15

4.2 General Procedure for Synthesis of **7ia** 16

4.3 Spectral Data of Products **6** and **7** 16

4.4 Investigation of reaction system of cyclic acyl precusor **3q** 35

5. Synthetic Applications of the Reaction 38

5.1 1.0 mmol Reaction 38

5.2 Gram-Scale Reaction 38

5.3 Transfomations of Products **6ba** and **7ia** 39

6. The Mechanism Studies 41

6.1 Luminescence Quenching Experiments 41

6.2 UV-Vis Absorption Spectra 41

6.3 Light On-Off Experiments 42

6.4 Determination of Quantum Yields 43

6.5 Non-Linear Effect Experiments 43

6.6 Radical Trapping Experiments 45

6.7 Radical Clock Experiments 46

7. Determination of the Absolute Configuration of Products 6bj and 7ja 47

8. The Spectra of Substrates and Products 62

9. Copies of HPLC Spectra 140

1. General Information

NMR spectra: ^{1}H NMR spectra were recorded on a 400 MHz spectrometer. Chemical shifts are reported in parts per million (ppm) and the spectra are calibrated to the resonance resulting from incomplete deuteration of the solvent ($CDCl_3$: 7.26 ppm). ^{13}C NMR spectra were recorded on the same spectrometer with complete proton decoupling. Chemical shifts are reported in ppm with the solvent resonance as the internal standard ($^{13}CDCl_3$: 77.0 ppm, t). Data are reported as follows: chemical shift δ/ppm, integration (^{1}H only), multiplicity (s = singlet, d = doublet, t = triplet, q = quartet, m = multiplet or combinations thereof; ^{13}C signals are singlets unless otherwise stated), coupling constants *J* in Hz, assignment. ^{19}F NMR spectra were recorded on the same Spectrometer. All air- and moisture-sensitive reactions were performed under an atmosphere of Ar in fire dried glassware.

High Resolution Mass Spectrometry (HRMS): All were recorded on Bruker micrOTOF II ESI-TOF using a positive electrospray ionization (ESI^+). Measured values are reported to 4 decimal places of the calculated value. The calculated values are based on the most abundant isotope.

Chromatography: Analytical thin layer chromatography was performed using Qingdao Puke Parting Materials Co. silica gel plates (Silicagel 60 F254). Visualisation was by ultraviolet fluorescence (λ = 254 nm) and/or staining with Phosphomolybdic acid or potassium permanganate ($KMnO_4$). Flash column chromatography was performed using 200-300 mesh silica gel. Optical rotations were measured with a polarimeter. $[\alpha]_D$ values are reported at a given temperature (° C) in degrees $cm^2 \cdot g^{-1}$ with concentration in g/100 mL.

Chiral HPLC or GC analysis: Enantiomeric ratio (ee) values were determined by chiral HPLC with chiral AS, AD, AZ and OD columns with hexane and *i*-PrOH as solvents. GC yields were determined by Agilent 7890B/5977B.

UV/Vis: Measurements were made with Agilent G9800A Spectro Fluorophotometer and Agilent Technologies Cary 60 UV-Vis.

2. Preparation of the Radical Precursors

2.1 Preparation of the Acyl Radical Precursors 3a-3p

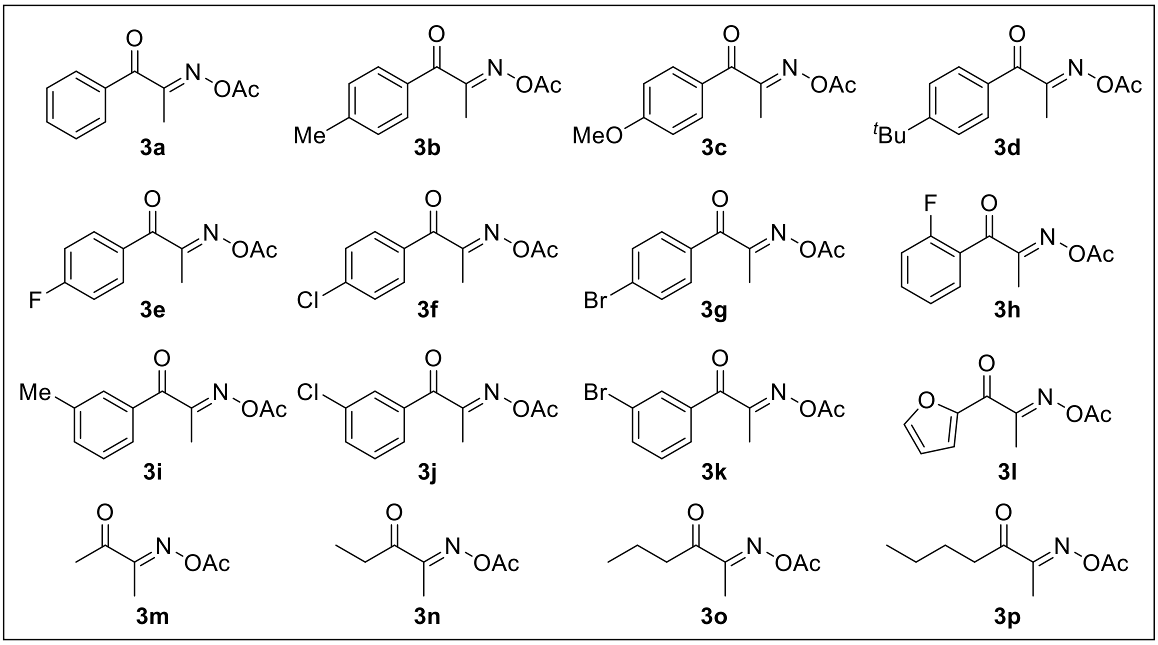

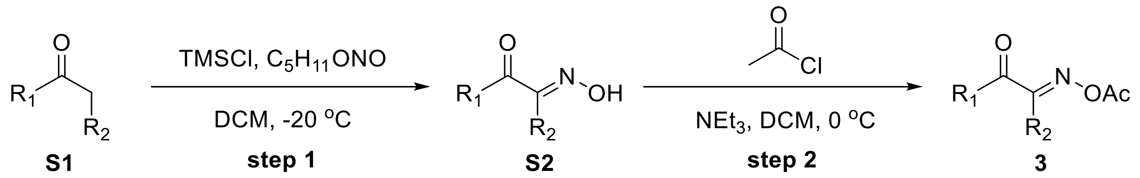

The above oxime esters **3a-3p** were synthesized according to the method below.

Step 1: To a solution of 10 mmol ketone in 20 mL DCM was added 1 equiv TMSCl (trimethylchlorosilane, 1.24 mL) at -20 ℃. To this cooled solution was dropwise added 1 equiv isoamyl nitrite (1.34 mL). The reaction was found to be instantaneous, but the mixture was stirred at r.t. for an additional period of 1 h before working up. The solution was directly concentrated in vacuo. The crude product was purified by flash column chromatography on silica gel (eluting with PE/EA = 20:1) and the corresponding oximes **S2** was obtained.

Step 2: To a solution of oxime **S2** and 1.5 equiv triethylamine (2.08 mL) in 20 mL DCM was slowly added a solution of 1.2 equiv acyl chloride in DCM (15 mL) at 0 ℃. The mixture was stirred at r.t. for 2 h. After completion, the reaction was quenched with 50 mL $NaHCO_3$ saturated solution and extracted with 50 mL DCE for three times. The extract was washed with brine and dried over Na_2SO_4 and concentrated in vacuo. The crude product was purified by flash column chromatography on silica gel (eluting with PE/EA = 20:1).

2.2 Preparation of the Cyclobutanone Oxime Esters 4a-4g

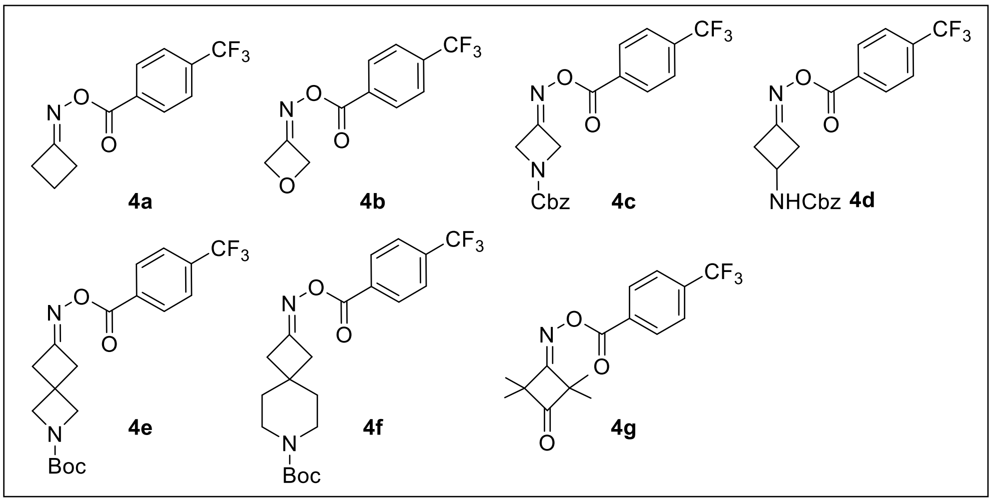

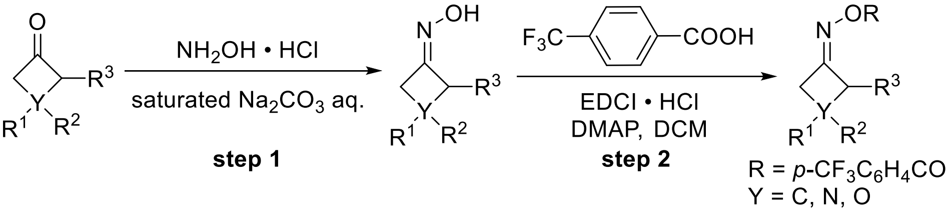

The above oxime esters **4a-4g** were synthesized according to the method below.

Step 1: The ketone (10.0 mmol, 1.0 equiv) and hydroxylamine hydrochloride (15 mmol, 1.5 equiv) were placed in a flask equipped with stirrer. The pH of the solution was held at 7-8 by adding saturated aq. sodium carbonate. Then stirring solution at 40 °C. After completion, the mixture was extracted with DCM, the solution was dried over Na_2SO_4 and concentrated in vacuo, provide crude products which were used in next step without further purification.

Step 2: To a solution of oxime (1.0 equiv) and 4-(trifluoromethyl)benzoic acid (1.5 equiv) in DCM (0.2 M) was added EDCI·HCl (2.0 equiv) and DMAP (0.2 equiv). The mixture was stirred at r.t. overnight. After completion, the reaction was diluted with water and extracted with DCM. The extract was washed with brine and dried over Na_2SO_4 and concentrated in vacuo. The residue was purified by flash column chromatography on silica gel with PE-EtOAc as an eluent to give oxime esters.

3. Detailed Optimization of Reaction Conditions

3.1 Optimization of Reaction Conditions And Control Experiments for Synthesis of 6aa

***Table S1*. The Effect of Solvents[a]**

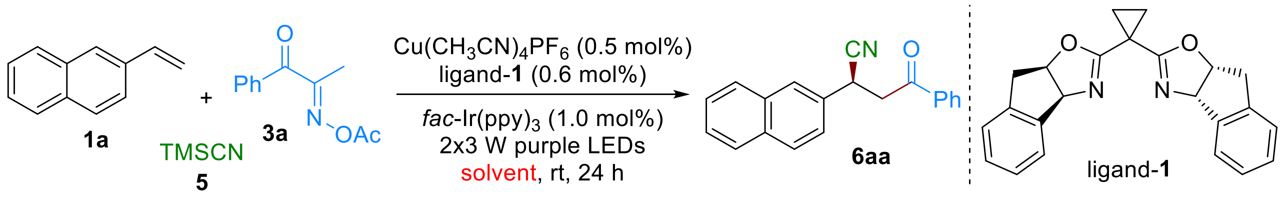

Entry	Solvents	Yield [%][b]	ee [%][c]
1	CH_3CN	6	90
2	DCM	9	90
3	THF	15	90
4	DCE	14	85
5	$CHCl_3$	31	84
6	DMA	41	88
7	DMF	39	89

[a] **3a** (0.3 mmol), **1a** (0.1 mmol, 1.0 equiv), **5** (0.3 mmol), $Cu(CH_3CN)_4PF_6$ (0.5 mol%), chiral ligand-**1** (0.6 mol%) and photocatalyst *fac*-Ir(ppy)$_3$ (1.0 mol%) in 2.0 mL solvent for 24 h under the irradiation of 2 x 3 W purple LEDs. [b] Determined by GC analysis using 1,3,5-trimethoxybenzene as an internal standard. [c] Determined by chiral HPLC.

As shown in **Table *S1***, among all the tested, DMA (2.0 mL) gave the best results (41% yield, 88% ee), and was thus selected for further optimization studies.

***Table S2*. The Ratio of Substrates[a]**

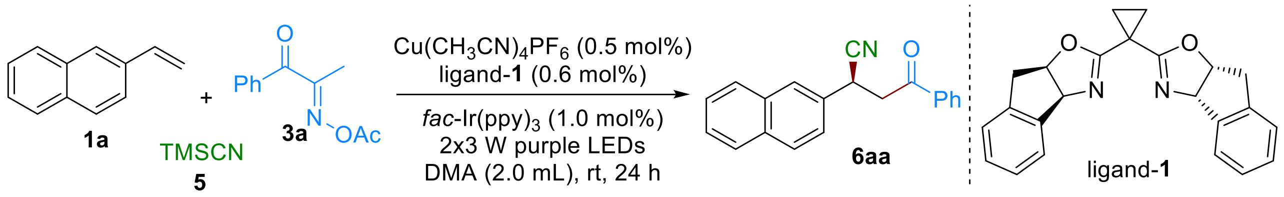

Entry	Ratio of X:Y:Z	Yield [%][b]	ee [%][c]
1	2:1:2	37	64
2	4:1:4	28	68
3	5:1:5	25	62
4	3:1:5	27	75
5	5:1:3	39	60
6	3:1:3	41	88

[a] **3a** (X equiv), **1a** (Y equiv, 0.1 mmol), **5** (Z equiv), $Cu(CH_3CN)_4PF_6$ (0.5 mol%), chiral ligand-**1** (0.6 mol%) and photocatalyst *fac*-Ir(ppy)$_3$ (1.0 mol%) in 2.0 mL solvents for 24 h under the irradiation of 2 x 3 W purple LEDs. [b] Determined by GC analysis using 1,3,5-trimethoxybenzene as an internal standard. [c] Determined by chiral HPLC.

As shown in **Table *S2***, among all the tested, the substrates ratio of X:Y:Z = 3:1:3 gave the best results (41% yield, 88% ee), and was thus selected for further optimization studies.

***Table S3.* The Effect of Loading of Photocatalyst** [a]

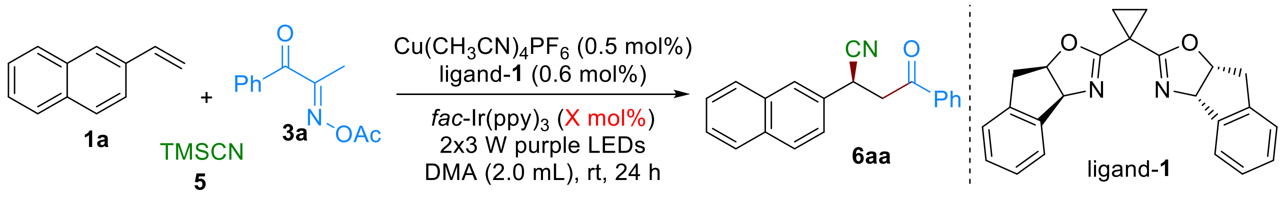

Entry	Loading of photocatalyst	Yield [%][b]	ee [%][c]
1	0.2 mol%	19	79
2	0.4 mol%	28	85
3	0.6 mol%	43	86
4	0.8 mol%	64	88
5	1.0 mol%	41	88

[a] **3a** (0.3 mmol), **1a** (0.1 mmol, 1.0 equiv), **5** (0.3 mmol), $Cu(CH_3CN)_4PF_6$ (0.5 mol%), chiral ligand-**1** (0.6 mol%) and photocatalyst *fac*-$Ir(ppy)_3$ (X mol%) in 2.0 mL DMA for 24 h under the irradiation of 2 x 3 W purple LEDs. [b] Determined by GC analysis using 1,3,5-trimethoxybenzene as an internal standard. [c] Determined by chiral HPLC.

As shown in **Table *S3***, among all the tested, 0.8 mol% of *fac*-$Ir(ppy)_3$ gave the best results (64% yield, 88% ee), and was thus selected for further optimization studies.

***Table S4*. The Effect of Copper Salts**[a]

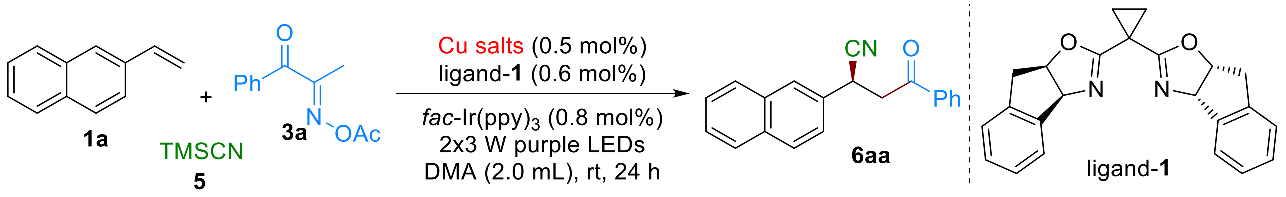

Entry	Copper salts	Yield [%][b]	ee [%][c]
1	CuTC	38	85
2	CuCN	62	86
3	CuCl	51	85
4	CuI	69	81
5	CuBr	57	82
6	$Cu(OTf)_2$	16	68
7	$Cu(OAc)_2$	66	82
8	$Cu(CH_3CN)_4PF_6$	64	88

[a] **3a** (0.3 mmol), **1a** (0.1 mmol, 1.0 equiv), **5** (0.3 mmol), copper salts (0.5 mol%), chiral ligand-**1** (0.6 mol%) and photocatalyst *fac*-$Ir(ppy)_3$ (0.8 mol%) in 2.0 mL DMA for 24 h under the irradiation of 2 x 3 W purple LEDs. [b] Determined by GC analysis using 1,3,5-trimethoxybenzene as an internal standard. [c] Determined by chiral HPLC.

As shown in **Table *S4***, among all the tested, $Cu(CH_3CN)_4PF_6$ gave the best results (64% yield, 88% ee), and was thus selected for further optimization studies.

***Table S5*. The Effect of Loading of Cu Salt and Chiral Ligand**[a]

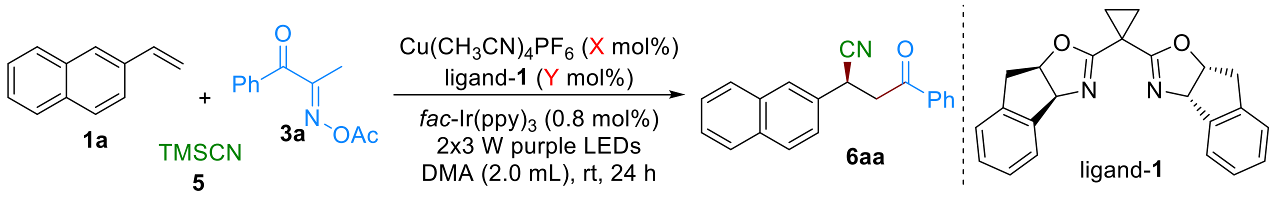

Entry	Loading of Cu /Ligand	Yield [%][b]	ee [%][c]
1	X = 1.2; Y = 1.44	73	88
2	X = 1.2; Y = 1.8	79	88
3	X = 1.5; Y = 2.25	78	90

[a] **3a** (0.3 mmol), **1a** (0.1 mmol, 1.0 equiv), **TMSCN** (0.3 mmol), $Cu(CH_3CN)_4PF_6$ (X mol%), chiral ligand-**1** (Y mol%) and photocatalyst *fac*-Ir(ppy)$_3$ (0.8 mol%) in 2.0 mL DMA for 24 h under the irradiation of 2 x 3 W purple LEDs. [b] Determined by GC analysis using 1,3,5-trimethoxybenzene as an internal standard. [c] Determined by chiral HPLC.

As shown in **Table *S5***, among all the tested, $Cu(CH_3CN)_4PF_6$ (1.5 mol%) and chiral ligand-**1** (2.25 mol%) gave the best results (78% yield, 90% ee), and was thus selected for further optimization studies.

***Table S6*. The Effect of Concentration** [a]

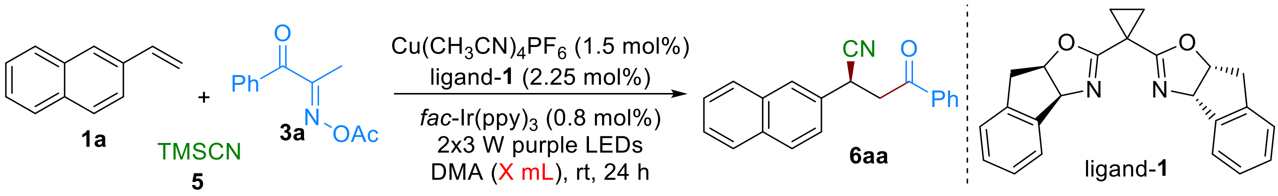

Entry	Concentration	Yield [%][b]	ee [%][c]
1	1.0 mL (0.1 M)	58	89
2	2.0 mL (0.05 M)	78	90
3	2.5 mL (0.04 M)	88	90
4	3.0 mL (0.03 M)	84	81

[a] **3a** (0.3 mmol), **1a** (0.1 mmol, 1.0 equiv), **5** (0.3 mmol), $Cu(CH_3CN)_4PF_6$ (1.5 mol%), chiral ligand-**1** (2.25 mol%) and photocatalyst *fac*-Ir(ppy)$_3$ (0.8 mol%) in DMA (X mL) for 24 h under the irradiation of 2 x 3 W purple LEDs. [b] Determined by GC analysis using 1,3,5-trimethoxybenzene as an internal standard. [c] Determined by chiral HPLC.

As shown in **Table *S6***, among all the tested, DMA (2.5 mL) gave the best results (88% yield, 90% ee), and was thus selected for further optimization studies.

***Table S7*. The Effect of Chiral Ligands[a]**

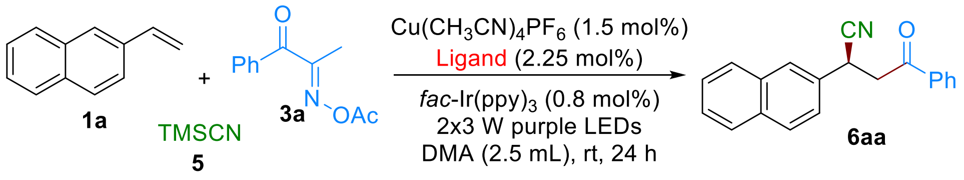

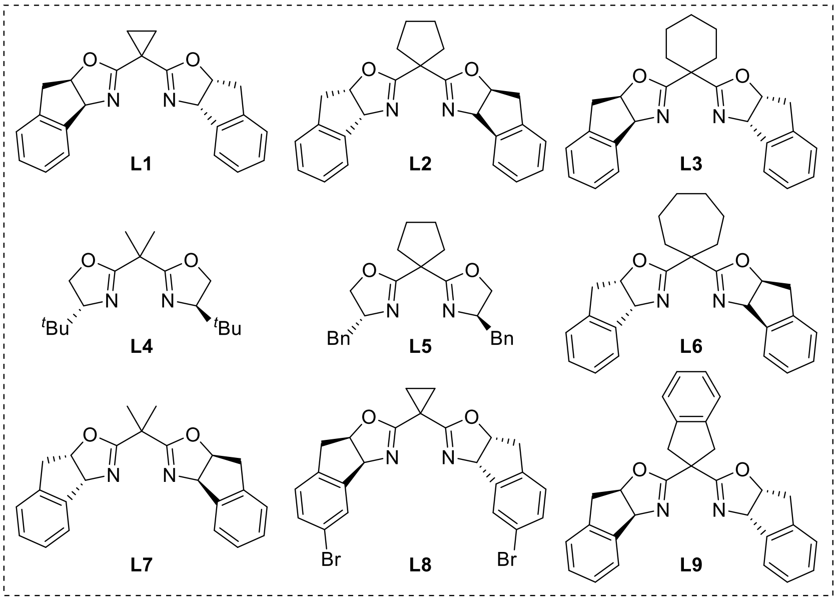

Entry	Concentration	Yield [%][b]	ee [%][c]
1	**L1**	88	90
2	**L2**	16	-13
3	**L3**	35	3
4	**L4**	36	-1
5	**L5**	33	-10
6	**L6**	34	-1
7	**L7**	32	-7
8	**L8**	45	77
9	**L9**	34	8

[a] **3a** (0.3 mmol), **1a** (0.1 mmol, 1.0 equiv), **5** (0.3 mmol), $Cu(CH_3CN)_4PF_6$ (1.5 mol%), chiral ligand (2.25 mol%) and photocatalyst *fac*-$Ir(ppy)_3$ (0.8 mol%) in DMA (2.5 mL) for 24 h under the irradiation of 2 x 3 W purple LEDs. [b] Determined by GC analysis using 1,3,5-trimethoxybenzene as an internal standard. [c] Determined by chiral HPLC.

As shown in **Table *S7***, among all the tested, **L1** gave the best results (88% yield, 90% ee), and was thus selected for further optimization studies.

Table S8. **The Effect of Protecting Groups[a]**

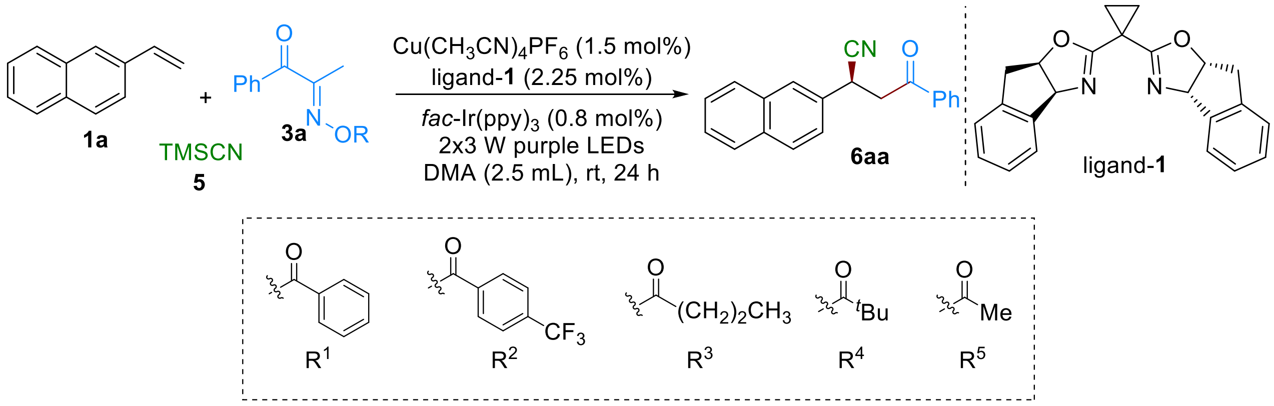

Entry	Protecting groups	Yield [%][b]	ee [%][c]
1	R^1	35	86
2	R^2	39	80
3	R^3	45	87
4	R^4	88	88
5	R^5	88	90

[a] **3a** (0.3 mmol), **1a** (0.1 mmol, 1.0 equiv), **5** (0.3 mmol), $Cu(CH_3CN)_4PF_6$ (1.5 mol%), chiral ligand-**1** (2.25 mol%) and photocatalyst *fac*-$Ir(ppy)_3$ (0.8 mol%) in DMA (2.5 mL) for 24 h under the irradiation of 2 x 3 W purple LEDs. [b] Determined by GC analysis using 1,3,5-trimethoxybenzene as an internal standard. [c] Determined by chiral HPLC.

As shown in **Table *S8***, among all the tested, 2-(acetoxyimino)-1-phenylpropan-1-one (R_1) gave the best results (88% yield, 90% ee), and was thus selected for further optimization studies.

Table S9. **Control Experimrnts[a]**

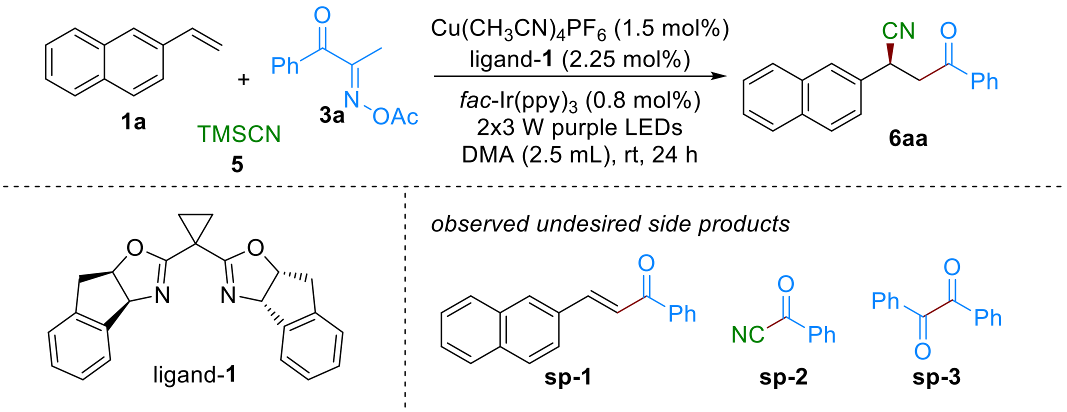

Entry[a]	*hv*	*fac*-$Ir(ppy)_3$	$Cu(CH_3CN)_4PF_6$	**L**	**sp-1/sp-2/sp-3** [%]b	Yield [%][b]	ee [%][c]
1[d]	×	√	√	√	-	N.D.	-
2[e]	√	×	√	√	2/3/2	trace.	-
3[f]	√	√	×	√	17/9/2	N.D.	-
4[g]	√	√	√	×	10/2/9	15	0
5	√	√	√	√	3/3/2	74[h]	90

[a] **3a** (0.3 mmol), **1a** (0.1 mmol, 1.0 equiv), **5** (0.3 mmol), $Cu(CH_3CN)_4PF_6$ (1.5 mol%), chiral ligand-**1** (2.25 mol%) and photocatalyst *fac*-$Ir(ppy)_3$ (0.8 mol%) in DMA (2.5 mL) for 24 h under the irradiation of 2 x 3 W purple LEDs. [b]

Determined by GC analysis using 1,3,5-trimethoxybenzene as an internal standard. [c] Determined by chiral HPLC. [d] Without *hv*. [e] Without photocatalyst *fac*-Ir(ppy)$_3$. [f] Without Cu(CH$_3$CN)$_4$PF$_6$. [g] chiral ligand-**1**. [h] Isolated yield.

The results of *Table **S9*** reveal that each component is essential for the reaction.

^{1}H-NMR and ^{13}C-NMR spectra of side products

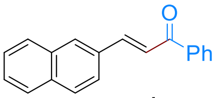

sp-1

^{1}H NMR (400 MHz, CDCl$_3$) δ (ppm) 8.11 – 8.03 (m, 3H), 7.98 (d, *J* = 15.7 Hz, 1H), 7.93 – 7.77 (m, 4H), 7.67 (s, 1H), 7.65 – 7.57 (m, 2H), 7.57 – 7.47 (m, 4H). ^{13}C NMR (100 MHz, CDCl$_3$) δ (ppm) 190.5, 144.9, 138.3, 134.4, 133.4, 132.8, 132.4, 130.7, 128.7, 128.6, 128.5, 127.8, 127.4, 126.8, 123.7, 122.2.

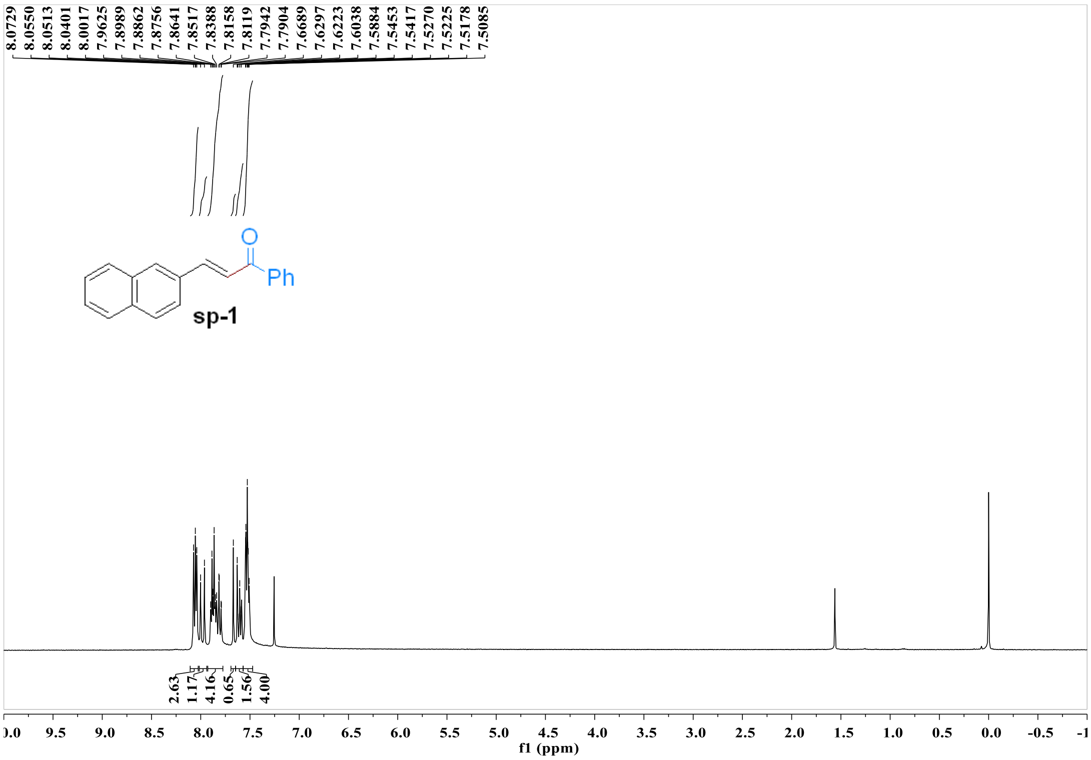

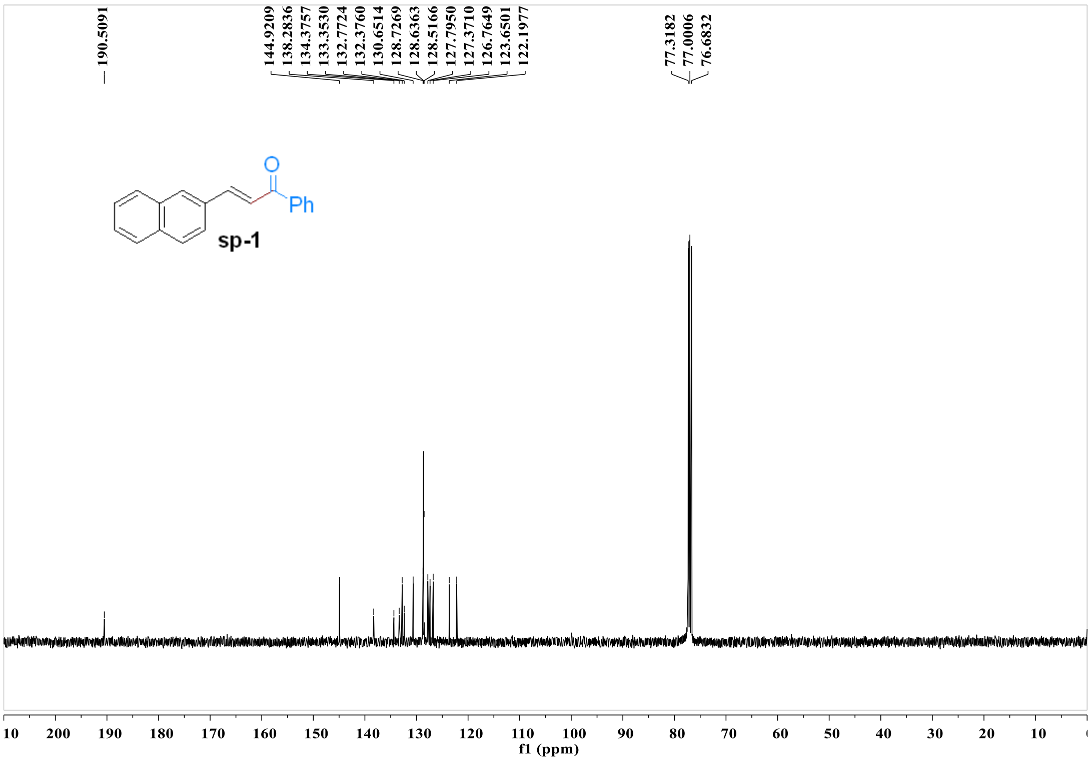
190.5091
144.9209
138.2836
134.3757
133.3530
132.7724
132.3760
130.6514
128.7269
128.6363
128.5166
127.7950
127.3710
126.7649
123.6501
122.1977
77.3182
77.0006
76.6832
O
Ph
sp-1
f1 (ppm)

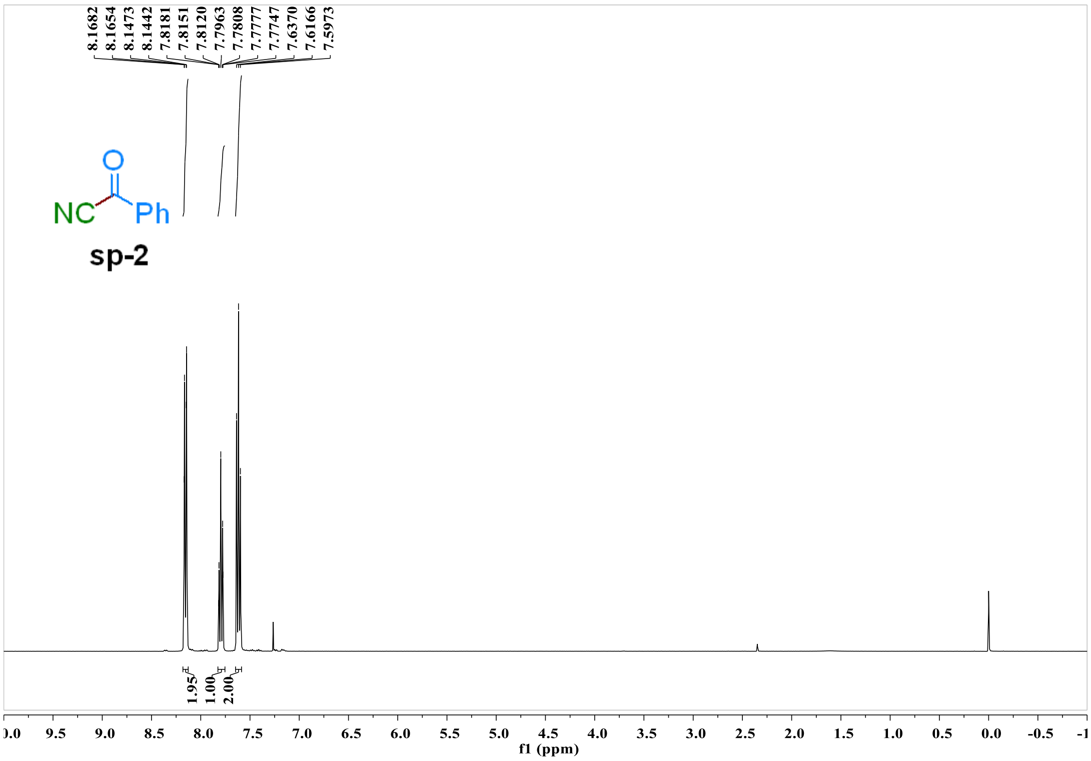
8.1682
8.1654
8.1473
8.1442
7.8181
7.8151
7.8120
7.7963
7.7808
7.7777
7.7747
7.6370
7.6166
7.5973
O
NC
Ph
sp-2
1.95
1.00
2.00
f1 (ppm)

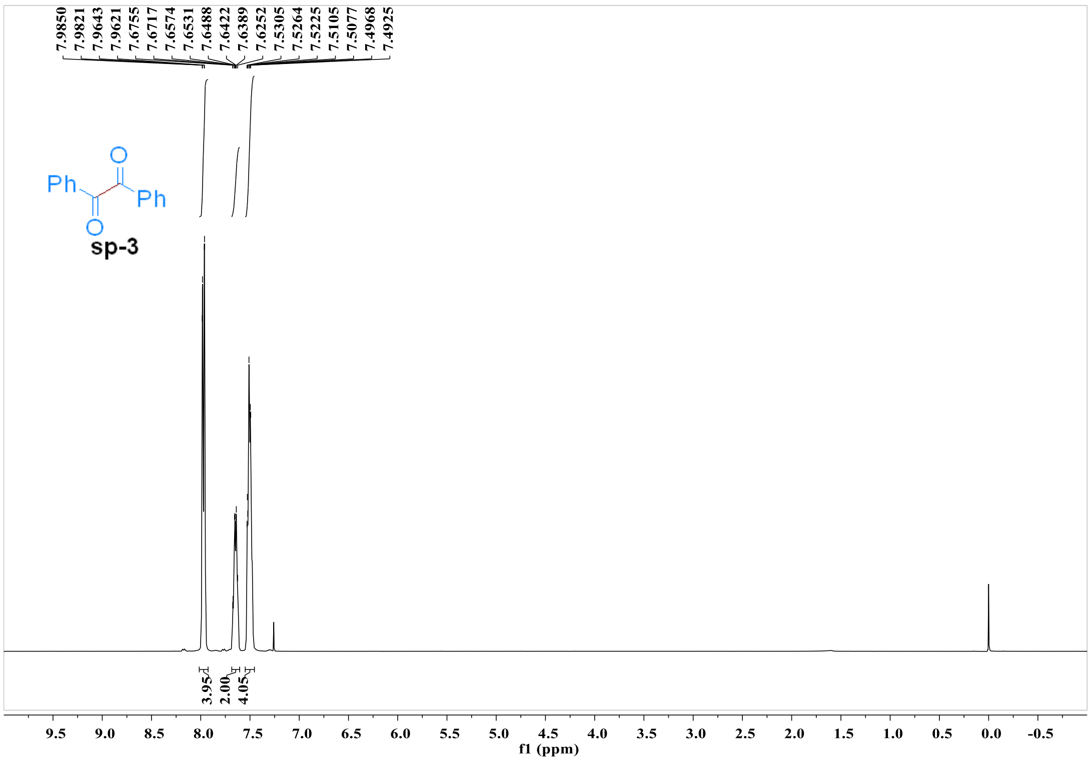

3.2 Optimization of Reaction Conditions And Control Experiments for Synthesis of 7ia

Table S10. **The Effect of Loading of Photocatalyst, Cu Salt and Chiral Ligand[a]**

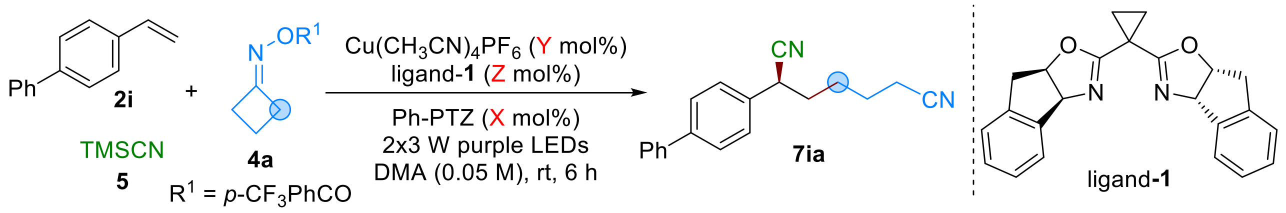

Entry	X	Y	Z	Yield [%][b]	ee [%][c]
1	5.0	10.0	12.0	5	83
2	2.5	5.0	6.0	15	84
3	2.5	2.5	3.0	16	87
4	2.5	1.0	1.2	39	90
5	1.25	0.5	0.6	65	90
6[d]	0.62	0.25	0.3	61	90
7[d]	0.31	0.12	0.15	30	89
8	1.0	0.5	0.6	55	90
9	1.5	0.5	0.6	56	90

[a] **2i** (0.1 mmol), **5** (0.3 mmol, 3 equiv), **4a** (0.3 mmol, 3 equiv), $Cu(CH_3CN)_4PF_6$ (Y mol%), chiral ligand-**1** (Z mol%) and photocatalyst Ph-PTZ (X mol%) in 2.0 mL of DMA for 6 h under the irradiation of 2 x 3 W purple LEDs. [b] Determined by GC analysis using 1,3,5-trimethoxybenzene as an internal standard. [c] Determined by chiral HPLC. [d] Reaction for 24 h.

As shown in **Table *S10***, among all the tested, $Cu(CH_3CN)_4PF_6$ (0.5 mol%), chiral ligand-**1** (0.6 mol%) and photocatalyst Ph-PTZ (1.25 mol%) gave the best results (65% yield, 90% ee), and was thus selected for further optimization studies.

***Table S11*. The Effect of concentration[a]**

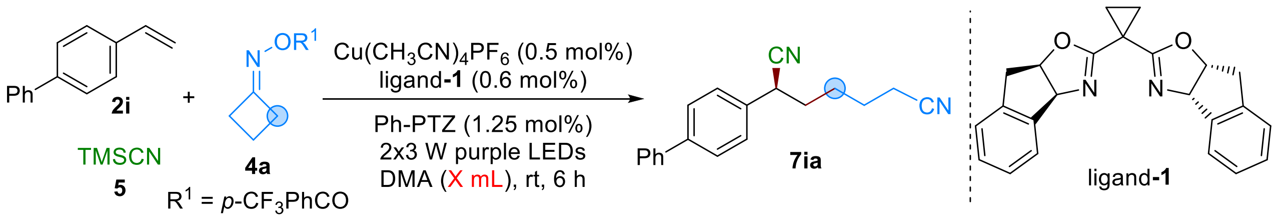

Entry	Concentration (X mL)	Yield [%][b]	ee [%][c]
1	0.5	56	90
2	1	56	90
3	2	69	90
4	4	70	90
5	6	68	90

[a] **2i** (0.2 mmol), **5** (0.6 mmol, 3 equiv), **4a** (0.6 mmol, 3 equiv), $Cu(CH_3CN)_4PF_6$ (0.5 mol%), chiral ligand-**1** (0.6 mol%) and photocatalyst Ph-PTZ (1.25 mol%) in X mL of DMA for 6 h under the irradiation of 2 x 3 W purple LEDs. [b] Determined by GC analysis using 1,3,5-trimethoxybenzene as an internal standard. [c] Determined by chiral HPLC.

As shown in **Table *S11***, among all the tested, DMA (4 mL) gave the best results (70% yield, 90% ee), and was thus selected for further optimization studies

***Table S12*. The Effect of ratio of 2i, 5 and 4a[a]**

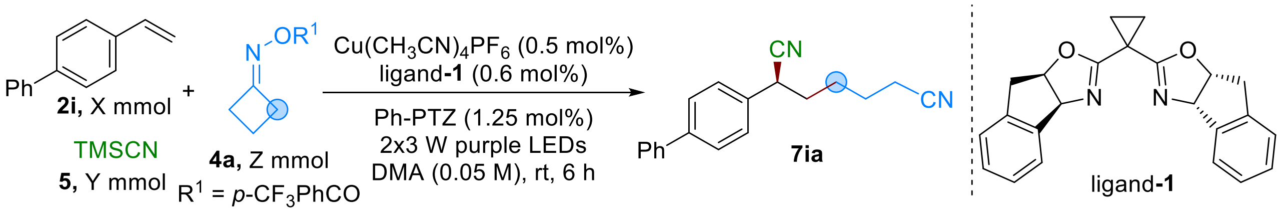

Entry	X	Y	Z	Yield [%][b]	ee [%][c]
1	0.2	0.4	0.4	39	91
2	0.2	0.6	0.6	70	90
3	0.2	0.8	0.8	65	90
4	0.2	1.0	1.0	62	90
5	0.2	1.0	0.6	65	90
6	0.2	0.6	1.0	60	90
7	0.6	0.6	0.2	39	91

[a] **2i** (X mmol), **5** (Y mmol), **4a** (Z mmol), $Cu(CH_3CN)_4PF_6$ (0.5 mol%), chiral ligand-**1** (0.6 mol%) and photocatalyst Ph-PTZ (1.25 mol%) in 4.0 mL of DMA for 6 h under the irradiation of 2 x 3 W purple LEDs. [b] Determined by GC analysis using 1,3,5-trimethoxybenzene as an internal standard. [c] Determined by chiral HPLC.

As shown in **Table *S12***, among all the tested, **2i** (0.2 mmol), **5** (0.6 mmol), **4a** (0.6 mmol) gave the best results (70% yield, 90% ee), and was thus selected for further optimization studies.

***Table S13*. The Effect of Solvents**[a]

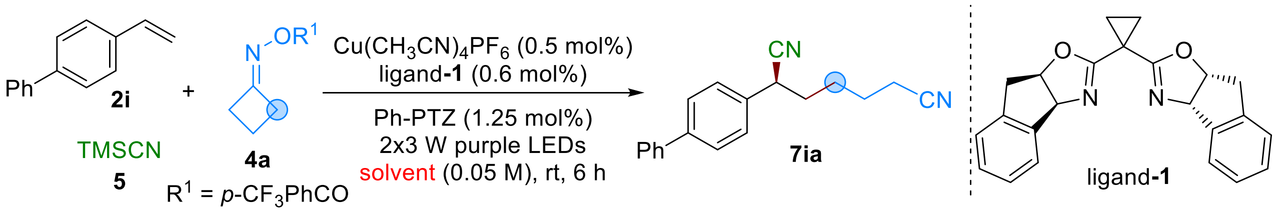

Entry	Solvent	Yield [%][b]	ee [%][c]
1	CH_3CN	15	89
2	DCM	48	89
3	THF	11	93
4	DMSO	35	75
5	DMA	70	90
6	toluene	26	89
7	DMF	56	89

[a] **2i** (0.2 mmol), **5** (0.6 mmol, 3 equiv), **4a** (0.6 mmol, 3 equiv), $Cu(CH_3CN)_4PF_6$ (0.5 mol%), chiral ligand-**1** (0.6 mol%) and photocatalyst Ph-PTZ (1.25 mol%) in 4.0 mL of solvent for 6 h under the irradiation of 2 x 3 W purple LEDs.. [b] Determined by GC analysis using 1,3,5-trimethoxybenzene as an internal standard. [c] Determined by chiral HPLC.

As shown in **Table *S13***, among all the solvents tested, **DMA** gave the best results (70% yield, 90% ee), and was thus selected for further optimization studies.

***Table S14*. The Effect of Copper Salts**[a]

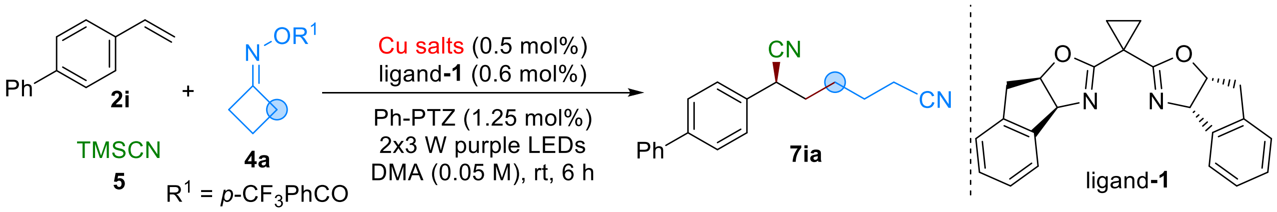

Entry	Cu salt	Yield [%][b]	ee [%][c]
1	CuCl	50	90
2	CuI	51	90
3	$Cu(OAc)_2$	66	90
4	CuCN	55	90
5	$Cu(CH_3CN)_4BF_4$	52	90
6	$Cu(CH_3CN)_4PF_6$	70	90
7	$Cu(OTf)_2$	55	90

[a] **2i** (0.2 mmol), **5** (0.6 mmol, 3 equiv), **4a** (0.6 mmol, 3 equiv), Cu salt (0.5 mol%), chiral ligand-**1** (0.6 mol%) and photocatalyst Ph-PTZ (1.25 mol%) in 4.0 mL of DMA for 6 h under the irradiation of 2 x 3 W purple LEDs.. [b] Determined by GC analysis using 1,3,5-trimethoxybenzene as an internal standard. [c] Determined by chiral HPLC.

As shown in **Table *S14***, among the copper salt tested, $Cu(CH_3CN)_4PF_6$ gave the best result (70% yield, 90% ee) and was thus selected for further studies

***Table S15*. The Effect of Chiral Ligands**[a]

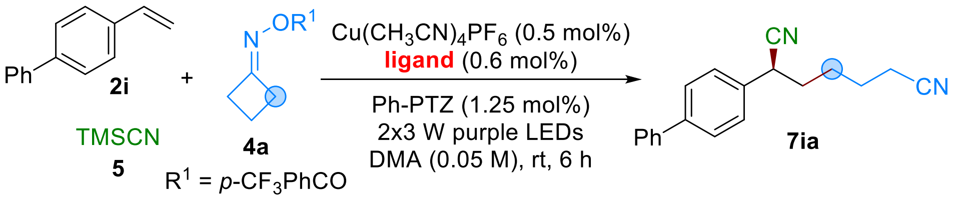

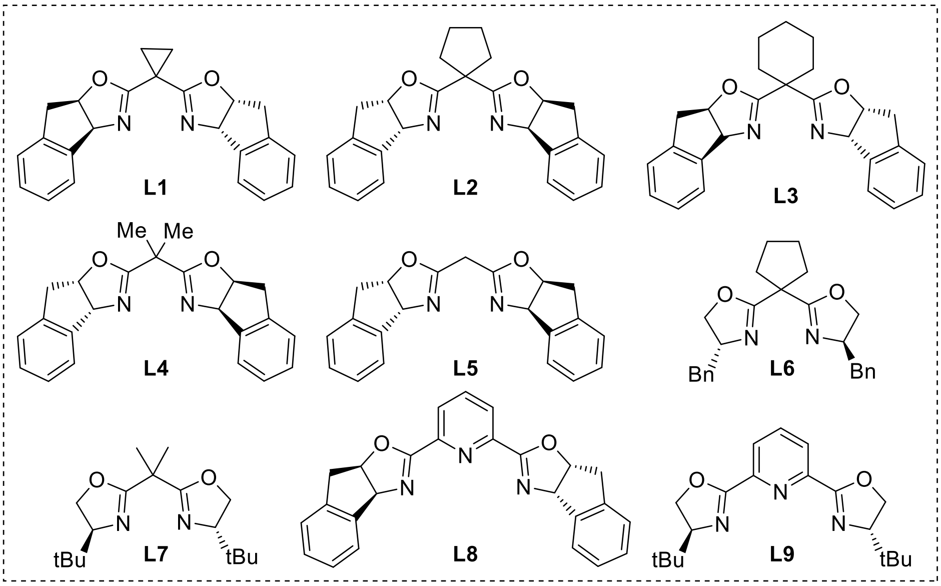

Entry	Chiral Ligand	Yield [%][b]	ee [%][c]
1	**L1**	70	90
2	**L2**	47	-35
3	**L3**	52	9
4	**L4**	48	-9
5	**L5**	43	-40
6	**L6**	39	-16
7	**L7**	51	3
8	**L8**	50	8
9	**L9**	49	10

[a] **2i** (0.2 mmol), **5** (0.6 mmol, 3 equiv), **4a** (0.6 mmol, 3 equiv), $Cu(CH_3CN)_4PF_6$ (0.5 mol%), chiral ligand **L** (0.6 mol%) and photocatalyst Ph-PTZ (1.25 mol%) in 4.0 mL of DMA for 6 h under the irradiation of 2 x 3 W purple LEDs.. [b] Determined by GC analysis using 1,3,5-trimethoxybenzene as an internal standard. [c] Determined by chiral HPLC.

As shown in **Table *S15*,** among all the chiral ligand tested, **L1** gave the best results (70% yield, 90% ee), and was thus selected for further studies.

***Table S16.* Control experiments**[a]

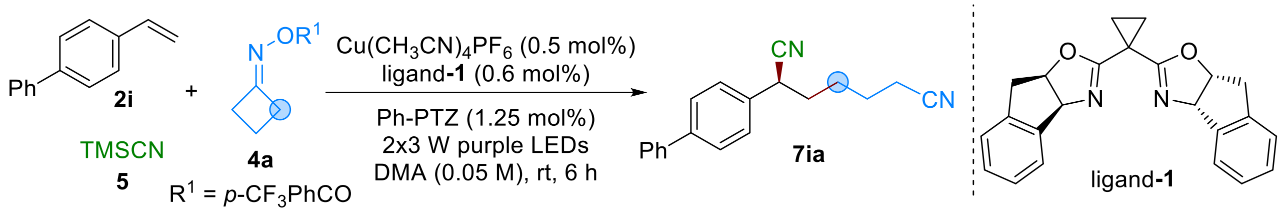

Entry[a]	*hv*	Ph-PTZ	$Cu(CH_3CN)_4PF_6$	Ligand-**1**	Yield [%][b]	ee [%][c]
1[d]	×	√	√	√	21	90
2[e]	√	×	√	√	44	90
3[f]	√	√	×	√	trace	-
4[g]	√	√	√	×	48	0
5	√	√	√	√	70(75)h	90

[a] **2i** (0.2 mmol), **5** (0.6 mmol), **4a** (0.6 mmol), $Cu(CH_3CN)_4PF_6$ (0.5 mol%), chiral ligand-**1** (0.6 mol%) and photocatalyst Ph-PTZ (1.25 mol%) in 4.0 mL of DMA for 6 h under the irradiation of 2 x 3 W purple LEDs. [b] Determined by GC analysis using 1,3,5-trimethoxybenzene as an internal standard. [c] Determined by chiral HPLC. [d] Without hv. [e] Without photocatalyst Ph-PTZ. [f] Without $Cu(CH_3CN)_4PF_6$. [g] Without ligand-**1**. [h] isolated yield in parentheses.

The results of **Table *S16*** reveal that copper and ligand is essential for the reaction, light and photocatalyst can improve the yield.

4. General Procedure and Spectral Data of Products

4.1 General Procedure for Synthesis of 6aa

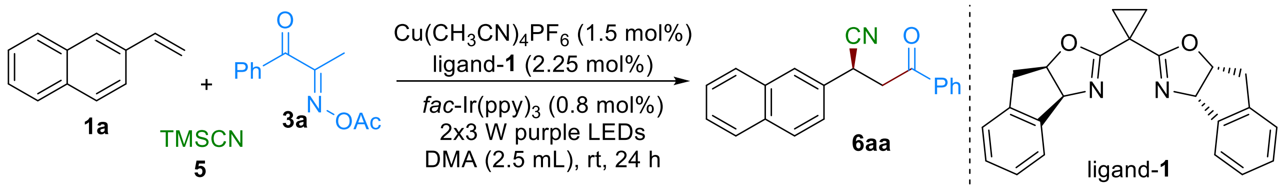

In a flame-dried 10 ml Schlenk tube equipped with a magnetic stirrer bar was charged sequentially with $Cu(CH_3CN)_4PF_6$ (0.56 mg, 0.0015 mmol) and chiral ligand-**1** (0.80 mg, 0.00225 mmol), followed by the addition of DMA (2.5 mL). Then the mixture was stirred at room temperature for 30 min. To the resulting mixture were added **3a** (62 mg, 0.30 mmol), **1a** (15 mg, 0.10 mmol), *fac*-$Ir(ppy)_3$ (0.53 mg, 0.0008 mmol). Then, the resulting mixture was degassed (3 times) under argon atmosphere. After that, TMSCN (0.3 mmol) was added into the mixture. At last, the mixture was stirred at a distance of ~1 cm from a 2 x 3 W purple LEDs at room temperature for 24 h until the reaction was completed, as monitored by TLC analysis. The reaction mixture was quenched with water(10 mL), diluted with EtOAc (3 x 10 mL), washed with NaCl (aq.) and dried over with anhydrous Na_2SO_4. After filtration and concentration, the residue was purified by silica gel chromatography with petroleum ether and ethyl acetate (PE/EA = 5:1) to afford **6aa**.

Note: Some substrates were liquid, which were added into the mixture after degssed. The racemic samples were prepared according to the general procedure by replacing the chiral ligand-**1** with dtbbpy (dtbbpy = 4,4'-di-tert-butyl-2,2'-bipyridine).

4.2 General Procedure for Synthesis of 7ia

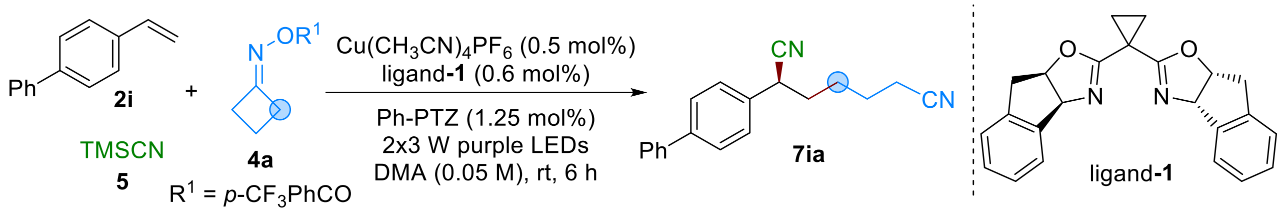

In a flame-dried 10 ml Schlenk tube equipped with a magnetic stirrer bar was charged sequentially with $Cu(CH_3CN)_4PF_6$ (0.001 mmol), ligand-**1** (0.0012 mmol) and organo-photocatalyst Ph-PTZ (0.0025 mmol), followed by the addition of DMA (4 mL). Then the mixture was stirred at room temperature for 30 min. To the resulting mixture were added **2i** (0.20 mmol) and **4a** (0.60 mmol). Then, the resulting mixture was degassed (3 times) under argon atmosphere. After that, TMSCN (0.60 mmol) was added into the mixture. At last, the mixture was stirred at a distance of ~1 cm from a 2 x 3 W purple LEDs at room temperature 6 h until the reaction was completed, as monitored by TLC analysis. The reaction mixture was diluted with water (10 mL). The mixture was firstly extracted with EtOAc (3 x 10 mL), then washed with $NaHCO_3$ (aq.) (15 mL), and finally washed with NaCl (aq.), dried over with anhydrous Na_2SO_4. After filtration and concentration, the residue was purified by silica gel chromatography with petroleum ether and ethyl acetate (PE/EA = 7:1) to afford final product**.**

Note: Some substrates were liquid, which were added into the mixture after degssed. The racemic samples were prepared according to the general procedure by replacing the chiral ligand-**1** with dtbbpy (dtbbpy = 4,4'-di-tert-butyl-2,2'-bipyridine).

4.3 Spectral Data of Products 6 and 7

(S)-2-(naphthalen-2-yl)-4-oxo-4-phenylbutanenitrile (6aa)

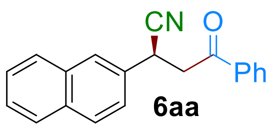

74% isolated yield, white solid, $[\alpha]_D^{25}$ = -24.12 (c = 0.5 in $CHCl_3$); 90% ee, determined by HPLC analysis (Chiralpak AZ column, hexane/*i*-PrOH, 80:20 v/v, flow rate 1.0 mL/min, λ = 220 nm, 25 °C), t_R (major) = 25.28 min, t_R (minor) = 28.73 min. ^{1}H NMR (400 MHz, $CDCl_3$) δ (ppm) 7.99 – 7.91 (m, 3H), 7.86 (dd, *J* = 13.8, 7.2 Hz, 3H), 7.59 (t, *J* = 7.4 Hz, 1H), 7.55 – 7.46 (m, 5H), 4.75 (dd, *J* = 7.9, 6.0 Hz, 1H), 3.81 (dd, *J* = 17.9, 7.9 Hz, 1H), 3.60 (dd, *J* = 17.9, 6.0 Hz, 1H). ^{13}C NMR (100 MHz, $CDCl_3$) δ (ppm) 194.6, 135.7, 133.9, 133.3, 132.9, 132.5, 129.3, 128.8, 128.1, 127.9, 127.7, 126.8, 126.7, 126.7, 124.8, 120.6, 77.3,

77.0, 76.7, 44.5, 32.1. HRMS (EI): m/z [M + Na]$^+$ calcd for $C_{20}H_{15}NNaO$: 308.1046, found: 308.1045.

(*S*)-4-oxo-2,4-diphenylbutanenitrile (6ba)

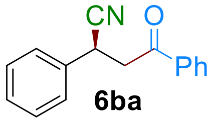

6ba

79% isolated yield, white solid, $[\alpha]_D^{25}$ = -16.30 (c = 0.5 in $CHCl_3$); 90% ee, determined by HPLC analysis (Chiralpak OD column, hexane/*i*-PrOH, 90:10 v/v, flow rate 1.0 mL/min, λ = 254 nm, 25 °C), t_R (major) = 22.46 min, t_R (minor) = 27.81 min. ^{1}H NMR (400 MHz, $CDCl_3$) δ (ppm) 7.93 (d, *J* = 7.2 Hz, 2H), 7.60 (t, *J* = 7.4 Hz, 1H), 7.49 – 7.32 (m, 7H), 4.58 (dd, *J* = 7.9, 6.1 Hz, 1H), 3.73 (dd, *J* = 17.9, 7.9 Hz, 1H), 3.56 – 3.49 (m, 1H). ^{13}C NMR (100 MHz, $CDCl_3$) δ (ppm) 194.6, 135.6, 135.2, 133.9, 129.3, 128.8, 128.4, 128.1, 127.5, 120.6, 77.3, 77.0, 76.7, 44.5, 31.9. HRMS (EI): m/z [M + Na]$^+$ calcd for $C_{16}H_{13}NNaO$: 235.0997, found: 235.0889.

(S)-4-oxo-4-phenyl-2-(p-tolyl)butanenitrile (6ca)

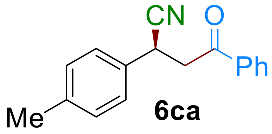

6ca

75% isolated yield, white solid, $[\alpha]_D^{25}$ = -23.00 (c = 0.5 in $CHCl_3$); 86% ee, determined by HPLC analysis (Chiralpak AZ column, hexane/*i*-PrOH, 80:20 v/v, flow rate 1.0 mL/min, λ = 254 nm, 25 °C), t_R (major) = 16.98 min, t_R (minor) = 18.28 min. ^{1}H NMR (400 MHz, $CDCl_3$) δ (ppm) 7.92 (d, *J* = 7.4 Hz, 2H), 7.59 (t, *J* = 7.4 Hz, 1H), 7.47 (t, *J* = 7.7 Hz, 2H), 7.32 (d, *J* = 8.0 Hz, 2H), 7.19 (d, *J* = 7.9 Hz, 2H), 4.58 – 4.47 (m, 1H), 3.71 (dd, *J* = 17.9, 7.9 Hz, 1H), 3.49 (dd, *J* = 17.9, 6.1 Hz, 1H), 2.35 (s, 3H). ^{13}C NMR (100 MHz, $CDCl_3$) δ (ppm) 194.7, 138.2, 135.7, 133.9, 132.2, 129.9, 128.8, 128.1, 127.3, 120.8, 77.3, 77.0, 76.7, 44.6, 31.5, 21.1. HRMS (EI): m/z [M + Na]$^+$ calcd for $C_{17}H_{15}NNaO$: 272.1046, found: 272.1046.

(S)-2-(4-(tert-butyl)phenyl)-4-oxo-4-phenylbutanenitrile (6da)

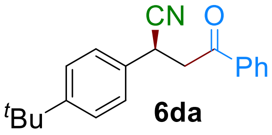

6da

73% isolated yield, white solid, $[\alpha]_D^{25}$ = -19.21 (c = 0.5 in $CHCl_3$); 86% ee, determined by HPLC analysis (Chiralpak AZ column, hexane/*i*-PrOH, 90:10 v/v, flow rate 1.0 mL/min, λ = 254 nm, 25 °C), t_R (major) = 20.23 min, t_R (minor) = 24.59 min. ^{1}H NMR (400 MHz, $CDCl_3$) δ (ppm) 8.01 – 7.86 (m, 2H), 7.62 – 7.33 (m, 7H), 4.54 (dd, *J* = 8.0, 6.0 Hz, 1H), 3.72 (dd, *J* = 18.0, 8.0 Hz, 1H), 3.50 (dd, *J* = 18.0, 6.0 Hz, 1H), 1.31 (s, 9H). ^{13}C NMR (100 MHz, $CDCl_3$) δ (ppm) 194.7, 151.4, 135.7, 133.8, 132.1, 128.8, 128.1, 127.1, 126.2, 120.8, 77.3, 77.0, 76.7, 44.5, 34.6, 31.4, 31.2. HRMS (EI): m/z [M + Na]$^+$ calcd for $C_{20}H_{21}NNaO$: 314.1515, found: 314.1515.

(S)-2-([1,1'-biphenyl]-4-yl)-4-oxo-4-phenylbutanenitrile (6ea)

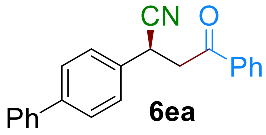

6ea

79% isolated yield, white solid, $[\alpha]_D^{25}$ = +1.2 (c = 0.5 in $CHCl_3$); 89% ee, determined by HPLC analysis (Chiralpak AZ column, hexane/*i*-PrOH, 80:20 v/v, flow rate 1.0 mL/min, λ = 254 nm, 25 °C), t_R (major) = 26.54 min, t_R (minor) = 33.81 min. ^{1}H NMR (400 MHz, $CDCl_3$) δ (ppm) 7.95 (d, *J* = 8.0 Hz, 2H), 7.59 (dd, *J* = 16.7, 8.0 Hz, 5H), 7.53 – 7.42 (m, 6H), 7.37 (t, *J* = 7.2 Hz, 1H), 4.62 (t, *J*

= 6.8 Hz, 1H), 3.77 (dd, *J* = 17.9, 7.8 Hz, 1H), 3.56 (dd, *J* = 18.0, 6.1 Hz, 1H). ^{13}C NMR (100 MHz, $CDCl_3$) δ (ppm) 194.6, 141.4, 140.1, 135.6, 134.2, 133.9, 128.9, 128.8, 128.1, 127.9, 127.9, 127.7, 127.1, 120.6, 77.3, 77.0, 76.7, 44.5, 31.6. HRMS (EI): m/z [M + Na]$^+$ calcd for $C_{22}H_{17}NNaO$: 334.1202, found: 334.1202.

(S)-2-(4-fluorophenyl)-4-oxo-4-phenylbutanenitrile (6fa)

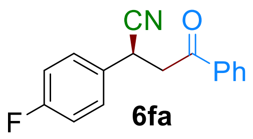

6fa

64% isolated yield, white solid, $[\alpha]_D^{25}$ = -17.40 (c = 0.5 in $CHCl_3$); 87% ee, determined by HPLC analysis (Chiralpak AZ column, hexane/*i*-PrOH, 90:10 v/v, flow rate 0.5 mL/min, λ = 254 nm, 25 °C), t_R (major) = 51.07 min, t_R (minor) = 54.02 min. ^{1}H NMR (400 MHz, $CDCl_3$) δ (ppm) 7.98 – 7.83 (m, 2H), 7.60 (t, *J* = 7.4 Hz, 1H), 7.52 – 7.38 (m, 4H), 7.07 (t, *J* = 7.6 Hz, 2H), 4.56 (t, *J* = 6.9 Hz, 1H), 3.71 (dd, *J* = 18.0, 7.5 Hz, 1H), 3.51 (dd, *J* = 17.9, 6.4 Hz, 1H). ^{13}C NMR (100 MHz, $CDCl_3$) δ (ppm) 194.4, 162.4 (d, *J* = 246.4 Hz),135.5, 133.9, 131.0 (d, *J* = 3.3 Hz), 129.3, (d, *J* = 8.2 Hz), 128.8, 128.0, 120.5, 116.2 (d, *J* = 2.2 Hz), 77.3, 77.0, 76.7, 44.4, 31.1. ^{19}F NMR (376 MHz, CDCl3) δ (ppm) -113.2. HRMS (EI): m/z [M + Na]$^+$ calcd for $C_{16}H_{12}FNNaO$: 276.0795, found: 276.0795.

(S)-2-(4-chlorophenyl)-4-oxo-4-phenylbutanenitrile (6ga)

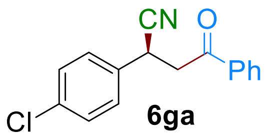

6ga

73% isolated yield, white solid, $[\alpha]_D^{25}$ = -16.82 (c = 0.5 in $CHCl_3$); 90% ee, determined by HPLC analysis (Chiralpak AZ column, hexane/*i*-PrOH, 85:15 v/v, flow rate 1.0 mL/min, λ = 254 nm, 25 °C), t_R (major) = 21.21 min, t_R (minor) = 22.82 min. ^{1}H NMR (400 MHz, $CDCl_3$) δ (ppm) 7.91 (dd, *J* = 8.4, 1.4 Hz, 2H), 7.60 (t, *J* = 7.4 Hz, 1H), 7.47 (t, *J* = 7.7 Hz, 2H), 7.42 – 7.32 (m, 4H), 4.56 (t, *J* = 6.9 Hz, 1H), 3.71 (dd, *J* = 17.9, 7.4 Hz, 1H), 3.51 (dd, *J* = 17.9, 6.5 Hz, 1H). ^{13}C NMR (100 MHz, $CDCl_3$) δ (ppm) 194.3, 135.5, 134.4, 134.0, 133.7, 129.4, 128.9, 128.8, 128.0, 120.2, 77.3, 77.0, 76.7, 44.2, 31.3. HRMS (EI): m/z [M + K]$^+$ calcd for $C_{16}H_{12}ClKNO$: 308.0239, found: 308.0240.

(S)-2-(4-bromophenyl)-4-oxo-4-phenylbutanenitrile (6ha)

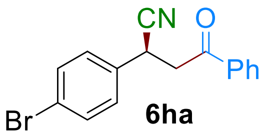

6ha

76% isolated yield, white solid, $[\alpha]_D^{25}$ = -11.71 (c = 0.5 in $CHCl_3$); 87% ee, determined by HPLC analysis (Chiralpak AZ column, hexane/*i*-PrOH, 80:20 v/v, flow rate 1.0 mL/min, λ = 254 nm, 25 °C), t_R (major) = 18.42 min, t_R (minor) = 20.15 min. ^{1}H NMR (400 MHz, $CDCl_3$) δ (ppm) 7.96 – 7.87 (m, 2H), 7.61 (t, *J* = 7.4 Hz, 1H), 7.56 – 7.44 (m, 4H), 7.32 (d, *J* = 8.4 Hz, 2H), 4.55 (t, *J* = 6.9 Hz, 1H), 3.71 (dd, *J* = 17.9, 7.4 Hz, 1H), 3.51 (dd, *J* = 18.0, 6.5 Hz, 1H). ^{13}C NMR (100 MHz, $CDCl_3$) δ (ppm) 194.3, 135.5, 134.3, 134.0, 132.4, 129.2, 128.9, 128.1, 122.5, 120.1, 77.3, 77.0, 76.7, 44.2, 31.4. HRMS (EI): m/z [M + Na]$^+$ calcd for $C_{16}H_{12}BrNNaO$: 335.9994, found: 335.9999.

(S)-4-(1-cyano-3-oxo-3-phenylpropyl)phenyl acetate (6ia)

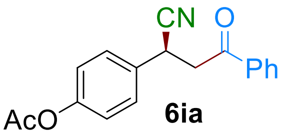

6ia

67% isolated yield, white solid, $[\alpha]_D^{25}$ = -16.31 (c = 0.5 in $CHCl_3$); 89% ee, determined by HPLC analysis (Chiralpak AZ column, hexane/*i*-PrOH, 80:20 v/v, flow rate 1.0 mL/min, λ = 254 nm, 25 °C), t_R (major) = 36.66 min, t_R (minor) = 45.46 min. ^{1}H NMR (400 MHz, $CDCl_3$) δ (ppm) 7.96 – 7.84 (m, 2H), 7.59 (t, *J* = 7.4 Hz, 1H), 7.52 – 7.43 (m, 4H), 7.11 (d, *J* = 8.6 Hz, 2H), 4.56 (dd, *J* = 7.9, 6.0 Hz, 1H), 3.71 (dd, *J* = 18.0, 7.9 Hz, 1H), 3.49 (dd, *J* = 18.0, 6.0 Hz, 1H), 2.29 (s, 3H). ^{13}C NMR (100 MHz, $CDCl_3$) δ (ppm) 194.4, 169.2, 150.4, 135.5, 133.9, 132.7, 128.8, 128.6, 128.0, 122.4, 120.4, 77.3, 77.0, 76.7, 44.4, 31.2, 21.0. HRMS (EI): m/z $[M + Na]^+$ calcd for $C_{18}H_{15}NNaO_3$: 316.0944, found: 316.0942.

(S)-4-oxo-4-phenyl-2-(4-(4,4,5,5-tetramethyl-1,3,2-dioxaborolan-2-yl)phenyl)butanenitrile (6ja)

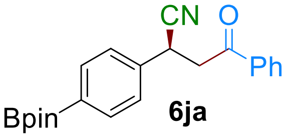

6ja

76% isolated yield, white solid, $[\alpha]_D^{25}$ = -24.36 (c = 0.5 in $CHCl_3$); 86% ee, determined by HPLC analysis (Chiralpak AZ column, hexane/*i*-PrOH, 95:5 v/v, flow rate 1.0 mL/min, λ = 254 nm, 25 °C), t_R (major) = 37.85 min, t_R (minor) = 58.23 min. ^{1}H NMR (400 MHz, $CDCl_3$) δ (ppm) 7.87 (dd, *J* = 32.6, 8.3 Hz, 4H), 7.63 – 7.39 (m, 5H), 4.63 – 4.54 (m, 1H), 3.72 (dd, *J* = 17.9, 8.0 Hz, 1H), 3.49 (dd, *J* = 17.9, 5.9 Hz, 1H), 1.34 (s, 11H). ^{13}C NMR (100 MHz, $CDCl_3$) δ (ppm) 194.5, 138.1, 135.7, 133.9, 128.8, 128.1, 126.8, 120.4, 84.0, 77.3, 77.0, 76.7, 44.5, 32.1, 24.9. HRMS (EI): m/z $[M + K]^+$ calcd for $C_{22}H_{24}BNKO_3$: 400.1481, found: 400.1472.

(S)-2-(4-methoxyphenyl)-4-oxo-4-phenylbutanenitrile (6ka)

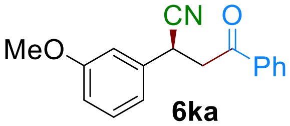

6ka

81% isolated yield, white solid, $[\alpha]_D^{25}$ = -28.83 (c = 0.5 in $CHCl_3$); 89% ee, determined by HPLC analysis (Chiralpak OD column, hexane/*i*-PrOH, 80:20 v/v, flow rate 1.0 mL/min, λ = 254 nm, 25 °C), t_R (major) = 19.74 min, t_R (minor) = 23.73 min. ^{1}H NMR (400 MHz, $CDCl_3$) δ (ppm) 8.00 – 7.83 (m, 2H), 7.59 (t, *J* = 7.4 Hz, 1H), 7.46 (t, *J* = 7.7 Hz, 2H), 7.29 (q, *J* = 8.0 Hz, 1H), 7.04 – 6.94 (m, 2H), 6.86 (dd, *J* = 8.3, 2.3 Hz, 1H), 7.06 – 6.81 (m, 3H), 4.53 (dd, *J* = 8.0, 5.9 Hz, 1H), 3.81 (s, 3H), 3.72 (dd, *J* = 17.9, 8.1 Hz, 1H), 3.50 (dd, *J* = 17.9, 5.8 Hz, 1H). ^{13}C NMR (100 MHz, $CDCl_3$) δ (ppm) 194.6, 160.1, 136.6, 135.6, 133.8, 130.3, 128.8, 128.0, 120.5, 119.6, 113.7, 113.2, 77.3, 77.0, 76.7, 55.3, 44.4, 31.8. HRMS (EI): m/z $[M + H]^+$ calcd for $C_{17}H_{16}NHO_2$: 266.1176, found: 266.1175.

(S)-4-oxo-4-phenyl-2-(p-tolyl)butanenitrile (6la)

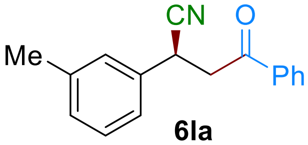

6la

68% isolated yield, white solid, $[\alpha]_D^{25}$ = -13.92 (c = 0.5 in $CHCl_3$); 90% ee, determined by HPLC analysis (Chiralpak OD column, hexane/*i*-PrOH, 85:15 v/v, flow rate 1.0 mL/min, λ = 254 nm, 25 °C), t_R (major) = 12.54 min, t_R (minor) = 15.88 min. ^{1}H NMR (400 MHz, $CDCl_3$) δ (ppm) 7.93 (d, *J* = 8.4 Hz, 2H), 7.59 (t, *J* = 7.4 Hz, 1H), 7.47 (t, *J* = 7.7 Hz, 2H), 7.29 – 7.20 (m,

3H), 7.14 (d, *J* = 7.4 Hz, 1H), 7.33 – 7.14 (m, 4H), 4.52 (dd, *J* = 8.2, 5.8 Hz, 1H), 3.72 (dd, *J* = 18.0, 8.2 Hz, 1H), 3.49 (dd, *J* = 17.9, 5.8 Hz, 1H), 2.37 (s, 3H). ^{13}C NMR (100 MHz, $CDCl_3$) δ (ppm) 194.7, 139.1, 135.7, 135.1, 133.8, 129.1, 129.1, 128.8, 128.1, 128.1, 124.5, 120.7, 77.3, 77.0, 76.7, 44.6, 31.8, 21.3. H$_R$MS (EI): m/z [M + Na]$^+$ calcd for $C_{17}H_{15}NNaO$: 272.1046, found: 272.1049.

(S)-2-(4-fluorophenyl)-4-oxo-4-phenylbutanenitrile (6ma)

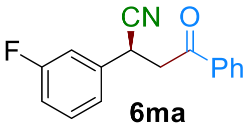

6ma

65% isolated yield, white solid, $[\alpha]_D^{25}$ = -16.47 (c = 0.5 in $CHCl_3$); 89% ee, determined by HPLC analy sis (Chiralpak OD column, hexane/*i*-PrOH, 90:10 v/v, flow rate 1.0 mL/min, λ = 254 nm, 25 °C), t_R (major) = 24.29 min, t_R (minor) = 33.28 min. ^{1}H NMR (400 MHz, $CDCl_3$) δ (ppm) 7.99 – 7.85 (m, 2H), 7.61 (t, *J* = 7.4 Hz, 1H), 7.48 (t, *J* = 7.7 Hz, 2H), 7.37 (td, *J* = 8.0, 6.0 Hz, 1H), 7.29 – 7.13 (m, 2H), 7.09 – 7.00 (m, 1H), 4.58 (dd, *J* = 7.6, 6.2 Hz, 1H), 3.73 (dd, *J* = 18.0, 7.6 Hz, 1H), 3.52 (dd, *J* = 18.0, 6.2 Hz, 1H). ^{13}C NMR (100 MHz, $CDCl_3$) δ (ppm) 194.2, 164.2, (d, *J* = 246.5 Hz), 137.5 (d, *J* = 7.37 Hz),135.5, 134.0, 130.9 (d, *J* = 8.3 Hz), 128.9, 128.1, 123.2, 123.2, 120.1, 115.6 (d, *J* = 20.8 Hz), 77.3, 77.0, 76.7, 44.2, 31.5. ^{19}F NMR (376 MHz, CDCl3) δ (ppm) -111.1. HRMS (EI): m/z [M + Na]$^+$ calcd for $C_{16}H_{12}FNNaO$: 276.0795, found: 276.0796.

(S)-4-oxo-4-phenyl-2-(o-tolyl)butanenitrile (6na)

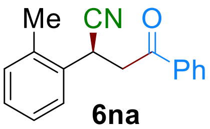

6na

61% isolated yield, white solid, $[\alpha]_D^{25}$ = -34.29 (c = 0.5 in $CHCl_3$); 89% ee, determined by HPLC analysis (Chiralpak AZ column, hexane/*i*-PrOH, 85:15 v/v, flow rate 1.0 mL/min, λ = 210 nm, 25 °C), t_R (major) = 18.80 min, t_R (minor) = 21.09 min. ^{1}H NMR (400 MHz, $CDCl_3$) δ (ppm) 8.00 – 7.88 (m, 2H), 7.61 (t, *J* = 7.4 Hz, 1H), 7.55 – 7.43 (m, 3H), 7.31 – 7.14 (m, 3H), 4.71 (dd, *J* = 9.0, 5.0 Hz, 1H), 3.74 (dd, *J* = 18.0, 9.0 Hz, 1H), 3.42 (dd, *J* = 18.0, 5.0 Hz, 1H), 2.42 (s, 3H). ^{13}C NMR (100 MHz, $CDCl_3$) δ (ppm) 194.8, 135.7, 135.3, 133.9, 133.4, 131.3, 128.9, 128.5, 128.1, 127.5, 127.0, 77.4, 77.0, 76.7, 43.1, 28.7, 19.3. HRMS (EI): m/z [M + Na]$^+$ calcd for $C_{17}H_{15}NNaO$: 272.1046, found: 272.1049.

(S)-2-(2-bromophenyl)-4-oxo-4-phenylbutanenitrile (6oa)

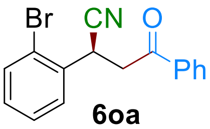

6oa

63% isolated yield, white solid, $[\alpha]_D^{25}$ = -24.13 (c = 0.5 in $CHCl_3$); 90% ee, determined by HPLC analysis (Chiralpak AZ column, hexane/*i*-PrOH, 85:15 v/v, flow rate 1.0 mL/min, λ = 210 nm, 25 °C), t_R (major) = 19.70 min, t_R (minor) = 23.89 min. ^{1}H NMR (400 MHz, $CDCl_3$) δ (ppm) 7.99 – 7.92 (m, 2H), 7.71 (d, *J* = 9.2 Hz, 1H), 7.61 (t, *J* = 8.0 Hz, 2H), 7.48 (t, *J* = 7.7 Hz, 2H), 7.41 (t, *J* = 7.6 Hz, 1H), 7.23 (d, *J* = 9.1 Hz, 1H), 4.93 (dd, *J* = 9.6, 4.3 Hz, 1H), 3.71 – 3.63 (m, 1H), 3.53 (dd, *J* = 18.0, 4.3 Hz, 1H). ^{13}C NMR (100 MHz, $CDCl_3$) δ (ppm) 194.4, 135.6, 134.4, 133.9, 133.6, 130.1, 129.5, 128.9, 128.4, 128.1, 122.9, 119.8, 77.3, 77.0, 76.7, 42.8, 32.5. HRMS (EI): m/z [M + Na]$^+$ calcd for $C_{16}H_{12}BrNNaO$: 335.9994, found: 335.9999.

(S)-2-(6-methoxynaphthalen-2-yl)-4-oxo-4-phenylbutanenitrile (6pa)

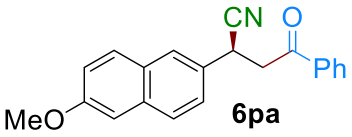

6pa

84% isolated yield, white solid, $[\alpha]_D^{25}$ = -30.81 (c = 0.5 in $CHCl_3$); 88% ee, determined by HPLC analysis (Chiralpak AZ column, hexane/*i*-PrOH, 80:20 v/v, flow rate 1.0 mL/min, λ = 220 nm, 25 °C), t_R (major) = 34.56 min, t_R (minor) = 39.40 min. ^{1}H NMR (400 MHz, $CDCl_3$) δ (ppm) 8.01 – 7.67 (m, 5H), 7.52 (dt, *J* = 50.6, 7.5 Hz, 4H), 7.22 – 7.04 (m, 2H), 4.69 (t, *J* = 6.9 Hz, 1H), 3.91 (s, 3H), 3.78 (dd, *J* = 17.9, 7.9 Hz, 1H), 3.58 (dd, *J* = 17.9, 6.0 Hz, 1H). ^{13}C NMR (100 MHz, $CDCl_3$) δ (ppm) 194.7, 158.2, 135.7, 134.1, 133.8, 130.1, 129.3, 128.8, 128.7, 128.1, 128.0, 126.5, 125.3, 120.8, 119.6, 105.6, 77.3, 77.0, 76.7, 55.3, 44.5, 31.9. HRMS (EI): m/z $[M + Na]^+$ calcd for $C_{21}H_{17}NNaO_2$: 338.1151, found: 338.1148.

((S)-2-(benzo[d][1,3]dioxol-5-yl)-4-oxo-4-phenylbutanenitrile (6qa)

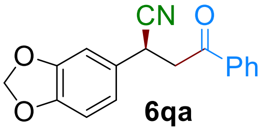

6qa

76% isolated yield, white solid, $[\alpha]_D^{25}$ = -15.00 (c = 0.5 in $CHCl_3$); 86% ee, determined by HPLC analysis (Chiralpak OD column, hexane/*i*-PrOH, 90:10 v/v, flow rate 1.0 mL/min, λ = 254 nm, 25 °C), t_R (major) = 58.40 min, t_R (minor) = 45.21 min. ^{1}H NMR (400 MHz, $CDCl_3$) δ (ppm) 7.98 – 7.87 (m, 2H), 7.60 (s, 1H), 7.48 (d, *J* = 7.9 Hz, 2H), 6.94 – 6.86 (m, 2H), 6.85 – 6.75 (m, 1H), 5.97 (s, 2H), 4.54 – 4.42 (m, 1H), 3.68 (dd, *J* = 17.9, 7.6 Hz, 1H), 3.49 (dd, *J* = 17.9, 6.3 Hz, 1H). ^{13}C NMR (100 MHz, $CDCl_3$) δ (ppm) 194.6, 148.3, 147.6, 135.6, 133.9, 128.8, 128.8, 128.0, 121.0, 120.7, 108.7, 107.9, 101.4, 77.3, 77.0, 76.7, 44.5, 31.5. HRMS (EI): m/z $[M + Na]^+$ calcd for $C_{17}H_{13}NNaO_3$: 302.0788, found: 302.0786.

(R)-4-oxo-4-phenyl-2-(thiophen-2-yl)butanenitrile (6ra)

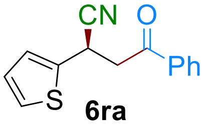

6ra

64% isolated yield, white solid, $[\alpha]_D^{25}$ = -18.00 (c = 0.5 in $CHCl_3$); 91% ee, determined by HPLC analysis (Chiralpak OD column, hexane/*i*-PrOH, 85:15 v/v, flow rate 1.0 mL/min, λ = 254 nm, 25 °C), t_R (major) = 19.65 min, t_R (minor) = 26.26 min. ^{1}H NMR (400 MHz, $CDCl_3$) δ (ppm) 7.95 (d, *J* = 7.3 Hz, 2H), 7.62 (t, *J* = 7.4 Hz, 1H), 7.49 (t, *J* = 7.7 Hz, 2H), 7.32 – 7.22 (m, 1H), 7.17 (d, *J* = 3.4 Hz, 1H), 7.05 – 6.95 (m, 1H), 4.87 (t, *J* = 6.8 Hz, 1H), 3.77 (dd, *J* = 17.9, 7.3 Hz, 1H), 3.63 (dd, *J* = 17.9, 6.5 Hz, 1H). ^{13}C NMR (100 MHz, $CDCl_3$) δ (ppm) 194.2, 136.9, 135.5, 134.0, 128.9, 128.1, 127.2, 126.7, 125.9, 119.7, 77.3, 77.0, 76.7, 44.5, 27.2.

HRMS (EI): m/z $[M + Na]^+$ calcd for $C_{14}H_{11}NNaOS$: 264.0454, found: 264.0448.

(S)-4-oxo-4-phenyl-2-(1-tosyl-1H-indol-2-yl)butanenitrile (6sa)

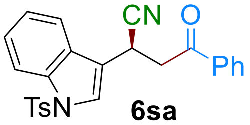

6sa

71% isolated yield, white solid, $[\alpha]_D^{25}$ = -17.46 (c = 0.5 in $CHCl_3$); 93% ee, determined by HPLC analysis (Chiralpak OD column, hexane/*i*-PrOH, 80:20 v/v, flow rate 1.0 mL/min, λ = 254 nm, 25 °C), t_R (major) = 47.30 min, t_R (minor) = 54.96 min. ^{1}H NMR (400 MHz, $CDCl_3$) δ (ppm) 8.01 (d, *J* = 8.3 Hz, 1H), 7.93 (d, *J* = 8.0 Hz, 2H), 7.77 (d, *J* = 8.3 Hz, 2H), 7.69 (s, 1H), 7.62 (t, *J* = 8.5 Hz, 2H), 7.48 (t, *J* = 7.7 Hz, 2H), 7.38 (t, *J* = 7.7 Hz, 1H), 7.30 (t, *J* = 7.6 Hz, 1H), 7.23 (d, *J* = 8.2 Hz, 2H), 4.77 (dd, *J* = 8.0, 5.7

Hz, 1H), 3.77 (dd, J = 17.9, 8.1 Hz, 1H), 3.60 (dd, J = 17.9, 5.6 Hz, 1H), 2.34 (s, 3H). ^{13}C NMR (100 MHz, $CDCl_3$) δ (ppm) 194.4, 135.5, 134.1, 130.1, 128.9, 128.1, 126.9, 125.5, 124.5, 123.7, 119.1, 116.0, 114.1, 77.3, 77.0, 76.7, 42.0, 23.4, 21.6. HRMS (EI): m/z [M + Na]$^+$ calcd for $C_{25}H_{20}N_3NaO_3S$: 451.1087, found: 451.1083.

(2S)-2-((8R,9S,13S)-13-methyl-17-oxo-7,8,9,11,12,13,14,15,16,17-decahydro-6H-cyclopenta[a]phenanthren-3-yl)-4-oxo-4-phenylbutanenitrile (6ta)

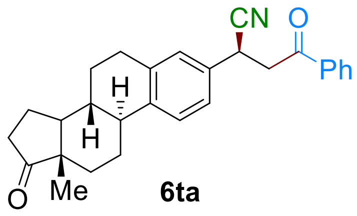

6ta

56% isolated yield, white solid, $[\alpha]_D^{25}$ = -18.06 (c = 0.5 in $CHCl_3$); 93:7 d.r., determined by HPLC analysis (Chiralpak OD column, hexane/*i*-PrOH, 70:30 v/v, flow rate 1.0 mL/min, λ = 220 nm, 25 ºC), t_R (major) = 27.80 min, t_R (minor) = 36.02 min. ^{1}H NMR (400 MHz, $CDCl_3$) δ (ppm) 7.93 (d, J = 7.6 Hz, 2H), 7.59 (t, J = 7.4 Hz, 1H), 7.47 (t, J = 7.7 Hz, 2H), 7.30 (d, J = 8.0 Hz, 1H), 7.19 (d, J = 10.9 Hz, 2H), 4.55 – 4.43 (m, 1H), 3.71 (dd, J = 18.0, 8.1 Hz, 1H), 3.50 (dd, J = 18.0, 5.9 Hz, 1H), 2.98 – 2.86 (m, 2H), 2.59 – 2.38 (m, 2H), 2.28 (t, J = 10.0 Hz, 1H), 2.23 – 1.90 (m, 4H), 1.70 – 1.35 (m, 6H), 0.91 (s, 3H). ^{13}C NMR (100 MHz, $CDCl_3$) δ (ppm) 194.6, 140.0, 137.5, 135.6, 133.8, 132.5, 128.7, 128.0, 127.9, 126.2, 124.7, 124.7, 120.7, 77.3, 77.0, 76.7, 50.3, 47.8, 44.4, 44.4, 44.1, 37.9, 35.7, 31.4, 31.3, 31.3, 29.2, 29.2, 26.2, 25.6, 21.5, 13.7. HRMS (EI): m/z [M + Na]$^+$ calcd for $C_{28}H_{29}NNaO_2$: 434.2091, found: 434.2091.

ethyl (S)-2-(3-(1-cyano-3-oxo-3-phenylpropyl)-4-isobutoxyphenyl)-4-methylthiazole-5-carboxylate (6ua)

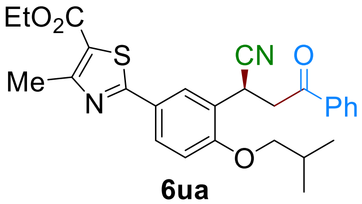

6ua

53% isolated yield, white solid, $[\alpha]_D^{25}$ = -17.46 (c = 0.5 in $CHCl_3$); 82% ee, determined by HPLC analysis (Chiralpak AD column, hexane/*i*-PrOH, 90:10 v/v, flow rate 1.0 mL/min, λ = 220 nm, 25 ºC), t_R (major) = 20.79 min, t_R (minor) = 25.09 min. ^{1}H NMR (400 MHz, $CDCl_3$) δ (ppm) 8.11 (s, 1H), 7.93 (dd, J = 15.6, 8.1 Hz, 3H), 7.60 (t, J = 7.3 Hz, 1H), 7.48 (t, J = 7.2 Hz, 2H), 6.96 (d, J = 8.4 Hz, 1H), 4.92 – 4.82 (m, 1H), 4.35 (q, J = 6.7 Hz, 2H), 3.87 (d, J = 5.9 Hz, 2H), 3.77 (dd, J = 17.8, 8.8 Hz, 1H), 3.55 (dd, J = 17.9, 3.6 Hz, 1H), 2.77 (s, 3H), 2.16 (dt, J = 12.6, 6.3 Hz, 1H), 1.39 (t, J = 6.6 Hz, 3H), 1.05 (t, J = 6.8 Hz, 6H). ^{13}C NMR (100 MHz, $CDCl_3$) δ (ppm) 194.9, 168.8, 162.2, 161.0, 157.9, 135.7, 133.7, 128.8, 128.5, 128.0, 127.6, 126.0, 124.0, 121.2, 120.0, 111.9, 77.3, 77.0, 76.7, 75.1, 61.2, 41.8, 28.2, 27.5, 19.3, 19.2, 17.5, 14.3. HRMS (EI): m/z [M + H]$^+$ calcd for $C_{27}H_{29}N_2O_4S$: 477.1843, found: 477.1841.

methyl (R)-2-((tert-butoxycarbonyl)amino)-3-(4-((S)-1-cyano-3-oxo-3-phenylpropyl)phenyl)propanoate (6va)

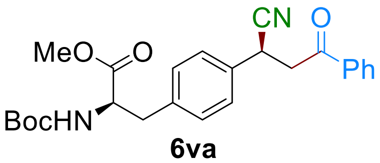

6va

61% isolated yield, yellow oil, $[\alpha]_D^{25}$ = -19.26 (c = 0.5 in $CHCl_3$); 93:7 d.r., determined by HPLC analysis (Chiralpak AD column, hexane/*i*-PrOH, 70:30 v/v, flow rate 1.0 mL/min, λ = 254 nm, 25 ºC), t_R (major) = 28.42 min, t_R (minor) =

24.78 min. ^{1}H NMR (400 MHz, $CDCl_3$) δ (ppm) 7.92 (d, *J* = 7.6 Hz, 2H), 7.59 (t, *J* = 7.3 Hz, 1H), 7.49 – 7.31 (m, 4H), 7.16 (d, *J* = 6.4 Hz, 2H), 5.07 (d, *J* = 7.3 Hz, 1H), 4.55 (dt, *J* = 13.8, 6.9 Hz, 2H), 3.71 (s, 4H), 3.49 (dd, *J* = 18.0, 5.6 Hz, 1H), 3.08 (dt, *J* = 32.5, 10.1 Hz, 2H), 1.40 (s, 10H). ^{13}C NMR (100 MHz, $CDCl_3$) δ (ppm) 194.5, 172.0, 154.9, 136.4, 136.4, 135.5, 133.8, 133.8, 130.1, 128.7, 127.9, 127.5, 120.5, 79.9, 77.3, 77.0, 76.7, 54.2, 52.2, 52.2, 44.3, 37.8, 37.7, 31.4, 28.1. HRMS (EI): m/z $[M + Na]^+$ calcd for $C_{25}H_{28}N_2NaO_5$: 459.1890, found: 459.1891.

(*S*)-4-oxo-2-phenyl-4-(p-tolyl)butanenitrile (6bb)

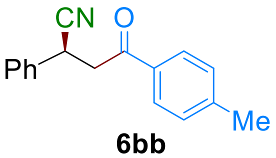

6bb

73% isolated yield, white solid, $[\alpha]_D^{25}$ = -20.00 (c = 0.5 in $CHCl_3$); 83% ee, determined by HPLC analysis (Chiralpak AZ column, hexane/*i*-PrOH, 85:15 v/v, flow rate 1.0 mL/min, λ = 254 nm, 25 °C), t_R (major) = 45.75 min, t_R (minor) = 48.83 min. ^{1}H NMR (400 MHz, $CDCl_3$) δ (ppm) 7.82 (d, *J* = 8.2 Hz, 2H), 7.46 – 7.30 (m, 5H), 7.25 (d, *J* = 3.9 Hz, 1H), 4.56 (dd, *J* = 7.9, 6.1 Hz, 1H), 3.70 (dd, *J* = 17.8, 8.0 Hz, 1H), 3.48 (dd, *J* = 17.8, 6.0 Hz, 1H), 2.41 (s, 3H). ^{13}C NMR (100 MHz, $CDCl_3$) δ (ppm) 194.2, 144.9, 135.3, 133.2, 129.5, 129.2, 128.3, 128.2, 127.4, 120.7, 77.3, 77.0, 76.7, 44.4, 31.9, 21.7. HRMS (EI): m/z $[M + Na]^+$ calcd for $C_{17}H_{15}NNaO$: 272.1046, found: 272.1046.

(*S*)-4-(4-methoxyphenyl)-4-oxo-2-phenylbutanenitrile (6bc)

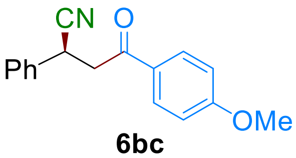

6bc

75% isolated yield, white solid, $[\alpha]_D^{25}$ = -18.18 (c = 0.5 in $CHCl_3$); 86% ee, determined by HPLC analysis (Chiralpak AD column, hexane/*i*-PrOH, 85:15 v/v, flow rate 1.0 mL/min, λ = 254 nm, 25 °C), t_R (major) = 20.83 min, t_R (minor) = 25.84 min. ^{1}H NMR (400 MHz, $CDCl_3$) δ (ppm) 7.98 – 7.84 (m, 2H), 7.65 – 7.53 (m, 1H), 7.47 (dd, *J* = 8.4, 7.1 Hz, 2H), 7.40 – 7.30 (m, 2H), 6.93 – 6.87 (m, 2H), 4.52 (dd, *J* = 7.7, 6.3 Hz, 1H), 3.80 (s, 3H), 3.69 (dd, *J* = 17.9, 7.7 Hz, 1H), 3.52 – 3.38 (m, 1H). ^{13}C NMR (100 MHz, $CDCl_3$) δ (ppm) 194.7, 159.5, 135.7, 133.9, 128.8, 128.7, 128.1, 127.1, 120.9, 114.6, 77.3, 77.0, 76.7, 55.3, 44.6, 31.1. HRMS (EI): m/z $[M + H]^+$ calcd for $C_{17}H_{16}NO_2$: 266.1175, found: 266.1175.

(*S*)-4-(4-(tert-butyl)phenyl)-4-oxo-2-phenylbutanenitrile (6bd)

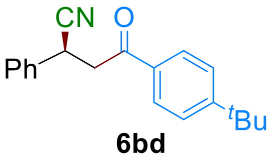

6bd

81% isolated yield, white solid, $[\alpha]_D^{25}$ = -13.22 (c = 0.5 in $CHCl_3$); 89% ee, determined by HPLC analysis (Chiralpak AS column, hexane/*i*-PrOH, 95:5 v/v, flow rate 0.5 mL/min, λ = 254 nm, 25 °C), t_R (major) = 38.37 min, t_R (minor) = 72.49 min. ^{1}H NMR (400 MHz, $CDCl_3$) δ (ppm) 7.86 (d, *J* = 8.6 Hz, 2H), 7.47 (d, *J* = 8.6 Hz, 2H), 7.43 (d, *J* = 8.8 Hz, 2H), 7.38 (t, *J* = 7.4 Hz, 2H), 7.33 (d, *J* = 7.1 Hz, 1H), 4.62 – 4.52 (m, 1H), 3.73 – 3.66 (m, 1H), 3.49 (dd, *J* = 17.9, 6.2 Hz, 1H), 1.33 (s, 9H). ^{13}C NMR (100 MHz, $CDCl_3$) δ (ppm) 194.2, 157.8, 135.3, 134.2, 133.1, 129.2, 128.3, 128.0, 127.5, 125.7, 120.7, 77.3, 77.0, 76.7, 44.4, 35.2, 31.8, 31.0. HRMS (EI): m/z $[M + Na]^+$ calcd for $C_{20}H_{21}NNaO$: 314.1515, found: 314.1512.

(*S*)-4-(4-fluorophenyl)-4-oxo-2-phenylbutanenitrile (6be)

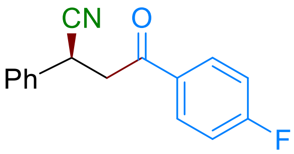

6be

77% isolated yield, white solid, $[\alpha]_D^{25}$ = -17.48 (c = 0.5 in $CHCl_3$); 91% ee, determined by HPLC analysis (Chiralpak OD column, hexane/*i*-PrOH, 90:10 v/v, flow rate 1.0 mL/min, λ = 254 nm, 25 °C), t_R (major) = 26.70 min, t_R (minor) = 34.33 min. ^1H NMR (400 MHz, $CDCl_3$) δ (ppm)) 7.96 (dd, *J* = 8.8, 5.4 Hz, 2H), 7.44 – 7.33 (m, 5H), 7.14 (t, *J* = 8.5 Hz, 2H), 4.55 (dd, *J* = 7.9, 6.0 Hz, 1H), 3.70 (dd, *J* = 17.9, 8.1 Hz, 1H), 3.47 (dd, *J* = 17.9, 5.9 Hz, 1H). ^{13}C NMR (100 MHz, $CDCl_3$) δ (ppm) 193.0, 166.2 (d, *J* = 254.7 Hz), 135.1, 132.1 (d, *J* = 3.0 Hz), 130.8 (d, *J* = 9.5 Hz), 129.3, 128.4, 127.4, 120.5, 116.0 (d, *J* = 2.2 Hz), 77.3, 77.0, 76.7, 44.4, 31.9. ^{19}F NMR (376 MHz, CDCl3) δ (ppm) -103.4. HRMS (EI): m/z $[M + Na]^+$ calcd for $C_{16}H_{12}FNNaO$: 276.0795, found: 276.0793.

(*S*)-4-(4-chlorophenyl)-4-oxo-2-phenylbutanenitrile (6bf)

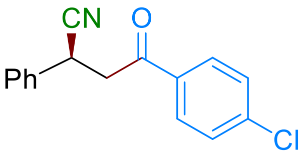

6bf

86% isolated yield, white solid, $[\alpha]_D^{25}$ = -0.6 (c = 0.5 in $CHCl_3$); 91% ee, determined by HPLC analysis (Chiralpak OD column, hexane/*i*-PrOH, 90:10 v/v, flow rate 1.0 mL/min, λ = 254 nm, 25 °C), t_R (major) = 26.45 min, t_R (minor) = 35.45 min. ^1H NMR (400 MHz, $CDCl_3$) δ (ppm) 7.86 (d, *J* = 8.7 Hz, 2H), 7.52 – 7.32 (m, 7H), 4.54 (dd, *J* = 8.1, 5.8 Hz, 1H), 3.69 (dd, *J* = 17.9, 8.1 Hz, 1H), 3.47 (dd, *J* = 18.0, 5.9 Hz, 1H). ^{13}C NMR (100 MHz, $CDCl_3$) δ (ppm) 193.5, 140.4, 135.0, 133.9, 129.4, 129.3, 129.1, 128.4, 127.4, 120.4, 77.3, 77.0, 76.7, 44.4, 31.9. HRMS (EI): m/z $[M + Na]^+$ calcd for $C_{16}H_{12}ClNNaO$: 292.0500, found: 292.0502.

(*S*)-4-(4-bromophenyl)-4-oxo-2-phenylbutanenitrile (6bg)

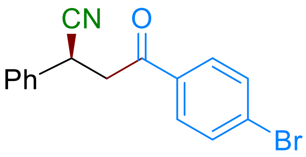

6bg

70% isolated yield, white solid, $[\alpha]_D^{25}$ = -16.17 (c = 0.5 in $CHCl_3$); 86% ee, determined by HPLC analysis (Chiralpak OD column, hexane/*i*-PrOH, 90:10 v/v, flow rate 1.0 mL/min, λ = 254 nm, 25 °C), t_R (major) = 29.52 min, t_R (minor) = 38.76 min. ^1H NMR (400 MHz, $CDCl_3$) δ (ppm) 7.78 (d, *J* = 8.3 Hz, 2H), 7.61 (d, *J* = 8.3 Hz, 2H), 7.43 – 7.33 (m, 5H), 4.62 – 4.46 (m, 1H), 3.69 (dd, *J* = 17.9, 8.1 Hz, 1H), 3.46 (dd, *J* = 17.9, 5.8 Hz, 1H). ^{13}C NMR (100 MHz, $CDCl_3$) δ (ppm) 193.7, 135.0, 134.3, 132.2, 129.5, 129.3, 129.2, 128.5, 127.4, 120.4, 77.3, 77.0, 76.7, 44.5, 31.9. HRMS (EI): m/z $[M + Na]^+$ calcd for $C_{16}H_{12}BrNNaO$: 335.9994, found: 335.9999.

(*S*)-4-(2-fluorophenyl)-4-oxo-2-phenylbutanenitrile (6bh)

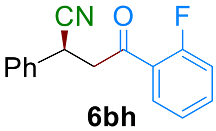

6bh

67% isolated yield, white solid, $[\alpha]_D^{25}$ = -6.96 (c = 0.5 in $CHCl_3$); 90% ee, determined by HPLC analysis (Chiralpak AZ column, hexane/*i*-PrOH, 90:10 v/v, flow rate 1.0 mL/min, λ = 220 nm, 25 °C), t_R (major) = 23.41 min, t_R (minor) = 21.04 min. ^1H NMR (400 MHz, $CDCl_3$) δ (ppm) 7.96 – 7.92 (m, 1H), 7.63 – 7.50 (m, 1H), 7.48 – 7.33 (m, 5H), 7.29 – 7.22 (m, 1H), 7.14 (dd, *J* = 11.1, 8.7 Hz, 1H), 4.55

(dd, J = 8.1, 5.9 Hz, 1H), 3.74 (ddd, J = 18.7, 8.3, 3.2 Hz, 1H), 3.52 (ddd, J = 18.7, 5.7, 3.2 Hz, 1H). ^{13}C NMR (100 MHz, $CDCl_3$) 192.8 (d, J = 4.0 Hz), 162.3 (d, J = 253.4 Hz), 135.5 (d, J = 9.2 Hz), 135.1, 130.8 (d, J = 2.3 Hz), 129.2, 128.3, 127.5, 124.7, (d, J = 3.3 Hz), 124.1 (d, J = 12.4 Hz), 120.5, 116.7 (d, J = 24.6 Hz), 77.3, 77.0, 76.7, 49.1, 49.0, 31.9, 31.8. ^{19}F NMR (376 MHz, CDCl3) δ (ppm) -108.5. HRMS (EI): m/z $[M + Na]^+$ calcd for $C_{16}H_{12}FNNaO$: 276.0795, found: 276.0795.

(*S*)-4-oxo-2-phenyl-4-(m-tolyl)butanenitrile (6bi)

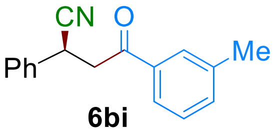

6bi

84% isolated yield, white solid, $[\alpha]_D^{25}$ = -26.41 (c = 0.5 in $CHCl_3$); 89% ee, determined by HPLC analysis (Chiralpak OD column, hexane/*i*-PrOH, 98:2 v/v, flow rate 1.0 mL/min, λ = 220 nm, 25 °C), t_R (major) = 24.40 min, t_R (minor) = 29.68 min. ^{1}H NMR (400 MHz, $CDCl_3$) δ (ppm) 7.76 – 7.66 (m, 2H), 7.41 (dd, J = 15.6, 7.1 Hz, 4H), 7.36 – 7.31 (m, 3H), 4.55 (dd, J = 7.9, 6.1 Hz, 1H), 3.70 (dd, J = 17.9, 8.0 Hz, 1H), 3.49 (dd, J = 18.0, 6.0 Hz, 1H), 2.38 (s, 3H). ^{13}C NMR (100 MHz, $CDCl_3$) δ (ppm) 194.8, 138.7, 135.7, 135.3, 134.7, 129.3, 128.7, 128.6, 128.4, 127.5, 125.3, 120.7, 77.4, 77.0, 76.7, 44.6, 31.9, 21.3. HRMS (EI): m/z $[M + Na]^+$ calcd for $C_{17}H_{15}NNaO$: 272.1046, found: 272.1049.

(*S*)-4-(3-chlorophenyl)-4-oxo-2-phenylbutanenitrile (6bj)

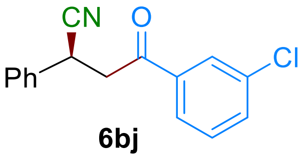

6bj

85% isolated yield, white solid, $[\alpha]_D^{25}$ = -28.83 (c = 0.5 in $CHCl_3$); 91% ee, determined by HPLC analysis (Chiralpak AZ column, hexane/*i*-PrOH, 85:15 v/v, flow rate 1.0 mL/min, λ = 254 nm, 25 °C), t_R (major) =22.87 min, t_R (minor) = 20.05min. ^{1}H NMR (400 MHz, $CDCl_3$) δ (ppm) 7.89 (s, 1H), 7.80 (d, J = 7.8 Hz, 1H), 7.57 (d, J = 9.0 Hz, 1H), 7.41 (d, J = 7.8 Hz, 4H), 7.38 – 7.32 (m, 1H), 4.55 (dd, J = 8.0, 6.0 Hz, 1H), 3.70 (dd, J = 18.0, 8.0 Hz, 1H), 3.48 (dd, J = 18.0, 5.9 Hz, 1H). ^{13}C NMR (100 MHz, $CDCl_3$) δ (ppm) 193.5, 137.1, 135.2, 135.0, 133.8, 130.2, 129.3, 128.5, 128.2, 127.5, 126.2, 120.4, 77.4, 77.0, 76.8, 44.6, 31.9. HRMS (EI): m/z $[M + Na]^+$ calcd for $C_{16}H_{12}ClNNaO$: 292.0500, found: 292.0502.

(*S*)-4-(3-bromophenyl)-4-oxo-2-phenylbutanenitrile (6bk)

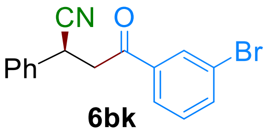

6bk

81% isolated yield, white solid, $[\alpha]_D^{25}$ = -15.73 (c = 0.5 in $CHCl_3$); 92% ee, determined by HPLC analysis (Chiralpak AZ column, hexane/*i*-PrOH, 90:10 v/v, flow rate 1.0 mL/min, λ = 254 nm, 25 °C), t_R (major) = 34.83 min, t_R (minor) = 28.94 min. ^{1}H NMR (400 MHz, $CDCl_3$) δ (ppm) 8.05 (s, 1H), 7.85 (d, J = 8.0 Hz, 1H), 7.72 (d, J = 9.7 Hz, 1H), 7.52 – 7.31 (m, 6H), 4.55 (dd, J = 7.9, 6.0 Hz, 1H), 3.70 (dd, J = 18.0, 8.0 Hz, 1H), 3.47 (dd, J = 18.0, 6.0 Hz, 1H). ^{13}C NMR (100 MHz, $CDCl_3$) δ (ppm) 193.4, 137.3, 136.7, 134.9, 131.1, 130.4, 129.3, 128.5, 127.4, 126.6, 123.2, 77.3, 77.0, 76.7, 44.6, 31.8. HRMS (EI): m/z $[M + Na]^+$ calcd for $C_{16}H_{12}BrNNaO$: 335.9994, found: 335.9994.

(*S*)-4-(furan-2-yl)-4-oxo-2-phenylbutanenitrile (6bl)

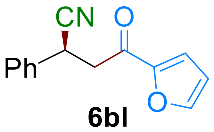

6bl

68% isolated yield, white solid, $[\alpha]_D^{25}$ = -19.20 (c = 0.5 in $CHCl_3$); 89% ee, determined by HPLC analysis (Chiralpak OD column, hexane/*i*-PrOH, 85:15 v/v, flow rate 1.0 mL/min, λ = 254 nm, 25 °C), t_R (major) = 18.68 min, t_R (minor) = 16.64 min. ^{1}H NMR (400 MHz, $CDCl_3$) δ (ppm) 7.59 (s, 1H), 7.40 – 7.31 (m, 5H), 7.24 (d, *J* = 3.6 Hz, 1H), 6.56 (dd, *J* = 3.7, 1.7 Hz, 1H), 4.54 (t, *J* = 7.2 Hz, 1H), 3.60 (dd, *J* = 17.6, 7.9 Hz, 1H), 3.38 (dd, *J* = 17.6, 6.5 Hz, 1H). ^{13}C NMR (100 MHz, $CDCl_3$) δ (ppm) 183.7, 151.8, 147.0, 135.0, 129.3, 128.4, 127.5, 120.3, 118.0, 112.7, 77.3, 77.0, 76.7, 43.9, 31.5. HRMS (EI): m/z $[M + Na]^+$ calcd for $C_{14}H_{11}NNaO_2$: 248.0682, found: 248.0680.

(*S*)-2-(naphthalen-2-yl)-4-oxopentanenitrile (6am)

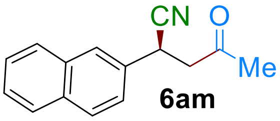

6am

67% isolated yield, colorless oil, $[\alpha]_D^{25}$ = -32.85 (c = 0.5 in $CHCl_3$); 86% ee, determined by HPLC analysis (Chiralpak AZ column, hexane/*i*-PrOH, 90:10 v/v, flow rate 1.0 mL/min, λ = 254 nm, 25 °C), t_R (major) = 25.14 min, t_R (minor) = 28.11 min. ^{1}H NMR (400 MHz, $CDCl_3$) δ (ppm) 7.85 (t, *J* = 9.8 Hz, 4H), 7.56 – 7.39 (m, 3H), 4.52 (t, *J* = 6.9 Hz, 1H), 3.26 (dd, *J* = 18.1, 7.8 Hz, 1H), 3.06 (dd, *J* = 18.0, 6.1 Hz, 1H), 2.19 (s, 3H). ^{13}C NMR (100 MHz, $CDCl_3$) δ (ppm) 203.0, 133.2, 132.8, 132.2, 129.3, 127.9, 127.7, 126.8, 126.7, 126.6, 124.6, 120.4, 77.3, 77.0, 76.7, 48.7, 31.7, 30.0. HRMS (EI): m/z $[M + Na]^+$ calcd for $C_{15}H_{13}NNaO$: 246.0889, found: 246.0889.

(*S*)-2-(naphthalen-2-yl)-4-oxohexanenitrile (6an)

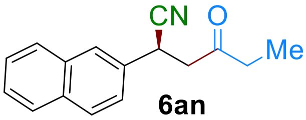

6an

69% isolated yield, colorless oil, $[\alpha]_D^{25}$ = -36.33 (c = 0.5 in $CHCl_3$); 87% ee, determined by HPLC analysis (Chiralpak AD column, hexane/*i*-PrOH, 99:1 v/v, flow rate 1.0 mL/min, λ = 220 nm, 25 °C), t_R (major) = 37.40 min, t_R (minor) = 32.27 min. ^{1}H NMR (400 MHz, $CDCl_3$) δ (ppm) 7.91 – 7.73 (m, 4H), 7.51 (q, *J* = 4.4 Hz, 2H), 7.41 (d, *J* = 8.5 Hz, 1H), 4.53 (t, *J* = 7.0 Hz, 1H), 3.21 (dd, *J* = 17.7, 7.8 Hz, 1H), 3.01 (dd, *J* = 17.7, 6.2 Hz, 1H), 2.56 – 2.24 (m, 2H), 1.05 (t, *J* = 7.3 Hz, 3H). ^{13}C NMR (100 MHz, $CDCl_3$) δ (ppm) 205.9, 133.2, 132.8, 132.3, 129.2, 129.2, 127.9, 127.8, 127.7, 126.8, 126.8, 126.6, 126.6, 126.5, 124.6, 120.4, 77.3, 77.0, 76.7, 47.4, 36.1, 31.7, 7.4. HRMS (EI): m/z $[M + Na]^+$ calcd for $C_{16}H_{15}NNaO$: 260.1046, found: 260.1045.

(*S*)-2-(naphthalen-2-yl)-4-oxoheptanenitrile (6ao)

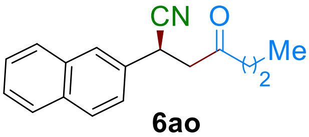

6ao

61% isolated yield, colorless oil, $[\alpha]_D^{25}$ = -38.92 (c = 0.5 in $CHCl_3$); 86% ee, determined by HPLC analysis (Chiralpak AZ column, hexane/*i*-PrOH, 90:10 v/v, flow rate 1.0 mL/min, λ = 254 nm, 25 °C), t_R (major) = 17.92 min, t_R (minor) = 19.98 min. ^{1}H NMR (400 MHz, $CDCl_3$) δ (ppm) 7.89 – 7.77 (m, 4H), 7.53 – 7.36 (m, 3H), 4.59 – 4.44 (m, 1H), 3.19 (dd, *J* = 17.8, 7.8 Hz,

1H), 2.99 (dd, J = 17.8, 6.2 Hz, 1H), 2.50 – 2.25 (m, 2H), 1.59 (h, J = 7.2 Hz, 2H), 0.87 (t, J = 7.4 Hz, 3H). ^{13}C NMR (100 MHz, $CDCl_3$) δ (ppm) 205.4, 133.1, 132.7, 132.3, 129.1, 127.8, 127.6, 126.7, 126.5, 126.5, 124.6, 120.4, 77.3, 77.0, 76.7, 47.7, 44.7, 31.6, 16.9, 13.5. HRMS (EI): m/z $[M + Na]^+$ calcd for $C_{17}H_{17}NNaO$: 274.1202, found: 274.1200.

(*S*)-2-(naphthalen-2-yl)-4-oxooctanenitrile (6ap)

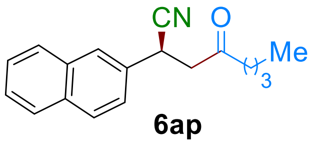

6ap

70% isolated yield, colorless oil, $[\alpha]_D^{25}$ = -40.89 (c = 0.5 in $CHCl_3$); 90% ee, determined by HPLC analysis (Chiralpak OD column, hexane/*i*-PrOH, 90:10 v/v, flow rate 1.0 mL/min, λ = 220 nm, 25 °C), t_R (major) = 21.05 min, t_R (minor) = 23.67 min. ^{1}H NMR (400 MHz, $CDCl_3$) δ (ppm) 7.88 – 7.77 (m, 5H), 7.51 (q, J = 5.1, 4.4 Hz, 2H), 7.40 (d, J = 8.5 Hz, 1H), 4.53 (t, J = 6.9 Hz, 1H), 3.20 (dd, J = 17.8, 7.8 Hz, 1H), 3.00 (dd, J = 17.8, 6.2 Hz, 1H), 2.49 – 2.27 (m, 2H), 1.57 – 1.50 (m, 2H), 1.31 – 1.21 (m, 2H), 0.85 (t, J = 7.3 Hz, 3H). ^{13}C NMR (100 MHz, $CDCl_3$) δ (ppm) 205.6, 133.2, 132.8, 132.3, 129.2, 127.8, 127.6, 126.7, 126.6, 126.6, 124.6, 120.4, 77.3, 77.0, 76.7, 47.7, 42.6, 31.7, 25.5, 22.1, 13.7. HRMS (EI): m/z $[M + Na]^+$ calcd for $C_{18}H_{19}NNaO$: 288.1359, found: 288.1360.

(S)-2-phenylheptanedinitrile (7aa)

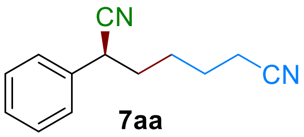

7aa

51% isolated yield, yellow oil, $[\alpha]_D^{25}$ = -16.64 (c = 0.67 in $CHCl_3$); 88% ee, determined by HPLC analysis (Chiralpak AD column, hexane/*i*-PrOH, 90:10 v/v, flow rate 1.0 mL/min, λ = 210 nm, 25 °C), t_R (major) = 14.49 min, t_R (minor) = 15.14 min; ^{1}H NMR (400 MHz, $CDCl_3$) δ (ppm) 7.42 – 7.29 (m, 5H), 3.81 (t, J = 7.2 Hz, 1H), 2.35 (t, J = 6.8 Hz, 2H), 2.02 – 1.87 (m, 2H), 1.74 – 1.61 (m, 4H). ^{13}C NMR (100 MHz, $CDCl_3$) δ (ppm) 135.1, 129.1, 128.2, 127.0, 120.3, 119.1, 37.0, 34.9, 26.0, 24.7, 16.9. HRMS (EI): m/z $[M + Na]^+$ calcd for $C_{13}H_{14}N_2Na$: 221.1049, found: 221.1046.

(S)-2-(p-tolyl)heptanedinitrile (7ba)

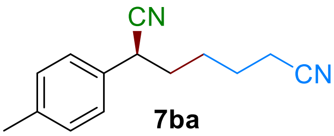

7ba

63% isolated yield, yellow oil, $[\alpha]_D^{25}$ = -12.74 (c = 0.81 in $CHCl_3$); 86% ee, determined by HPLC analysis (Chiralpak AZ column, hexane/*i*-PrOH, 85:15 v/v, flow rate 0.5 mL/min, λ = 220 nm, 25 °C), t_R (major) = 39.31 min, t_R (minor) = 37.37 min; ^{1}H NMR (400 MHz, $CDCl_3$) δ (ppm) 7.20 (s, 4H), 3.77 (t, J = 7.3 Hz, 1H), 2.34 (d, J = 8.9 Hz, 5H), 2.02 – 1.82 (m, 2H), 1.68 (dt, J = 15.8, 8.5 Hz, 4H). ^{13}C NMR (100 MHz, $CDCl_3$) δ (ppm) 138.0, 132.1, 129.7, 126.9, 120.5, 119.1, 36.6, 34.9, 26.0, 24.8, 20.9, 16.9. HRMS (EI): m/z $[M + Na]^+$ calcd for $C_{14}H_{16}N_2Na$: 235.1205, found: 235.1204.

(S)-2-(4-(tert-butyl)phenyl)heptanedinitrile (7ca)

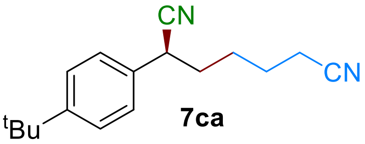

7ca

57% isolated yield, colorless oil, $[\alpha]_D^{25}$ = -8.97 (c = 0.85 in $CHCl_3$); 83% ee, determined by HPLC analysis (Chiralpak OD column, hexane/*i*-PrOH, 85:15 v/v, flow rate 1.0 mL/min, λ = 210 nm, 25 °C), t_R (major) = 14.53 min, t_R (minor) = 17.19 min; ^1H NMR (400 MHz, $CDCl_3$) δ (ppm) 7.40 (d, *J* = 8.3 Hz, 2H), 7.25 (d, *J* = 8.4 Hz, 2H), 3.78 (t, *J* = 7.7 Hz, 1H), 2.36 (t, *J* = 6.7 Hz, 2H), 2.02 – 1.83 (m, 2H), 1.74 – 1.59 (m, 4H), 1.32 (s, 9H). ^{13}C NMR (100 MHz, $CDCl_3$) δ (ppm) 151.2, 132.1, 126.7, 126.0, 120.5, 119.2, 36.5, 34.8, 34.4, 31.1, 26.1, 24.7, 16.9. HRMS (EI): m/z $[M + Na]^+$ calcd for $C_{17}H_{22}N_2Na$: 277.1675, found: 277.1672.

(S)-2-(4-bromophenyl)heptanedinitrile (7da)

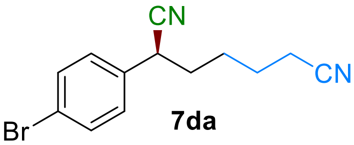

7da

72% isolated yield, yellow oil, $[\alpha]_D^{25}$ = 0.19 (c = 1.21 in $CHCl_3$); 89% ee, determined by HPLC analysis (Chiralpak AZ column, hexane/*i*-PrOH, 85:15 v/v, flow rate 1.0 mL/min, λ = 210 nm, 25 °C), t_R (major) = 23.51 min, t_R (minor) = 21.51 min; ^1H NMR (400 MHz, $CDCl_3$) δ (ppm) 7.53 (d, *J* = 8.2 Hz, 2H), 7.21 (d, *J* = 8.1 Hz, 2H), 3.79 (t, *J* = 7.3 Hz, 1H), 2.36 (t, *J* = 6.8 Hz, 2H), 2.01 – 1.84 (m, 2H), 1.74 – 1.58 (m, 4H). ^{13}C NMR (100 MHz, $CDCl_3$) δ (ppm) 134.2, 132.2, 128.7, 122.2, 119.8, 119.0, 36.5, 34.7, 25.9, 24.6, 16.9. HRMS (EI): m/z $[M + Na]^+$ calcd for $C_{13}H_{13}BrN_2Na$: 299.0154, found: 299.0156.

(S)-2-(4-chlorophenyl)heptanedinitrile (7ea)

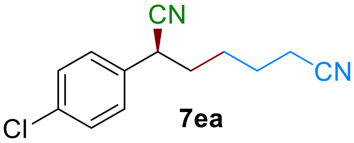

7ea

63% isolated yield, colorless oil, $[\alpha]_D^{25}$ = -35.65 (c = 0.76 in $CHCl_3$); 90% ee, determined by HPLC analysis (Chiralpak AZ column, hexane/*i*-PrOH, 80:20 v/v, flow rate 1.0 mL/min, λ = 210 nm, 25 °C), t_R (major) = 15.61 min, t_R (minor) = 14.30 min; ^1H NMR (400 MHz, $CDCl_3$) δ (ppm) 7.37 (d, *J* = 8.3 Hz, 2H), 7.27 (d, *J* = 8.3 Hz, 2H), 3.80 (t, *J* = 7.3 Hz, 1H), 2.37 (t, *J* = 6.8 Hz, 2H), 1.98 – 1.85 (m, 2H), 1.75 – 1.59 (m, 4H). ^{13}C NMR (100 MHz, $CDCl_3$) δ (ppm) 134.2, 133.6, 129.3, 128.4, 119.9, 119.1, 36.5, 34.8, 26.0, 24.7, 16.9. HRMS (EI): m/z $[M + Na]^+$ calcd for $C_{13}H_{13}ClN_2Na$: 255.0659, found: 255.0658.

(S)-2-(4-(trifluoromethyl)phenyl)heptanedinitrile (7fa)

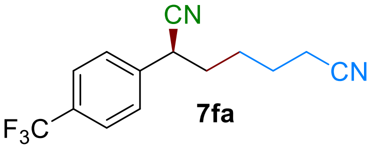

7fa

73% isolated yield, colorless oil, $[\alpha]_D^{25}$ = -1.48 (c = 1.23 in $CHCl_3$); 96% ee, determined by HPLC analysis (Chiralpak AS column, hexane/*i*-PrOH, 90:10 v/v, flow rate 0.5 mL/min, λ = 210 nm, 25 °C), t_R (major) = 66.12 min, t_R (minor) = 70.53 min; ^1H NMR (400 MHz, $CDCl_3$) δ (ppm) 7.67 (d, *J* = 7.8 Hz, 2H), 7.48 (d, *J* = 8.0 Hz, 2H), 3.90 (t, *J* = 7.4 Hz, 1H), 2.38 (t, *J* = 6.8 Hz, 2H), 2.05 – 1.90 (m, 2H), 1.77 – 1.62 (m, 4H).. ^{13}C NMR (101 MHz, $CDCl_3$) δ (ppm) 139.1 ,

130.6 (q, J = 33.3 Hz), 127.6, 126.2 (q, J = 4.0 Hz), 123.6 (q, J = 273.7 Hz), 119.5, 119.0, 36.9, 34.8, 26.0, 24.7, 16.9. ^{19}F NMR (376 MHz, $CDCl_3$) δ -62.74. HRMS (EI): m/z [M + Na]$^+$ calcd for $C_{14}H_{13}F_3N_2Na$: 289.0923, found: 289.0925.

(S)-methyl-4-(1,5-dicyanopentyl)benzoate (7ga)

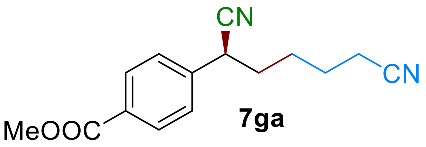

62% isolated yield, colorless oil, $[\alpha]_D^{25}$ = -2.68 (c = 1.08 in $CHCl_3$); 89% ee, determined by HPLC analysis (Chiralpak AD column, hexane/*i*-PrOH, 80:20 v/v, flow rate 1.0 mL/min, λ = 210 nm, 25 °C), t_R (major) = 13.59 min, t_R (minor) = 15.15 min; ^{1}H NMR (400 MHz, $CDCl_3$) δ (ppm) 8.07 (d, J = 8.3 Hz, 2H), 7.42 (d, J = 8.3 Hz, 2H), 3.93 (s, 3H), 3.89 (t, J = 7.3 Hz, 1H), 2.37 (t, J = 6.7 Hz, 2H), 1.97 (dt, J = 15.1, 7.0 Hz, 2H), 1.73 – 1.64 (m, 4H).^{13}C NMR (100 MHz, $CDCl_3$) δ (ppm) 166.2, 140.0, 130.4, 130.2, 127.2, 119.7, 119.0, 52.2, 37.1, 34.7, 26.0, 24.7, 16.9. HRMS (EI): m/z [M + Na]$^+$ calcd for $C_{15}H_{16}N_2O_2Na$: 279.1103, found: 279.1106.

(S)-2-(4-cyanophenyl)heptanedinitrile (7ha)

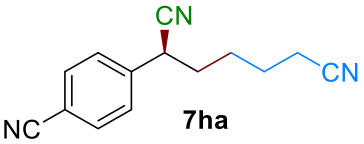

59% isolated yield, colorless oil, $[\alpha]_D^{25}$ = -9.84 (c = 0.66 in $CHCl_3$); 90% ee, determined by HPLC analysis (Chiralpak AD column, hexane/*i*-PrOH, 80:20 v/v, flow rate 1.0 mL/min, λ = 210 nm, 25 °C), t_R (major) = 19.60 min, t_R (minor) = 16.81 min; ^{1}H NMR (400 MHz, $CDCl_3$) δ (ppm) 7.71 (d, J = 6.6 Hz, 2H), 7.48 (d, J = 6.7 Hz, 2H), 3.92 (t, J = 6.4 Hz, 1H), 2.39 (t, J = 6.8 Hz, 2H), 2.02 – 1.91 (m, 2H), 1.77 – 1.63 (m, 4H). ^{13}C NMR (100 MHz, $CDCl_3$) δ (ppm) 140.3, 132.9, 128.0, 119.1, 119.0, 117.9, 112.4, 37.1, 34.6, 26.0, 24.6, 16.9. HRMS (EI): m/z [M + Na]$^+$ calcd for $C_{14}H_{13}N_3Na$: 246.1001, found: 246.0997.

(S)-2-([1,1'-biphenyl]-4-yl)heptanedinitrile (7ia)

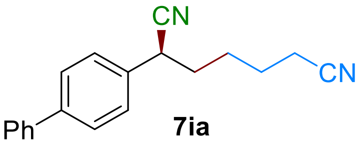

75% isolated yield, white solid, $[\alpha]_D^{25}$ = -2.19 (c = 0.56 in $CHCl_3$); 90% ee, determined by HPLC analysis (Chiralpak AD column, hexane/*i*-PrOH, 80:20 v/v, flow rate 1.0 mL/min, λ = 254 nm, 25 °C), t_R (major) = 11.20 min, t_R (minor) = 13.44 min; ^{1}H NMR (400 MHz, $CDCl_3$) δ (ppm) 7.64 – 7.56 (m, 4H), 7.45 (td, J = 7.2, 6.2, 1.3 Hz, 2H), 7.42 – 7.34 (m, 3H), 3.86 (t, J = 7.3 Hz, 1H), 2.37 (t, J = 6.7 Hz, 2H), 2.07 – 1.89 (m, 2H), 1.77 – 1.63 (m, 4H). ^{13}C NMR (100 MHz, $CDCl_3$) δ (ppm) 141.2, 140.0, 134.1, 128.8, 127.8, 127.6, 127.5, 127.0, 120.3, 119.1, 36.8, 34.9, 26.1, 24.8, 17.0. HRMS (EI): m/z [M + Na]$^+$ calcd for $C_{19}H_{18}N_2Na$: 297.1362, found: 297.1358.

(S)-2-(4'-chloro-[1,1'-biphenyl]-4-yl)heptanedinitrile (7ja)

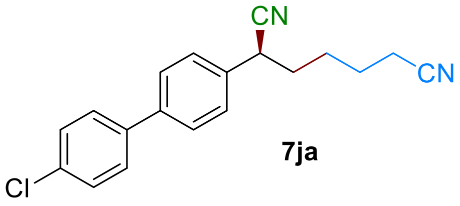

7ja

70% isolated yield, white solid, $[\alpha]_D^{25}$ = -1.72 (c = 1.22 in $CHCl_3$); 91% ee, determined by HPLC analysis (Chiralpak AD column, hexane/*i*-PrOH, 80:20 v/v, flow rate 1.0 mL/min, λ = 254 nm, 25 °C), t_R (major) = 12.46 min, t_R (minor) = 14.00 min; ^1H NMR (400 MHz, $CDCl_3$) δ (ppm) 7.57 (d, *J* = 8.4 Hz, 2H), 7.50 (d, *J* = 8.6 Hz, 2H), 7.40 (dd, *J* = 8.4, 6.3 Hz, 4H), 3.85 (t, *J* = 7.3 Hz, 1H), 2.37 (t, *J* = 6.7 Hz, 2H), 2.05 – 1.92 (m, 2H), 1.75 – 1.61 (m, 4H). ^{13}C NMR (100 MHz, $CDCl_3$) δ (ppm) 139.9, 138.4, 134.5, 133.7, 128.9, 128.2, 127.6, 127.6, 120.2, 119.1, 36.7, 34.8, 26.0, 24.8, 16.9. HRMS (EI): m/z $[M + Na]^+$ calcd for $C_{19}H_{17}ClN_2Na$: 331.0972, found: 331.0972.

(S)-2-(3-methoxyphenyl)heptanedinitrile (7ka)

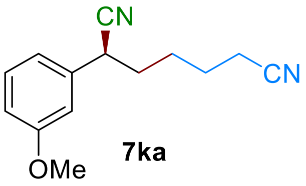

7ka

51% isolated yield, colorless oil, $[\alpha]_D^{25}$ = -14.92 (c = 0.65 in $CHCl_3$); 88% ee, determined by HPLC analysis (Chiralpak AD column, hexane/*i*-PrOH, 95:5 v/v, flow rate 1.0 mL/min, λ = 214 nm, 25 °C), t_R (major) = 37.73 min, t_R (minor) = 39.97 min; ^1H NMR (400 MHz, $CDCl_3$) δ (ppm) 7.34 – 7.27 (m, 1H), 6.93 – 6.83 (m, 3H), 3.82 (s, 3H), 3.78 (t, *J* = 7.6 Hz, 1H), 2.36 (t, *J* = 6.8 Hz, 2H), 2.02 – 1.89 (m, 2H), 1.75 – 1.58 (m, 4H). ^{13}C NMR (100 MHz, $CDCl_3$) δ (ppm) 160.0, 136.6, 130.2, 120.3, 119.3, 119.1, 113.4, 113.0, 55.2, 37.0, 34.8, 26.0, 24.7, 16.9. HRMS (EI): m/z $[M + Na]^+$ calcd for $C_{14}H_{16}N_2NaO$: 251.1154, found: 251.1151.

(S)-2-(3-fluorophenyl)heptanedinitrile (7la)

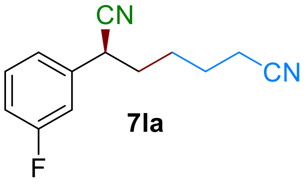

7la

68% isolated yield, colorless oil, $[\alpha]_D^{25}$ = -14.43 (c = 0.94 in $CHCl_3$); 92% ee, determined by HPLC analysis (Chiralpak AZ column, hexane/*i*-PrOH, 85:15 v/v, flow rate 0.5 mL/min, λ = 210 nm, 25 °C), t_R (major) = 42.68 min, t_R (minor) = 40.82 min; ^1H NMR (400 MHz, $CDCl_3$) δ (ppm) 7.38 (q, *J* = 7.4 Hz, 1H), 7.13 (d, *J* = 7.7 Hz, 1H), 7.05 (t, *J* = 8.2 Hz, 2H), 3.83 (t, *J* = 7.3 Hz, 1H), 2.37 (t, *J* = 6.8 Hz, 2H), 2.02 – 1.88 (m, 2H), 1.75 – 1.58 (m, 4H). ^{13}C NMR (100 MHz, $CDCl_3$) δ (ppm) 162.89 (d, *J* = 246.0 Hz), 137.5 (d, *J* = 7.0 Hz), 130.8 (d, *J* = 8.0 Hz), 122.8 (d, *J* = 3.0 Hz), 119.7, 119.0, 115.3 (d, *J* = 21.0 Hz), 114.3(d, *J* = 23.0 Hz), 36.7, 34.6, 25.9, 24.7, 16.8. ^{19}F NMR (376 MHz, CDC13) δ -111.34 (s). HRMS (EI): m/z $[M + Na]^+$ calcd for $C_{13}H_{13}FN_2Na$: 239.0954, found: 239.0957.

(S)-2-(2-methoxyphenyl)heptanedinitrile (7ma)

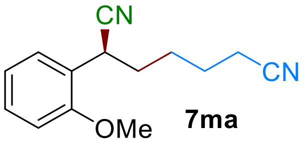
7ma

57% isolated yield, colorless oil, $[\alpha]_D^{25}$ = -29.12 (c = 0.80 in $CHCl_3$); 82% ee, determined by HPLC analysis (Chiralpak OD column, hexane/*i*-PrOH, 80:20 v/v, flow rate 0.25 mL/min, λ = 220 nm, 25 °C), t_R (major) = 76.59 min, t_R (minor) = 71.52 min; ^{1}H NMR (400 MHz, $CDCl_3$) δ (ppm) 7.39 (d, *J* = 7.5 Hz, 1H), 7.31 (t, *J* = 7.9 Hz, 1H), 6.99 (t, *J* = 7.5 Hz, 1H), 6.90 (d, *J* = 8.2 Hz, 1H), 4.20 (t, *J* = 7.2 Hz, 1H), 3.86 (s, 3H), 2.36 (t, *J* = 6.8 Hz, 2H), 1.92 – 1.86 (m, 2H), 1.75 – 1.57 (m, 4H). ^{13}C NMR (100 MHz, $CDCl_3$) δ (ppm) 155.8, 129.4, 128.1, 123.4, 120.8, 120.7, 119.2, 110.7, 55.4, 32.7, 31.0, 26.1, 24.7, 16.8. HRMS (EI): m/z $[M + Na]^+$ calcd for $C_{14}H_{16}N_2NaO$: 251.1154, found: 251.1154.

(S)-2-(2-chlorophenyl)heptanedinitrile (7na)

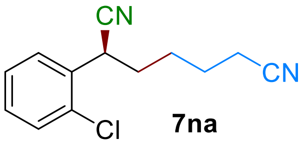
7na

78% isolated yield, colorless oil, $[\alpha]_D^{25}$ = -43.27 (c = 1.20 in $CHCl_3$); 97% ee, determined by HPLC analysis (Chiralpak AZ column, hexane/*i*-PrOH, 90:10 v/v, flow rate 1.0 mL/min, λ = 210 nm, 25 °C), t_R (major) = 25.23 min, t_R (minor) = 23.48 min; ^{1}H NMR (400 MHz, $CDCl_3$) δ (ppm) 7.56 (d, *J* = 7.5 Hz, 1H), 7.41 (d, *J* = 7.6 Hz, 1H), 7.37 – 7.26 (m, 2H), 4.31 (t, *J* = 7.1 Hz, 1H), 2.38 (t, *J* = 6.7 Hz, 2H), 1.98 – 1.88 (m, 2H), 1.77 – 1.65 (m, 4H). ^{13}C NMR (100 MHz, $CDCl_3$) δ (ppm) 133.0, 132.4, 130.0, 129.6, 128.7, 127.6, 119.7, 119.1, 34.2, 33.1, 26.0, 24.6, 16.8. HRMS (EI): m/z $[M + Na]^+$ calcd for $C_{13}H_{13}ClN_2Na$: 255.0659, found: 255.0655.

(S)-2-(naphthalen-2-yl)heptanedinitrile (7oa)

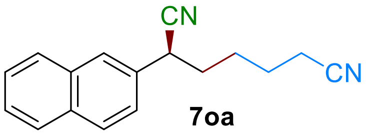
7oa

56% isolated yield, white solid, $[\alpha]_D^{25}$ = -9.83 (c = 0.79 in $CHCl_3$); 90% ee, determined by HPLC analysis (Chiralpak OD column, hexane/*i*-PrOH, 85:15 v/v, flow rate 1.0 mL/min, λ = 254 nm, 25 °C), t_R (major) = 44.85 min, t_R (minor) = 51.75 min; ^{1}H NMR (400 MHz, $CDCl_3$) δ (ppm) 7.90 – 7.79 (m, 4H), 7.56 – 7.48 (m, 2H), 7.39 (dd, *J* = 8.5, 1.8 Hz, 1H), 3.97 (t, *J* = 7.2 Hz, 1H), 2.33 (t, *J* = 6.6 Hz, 2H), 2.07 – 1.97 (m, 2H), 1.73 – 1.57 (m, 4H). ^{13}C NMR (100 MHz, $CDCl_3$) δ (ppm) 133.1, 132.7, 132.4, 129.1, 127.7, 127.6, 126.7, 126.5, 126.2, 124.4, 120.3, 119.1, 37.2, 34.8, 26.1, 24.8, 16.9. HRMS (EI): m/z $[M + Na]^+$ calcd for $C_{17}H_{16}N_2Na$: 271.1205, found: 271.1198.

(S)-2-(benzo[d][1,3]dioxol-5-yl)heptanedinitrile (7pa)

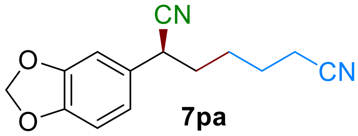
7pa

58% isolated yield, colorless oil, $[\alpha]_D^{25}$ = -6.94 (c = 0.84 in $CHCl_3$); 84% ee, determined by HPLC analysis (Chiralpak AD column, hexane/*i*-PrOH, 95:5 v/v, flow rate 1.0 mL/min, λ = 210 nm, 25 °C), t_R (major) = 61.70 min, t_R (minor) = 65.44 min; ^{1}H NMR (400 MHz, $CDCl_3$) δ (ppm) 6.83 – 6.75 (m, 3H), 5.99 (s, 2H), 3.71 (t, *J* = 7.3 Hz, 1H), 2.36 (t, *J* = 6.8 Hz, 2H), 1.96 – 1.84 (m, 2H), 1.74 – 1.55 (m, 4H). ^{13}C NMR (100 MHz, $CDCl_3$) δ (ppm) 148.3, 147.5, 128.7, 120.6, 120.4, 119.1,

108.6, 107.4, 101.4, 36.7, 35.0, 26.0, 24.8, 16.9. HRMS (EI): m/z [M + Na]$^+$ calcd for $C_{14}H_{14}N_2NaO_2$: 265.0947, found: 265.0942.

(R)-2-(thiophen-2-yl)heptanedinitrile (7qa)

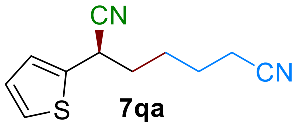

54% isolated yield, colorless oil, $[\alpha]_D^{25}$ = -13.06 (c = 0.62 in $CHCl_3$); 94% ee, determined by HPLC analysis (Chiralpak AD column, hexane/*i*-PrOH, 80:20 v/v, flow rate 1.0 mL/min, λ = 210 nm, 25 °C), t_R (major) = 8.55 min, t_R (minor) = 8.91 min; ^{1}H NMR (400 MHz, $CDCl_3$) δ (ppm) 7.29 (dd, *J* = 5.1, 1.3 Hz, 1H), 7.07 (dt, *J* = 3.5, 1.1 Hz, 1H), 6.99 (dd, *J* = 5.2, 3.5 Hz, 1H), 4.10 (t, *J* = 7.1 Hz, 1H), 2.37 (t, *J* = 6.8 Hz, 2H), 2.08 – 1.98 (m, 2H), 1.76 – 1.62 (m, 4H). ^{13}C NMR (100 MHz, $CDCl_3$) δ (ppm) 137.1, 127.1, 126.2, 125.6, 119.4, 119.1, 34.9, 32.2, 25.9, 24.7, 16.9. HRMS (EI): m/z [M + Na]$^+$ calcd for $C_{11}H_{12}N_2NaS$: 227.0613, found: 227.0609.

(S)-2-(1-tosyl-1H-indol-3-yl)heptanedinitrile (7ra)

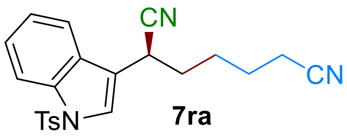

34% isolated yield, yellow oil, $[\alpha]_D^{25}$ = -15.06 (c = 0.81 in $CHCl_3$); 85% ee, determined by HPLC analysis (Chiralpak AD column, hexane/*i*-PrOH, 80:20 v/v, flow rate 1.0 mL/min, λ = 210 nm, 25 °C), t_R (major) = 25.13 min, t_R (minor) = 21.06 min; ^{1}H NMR (400 MHz, $CDCl_3$) δ (ppm) 8.01 (d, *J* = 8.3 Hz, 1H), 7.78 (d, *J* = 8.4 Hz, 2H), 7.61 (s, 1H), 7.54 (d, *J* = 7.9 Hz, 1H), 7.38 (t, *J* = 7.3 Hz, 1H), 7.32 – 7.22 (m, 3H), 4.00 (t, *J* = 7.0 Hz, 1H), 2.34 – 2.37 (m, 5H), 2.09 – 2.00 (m, 2H), 1.76 – 1.62 (m, 4H). ^{13}C NMR (100 MHz, $CDCl_3$) δ (ppm) 145.4, 135.2, 134.7, 130.0, 127.8, 126.8, 125.5, 123.9, 123.6, 119.3, 119.0, 118.9, 116.2, 113.9, 32.2, 28.5, 26.1, 24.7, 21.5, 16.9. HRMS (EI): m/z [M + Na]$^+$ calcd for $C_{22}H_{21}N_3NaO_2S$: 414.1246, found: 414.1239.

(S)-2-(4-((((3R,5aS,6R,8aS,9R,10S,12R,12aR)-3,6,9-trimethyldecahydro-12H-3,12-epoxy[1,2]dioxepino[4,3-i]isochromen-10-yl)oxy)methyl)phenyl)heptanedinitrile (7sa)

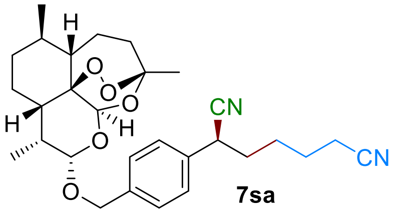

34% isolated yield, colorless oil, $[\alpha]_D^{25}$ = 7.44 (c = 1.07 in $CHCl_3$); 90:10 dr, determined by HPLC analysis (Chiralpak AD column, hexane/*i*-PrOH, 80:20 v/v, flow rate 1.0 mL/min, λ = 210 nm, 25 °C), t_R (major) = 15.48 min, t_R (minor) = 14.10 min; ^{1}H NMR (400 MHz, $CDCl_3$) δ (ppm) 7.32 (q, *J* = 8.2 Hz, 4H), 5.46 (s, 1H), 4.95 – 4.87 (m, 2H), 4.53 (d, *J* = 12.6 Hz, 1H), 3.81 (t, *J* = 7.4 Hz, 1H), 2.71 – 2.67 (m, 1H), 2.40 – 2.31 (m, 3H), 2.08 – 2.02 (m, 1H), 1.99 – 1.85 (m, 3H), 1.84 – 1.77 (m, 2H), 1.76 – 1.59 (m, 6H), 1.55 – 1.47 (m, 2H), 1.46 (s, 3H), 1.35 – 1.22 (m, 2H), 0.96 (d, *J* = 1.7 Hz, 3H), 0.95 (s, 3H). ^{13}C NMR (100 MHz, $CDCl_3$) δ (ppm) 138.6, 134.2, 127.8, 127.1, 120.3, 119.1, 104.1, 101.4, 87.9, 81.0, 69.0, 52.5, 44.3, 37.3, 36.8, 36.3, 34.9, 34.5, 30.8, 26.1, 26.1, 24.8, 24.6, 24.4, 20.2, 16.9, 13.0. HRMS (EI): m/z [M + Na]$^+$ calcd for $C_{29}H_{38}N_2NaO_5$: 517.2672, found: 517.2666.

(1R,2S,4bR,7S,9aS,10S,10aR)-10-((4-((S)-1,5-dicyanopentyl)phenoxy)carbonyl)-1-methyl-8-methylene-13-oxo-1,2,5,6,8,9,10,10a-octahydro-4a,1-(epoxymethano)-7,9a-methanobenzo[a]azulene-2,7(4bH)-diyl diacetate (7ta)

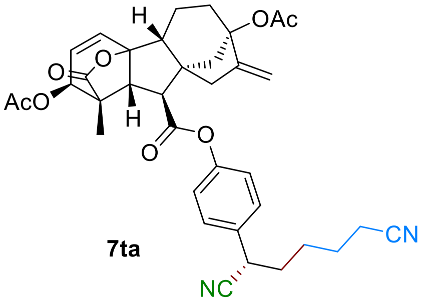

7ta

51% isolated yield, colorless oil, $[\alpha]_D^{25}$ = 12.11 (c = 1.83 in $CHCl_3$); 90:10 dr, determined by HPLC analysis (Chiralpak AD column, hexane/*i*-PrOH, 80:20 v/v, flow rate 1.0 mL/min, λ = 210 nm, 25 °C), t_R (major) = 39.56 min, t_R (minor) = 44.26 min; ^{1}H NMR (400 MHz, $CDCl_3$) δ (ppm) 7.38 (d, *J* = 8.5 Hz, 2H), 7.16 (d, *J* = 8.5 Hz, 2H), 6.41 (d, *J* = 9.3 Hz, 1H), 5.90 (dd, *J* = 9.3, 3.8 Hz, 1H), 5.37 (d, *J* = 3.8 Hz, 1H), 5.20 (s, 1H), 5.05 (s, 1H), 3.84 (t, *J* = 7.2 Hz, 1H), 3.40 (d, *J* = 11.1 Hz, 1H), 3.03 (d, *J* = 11.1 Hz, 1H), 2.61 (d, *J* = 10.8 Hz, 1H), 2.52 (dt, *J* = 14.6, 3.0 Hz, 1H), 2.43 – 2.25 (m, 5H), 2.11 (s, 3H), 2.04 (s, 3H), 2.00 – 1.81 (m, 4H), 1.79 – 1.59 (m, 6H), 1.26 (s, 3H). ^{13}C NMR (100 MHz, $CDCl_3$) δ (ppm) 176.7, 170.1, 169.8, 169.8, 152.9, 149.9, 134.0, 133.2, 129.1, 128.4, 122.1, 119.9, 119.0, 108.4, 89.7, 83.8, 69.9, 53.2, 52.0, 51.2, 50.9, 50.1, 42.6, 39.2, 36.5, 36.4, 34.9, 26.0, 24.7, 21.9, 20.7, 16.9, 16.6, 14.4. HRMS (EI): m/z $[M + Na]^+$ calcd for $C_{36}H_{38}N_2NaO_8$: 649.2520, found: 649.2501.

(S)-2-([1,1'-biphenyl]-4-yl)-4-(cyanomethoxy)butanenitrile (7ib)

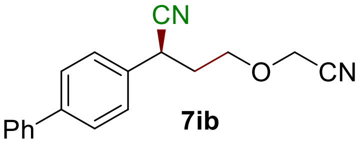

7ib

24% isolated yield, colorless oil, $[\alpha]_D^{25}$ = 3.60 (c = 0.38 in $CHCl_3$); 89% ee, determined by HPLC analysis (Chiralpak AD column, hexane/*i*-PrOH, 80:20 v/v, flow rate 1.0 mL/min, λ = 254 nm, 25 °C), t_R (major) = 10.77 min, t_R (minor) = 11.86 min; ^{1}H NMR (400 MHz, $CDCl_3$) δ (ppm) 7.65 – 7.55 (m, 4H), 7.49 – 7.34 (m, 5H), 4.38 – 4.20 (m, 2H), 4.06 (t, *J* = 7.8 Hz, 1H), 3.78 – 3.73 (m, 1H), 3.70 – 3.65 (m, 1H), 2.36 – 2.14 (m, 2H). ^{13}C NMR (100 MHz, $CDCl_3$) δ (ppm) 141.4, 140.0, 133.6, 128.8, 127.9, 127.7, 127.6, 127.0, 120.2, 115.5, 67.8, 56.4, 35.3, 33.3. HRMS (EI): m/z $[M + Na]^+$ calcd for $C_{18}H_{16}N_2NaO$: 299.1154, found: 299.1152.

benzyl (S)-(3-([1,1'-biphenyl]-4-yl)-3-cyanopropyl)(cyanomethyl)carbamate (7ic)

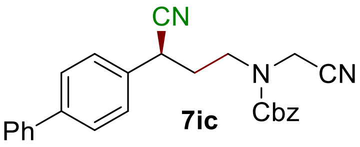

7ic

70% isolated yield, yellow oil, $[\alpha]_D^{25}$ = -0.95 (c = 1.05 in $CHCl_3$); 87% ee, determined by HPLC analysis (Chiralpak AD column, hexane/i-PrOH, 80:20 v/v, flow rate 1.0 mL/min, λ = 254 nm, 25 °C), t_R (major) = 17.19 min, t_R (minor) = 20.17 min; ^{1}H NMR (400 MHz, $CDCl_3$) δ (ppm) 7.56 (d, *J* = 7.4 Hz, 4H), 7.45 (t, *J* = 7.5 Hz, 2H), 7.39 – 7.32 (m, 8H), 5.20 (s, 2H), 4.37 – 4.05 (m, 2H), 3.98 – 3.78 (m, 1H), 3.65 – 3.58 (m, 2H), 2.26 (s, 2H). ^{13}C NMR (100 MHz, $CDCl_3$) δ (ppm) 155.2, 141.4, 139.9, 135.3, 133.3, 128.8, 128.6, 128.5, 128.3, 127.9, 127.7, 127.5, 127.0, 119.8, 115.5, 68.6, 45.6, 36.1, 34.4, 33.6. HRMS (EI): m/z $[M + Na]^+$ calcd for $C_{26}H_{23}N_3NaO_2$: 432.1682, found: 432.1674.

benzyl ((5S)-5-([1,1'-biphenyl]-4-yl)-1,5-dicyanopentan-2-yl)carbamate (7id)

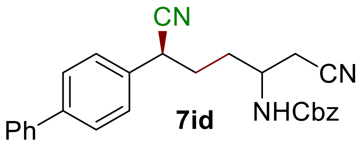

7id

65%, 1:1 d.r., isolated yield, colorless oil, $[\alpha]_D^{25}$ = -2.62 (c = 1.08 in $CHCl_3$); 86% ee, 83% ee, determined by HPLC analysis (Chiralpak AD column, hexane/*i*-PrOH, 90:10 v/v, flow rate 0.5 mL/min, λ = 254 nm, 25 °C), t_R (major) = 68.75 min, t_R (minor) = 107.74 min, t_R (major) = 91.00 min, t_R (minor) = 85.30 min; ^{1}H NMR (400 MHz, $CDCl_3$) δ (ppm, major + minor) 7.67 – 7.53 (m, 4H), 7.45 (t, *J* = 7.5 Hz, 2H), 7.42 – 7.26 (m, 8H), 5.17 – 4.99 (m, 3H), 4.05 – 3.79 (m, 2H), 2.76 – 2.69 (m, 1H), 2.58 – 2.50 (m, 1H), 2.10 – 1.91 (m, 2H), 1.89 – 1.71 (m, 2H). ^{13}C NMR (100 MHz, $CDCl_3$) δ (ppm, major + minor) 155.7, 155.6, 141.4, 141.4, 140.0, 135.8, 133.8, 133.6, 128.9, 128.6, 128.4, 128.1, 127.9, 127.7, 127.6, 127.5, 127.0, 120.1, 120.1, 116.7, 77.2, 77.0, 76.8, 67.3, 47.4, 47.0, 36.4, 36.3, 32.3, 32.1, 31.2, 30.8, 24.3, 24.2. HRMS (EI): m/z $[M + Na]^+$ calcd for $C_{27}H_{25}N_3NaO_2$: 446.1838, found: 446.1833.

tert-butyl (S)-3-(3-([1,1'-biphenyl]-4-yl)-3-cyanopropyl)-3-(cyanomethyl)azetidine-1-carboxylate (7ie)

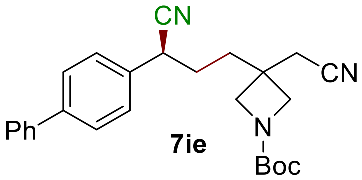

7ie

58% isolated yield, white solid, $[\alpha]_D^{25}$ = -5.31 (c = 1.18 in $CHCl_3$); 92% ee, determined by HPLC analysis (Chiralpak AD column, hexane/*i*-PrOH, 80:20 v/v, flow rate 1.0 mL/min, λ = 254 nm, 25 °C), t_R (major) = 14.92 min, t_R (minor) = 38.93 min; ^{1}H NMR (400 MHz, $CDCl_3$) δ (ppm) 7.61 (dd, *J* = 20.0, 7.9 Hz, 4H), 7.48 – 7.36 (m, 5H), 3.90 (t, *J* = 6.4 Hz, 1H), 3.75 – 3.70 (m, 4H), 2.63 (s, 2H), 2.00 – 1.85 (m, 4H), 1.43 (s, 9H). ^{13}C NMR (100 MHz, $CDCl_3$) δ (ppm) 155.9, 141.6, 139.9, 133.5, 128.8, 128.0, 127.7, 127.5, 127.0, 119.9, 116.5, 80.3, 57.5, 57.4, 36.9, 35.0, 33.7, 30.6, 28.2, 26.0. HRMS (EI): m/z $[M + Na]^+$ calcd for $C_{26}H_{29}N_3NaO_2$: 438.2151, found: 438.2150.

tert-butyl (S)-4-(3-([1,1'-biphenyl]-4-yl)-3-cyanopropyl)-4-(cyanomethyl)piperidine-1-carboxylate (7if)

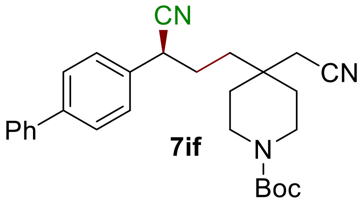

7if

49% isolated yield, white solid, $[\alpha]_D^{25}$ = -4.43 (c = 0.88 in $CHCl_3$); 90% ee, determined by HPLC analysis (Chiralpak AD column, hexane/*i*-PrOH, 80:20 v/v, flow rate 1.0 mL/min, λ = 220 nm, 25 °C), t_R (major) = 9.58 min, t_R (minor) = 12.40 min; ^{1}H NMR (400 MHz, $CDCl_3$) δ (ppm) 7.60 (dd, *J* = 17.1, 7.7 Hz, 4H), 7.49 – 7.34 (m, 5H), 3.85 (t, *J* = 7.1 Hz, 1H), 3.46 – 3.40 (m, 2H), 3.38 – 3.27 (m, 2H), 2.36 (s, 2H), 2.01 – 1.85 (m, 2H), 1.82 – 1.74 (m 1H), 1.70 – 1.62 (m, 2H), 1.56 – 1.48 (m, 4H), 1.44 (s, 9H). ^{13}C NMR (100 MHz, $CDCl_3$) δ (ppm) 154.5, 141.4, 140.0, 133.8, 128.8, 127.9, 127.6, 127.5, 127.0, 120.1, 117.0, 79.9, 39.3, 39.1, 37.2, 34.2, 34.1, 34.0, 33.8, 29.6, 28.3, 25.7. HRMS (EI): m/z $[M + Na]^+$ calcd for $C_{28}H_{33}N_3NaO_2$: 466.2465, found: 466.2474.

(S)-6-([1,1'-biphenyl]-4-yl)-2,2,4,4-tetramethyl-3-oxoheptanedinitrile (7ig)

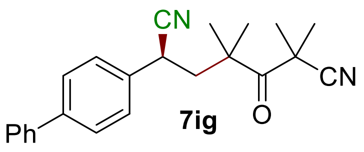

53% isolated yield, colorless oil, $[\alpha]_D^{25}$ = -12.17 (c = 0.84 in $CHCl_3$); 85% ee, determined by HPLC analysis (Chiralpak AZ column, hexane/*i*-PrOH, 80:20 v/v, flow rate 1.0 mL/min, λ = 254 nm, 25 °C), t_R (major) = 13.42 min, t_R (minor) = 11.21 min; ^1H NMR (400 MHz, $CDCl_3$) δ (ppm) 7.64 – 7.54 (m, 4H), 7.50 – 7.42 (m, 4H), 7.40 – 7.33 (m, 1H), 3.83 (dd, *J* = 10.2, 4.1 Hz, 1H), 2.38 (dd, *J* = 14.4, 10.2 Hz, 1H), 2.22 (dd, *J* = 14.4, 4.1 Hz, 1H), 1.64 – 1.53 (m, 12H). ^{13}C NMR (100 MHz, $CDCl_3$) δ (ppm) 207.1, 141.2, 140.1, 135.4, 128.8, 127.9, 127.7, 127.6, 127.0, 123.0, 121.1, 49.5, 45.9, 40.2, 33.1, 26.7, 26.6, 24.9, 24.1. HRMS (EI): m/z $[M + Na]^+$ calcd for $C_{23}H_{24}N_2NaO$: 367.1780, found: 367.1772.

4.4 Investigation of reaction system of cyclic acyl precusor 3q

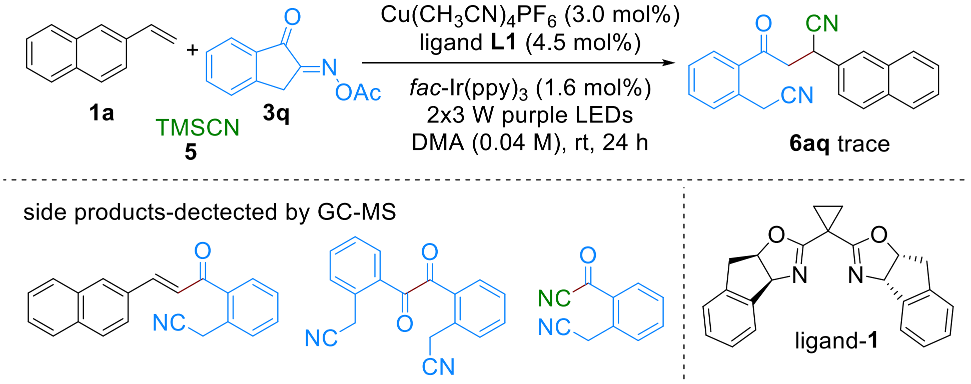

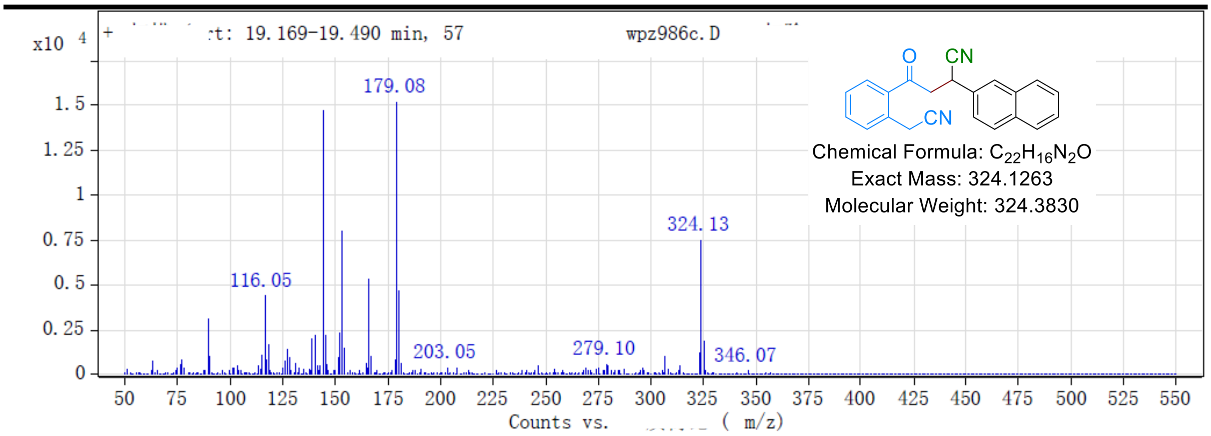

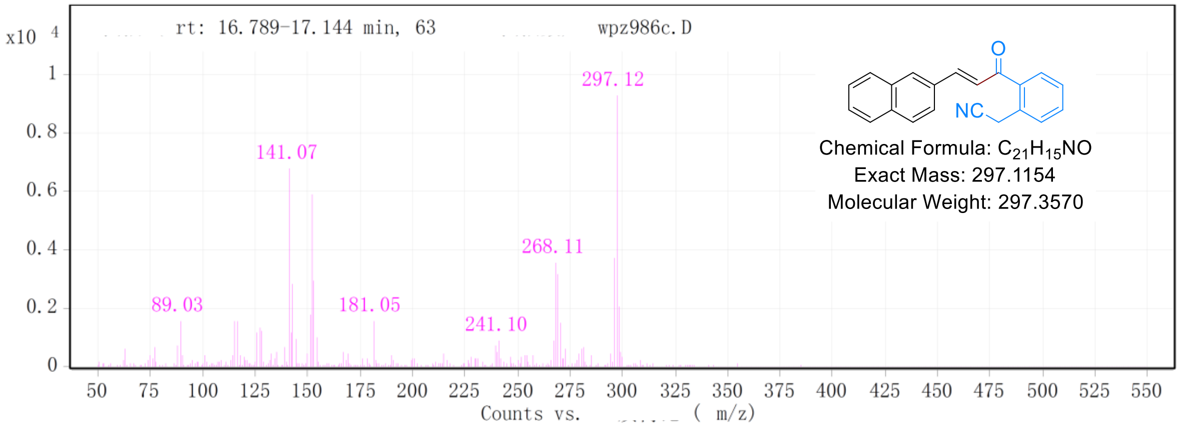

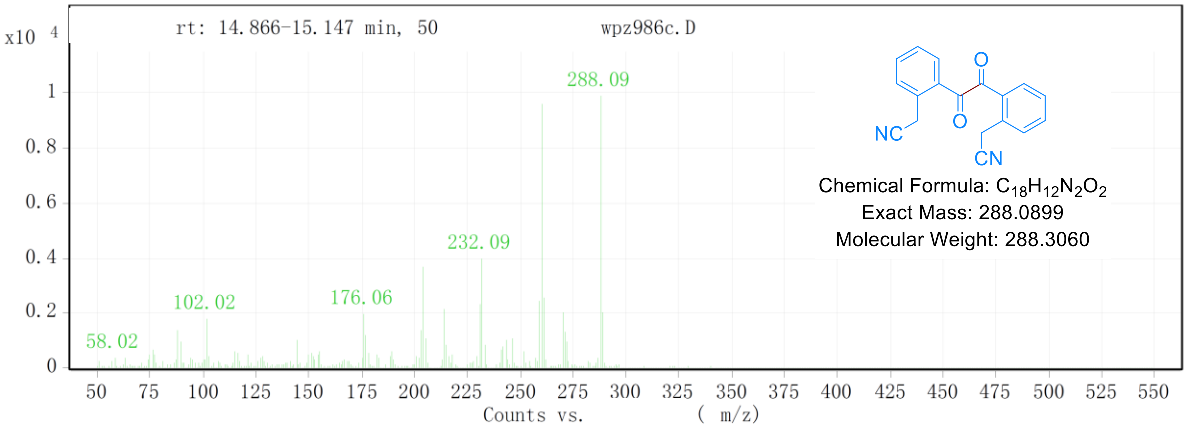

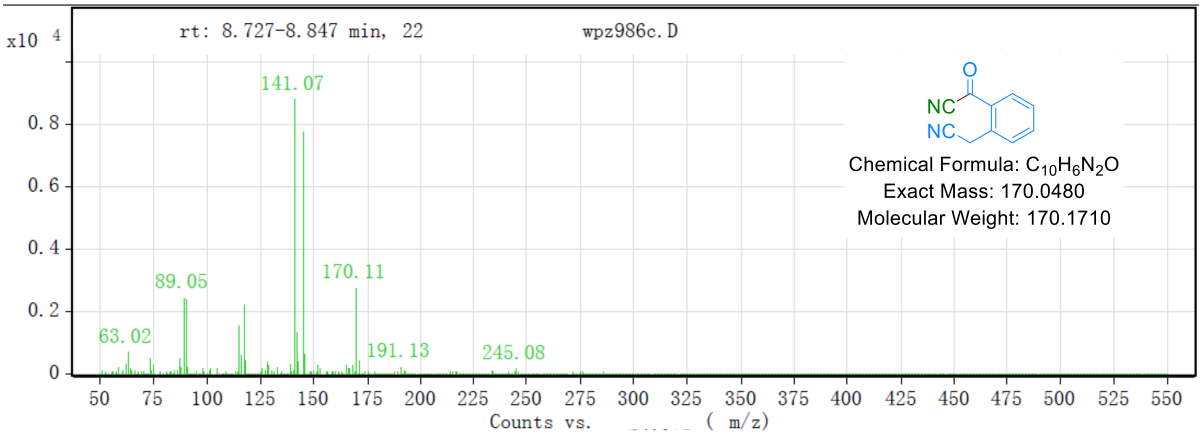

Spectral data of acyl radical precursor 3q

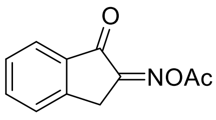

3q

^{1}H NMR (400 MHz, CDCl$_3$) δ (ppm) 7.91 (d, J = 7.7 Hz, 1H), 7.72 (t, J = 7.1 Hz, 1H), 7.57 – 7.45 (m, 2H), 3.97 (s, 2H), 2.36 (s, 3H). ^{13}C NMR (100 MHz, CDCl$_3$) δ (ppm) 187.9, 168.8, 159.2, 146.2, 137.3, 136.8, 128.5, 126.8, 125.0, 29.8, 19.7.

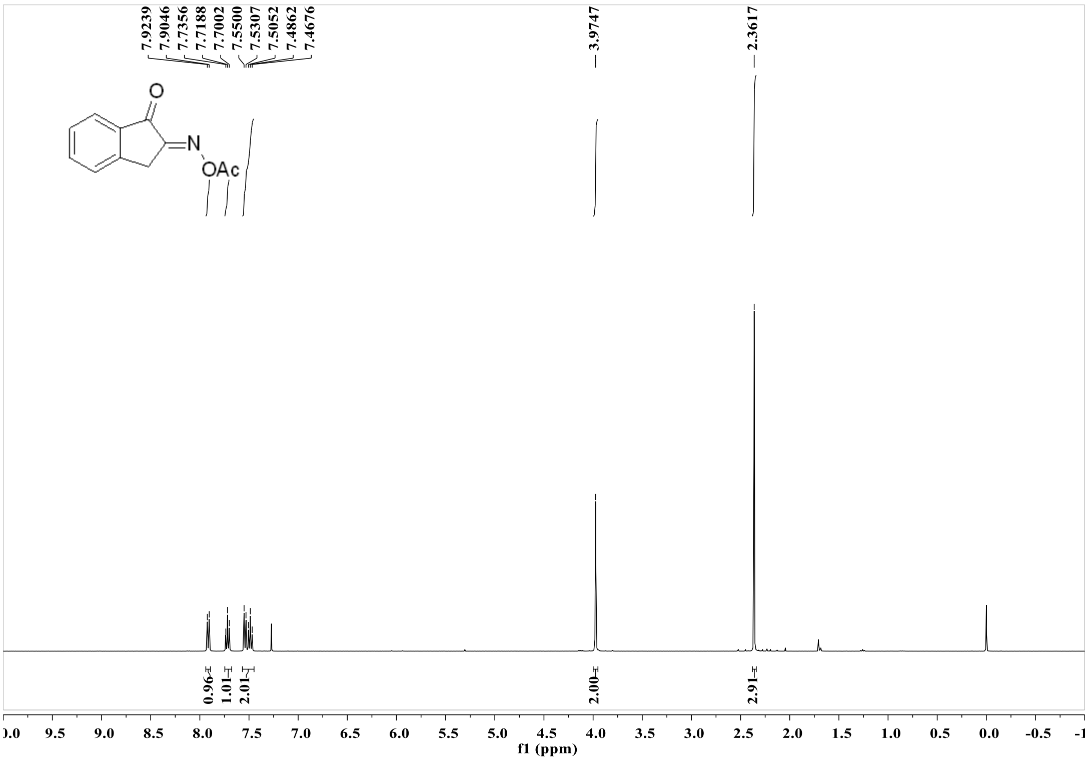
7.9239
7.9046
7.7356
7.7188
7.7002
7.5500
7.5307
7.5052
7.4862
7.4676
3.9747
2.3617
OAc
0.96
1.01
2.01
2.00
2.91
f1 (ppm)

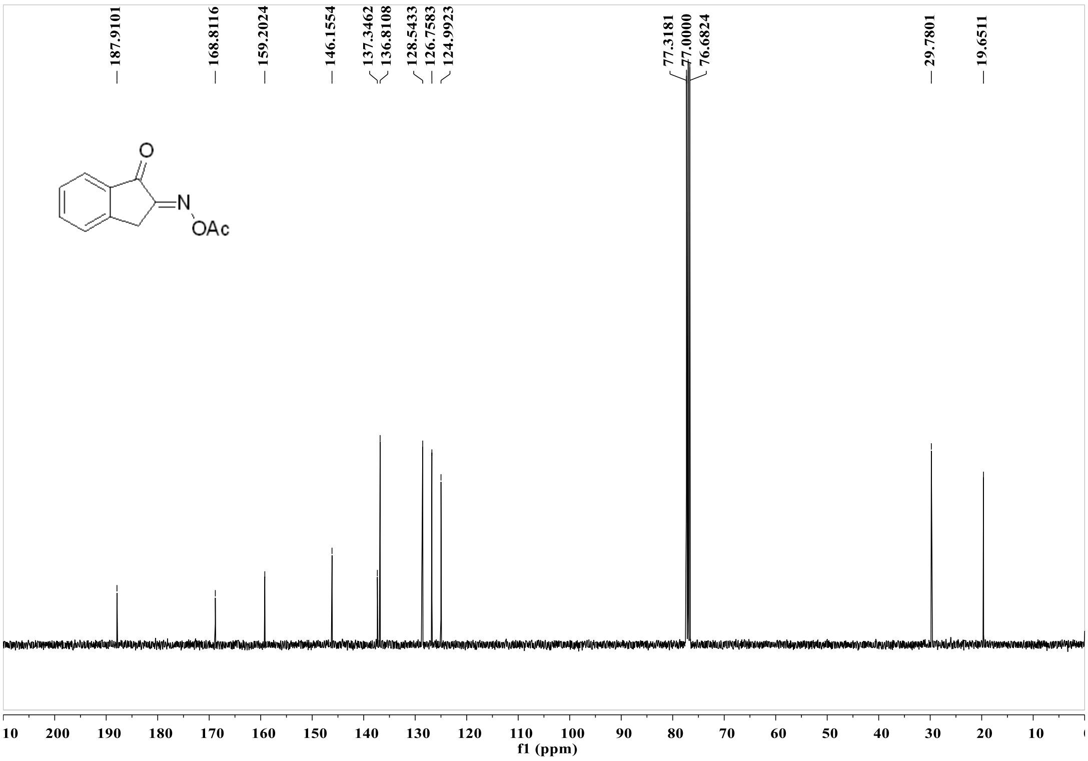
187.9101
168.8116
159.2024
146.1554
137.3462
136.8108
128.5433
126.7583
124.9923
77.3181
77.0000
76.6824
29.7801
19.6511
OAc
f1 (ppm)

5. Synthetic Applications of the Reaction

5.1 1.0 mmol Reaction

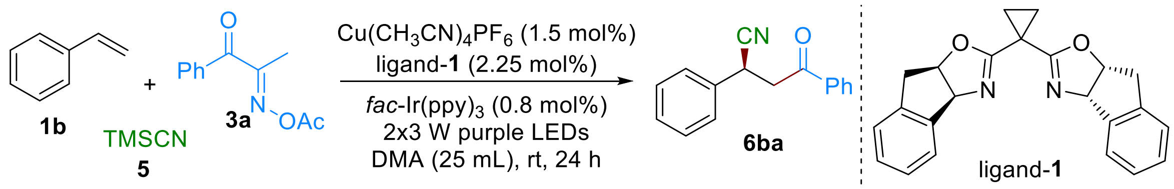

In a flame-dried 50 mL Schlenk flask equipped with a magnetic stirrer bar was charged sequentially with $Cu(CH_3CN)_4PF_6$ (5.6 mg, 0.015 mmol) and ligand-**1** (8.0 mg, 0.0225 mmol), followed by the addition of DMA (25 mL). Then the mixture was stirred at room temperature for 30 min. To the resulting mixture were added **3a** (620 mg, 3.0 mmol), *fac*-Ir(ppy)$_3$ (5.3 mg, 0.008 mmol). Then, the resulting mixture was degassed (3 times) under argon atmosphere. After that, TMSCN (3.0 mmol) and **1b** (104 mg, 1.0 mmol) were added into the mixture. At last, the mixture was stirred at a distance of ~1 cm from a 2 x 3 W purple LEDs at room temperature for 24 h until the reaction was completed, as monitored by TLC analysis. The reaction mixture was quenched with water(30 mL), diluted with EtOAc (3 x 30 mL), washed with NaCl (aq) and dried over with anhydrous Na_2SO_4. After filtration and concentration, the residue was purified by silica gel chromatography with petroleum ether and ethyl acetate (PE/EA = 5:1) to afford **6ba** in 74% yield, 90% ee.

5.2 Gram-Scale Reaction

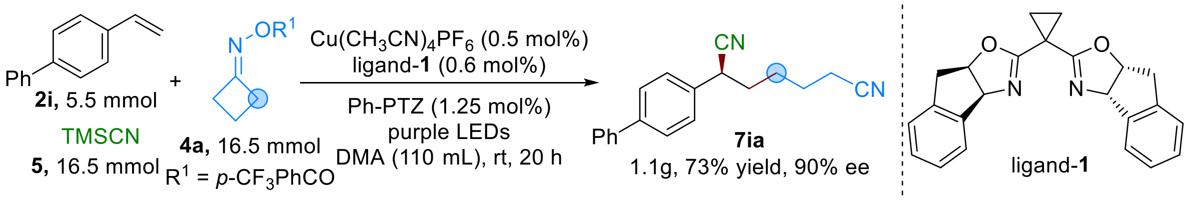

In a flame-dried 150 mL Schlenk flask equipped with a magnetic stirrer bar was charged sequentially with $Cu(CH_3CN)_4PF_6$ (10.2 mg, 0.027 mmol), ligand-**1** (11.7 mg, 0.033 mmol), and organo-photocatalyst Ph-PTZ (19.0 mg, 0.069 mmol) followed by the addition of DMA (110 mL). Then the mixture was stirred at room temperature for 30 min. To the resulting mixture were added **2i** (5.5 mmol), **4a** (16.5 mmol). Then, the resulting mixture was degassed (3 times) under argon atmosphere. After that, TMSCN (16.5 mmol) was added into the mixture. At last, the mixture was stirred at a distance of ~1 cm from purple LEDs at room temperature about 20 h until the reaction was completed, as monitored by TLC analysis. The reaction mixture was diluted with water (100 mL). The mixture was firstly extracted with EtOAc (3 x 150 mL), then washed with $NaHCO_3$ (aq.) (150 mL), and finally washed with NaCl (aq), dried over with anhydrous Na_2SO_4. After filtration and concentration, the residue was purified by silica gel chromatography with petroleum ether and ethyl acetate (PE/EA = 7:1) to afford final product in 73% isolated yield (1.1 g) and 90% ee.

5.3 Transfomations of Products 6ba and 7ia

a). The procedure for the synthesis of amide

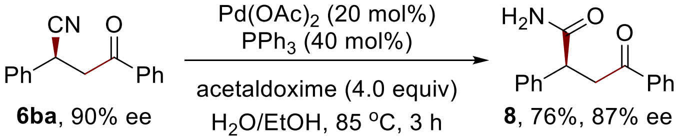

A mixture of **6ba** (0.1 mmol, 23.5 mg), acetaldoxime (0.4 mmol, 24 μL), $Pd(OAc)_2$ (0.02 mmol, 4.5 mg), and PPh_3 (0.04 mmol, 10.5 mg) in aqueous EtOH ($EtOH/H_2O$ = 4/1, 1.5 mL) was heated to 85°C in a sealed tube, the reaction was stirred for 3h under argon atmosphere. The reaction mixture was filtered through a Celite pad and washed with EtOH/DCM. After removal of solvent and column chromatographic purification process (DCM/MeOH), get product **8** as a white solid (19.2 mg, 76% yield, 87% ee). $[\alpha]_D^{25}$ = -26.37 (c = 0.50 in $CHCl_3$); 87% ee, determined by HPLC analysis (Chiralpak AZ column, hexane/*i*-PrOH, 85:15 v/v, flow rate 1.0 mL/min, λ = 254 nm, 25 ºC), t_R (major) = 32.42 min, t_R (minor) = 23.99 min, ^1H NMR (400 MHz, $CDCl_3$) δ (ppm) 7.97 (d, *J* = 7.5 Hz, 2H), 7.55 (t, *J* = 7.4 Hz, 1H), 7.48 – 7.25 (m, 10H), 5.61 (d, *J* = 27.3 Hz, 2H), 4.23 (dd, *J* = 8.9, 4.6 Hz, 1H), 4.06 (dd, *J* = 17.9, 9.0 Hz, 1H), 3.23 (dd, *J* = 17.9, 4.6 Hz, 1H). ^{13}C NMR (100 MHz, $CDCl_3$) δ (ppm) 198.1, 174.8, 139.4, 136.5, 133.2, 129.0, 128.5, 128.1, 127.9, 127.6, 77.3, 77.0, 76.7, 47.2, 42.5. HRMS (EI): m/z $[M + Na]^+$ calcd for $C_{16}H_{25}NNaO_2$: 276.0995, found: 276.0993.

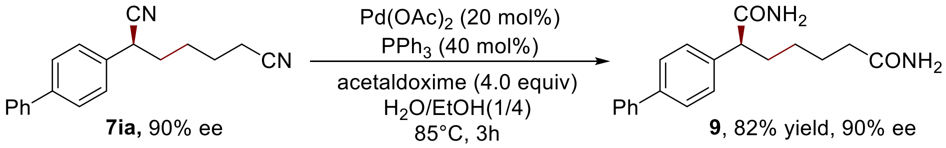

A mixture of **7ia** (0.1 mmol, 27.4 mg), acetaldoxime (0.4 mmol, 24 μL), $Pd(OAc)_2$ (0.02 mmol, 4.5 mg), and PPh_3 (0.04 mmol, 10.5 mg) in aqueous EtOH ($EtOH/H_2O$ = 4/1, 1.5 mL) was heated to 85°C in a sealed tube, the reaction was stirred for 3 h under argon atmosphere. The reaction mixture was filtered through a Celite pad and washed with EtOH/DCM. After removal of solvent and column chromatographic purification process (DCM/MeOH), get product **9** as a white solid (25.6 mg, 82% yield, 90% ee). $[\alpha]_D^{25}$ = 35.36 (c = 0.61 in $CHCl_3$); 90% ee, determined by HPLC analysis (Chiralpak AD column, hexane/*i*-PrOH, 80:20 v/v, flow rate 1.0 mL/min, λ = 254 nm, 25 ºC), t_R (major) = 15.03 min, t_R (minor) = 31.10 min; ^1H NMR (400 MHz, DMSO-d_6) δ (ppm) 7.68 – 7.54 (m, 4H), 7.51 – 7.30 (m, 6H), 7.23 (s, 1H), 6.84 (s, 1H), 6.68 (s, 1H), 3.45 (t, *J* = 7.3 Hz, 1H), 2.07 – 1.98 (m, 2H), 1.97 – 1.90 (m, 1H), 1.66 – 1.57 (m, 1H), 1.54 – 1.47 (m, 2H), 1.23 – 1.14 (m, 2H). ^{13}C NMR (100 MHz, DMSO-d_6) δ (ppm) 174.7, 174.3, 140.5, 140.1, 138.5, 129.0, 128.3, 127.3, 126.6, 126.5, 50.7, 35.1, 32.7, 27.0, 25.1. HRMS (EI): m/z $[M + Na]^+$ calcd for $C_{19}H_{22}N_2NaO_2$: 333.1573, found: 333.1575.

b). The procedure for the reduction of nitriles to Boc amines

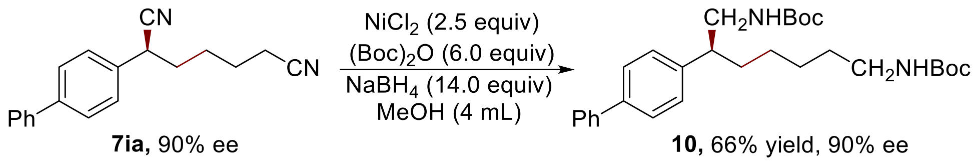

A mixture of **7ia** (0.1 mmol, 27.4 mg), $NiCl_2$ (0.25 mmol, 32.4 mg), $(Boc)_2O$ (0.6 mmol, 138 μL) in dry methanol (4 mL) was cooled to 0°C, Then $NaBH_4$ (1.4 mmol, 52.9 mg) was added in small portions. The miture was allowed to stirred overnight at room temperature. The reaction was quenched with a saturated aqueous solution of NH_4Cl and extracted with EtOAc. The combined organic phase were dried over Na_2SO_4, filtered and concentrated under vacuum. Purification by column chromatography on silica gel, get product **10** as colorless oil (32.1 mg, 66% yield, 90% ee). $[\alpha]_D^{25}$ = -13.25 (c = 0.80 in $CHCl_3$); 90% ee, determined by HPLC analysis (Chiralpak AD column, hexane/*i*-PrOH, 90:10 v/v, flow rate 0.5 mL/min, λ = 254 nm, 25 °C), t_R (major) = 21.30 min, t_R (minor) = 18.94 min; ^1H NMR (400 MHz, $CDCl_3$) δ (ppm) 7.62 – 7.52 (m, 4H), 7.47 – 7.40 (m, 2H), 7.37 – 7.30 (m, 1H), 7.22 (d, *J* = 7.9 Hz, 2H), 4.46 (d, *J* = 27.6 Hz, 2H), 3.53 (dt, *J* = 12.9, 6.4 Hz, 1H), 3.19 – 3.12 (m, 1H), 3.05 (q, *J* = 6.7 Hz, 2H), 2.83 – 2.69 (m, 1H), 1.73 – 1.55 (m, 3H), 1.43 (s, 9H), 1.40 (s, 9H), 1.32 – 1.19 (m, 5H). ^{13}C NMR (100 MHz, $CDCl_3$) δ (ppm) 155.9, 155.8, 141.8, 140.7, 139.4, 128.7, 128.1, 127.2, 127.1, 126.9, 79.1, 78.9, 46.2, 45.7, 40.4, 33.4, 29.8, 28.3, 28.3, 26.9, 26.7. HRMS (EI): m/z $[M + Na]^+$ calcd for $C_{29}H_{42}N_2NaO_4$: 505.3036, found: 505.3036.

c). The procedure for the esterification of nitriles

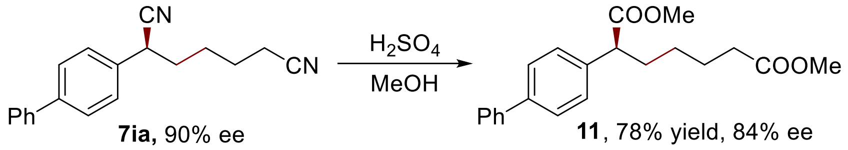

To a solution of **7ia** (0.1 mmol, 27.4 mg) in methanol (1.6 mL) at 0°C was added concentrated sulphuric acid (0.6 mL), the reaction was heated to 70°C in a sealed tube and stirred overnight under argon atmosphere. The reaction was quenched with cold water and extracted with EtOAc. The combined organic phase were dried over Na_2SO_4, filtered and concentrated under vacuum. Purification by column chromatography on silica gel, get product **11** as colorless oil (26.6 mg, 78% yield, 84% ee). $[\alpha]_D^{25}$ = 2.91 (c = 0.71 in $CHCl_3$); 84% ee, determined by HPLC analysis (Chiralpak AD column, hexane/*i*-PrOH, 95:5 v/v, flow rate 0.5 mL/min, λ = 254 nm, 25 °C), t_R (major) = 20.84 min, t_R (minor) = 22.40 min; ^1H NMR (400 MHz, $CDCl_3$) δ (ppm) 7.61 – 7.50 (m, 4H), 7.45 – 7.41 (m, 2H), 7.38 – 7.30 (m, 3H), 3.67 (s, 3H), 3.64 (s, 3H), 3.59 (t, *J* = 7.7 Hz, 1H), 2.29 (t, *J* = 7.5 Hz, 2H), 2.16 – 2.07 (m, 1H), 1.87 – 1.78 (m, 1H), 1.72 – 1.61 (m, 2H), 1.39 – 1.25 (m, 2H). ^{13}C NMR (100 MHz, $CDCl_3$) δ (ppm) 174.3, 173.9, 140.6, 140.1, 137.9, 128.7, 128.2, 127.3, 127.2, 127.0, 52.0, 51.4, 51.0, 33.7, 33.0, 27.0, 24.6. HRMS (EI): m/z $[M + Na]^+$ calcd for $C_{21}H_{24}NaO_4$: 363.1566, found:

363.1558.

6. The Mechanism Studies

6.1 Luminescence Quenching Experiments

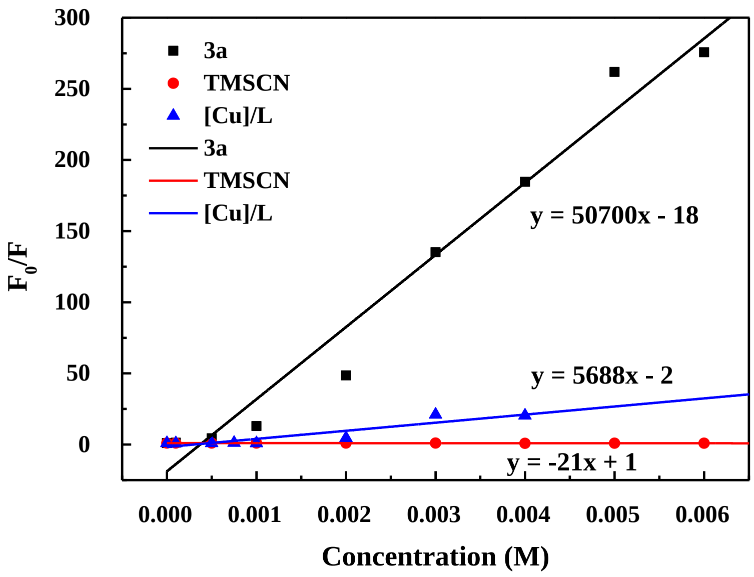

Figure S1. *fac*-Ir(ppy)$_3$ emission quenching by **3a**, TMSCN and Cu(CH_3CN)$_4$$PF_6$/ ligand-**1**

Fluorescence spectra was collected on Agilent Fluorescence Spectrophotometer G9800AS24 for all experiments. All *fac*-Ir(ppy)$_3$ solutions were excited at 288 nm and the emission intensity was collected at 519 nm. In a typical experiment, the emission spectrum of a 1×10^{-5} M solution of *fac*-Ir(ppy)$_3$ in DMA was collected. The significant decrease of *fac*-Ir(ppy)$_3$ luminescence could be observed in the presence of substrate **3a**. And a slightly decrease of *fac*-Ir(ppy)$_3$ luminescence was observed in the presence of Cu(I)/ligand-**1** catalyst and TMSCN.

6.2 UV-Vis absorption spectra

UV-Vis absorption spectra were collected on a Agilent Technologies Cary 60 UV-Vis. All samples were dissolved in DMA. The UV-Vis absorption of **1a** ($1*10^{-3}$ M), **3a** ($1*10^{-3}$ M), *fac*-Ir(ppy)$_3$ ($8*10^{-5}$ M), Cu(CH_3CN)$_4$$PF_6$ ($1.5*10^{-4}$ M), [Cu] /ligand-**1**, [Cu]/ligand-**1**/PC and reaction mixture were showed in the figure below.

It was found that **3a**, **1a**, Cu(CH_3CN)$_4$$PF_6$, and Cu($CH_3CN$)$_4$$PF_6$/ ligand-**1** did not show any very strong absorption bands around the visible region. In contrast, the UV-Vis spectra of photocatalyst, mixture of Cu(CH_3CN)$_4$$PF_6$/ ligand-**1**/PC and reaction mixture have the same absorption band at 375 nm, which implied that photocatalyst *fac*-Ir(ppy)$_3$ could be more efficiently excited by purple LEDs (λmax = 390 nm) than by blue LEDs (λmax = 460 nm).

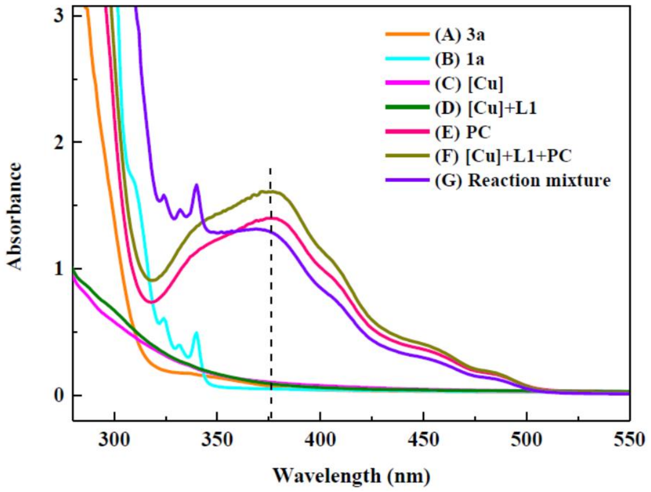

Figure S2. Absorption spectra in DMA of (A) **3a**; (B) **1a**; (C) $Cu(CH_3CN)_4PF_6$; (D) $Cu(CH_3CN)_4PF_6$/**L1**; (E) *fac*-Ir(ppy)$_3$; (F) $Cu(CH_3CN)_4PF_6$/**L1**/*fac*-Ir(ppy)$_3$; (G) reaction mixture.

6.3 Light On-Off Experiments

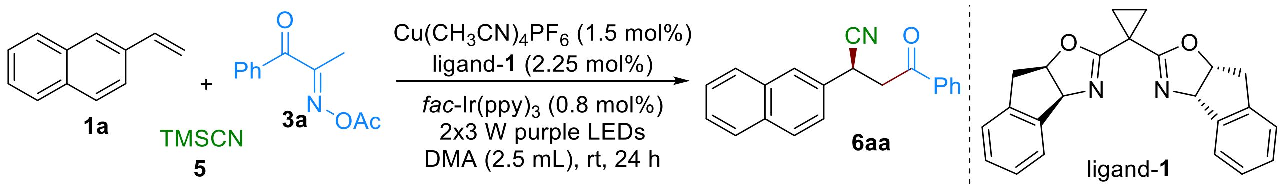

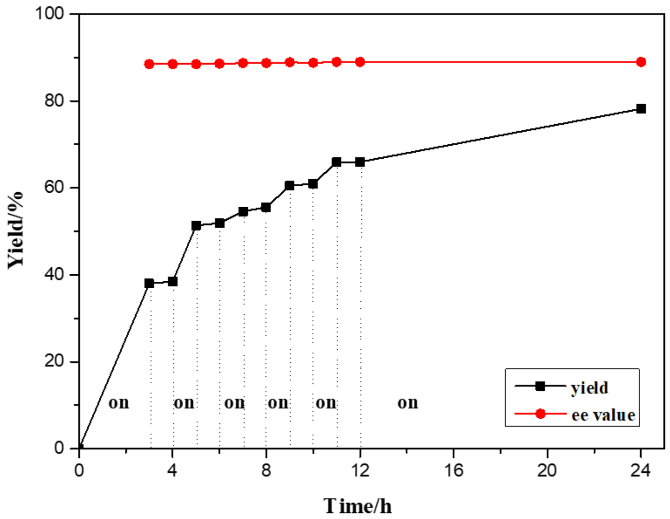

Figure S3. Light on-off experiments

The yield of **6aa** was determined by GC using 1,3,5-trimethoxybenzene as an internal standard.

The results revealed that a radical chain process was not the major reaction pathway, while it could not be completely ruled out at the current stage.

6.4 Determination of Quantum Yields

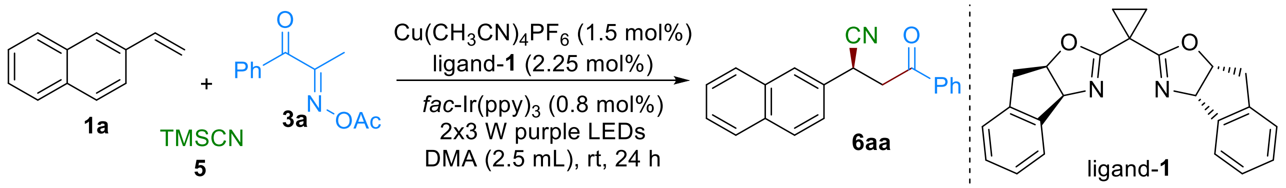

In a flame-dried 10 mL Schlenk tube equipped with a magnetic stirrer bar was charged sequentially with $Cu(CH_3CN)_4PF_6$ (1.12mg, 0.003 mmol) and ligand-**1** (1.60 mg, 0.005 mmol), followed by the addition of DMA (5 mL). Then the mixture was stirred at room temperature for 30 min. To the resulting mixture were added **3a** (124 mg, 0.6 mmol), *fac*-Ir(ppy)$_3$ (1.06 mg, 0.016 mmol) and **1a** (30.8 mg, 0.20 mmol). Then, the resulting mixture was degassed (3 times) under argon atmosphere. After that, TMSCN (0.6 mmol) were added into the mixture. At last, the solution was removed into a cuvette in an argon-filled glove box. The sample was irradiated (λ = 395 nm, slit width = 3.0 mm, slit height 5.0 mm with intensity of 0.353 mW•cm^{-2}) for 13915 s. After irradiation, the yield of product formed was determined by GC based on a 1,3,5-trimethoxybenzene standard. The quantum yield was determined as follows.

ϕ = Mole number for product/Mole number for absorption of photons = 0.336

$$\Phi = \frac{n_{3a}\, N_A/t}{f\, P\, \lambda/hc}$$

n_{3a}: the mole number of the product **6aa**; t: reaction time (13915 s); N_A: 6.02×10^{23}/mol; f: $1\text{-}10^{-A}$ (455 nm, A = 1.702); P: P = E*S (E: illumination intensity, E = 0.353 mW/cm^2; S: the area that irradiated S = 0.15 cm^2); λ: wavelength ($\lambda = 3.95 \times 10^{-7}$ m); h: planck constant (h = 6.626×10^{-34} J*s); c: velocity of light (c = 3×10^8 m/s).

This result reveals that the radical chain process is not main pathway.

6.5 Non-Linear Effect Experiments

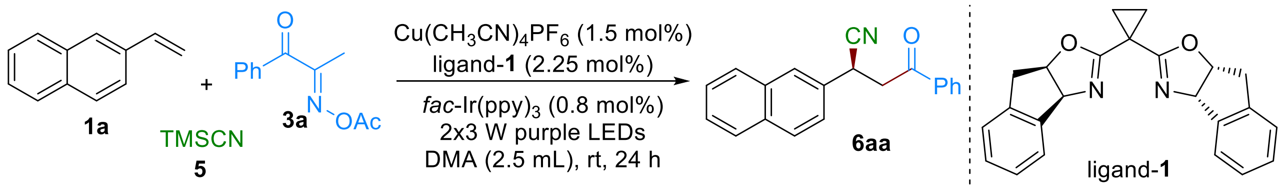

These reactions were conducted according to the general procedure: In a flame-dried 10 mL Schlenk tube equipped with a magnetic stirrer bar was charged sequentially with $Cu(CH_3CN)_4PF_6$ (0.56 mg, 0.0015 mmol) and ligand-**1** (0.80 mg, 0.00225 mmol, x% ee), followed by the addition of DMA (2.5 mL). Then the mixture was stirred at room temperature for 30 min. To the resulting mixture were added **1a** (62 mg, 0.30 mmol), **3a** (15 mg, 0.10 mmol), *fac*-Ir(ppy)$_3$ (0.53 mg, 0.0008 mmol). Then, the resulting mixture was degassed (3 times) under argon atmosphere. After

that, TMSCN (0.3 mmol) was added into the mixture. At last, the mixture was stirred at a distance of ~1 cm from 2 x 3 W purple LEDs at room temperature for 24 h until the reaction was completed, as monitored by TLC analysis.

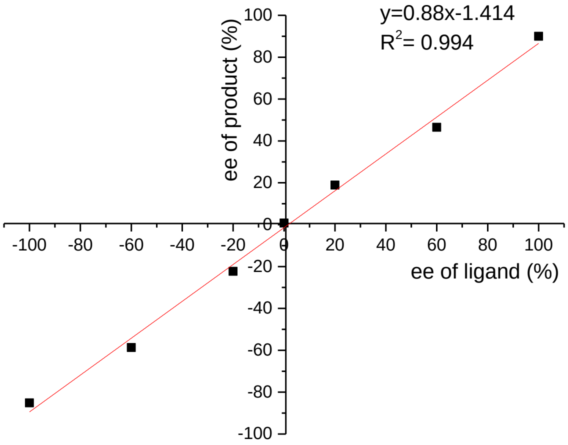

Figure S4. Relationship between ee values of ligand and product

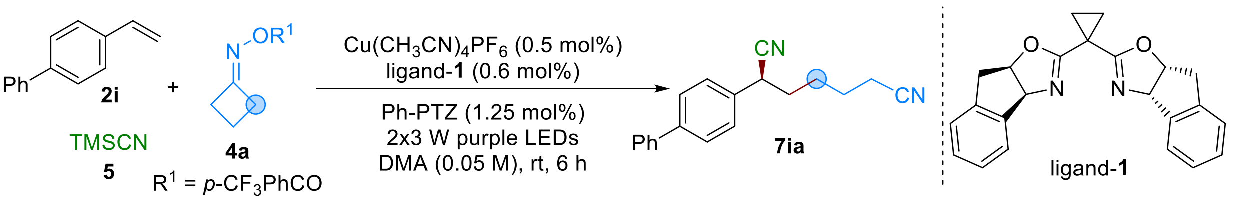

These reactions were conducted according to the general procedure: In a flame-dried 10 mL Schlenk tube equipped with a magnetic stirrer bar was charged sequentially with $Cu(CH_3CN)_4PF_6$ (0.001 mmol), ligand-**1** (0.0012 mmol, x% ee) and organo-photocatalyst Ph-PTZ (0.0025 mmol) followed by the addition of DMA (4.0 mL). Then the mixture was stirred at room temperature for 30min. The mixture were added **2i** (0.20 mmol), **4a** (0.6 mmol). Then, the resulting mixture was degassed (3 times) under argon atmosphere. After that, TMSCN (0.6 mmol) was added into the mixture. At last, the mixture was stirred at a distance of ~1cm from a 2 x 3 W purple LEDs at rt about 6 h until the reaction was completed, as monitored by TLC analysis.

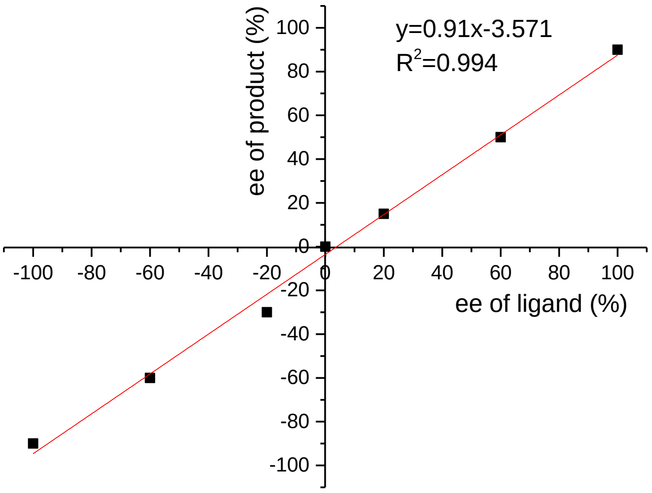

Figure S5. Relationship between ee values of ligand and product

6.6 Radical Trapping Experiments

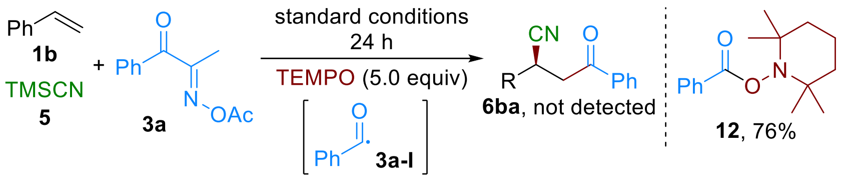

In a flame-dried 10 mL Schlenk tube equipped with a magnetic stirrer bar was charged sequentially with $Cu(CH_3CN)_4PF_6$ (0.56 mg, 0.0015 mmol) and ligand-**1** (0.80 mg, 0.00225 mmol), followed by the addition of DMA (2.5 mL). Then the mixture was stirred at room temperature for 30 min. To the resulting mixture were added **3a** (62 mg, 0.30 mmol), *fac*-Ir(ppy)$_3$ (0.53 mg, 0.0008 mmol) and TEMPO (0.50 mmol). Then, the resulting mixture was degassed (3 times) under argon atmosphere. After that, TMSCN (0.3 mmol) and **1b** (15 mg, 0.10 mmol) were added into the mixture. At last, the mixture was stirred at a distance of ~1 cm from a 2 x 3 W purple LEDs at room temperature for 24 h until the reaction was completed, as monitored by TLC analysis. The reaction mixture was quenched with water(10 mL), diluted with EtOAc (3 x 10 mL), washed with NaCl (aq) and dried over with anhydrous Na_2SO_4. After filtration and concentration, the residue was purified by silica gel chromatography with petroleum ether and ethyl acetate (PE/EA = 5:1) to afford **12** in 76% yield. ^{1}H NMR (400 MHz, $CDCl_3$) δ (ppm) 8.08 (d, J = 8.1 Hz, 2H), 7.63 – 7.53 (m, 1H), 7.46 (t, J = 7.2 Hz, 2H), 1.86 – 1.65 (m, 3H), 1.59 (d, J = 12.5 Hz, 2H), 1.46 (d, J = 12.5 Hz, 1H), 1.28 (s, 6H), 1.13 (s, 6H). ^{13}C NMR (100 MHz, $CDCl_3$) δ (ppm) 166.3, 132.8, 129.6, 129.5, 128.4, 77.3, 77.0, 76.7, 60.3, 39.0, 31.9, 20.8, 16.9. HRMS (EI): m/z [M + Na]$^+$ calcd for $C_{16}H_{23}NNaO_2$: 284.1621, found: 284.1625.

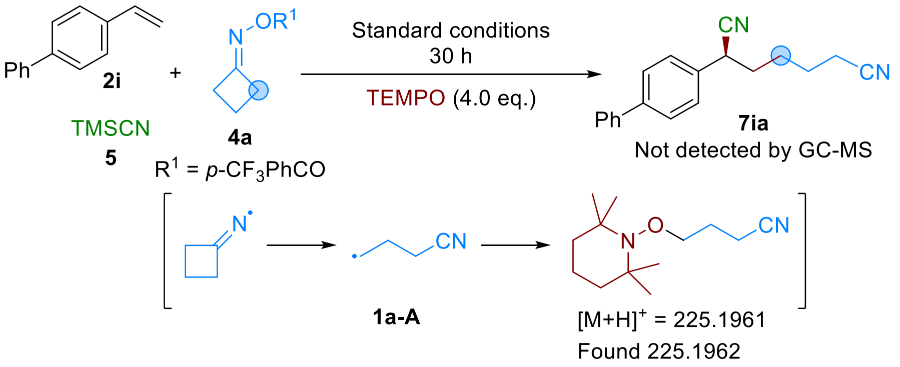

In a flame-dried 10 mL Schlenk tube equipped with a magnetic stirrer bar was charged sequentially with $Cu(CH_3CN)_4PF_6$ (0.001 mmol), ligand-**1** (0.0012 mmol) and organo-photocatalyst Ph-PTZ (0.0025 mmol) followed by the addition of DMA (4.0 mL). Then the mixture was stirred at room temperature for 30 min. To the resulting mixture were added **2i** (0.20 mmol), **4a** (0.6 mmol) and TEMPO (0.80 mmol). Then, the resulting mixture was degassed (3 times) under argon atmosphere. After that, TMSCN (0.6 mmol) was added into the mixture. At last, the mixture was stirred at a distance of ~1 cm from a 2 x 3 W purple LEDs at room temperature about 30 h. The reaction mixture was detected by HRMS. HRMS (EI): m/z $[M + H]^+$ calcd for $C_{13}H_{24}N_2O$: 225.1961, found:225.1962.

6.7 Radical Clock Experiments

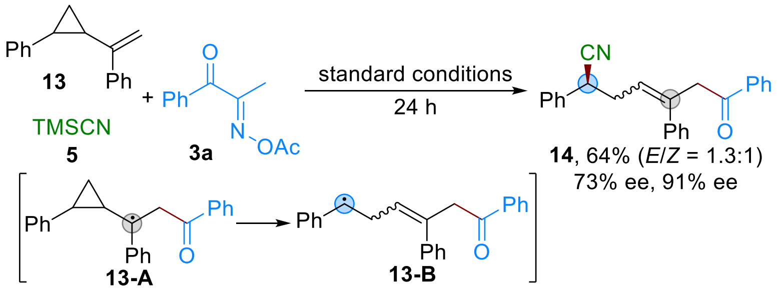

In a flame-dried 10 mL Schlenk tube equipped with a magnetic stirrer bar was charged sequentially with $Cu(CH_3CN)_4PF_6$ (0.56 mg, 0.0015 mmol) and ligand-**1** (0.80 mg, 0.00225 mmol), followed by the addition of DMA (2.5 mL). Then the mixture was stirred at room temperature for 30 min. To the resulting mixture were added **3a** (62 mg, 0.30 mmol), **13** (22 mg, 0.10 mmol), *fac*-Ir(ppy)$_3$ (0.53 mg, 0.0008 mmol). Then, the resulting mixture was degassed (3 times) under argon atmosphere. After that, TMSCN (0.3 mmol) was added into the mixture. At last, the mixture was stirred at a distance of ~1 cm from a 2 x 3 W purple LEDs at room temperature for 24 h until the reaction was completed, as monitored by TLC analysis. The reaction mixture was quenched with water(10 mL), diluted with EtOAc (3 x 10 mL), washed with NaCl (aq) and dried over with anhydrous Na_2SO_4. After filtration and concentration, the residue was purified by silica gel chromatography with petroleum ether and ethyl acetate (PE/EA = 5:1) to afford **14** in 64% yield (1.3:1 *E*/*Z*). 73% ee, 91% ee, determined by HPLC analysis (Chiralpak AZ column, hexane/*i*-PrOH, 90:10 v/v, flow rate

1.0 mL/min, λ = 254 nm, 25 °C), t_R (major) = 28.23 min, t_R (minor) = 31.19 min; t_R (major) = 42.44 min, t_R (minor) = 69.99 min. ^{1}H NMR (400 MHz, $CDCl_3$) δ (ppm) 7.91 (dd, *J* = 17.8, 7.6 Hz, 4H), 7.56 (dt, *J* = 13.0, 7.4 Hz, 2H), 7.49 – 7.33 (m, 10H), 7.30 – 7.21 (m, 11H), 7.16 (d, *J* = 3.6 Hz, 2H), 7.02 (d, *J* = 7.9 Hz, 2H), 6.02 (t, *J* = 7.5 Hz, 1H), 5.63 (t, *J* = 7.2 Hz, 1H), 4.08 (d, *J* = 7.3 Hz, 2H), 4.04 – 3.96 (m, 3H), 3.76 (t, *J* = 7.1 Hz, 1H), 2.82 – 2.68 (m, 2H), 2.59 (t, *J* = 7.3 Hz, 2H). ^{13}C NMR (100 MHz, $CDCl_3$) δ (ppm) 197.3, 196.3, 142.2, 139.4, 139.3, 137.3, 136.6, 136.5, 135.2, 135.0, 133.4, 133.2, 129.1, 129.0, 128.7, 128.6, 128.5, 128.4, 128.4, 128.3, 128.2, 128.1, 127.4, 127.4, 126.4, 126.2, 125.8, 120.6, 120.4, 77.4, 77.0, 76.7, 48.7, 40.8, 37.5, 37.4, 35.5, 35.1.

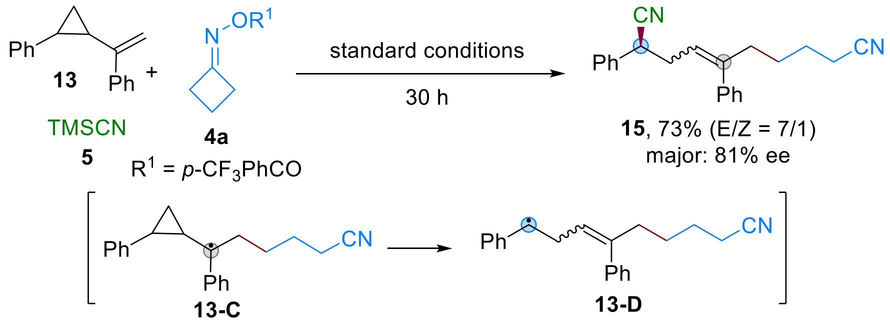

To gain more insight into the possible radical reaction mechanism, the reaction of radical clock substrate **13** bearing a cyclopropyl moiety was carried out under the sandard conditions, giving the ring-opening product **15** in 73% yield, 81% ee value (major) with 7:1 E/Z ratio.These observations indicated the radical property of the cross-coupling process and the involvement of cyanoalkyl radical and benzylic radical such as **13-C**. $[\alpha]_D^{25}$ = -7.42 (c = 0.51 in $CHCl_3$); 81% ee, determined by HPLC analysis (Chiralpak AD column, hexane/*i*-PrOH, 80:20 v/v, flow rate 1.0 mL/min, λ =254 nm, 25 °C), t_R (major) = 9.16 min, t_R (minor) = 8.54 min; ^{1}H NMR (400 MHz, $CDCl_3$) δ (ppm) 7.45 – 7.34 (m, 5H), 7.33 – 7.23 (m, 5H), 5.64 (t, *J* = 7.5 Hz, 1H), 3.93 (t, *J* = 7.0 Hz, 1H), 2.86 – 7.74 (m, 2H), 2.41 (t, *J* = 7.7 Hz, 2H), 2.19 (t, *J* = 7.1 Hz, 2H), 1.54 – 1.46 (m, 2H), 1.36 – 1.25 (m, 2H). ^{13}C NMR (100 MHz, $CDCl_3$) δ (ppm) 143.8, 141.9, 135.2, 129.1, 128.4, 128.2, 127.4, 126.4, 122.8, 120.4, 119.4, 37.6, 34.8, 29.1, 27.3, 25.0, 16.9 . HRMS (EI): m/z $[M + H]^+$ calcd for $C_{22}H_{22}N_2Na$: 337.1675, found: 337.1666.

7. Determination of the Absolute Configuration of Products 6bj and 7ja

Single crystals of $C_{16}H_{12}ClNO$ [**6bj**]. A suitable crystal was selected and [**6bj**] on a '**Bruker APEX-II CCD**' diffractometer. The crystal was kept at 296(2) K during data collection.

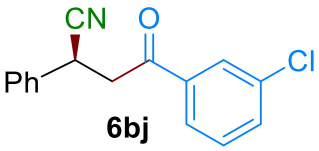

6bj

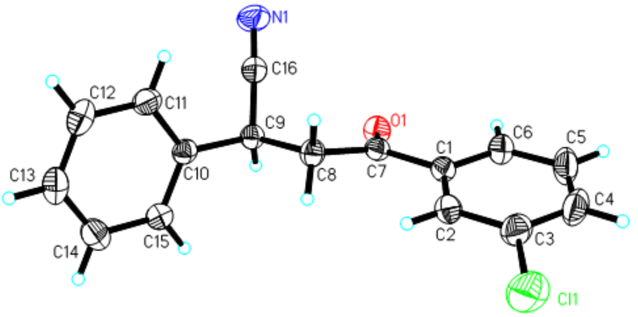

Figure S6. X-ray crystallography of **6bj**

Crystal Data for $C_{16}H_{12}ClNO$ (M = 269.72 g/mol): monoclinic, space group P2$_1$ (no. 4), a = 5.0263(13) Å, b = 10.678(3) Å, c = 12.754(3) Å, β = 95.121(7)°, V = 681.8(3) Å^3, Z = 2, T = 296(2) K, μ(MoKα) = 0.270 mm^{-1}, $Dcalc$ = 1.314 g/cm^3, 4107 reflections measured (3.206° ≤ 2Θ ≤ 52.306°), 2572 unique (R_{int} = 0.0344, R_{sigma} = 0.0523) which were used in all calculations. The final R_1 was 0.0473 (I > 2σ(I)) and wR_2 was 0.1309 (all data).

Table 1. Crystal data and structure refinement for **6bj**.

Identification code	mo_201007f_0m	
Empirical formula	C16 H12 Cl N O	
Formula weight	269.72	
Temperature	296(2) K	
Wavelength	0.71073 Å	
Crystal system	Monoclinic	
Space group	P 1 21 1	
Unit cell dimensions	a = 5.0263(13) Å	α = 90°.
	b = 10.678(3) Å	β = 95.121(7)°.
	c = 12.754(3) Å	γ = 90°.
Volume	681.8(3) Å^3	
Z	2	
Density (calculated)	1.314 Mg/m^3	
Absorption coefficient	0.270 mm^{-1}	
F(000)	280	
Crystal size	0.3 x 0.2 x 0.1 mm^3	
Theta range for data collection	1.603 to 26.153°.	
Index ranges	-6<=h<=5, -12<=k<=13, -15<=l<=15	
Reflections collected	4107	
Independent reflections	2572 [R(int) = 0.0344]	
Completeness to theta = 25.242°	99.7 %	
Absorption correction	Semi-empirical from equivalents	
Max. and min. transmission	0.7454 and 0.5994	
Refinement method	Full-matrix least-squares on F^2	
Data / restraints / parameters	2572 / 1 / 172	
Goodness-of-fit on F^2	1.069	
Final R indices [I>2sigma(I)]	R1 = 0.0473, wR2 = 0.1181	
R indices (all data)	R1 = 0.0669, wR2 = 0.1309	

Absolute structure parameter	0.02(6)
Extinction coefficient	n/a
Largest diff. peak and hole	0.243 and -0.179 e.Å^{-3}

Table 2. Atomic coordinates (x 10^4) and equivalent isotropic displacement parameters (Å^{2}x 10^3) for mo_201007F_0m. U(eq) is defined as one third of the trace of the orthogonalized U^{ij} tensor.

	x	y	z	U(eq)
C(1)	1662(8)	3892(4)	7308(3)	46(1)
C(2)	3603(9)	3013(4)	7133(3)	52(1)
C(3)	3990(10)	2685(5)	6108(4)	65(1)
C(4)	2567(11)	3236(6)	5274(4)	74(2)
C(5)	680(11)	4133(6)	5448(4)	78(2)
C(6)	198(9)	4444(4)	6459(3)	58(1)
C(7)	1081(7)	4272(4)	8394(3)	42(1)
C(8)	3076(7)	3968(4)	9308(3)	46(1)
C(9)	2120(7)	4312(3)	10375(3)	41(1)
C(10)	3991(7)	3808(4)	11277(3)	41(1)
C(11)	6075(8)	4510(4)	11745(3)	52(1)
C(12)	7776(9)	4008(5)	12546(4)	67(1)
C(13)	7408(9)	2800(5)	12869(3)	64(1)
C(14)	5354(10)	2101(5)	12413(4)	62(1)
C(15)	3650(9)	2599(4)	11611(3)	51(1)
C(16)	1730(9)	5664(4)	10482(3)	51(1)
Cl(1)	6261(3)	1516(2)	5879(1)	101(1)
N(1)	1500(9)	6719(4)	10600(4)	75(1)
O(1)	-958(6)	4827(3)	8523(2)	54(1)

Table 3. Bond lengths [Å] and angles [°] for mo_201007F_0m.

C(1)-C(2)	1.387(6)
C(1)-C(6)	1.385(6)
C(1)-C(7)	1.497(5)
C(2)-H(2)	0.9300
C(2)-C(3)	1.383(6)
C(3)-C(4)	1.362(7)
C(3)-Cl(1)	1.734(5)
C(4)-H(4)	0.9300
C(4)-C(5)	1.380(8)
C(5)-H(5)	0.9300
C(5)-C(6)	1.374(7)
C(6)-H(6)	0.9300

C(7)-C(8)	1.504(5)
C(7)-O(1)	1.207(4)
C(8)-H(8A)	0.9700
C(8)-H(8B)	0.9700
C(8)-C(9)	1.527(5)
C(9)-H(9)	0.9800
C(9)-C(10)	1.518(5)
C(9)-C(16)	1.464(6)
C(10)-C(11)	1.380(6)
C(10)-C(15)	1.375(6)
C(11)-H(11)	0.9300
C(11)-C(12)	1.381(6)
C(12)-H(12)	0.9300
C(12)-C(13)	1.372(7)
C(13)-H(13)	0.9300
C(13)-C(14)	1.361(7)
C(14)-H(14)	0.9300
C(14)-C(15)	1.382(6)
C(15)-H(15)	0.9300
C(16)-N(1)	1.144(6)
C(2)-C(1)-C(7)	122.1(3)
C(6)-C(1)-C(2)	119.6(4)
C(6)-C(1)-C(7)	118.3(4)
C(1)-C(2)-H(2)	120.5
C(3)-C(2)-C(1)	119.0(4)
C(3)-C(2)-H(2)	120.5
C(2)-C(3)-Cl(1)	119.4(4)
C(4)-C(3)-C(2)	121.4(5)
C(4)-C(3)-Cl(1)	119.2(4)
C(3)-C(4)-H(4)	120.2
C(3)-C(4)-C(5)	119.6(4)
C(5)-C(4)-H(4)	120.2
C(4)-C(5)-H(5)	120.0
C(6)-C(5)-C(4)	120.0(5)
C(6)-C(5)-H(5)	120.0
C(1)-C(6)-H(6)	119.8
C(5)-C(6)-C(1)	120.3(5)
C(5)-C(6)-H(6)	119.8
C(1)-C(7)-C(8)	119.1(3)
O(1)-C(7)-C(1)	119.8(3)
O(1)-C(7)-C(8)	121.1(3)
C(7)-C(8)-H(8A)	108.9
C(7)-C(8)-H(8B)	108.9
C(7)-C(8)-C(9)	113.4(3)

H(8A)-C(8)-H(8B)	107.7
C(9)-C(8)-H(8A)	108.9
C(9)-C(8)-H(8B)	108.9
C(8)-C(9)-H(9)	107.3
C(10)-C(9)-C(8)	111.5(3)
C(10)-C(9)-H(9)	107.3
C(16)-C(9)-C(8)	112.1(3)
C(16)-C(9)-H(9)	107.3
C(16)-C(9)-C(10)	111.0(3)
C(11)-C(10)-C(9)	122.0(4)
C(15)-C(10)-C(9)	118.8(3)
C(15)-C(10)-C(11)	119.2(4)
C(10)-C(11)-H(11)	119.8
C(10)-C(11)-C(12)	120.3(4)
C(12)-C(11)-H(11)	119.8
C(11)-C(12)-H(12)	120.1
C(13)-C(12)-C(11)	119.7(4)
C(13)-C(12)-H(12)	120.1
C(12)-C(13)-H(13)	119.8
C(14)-C(13)-C(12)	120.3(4)
C(14)-C(13)-H(13)	119.8
C(13)-C(14)-H(14)	119.9
C(13)-C(14)-C(15)	120.1(4)
C(15)-C(14)-H(14)	119.9
C(10)-C(15)-C(14)	120.3(4)
C(10)-C(15)-H(15)	119.9
C(14)-C(15)-H(15)	119.9
N(1)-C(16)-C(9)	177.2(5)

Symmetry transformations used to generate equivalent atoms:

Table 4. Anisotropic displacement parameters ($Å^2$x 10^3) for mo_201007F_0m. The anisotropic displacement factor exponent takes the form: $-2p^2[h^2 a*^2U^{11} + ... + 2 h k a* b* U^{12}]$

	U^{11}	U^{22}	U^{33}	U^{23}	U^{13}	U^{12}
C(1)	51(2)	44(2)	41(2)	3(2)	3(2)	-7(2)
C(2)	60(2)	52(2)	45(2)	1(2)	11(2)	-3(2)
C(3)	74(3)	63(3)	59(3)	-8(2)	20(2)	-12(2)
C(4)	90(4)	93(4)	43(3)	-16(3)	17(3)	-21(3)
C(5)	93(4)	98(4)	39(3)	5(3)	-5(2)	-10(4)
C(6)	64(3)	63(3)	46(3)	1(2)	-2(2)	-1(2)
C(7)	43(2)	38(2)	44(2)	2(2)	5(2)	-2(2)
C(8)	47(2)	51(2)	41(2)	-2(2)	6(2)	2(2)
C(9)	41(2)	43(2)	40(2)	-1(2)	6(2)	1(2)

C(10)	44(2)	42(2)	38(2)	-6(2)	9(2)	2(2)
C(11)	57(2)	48(2)	52(2)	-6(2)	6(2)	-7(2)
C(12)	62(3)	85(4)	51(3)	-11(3)	-8(2)	-6(3)
C(13)	64(3)	83(4)	44(2)	1(3)	-4(2)	12(3)
C(14)	70(3)	60(3)	56(3)	11(2)	8(2)	8(2)
C(15)	57(2)	46(3)	49(2)	-2(2)	1(2)	-1(2)
C(16)	57(3)	51(3)	46(2)	0(2)	1(2)	1(2)
Cl(1)	125(1)	88(1)	97(1)	-17(1)	54(1)	14(1)
N(1)	96(3)	42(2)	84(3)	-9(2)	-7(2)	11(2)
O(1)	50(2)	58(2)	54(2)	0(1)	3(1)	9(1)

Table 5. Hydrogen coordinates (x 10^4) and isotropic displacement parameters (Å^{2}x 10^3) for mo_201007F_0m.

	x	y	z	U(eq)
H(2)	4628	2649	7695	62
H(4)	2865	3009	4590	89
H(5)	-266	4527	4881	93
H(6)	-1117	5028	6574	70
H(8A)	4725	4413	9222	55
H(8B)	3461	3078	9301	55
H(9)	380	3911	10418	49
H(11)	6334	5326	11519	63
H(12)	9167	4488	12865	80
H(13)	8566	2457	13402	77
H(14)	5099	1286	12643	75
H(15)	2264	2114	11295	61

Table 6. Torsion angles [°] for mo_201007F_0m.

C(1)-C(2)-C(3)-C(4)	2.1(7)
C(1)-C(2)-C(3)-Cl(1)	-175.9(3)
C(1)-C(7)-C(8)-C(9)	-175.2(3)
C(2)-C(1)-C(6)-C(5)	-0.6(7)
C(2)-C(1)-C(7)-C(8)	16.4(6)
C(2)-C(1)-C(7)-O(1)	-164.4(4)
C(2)-C(3)-C(4)-C(5)	-0.5(8)
C(3)-C(4)-C(5)-C(6)	-1.6(8)
C(4)-C(5)-C(6)-C(1)	2.2(8)
C(6)-C(1)-C(2)-C(3)	-1.5(6)
C(6)-C(1)-C(7)-C(8)	-163.6(4)
C(6)-C(1)-C(7)-O(1)	15.6(6)

C(7)-C(1)-C(2)-C(3)	178.5(4)
C(7)-C(1)-C(6)-C(5)	179.4(4)
C(7)-C(8)-C(9)-C(10)	171.1(3)
C(7)-C(8)-C(9)-C(16)	-63.8(4)
C(8)-C(9)-C(10)-C(11)	92.5(4)
C(8)-C(9)-C(10)-C(15)	-85.2(4)
C(9)-C(10)-C(11)-C(12)	-178.4(4)
C(9)-C(10)-C(15)-C(14)	178.5(4)
C(10)-C(11)-C(12)-C(13)	0.7(7)
C(11)-C(10)-C(15)-C(14)	0.8(6)
C(11)-C(12)-C(13)-C(14)	-0.8(7)
C(12)-C(13)-C(14)-C(15)	0.9(7)
C(13)-C(14)-C(15)-C(10)	-0.9(7)
C(15)-C(10)-C(11)-C(12)	-0.7(6)
C(16)-C(9)-C(10)-C(11)	-33.2(5)
C(16)-C(9)-C(10)-C(15)	149.1(4)
Cl(1)-C(3)-C(4)-C(5)	177.5(4)
O(1)-C(7)-C(8)-C(9)	5.6(5)

Symmetry transformations used to generate equivalent atoms:

Table 7. Hydrogen bonds for mo_201007F_0m [Å and °].

D-H...A	d(D-H)	d(H...A)	d(D...A)	<(DHA)

Single crystals of $C_{19}H_{17}ClN_2$ [**7ja**]. A suitable crystal was selected and [**7ja**] on a '**Bruker APEX-II CCD**' diffractometer. The crystal was kept at 296.15 K during data collection.

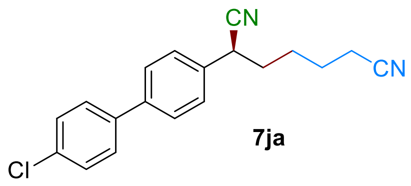

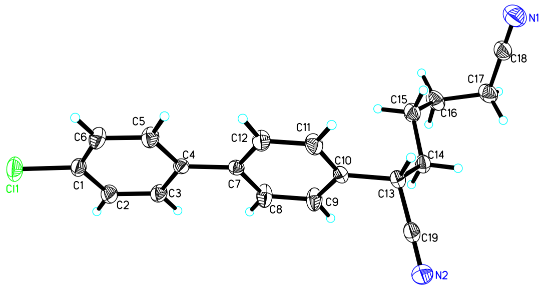

Figure S6. X-ray crystallography of **7ja**

Crystal Data of **7ja**: $C_{19}H_{17}ClN_2$ (M = 308.79 g/mol): orthorhombic, space group $P2_12_12_1$ (no. 19), a = 5.6856(9) Å, b = 7.6678(13) Å, c = 36.821(6) Å, V = 1605.3(5) Å^3, Z = 4, T = 296.15 K, μ(MoKα) = 0.236 mm^{-1}, $Dcalc$ = 1.278 g/cm^3, 15471 reflections measured (2.212° ≤ 2Θ ≤ 59.104°), 4469 unique (R_{int} = 0.0329, R_{sigma} = 0.0352) which were used in all calculations. The final R_1 was 0.0489 (I > 2σ(I)) and wR_2 was 0.1477 (all data).

Table 1. Crystal data and structure refinement for mo_200717d_0m **7ja**.

Identification code	mo_200717d_0m	
Empirical formula	C19 H17 Cl N2	
Formula weight	308.79	
Temperature	296.15 K	
Wavelength	0.71073 Å	
Crystal system	Orthorhombic	
Space group	$P2_12_12_1$	
Unit cell dimensions	a = 5.6856(9) Å	α= 90°.
	b = 7.6678(13) Å	β= 90°.
	c = 36.821(6) Å	γ= 90°.
Volume	1605.3(5) Å^3	
Z	4	
Density (calculated)	1.278 Mg/m^3	
Absorption coefficient	0.236 mm^{-1}	
F(000)	648	
Crystal size	0.15 x 0.12 x 0.1 mm^3	
Theta range for data collection	1.106 to 29.552°.	
Index ranges	-7<=h<=7, -9<=k<=10, -51<=l<=50	
Reflections collected	15471	
Independent reflections	4469 [R(int) = 0.0329]	
Completeness to theta = 25.242°	99.7 %	

Absorption correction	Semi-empirical from equivalents
Max. and min. transmission	0.7459 and 0.6593
Refinement method	Full-matrix least-squares on F^2
Data / restraints / parameters	4469 / 0 / 199
Goodness-of-fit on F^2	1.046
Final R indices [I>2sigma(I)]	R1 = 0.0489, wR2 = 0.1403
R indices (all data)	R1 = 0.0591, wR2 = 0.1477
Absolute structure parameter	0.06(3)
Extinction coefficient	n/a
Largest diff. peak and hole	0.211 and -0.281 e.Å^{-3}

Table 2. Atomic coordinates (x 10^4) and equivalent isotropic displacement parameters (Å^{2}x 10^3) for mo_200717d_0m. U(eq) is defined as one third of the trace of the orthogonalized U^{ij} tensor.

	x	y	z	U(eq)
C(1)	2511(5)	4312(3)	5650(1)	40(1)
C(2)	595(5)	5251(4)	5525(1)	46(1)
C(3)	450(5)	5629(4)	5156(1)	42(1)
C(4)	2171(4)	5100(3)	4909(1)	31(1)
C(5)	4090(5)	4192(4)	5048(1)	44(1)
C(6)	4251(6)	3800(4)	5419(1)	47(1)
C(7)	1939(4)	5435(3)	4511(1)	30(1)
C(8)	4(6)	6291(5)	4368(1)	50(1)
C(9)	-275(6)	6493(5)	3996(1)	51(1)
C(10)	1376(4)	5870(3)	3753(1)	33(1)
C(11)	3323(5)	5035(5)	3894(1)	49(1)
C(12)	3606(5)	4828(5)	4264(1)	50(1)
C(13)	1064(5)	6042(3)	3343(1)	36(1)
C(14)	842(5)	7945(4)	3210(1)	40(1)
C(15)	2987(6)	9038(4)	3303(1)	49(1)
C(16)	2778(7)	10917(4)	3168(1)	54(1)
C(17)	3027(6)	11134(5)	2760(1)	54(1)
C(18)	5417(6)	10767(5)	2635(1)	51(1)
C(19)	-1049(6)	5067(4)	3232(1)	43(1)
Cl(1)	2694(2)	3762(1)	6107(1)	62(1)
N(1)	7264(7)	10474(5)	2539(1)	74(1)
N(2)	-2722(6)	4355(4)	3151(1)	63(1)

Table 3. Bond lengths [Å] and angles [°] for mo_200717d_0m.

C(1)-C(2)	1.385(4)
C(1)-C(6)	1.364(4)
C(1)-Cl(1)	1.738(2)
C(2)-H(2)	0.9300
C(2)-C(3)	1.391(4)
C(3)-H(3)	0.9300
C(3)-C(4)	1.396(3)
C(4)-C(5)	1.392(4)
C(4)-C(7)	1.495(3)
C(5)-H(5)	0.9300
C(5)-C(6)	1.399(4)
C(6)-H(6)	0.9300
C(7)-C(8)	1.384(4)
C(7)-C(12)	1.392(3)
C(8)-H(8)	0.9300
C(8)-C(9)	1.388(4)
C(9)-H(9)	0.9300
C(9)-C(10)	1.382(4)
C(10)-C(11)	1.380(4)
C(10)-C(13)	1.525(3)
C(11)-H(11)	0.9300
C(11)-C(12)	1.383(4)
C(12)-H(12)	0.9300
C(13)-H(13)	0.9800
C(13)-C(14)	1.545(4)
C(13)-C(19)	1.473(4)
C(14)-H(14A)	0.9700
C(14)-H(14B)	0.9700
C(14)-C(15)	1.519(4)
C(15)-H(15A)	0.9700
C(15)-H(15B)	0.9700
C(15)-C(16)	1.529(4)
C(16)-H(16A)	0.9700
C(16)-H(16B)	0.9700
C(16)-C(17)	1.519(4)

C(17)-H(17A)	0.9700
C(17)-H(17B)	0.9700
C(17)-C(18)	1.462(5)
C(18)-N(1)	1.131(5)
C(19)-N(2)	1.136(4)

C(2)-C(1)-Cl(1)	119.8(2)
C(6)-C(1)-C(2)	120.7(2)
C(6)-C(1)-Cl(1)	119.5(2)
C(1)-C(2)-H(2)	120.6
C(1)-C(2)-C(3)	118.8(3)
C(3)-C(2)-H(2)	120.6
C(2)-C(3)-H(3)	118.9
C(2)-C(3)-C(4)	122.2(3)
C(4)-C(3)-H(3)	118.9
C(3)-C(4)-C(7)	121.8(2)
C(5)-C(4)-C(3)	117.0(2)
C(5)-C(4)-C(7)	121.1(2)
C(4)-C(5)-H(5)	119.4
C(4)-C(5)-C(6)	121.2(3)
C(6)-C(5)-H(5)	119.4
C(1)-C(6)-C(5)	120.0(3)
C(1)-C(6)-H(6)	120.0
C(5)-C(6)-H(6)	120.0
C(8)-C(7)-C(4)	121.6(2)
C(8)-C(7)-C(12)	116.9(2)
C(12)-C(7)-C(4)	121.5(2)
C(7)-C(8)-H(8)	119.4
C(7)-C(8)-C(9)	121.2(2)
C(9)-C(8)-H(8)	119.4
C(8)-C(9)-H(9)	119.2
C(10)-C(9)-C(8)	121.5(3)
C(10)-C(9)-H(9)	119.2
C(9)-C(10)-C(13)	122.1(2)
C(11)-C(10)-C(9)	117.5(2)
C(11)-C(10)-C(13)	120.3(2)
C(10)-C(11)-H(11)	119.4
C(10)-C(11)-C(12)	121.1(2)

C(12)-C(11)-H(11)	119.4
C(7)-C(12)-H(12)	119.2
C(11)-C(12)-C(7)	121.7(2)
C(11)-C(12)-H(12)	119.2
C(10)-C(13)-H(13)	108.3
C(10)-C(13)-C(14)	113.9(2)
C(14)-C(13)-H(13)	108.3
C(19)-C(13)-C(10)	109.0(2)
C(19)-C(13)-H(13)	108.3
C(19)-C(13)-C(14)	109.0(2)
C(13)-C(14)-H(14A)	109.1
C(13)-C(14)-H(14B)	109.1
H(14A)-C(14)-H(14B)	107.8
C(15)-C(14)-C(13)	112.6(2)
C(15)-C(14)-H(14A)	109.1
C(15)-C(14)-H(14B)	109.1
C(14)-C(15)-H(15A)	109.1
C(14)-C(15)-H(15B)	109.1
C(14)-C(15)-C(16)	112.6(3)
H(15A)-C(15)-H(15B)	107.8
C(16)-C(15)-H(15A)	109.1
C(16)-C(15)-H(15B)	109.1
C(15)-C(16)-H(16A)	108.6
C(15)-C(16)-H(16B)	108.6
H(16A)-C(16)-H(16B)	107.6
C(17)-C(16)-C(15)	114.7(3)
C(17)-C(16)-H(16A)	108.6
C(17)-C(16)-H(16B)	108.6
C(16)-C(17)-H(17A)	109.2
C(16)-C(17)-H(17B)	109.2
H(17A)-C(17)-H(17B)	107.9
C(18)-C(17)-C(16)	112.2(3)
C(18)-C(17)-H(17A)	109.2
C(18)-C(17)-H(17B)	109.2
N(1)-C(18)-C(17)	179.6(4)
N(2)-C(19)-C(13)	177.8(3)

Symmetry transformations used to generate equivalent atoms:

Table 4. Anisotropic displacement parameters ($Å^2$x 10^3) for mo_200717d_0m. The anisotropic displacement factor exponent takes the form: $-2\pi^2[h^2 a^{*2}U^{11} + ... + 2 h k a^* b^* U^{12}]$

	U^{11}	U^{22}	U^{33}	U^{23}	U^{13}	U^{12}
C(1)	49(2)	36(1)	33(1)	3(1)	-8(1)	-7(1)
C(2)	42(1)	60(2)	36(1)	3(1)	3(1)	3(1)
C(3)	34(1)	54(2)	38(1)	2(1)	-1(1)	8(1)
C(4)	31(1)	28(1)	34(1)	2(1)	-1(1)	-2(1)
C(5)	44(1)	50(2)	39(1)	3(1)	1(1)	14(1)
C(6)	51(2)	49(2)	40(1)	4(1)	-8(1)	12(1)
C(7)	30(1)	27(1)	33(1)	2(1)	1(1)	-1(1)
C(8)	52(2)	65(2)	32(1)	2(1)	6(1)	29(2)
C(9)	51(2)	66(2)	35(1)	7(1)	2(1)	27(2)
C(10)	38(1)	31(1)	29(1)	2(1)	5(1)	-2(1)
C(11)	41(1)	71(2)	36(1)	4(1)	9(1)	16(1)
C(12)	36(1)	73(2)	40(1)	9(1)	4(1)	20(1)
C(13)	40(1)	39(1)	29(1)	2(1)	4(1)	0(1)
C(14)	48(2)	39(1)	33(1)	4(1)	0(1)	-2(1)
C(15)	60(2)	48(2)	39(1)	10(1)	-2(1)	-16(1)
C(16)	71(2)	42(1)	51(2)	1(1)	15(2)	-14(2)
C(17)	54(2)	53(2)	55(2)	22(1)	5(1)	-2(2)
C(18)	58(2)	52(2)	44(1)	3(1)	4(1)	-11(1)
C(19)	57(2)	42(1)	32(1)	1(1)	7(1)	-4(1)
Cl(1)	83(1)	69(1)	34(1)	9(1)	-9(1)	-4(1)
N(1)	65(2)	86(2)	71(2)	-5(2)	16(2)	-4(2)
N(2)	68(2)	70(2)	52(1)	-4(1)	-1(1)	-27(2)

Table 5. Hydrogen coordinates (x 10^4) and isotropic displacement parameters ($Å^2$x 10 ^{3}) for mo_200717d_0m.

	x	y	z	U(eq)
H(2)	-572	5621	5684	55
H(3)	-838	6256	5071	50
H(5)	5286	3839	4893	53
H(6)	5544	3190	5507	56
H(8)	-1129	6739	4525	60

H(9)	-1603	7061	3908	61
H(11)	4465	4604	3737	59
H(12)	4945	4270	4351	60
H(13)	2432	5514	3224	43
H(14A)	623	7947	2949	48
H(14B)	-539	8471	3319	48
H(15A)	4370	8506	3196	59
H(15B)	3199	9044	3565	59
H(16A)	1259	11373	3242	65
H(16B)	3976	11615	3287	65
H(17A)	1940	10352	2639	65
H(17B)	2610	12319	2694	65

Table 6. Torsion angles [°] for mo_200717d_0m.

C(1)-C(2)-C(3)-C(4)	-0.2(5)
C(2)-C(1)-C(6)-C(5)	-1.3(5)
C(2)-C(3)-C(4)-C(5)	-1.1(4)
C(2)-C(3)-C(4)-C(7)	176.9(3)
C(3)-C(4)-C(5)-C(6)	1.3(4)
C(3)-C(4)-C(7)-C(8)	0.2(4)
C(3)-C(4)-C(7)-C(12)	-176.8(3)
C(4)-C(5)-C(6)-C(1)	-0.1(5)
C(4)-C(7)-C(8)-C(9)	-175.6(3)
C(4)-C(7)-C(12)-C(11)	175.7(3)
C(5)-C(4)-C(7)-C(8)	178.0(3)
C(5)-C(4)-C(7)-C(12)	1.1(4)
C(6)-C(1)-C(2)-C(3)	1.4(4)
C(7)-C(4)-C(5)-C(6)	-176.7(3)
C(7)-C(8)-C(9)-C(10)	-0.8(5)
C(8)-C(7)-C(12)-C(11)	-1.4(5)
C(8)-C(9)-C(10)-C(11)	0.0(5)
C(8)-C(9)-C(10)-C(13)	178.5(3)
C(9)-C(10)-C(11)-C(12)	0.1(5)
C(9)-C(10)-C(13)-C(14)	59.6(4)
C(9)-C(10)-C(13)-C(19)	-62.4(3)
C(10)-C(11)-C(12)-C(7)	0.6(5)

C(10)-C(13)-C(14)-C(15)	59.8(3)
C(11)-C(10)-C(13)-C(14)	-122.0(3)
C(11)-C(10)-C(13)-C(19)	116.1(3)
C(12)-C(7)-C(8)-C(9)	1.5(5)
C(13)-C(10)-C(11)-C(12)	-178.4(3)
C(13)-C(14)-C(15)-C(16)	179.5(2)
C(14)-C(15)-C(16)-C(17)	-72.8(4)
C(15)-C(16)-C(17)-C(18)	-68.5(4)
C(19)-C(13)-C(14)-C(15)	-178.3(2)
Cl(1)-C(1)-C(2)-C(3)	-178.0(2)
Cl(1)-C(1)-C(6)-C(5)	178.2(2)

Symmetry transformations used to generate equivalent atoms:

8. The Spectra of Substrates and Products

^{1}H NMR (400 MHz, $CDCl_3$) and ^{13}C NMR (100 MHz, $CDCl_3$) spectra of substrate 6aa

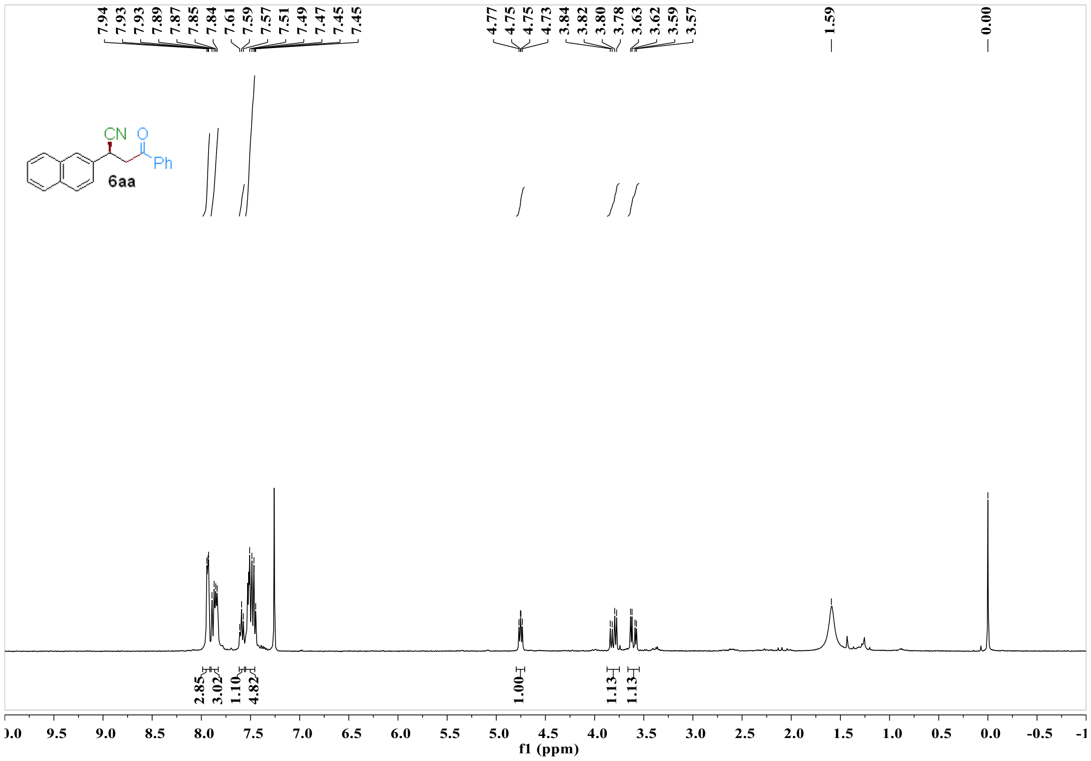

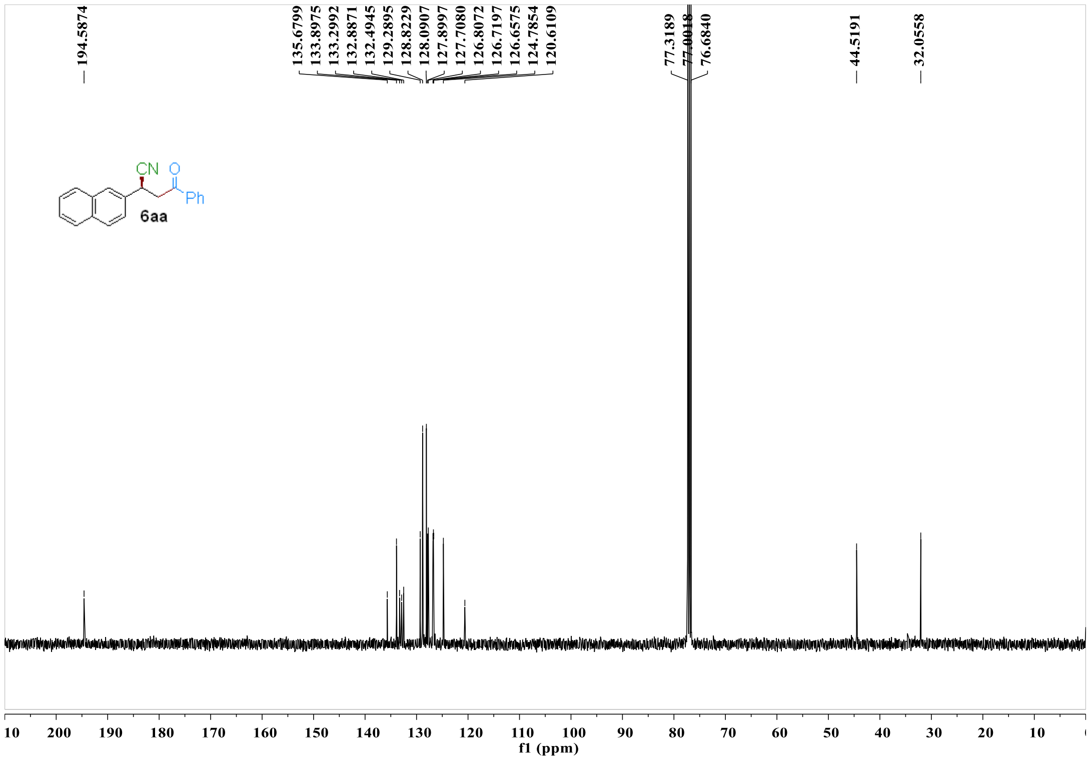

^{1}H NMR (400 MHz, $CDCl_3$) and ^{13}C NMR (100 MHz, $CDCl_3$) spectra of substrate 6ba

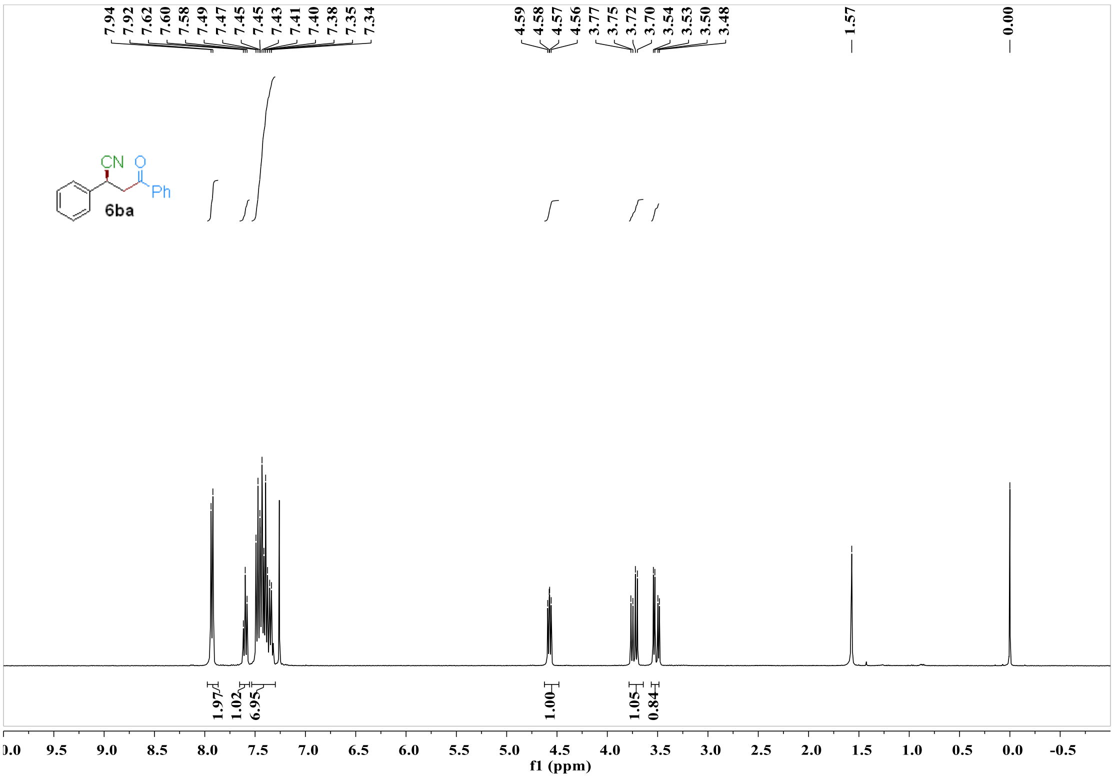

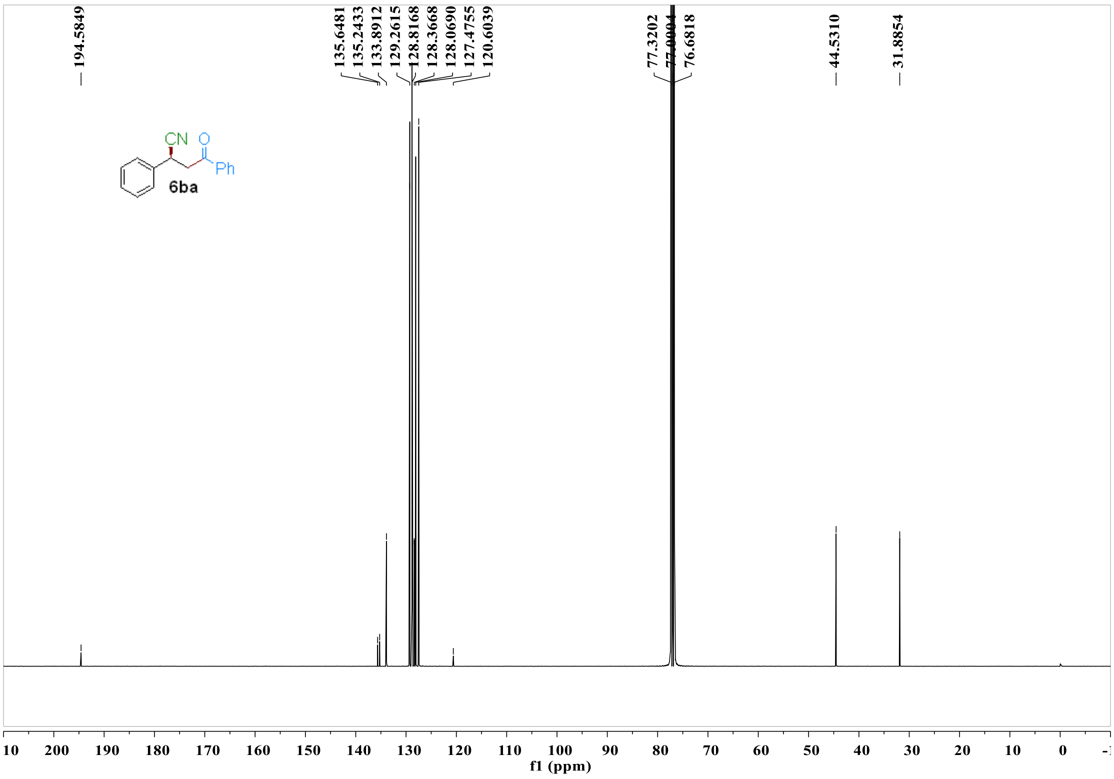

^{1}H NMR (400 MHz, CDCl3) and ^{13}C NMR (100 MHz, CDCl3) spectra of substrate 6ca

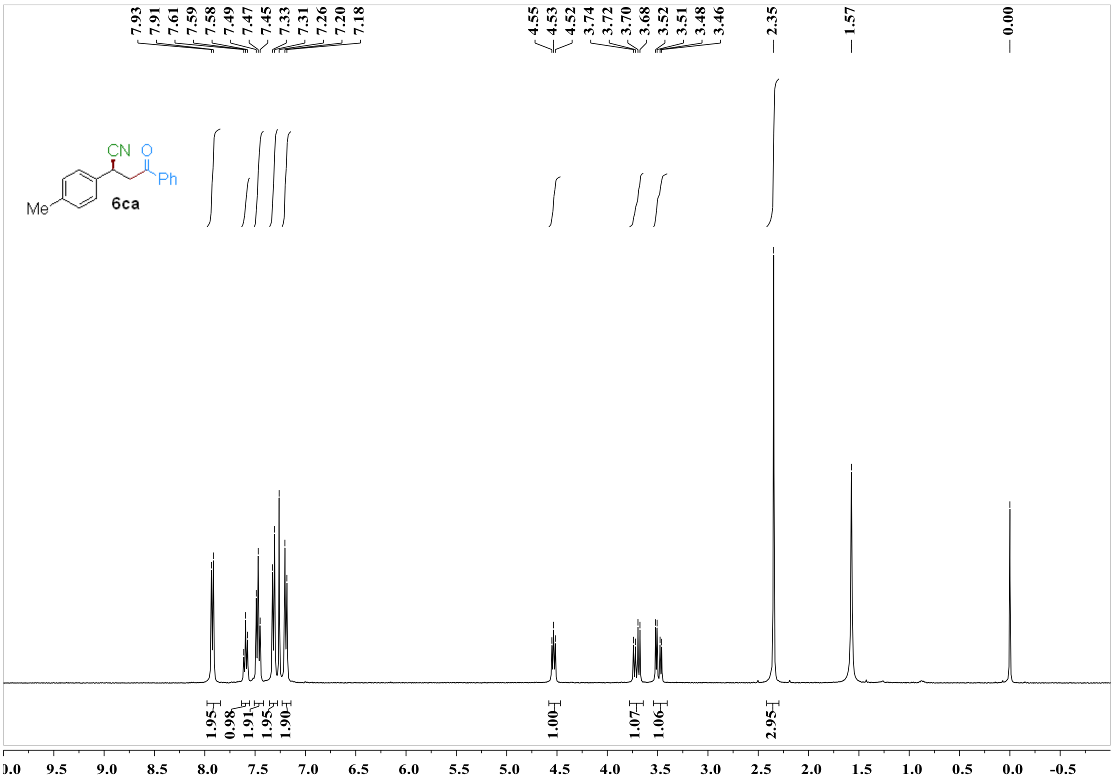

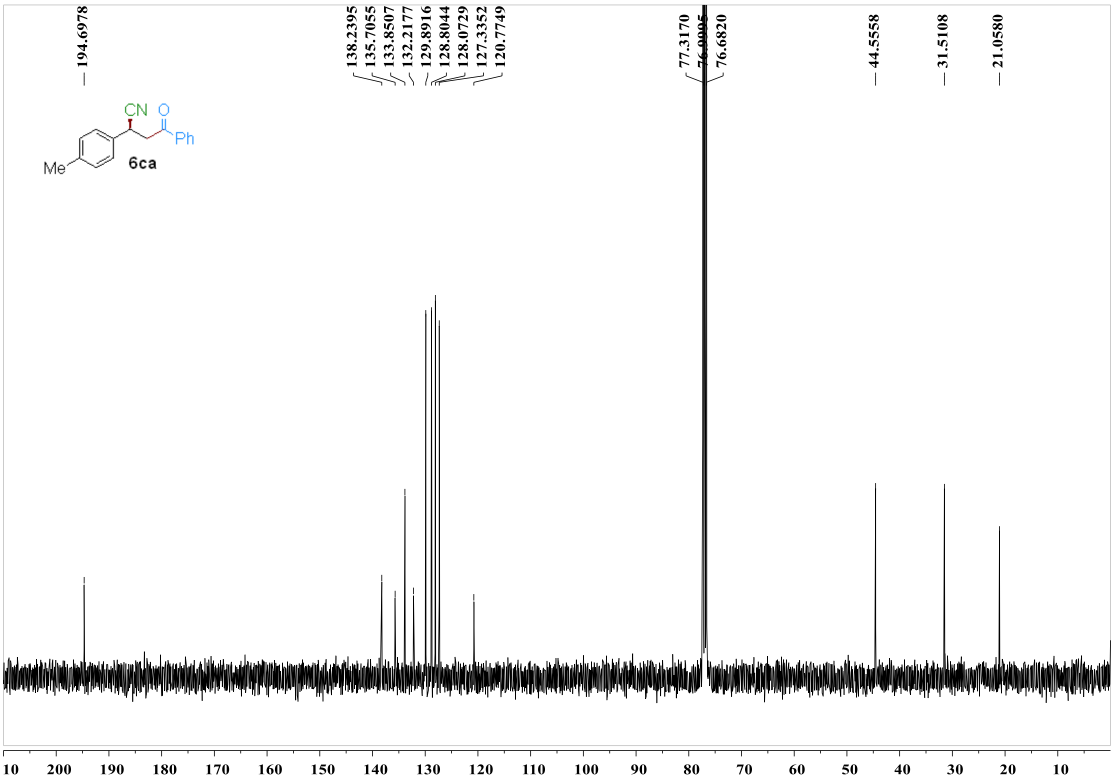

^{1}H NMR (400 MHz, $CDCl_3$) and ^{13}C NMR (100 MHz, $CDCl_3$) spectra of substrate 6da

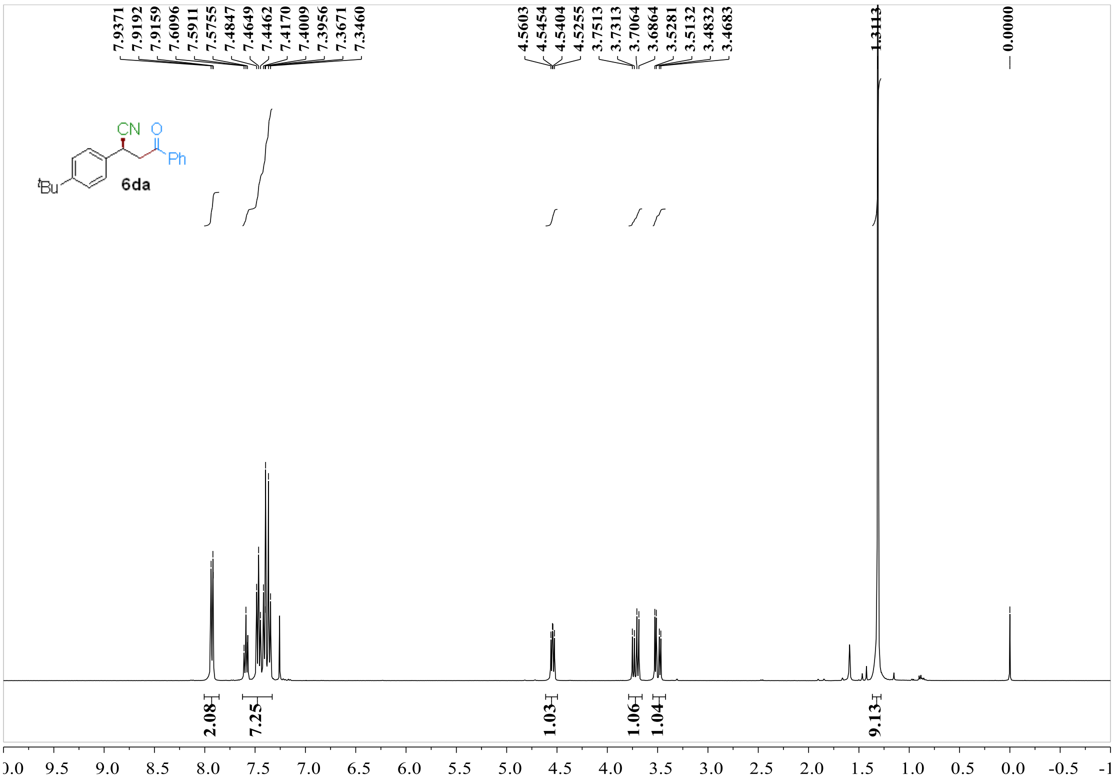

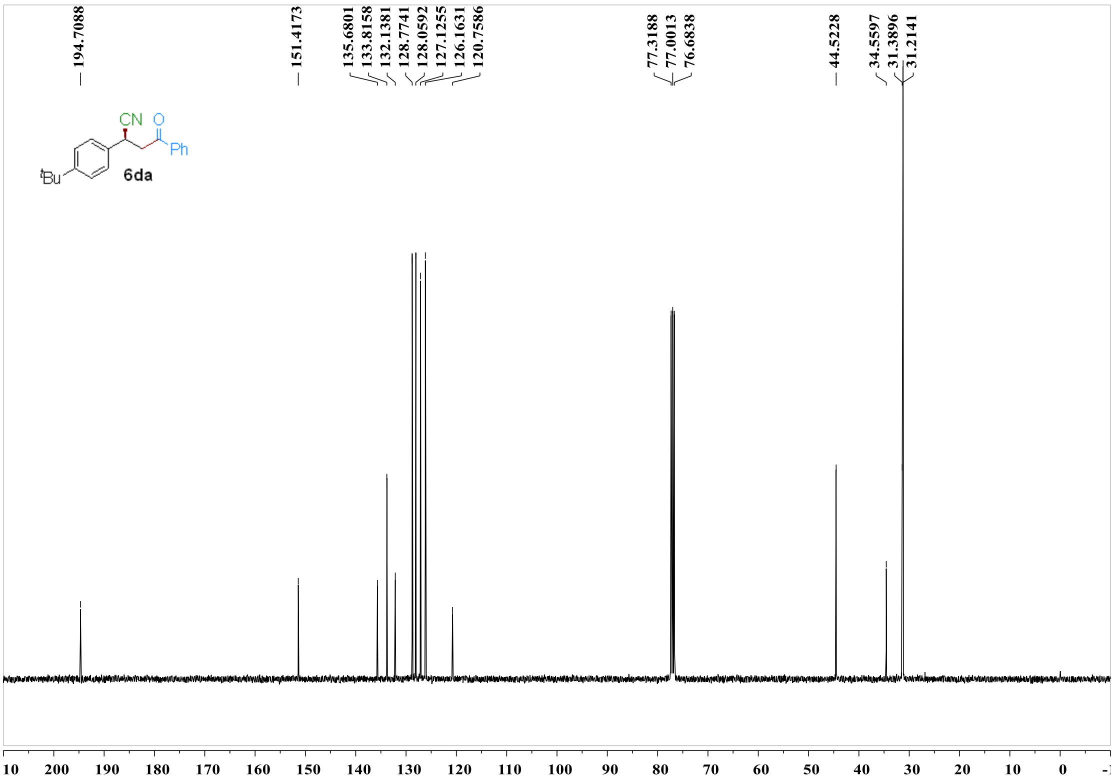

^{1}H NMR (400 MHz, CDCl3) and ^{13}C NMR (100 MHz, CDCl3) spectra of substrate 6ea

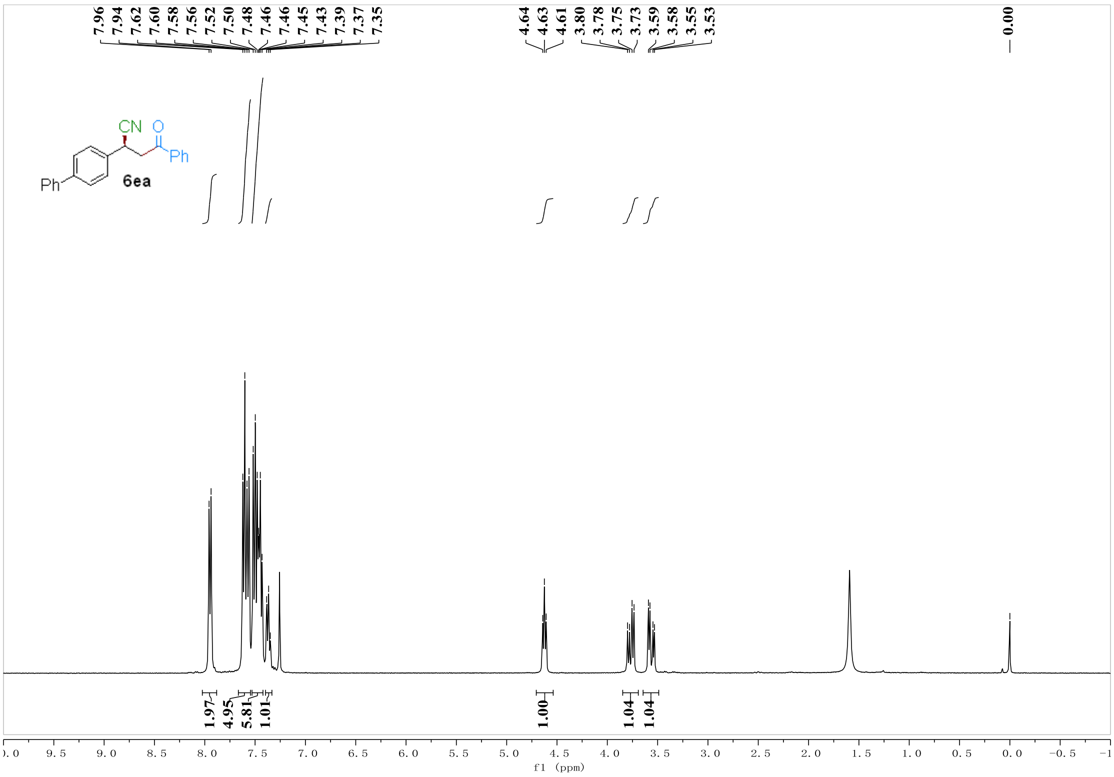

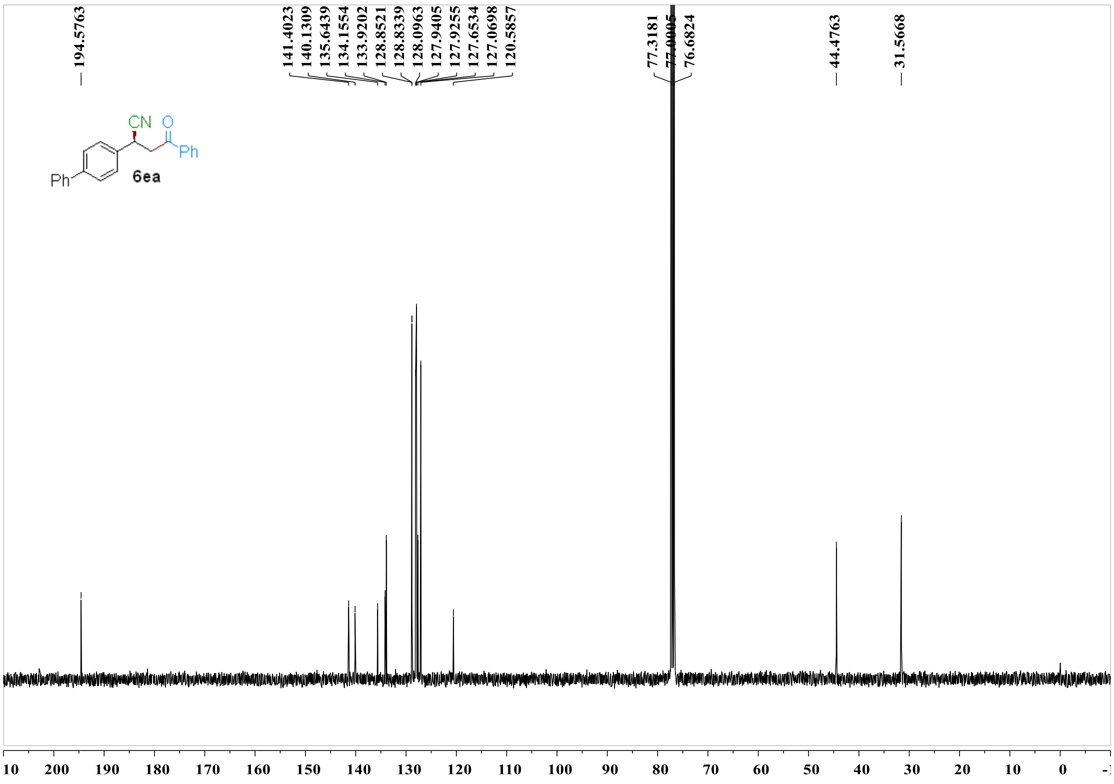

^{1}H NMR (400 MHz, $CDCl_3$), ^{13}C NMR (100 MHz, $CDCl_3$), ^{19}F NMR (376 MHz, $CDCl_3$) spectra of substrate 6fa

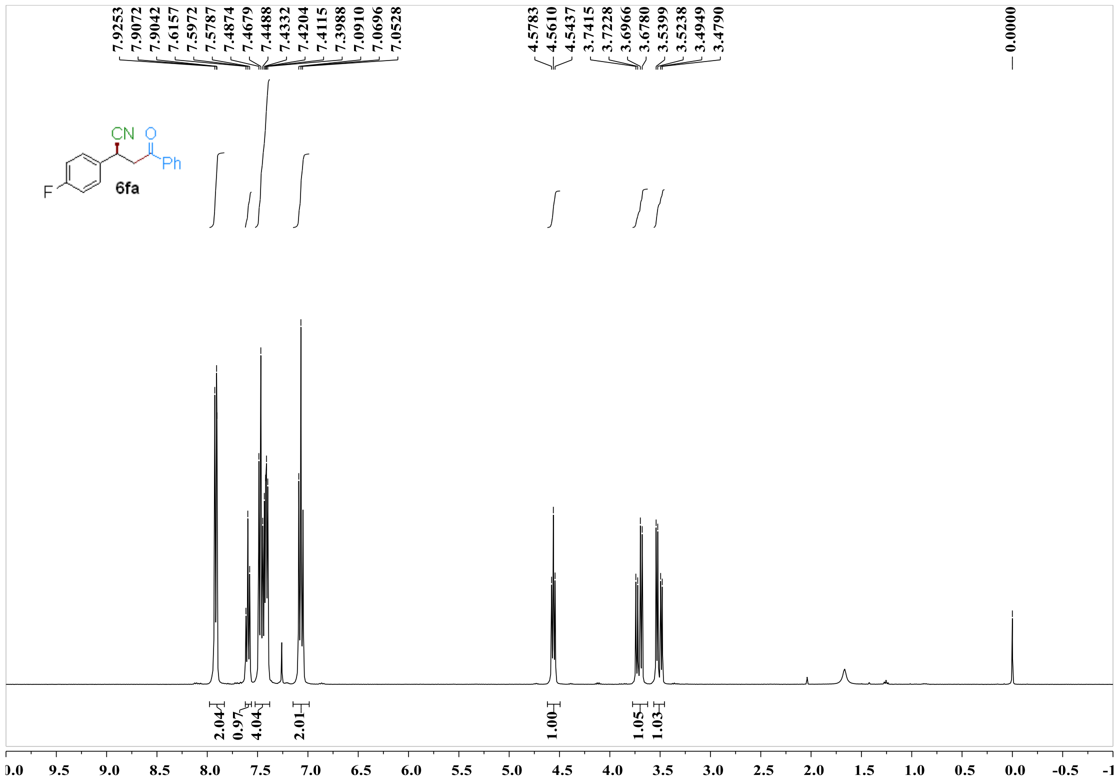

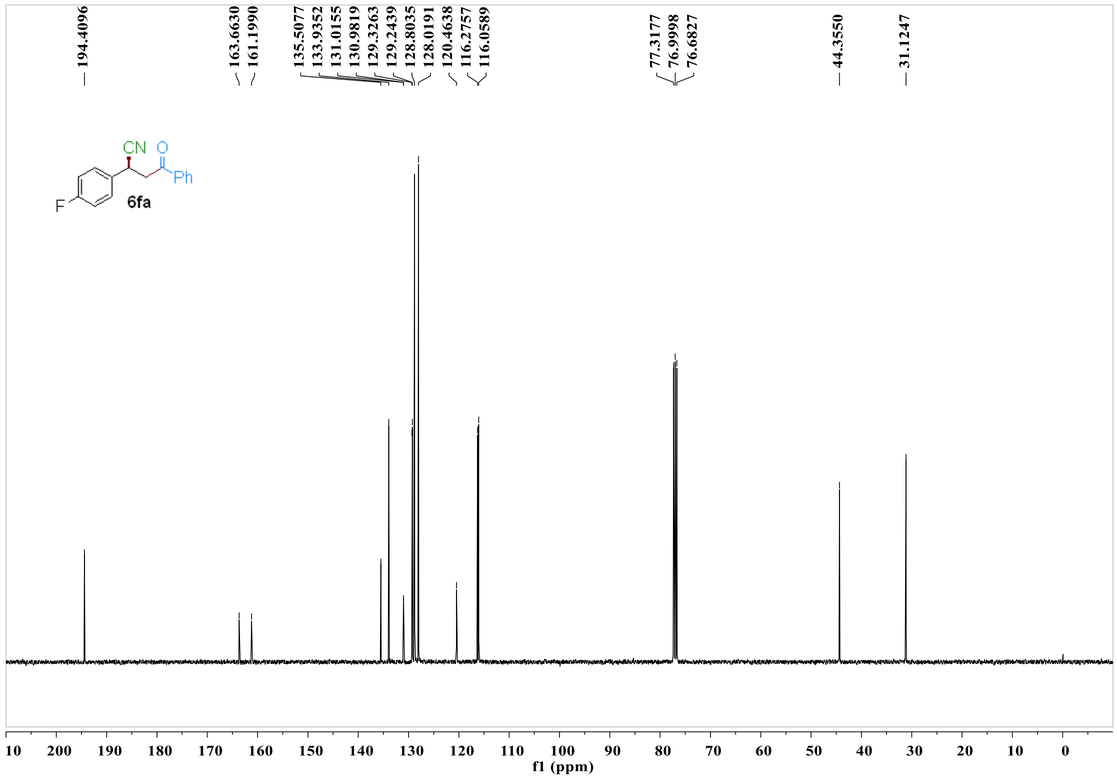

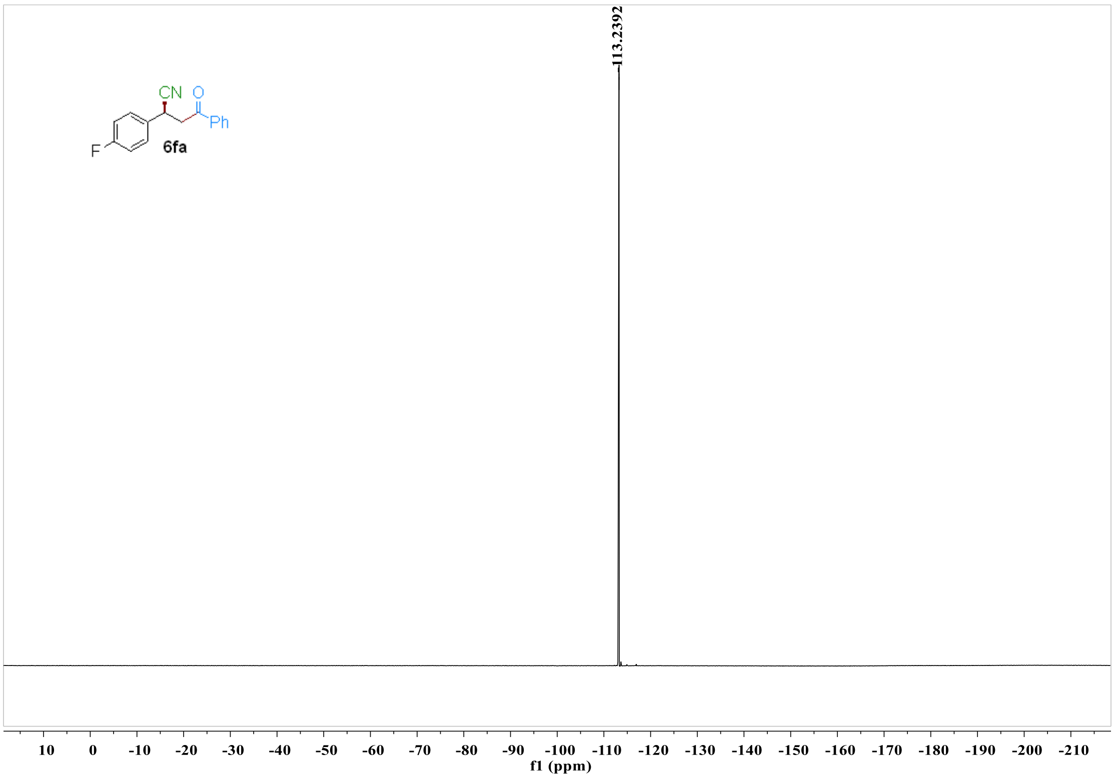
-113.2392
CN
O
Ph
F
6fa
10
0
-10
-20
-30
-40
-50
-60
-70
-80
-90
-100
-110
-120
-130
-140
-150
-160
-170
-180
-190
-200
-210
f1 (ppm)

^{1}H NMR (400 MHz, $CDCl_3$) and ^{13}C NMR (100 MHz, $CDCl_3$) spectra of substrate 6ga

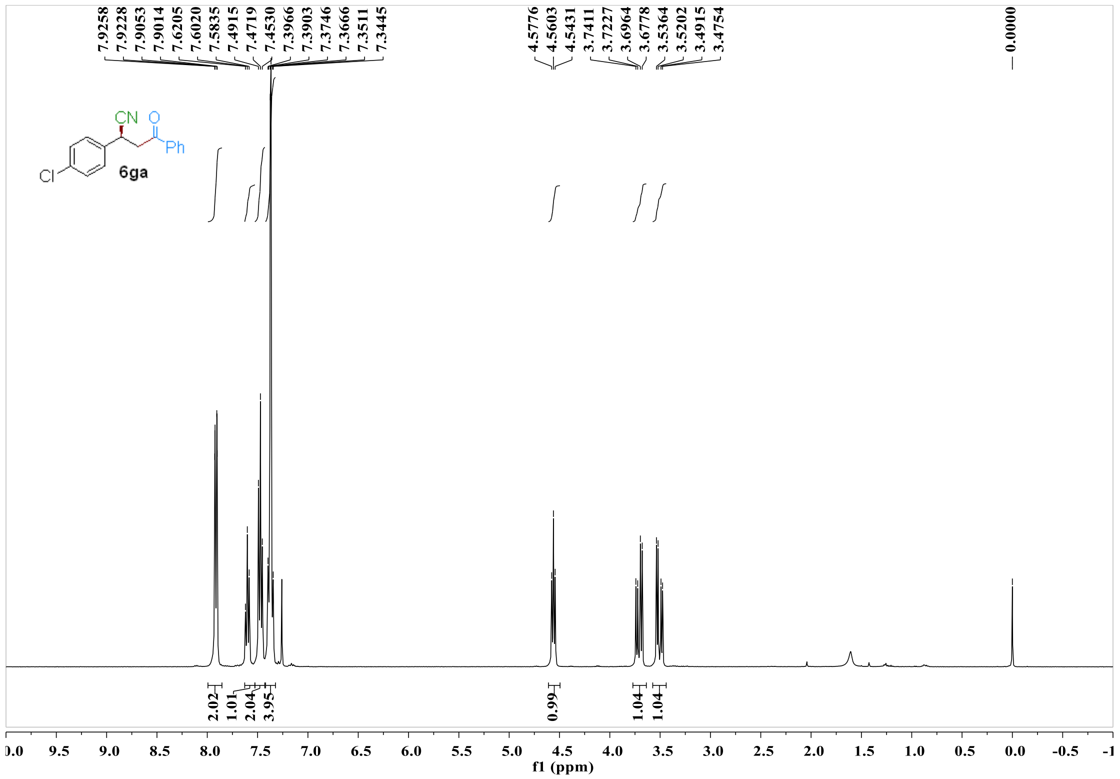

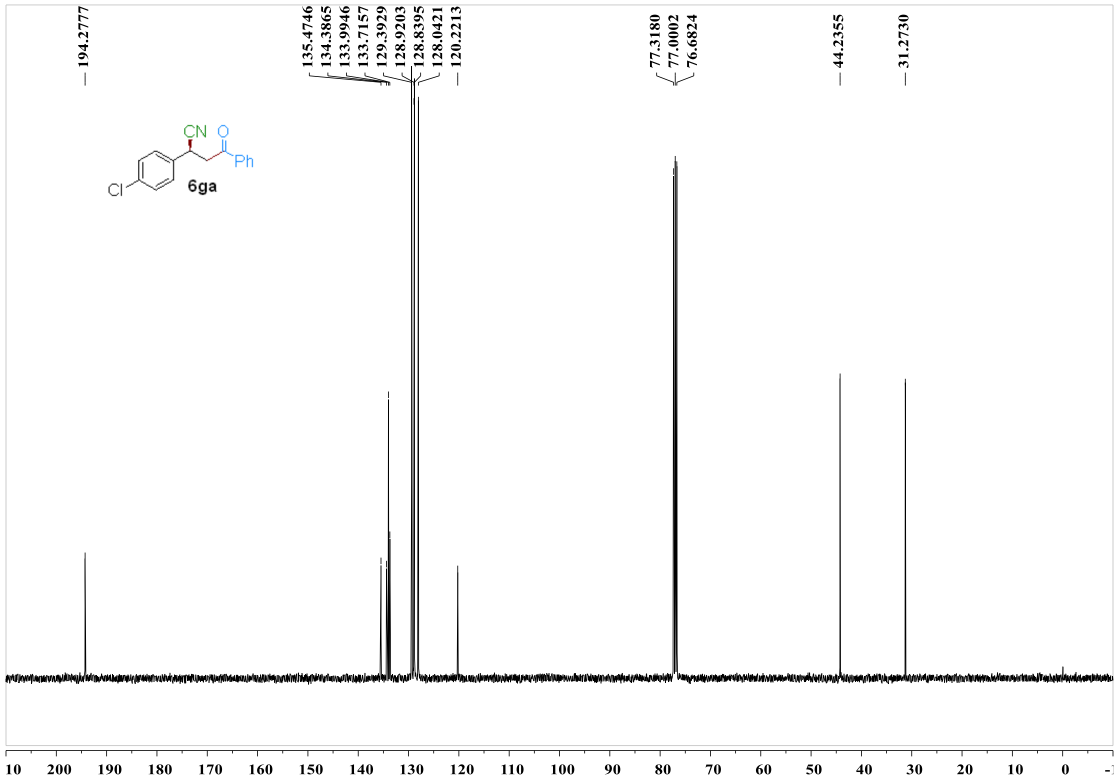

^{1}H NMR (400 MHz, $CDCl_3$) and ^{13}C NMR (100 MHz, $CDCl_3$) spectra of substrate 6ha

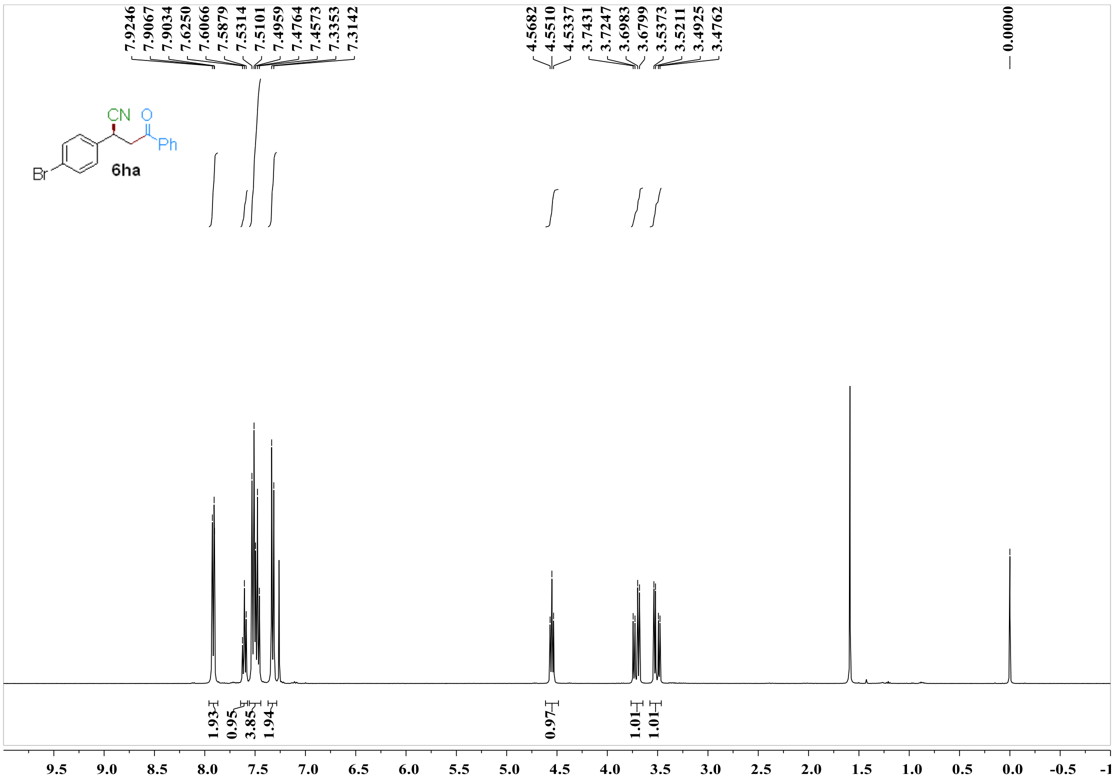

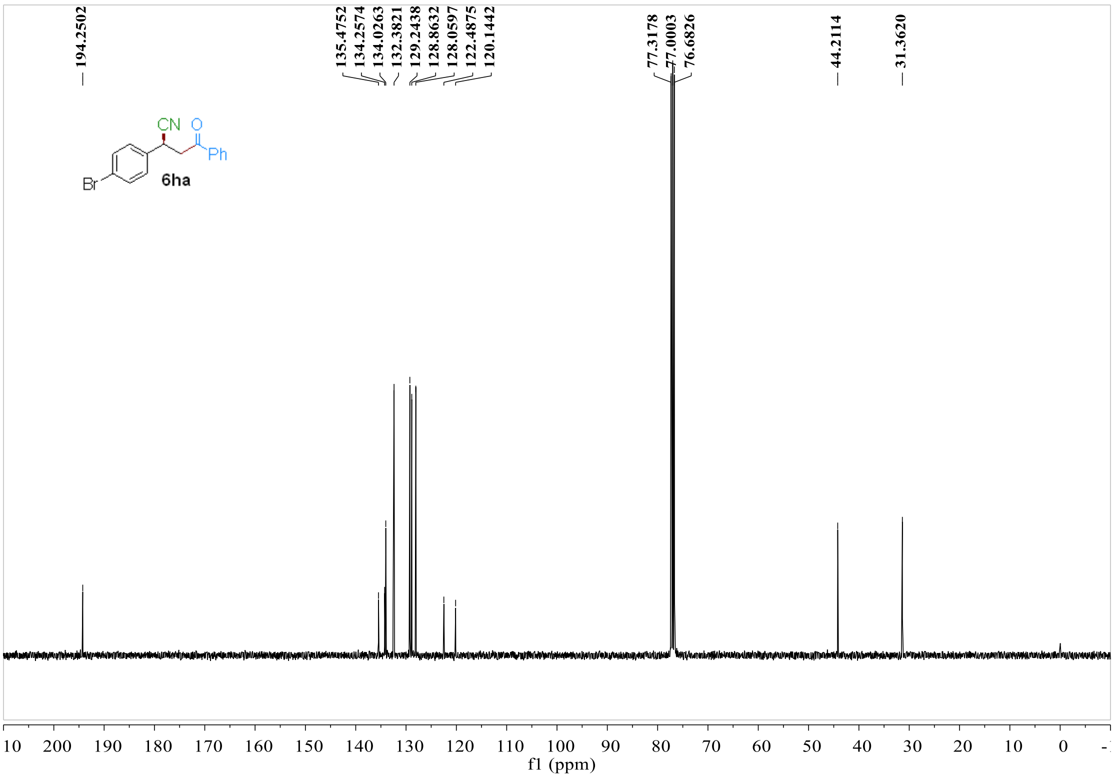

^{1}H NMR (400 MHz, $CDCl_3$) and ^{13}C NMR (100 MHz, $CDCl_3$) spectra of substrate 6ia

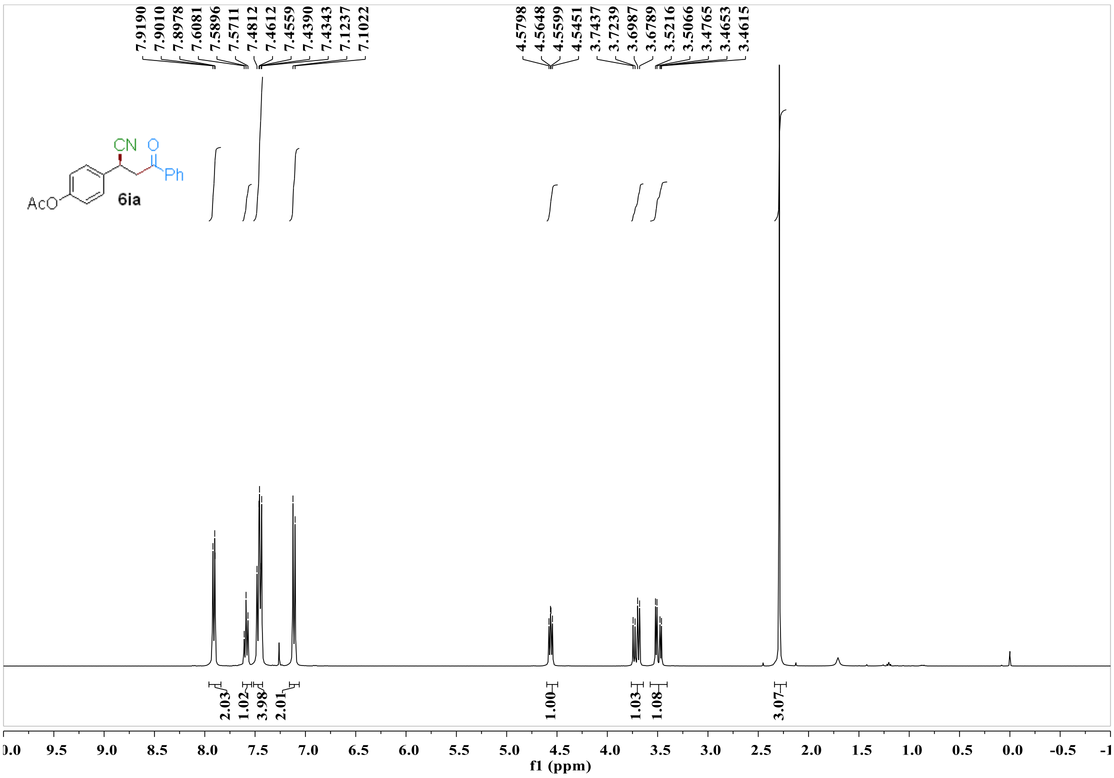

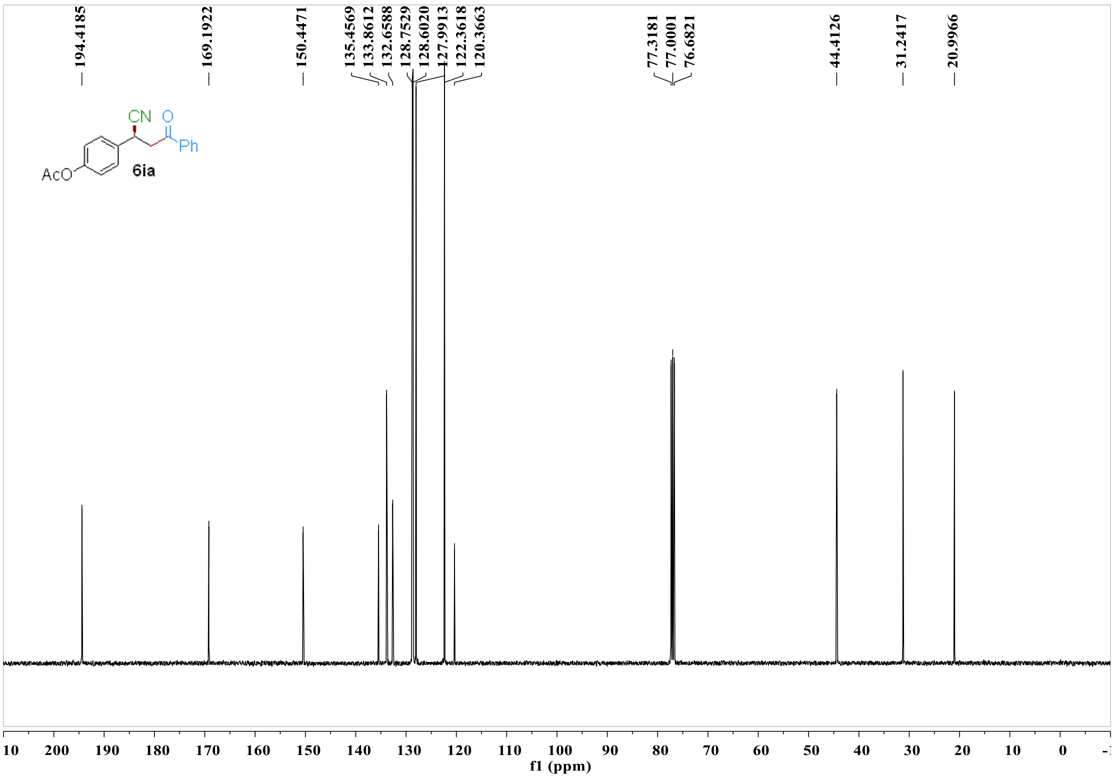

^{1}H NMR (400 MHz, $CDCl_3$) and ^{13}C NMR (100 MHz, $CDCl_3$) spectra of substrate 6ja

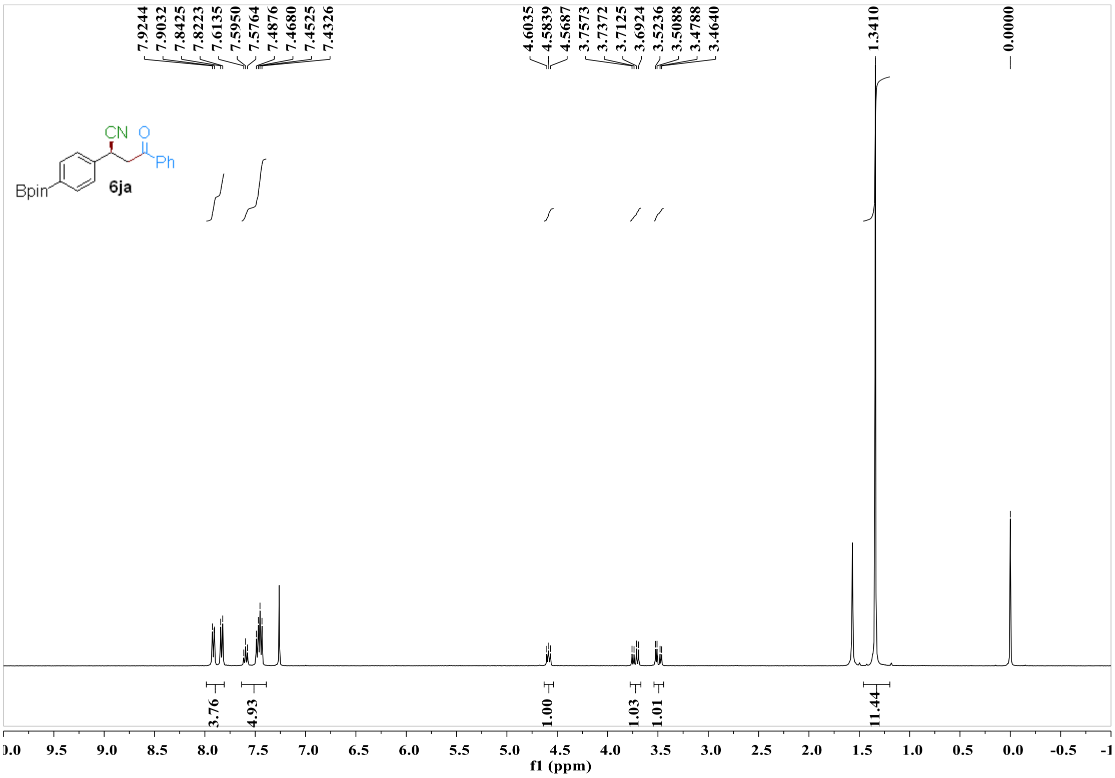

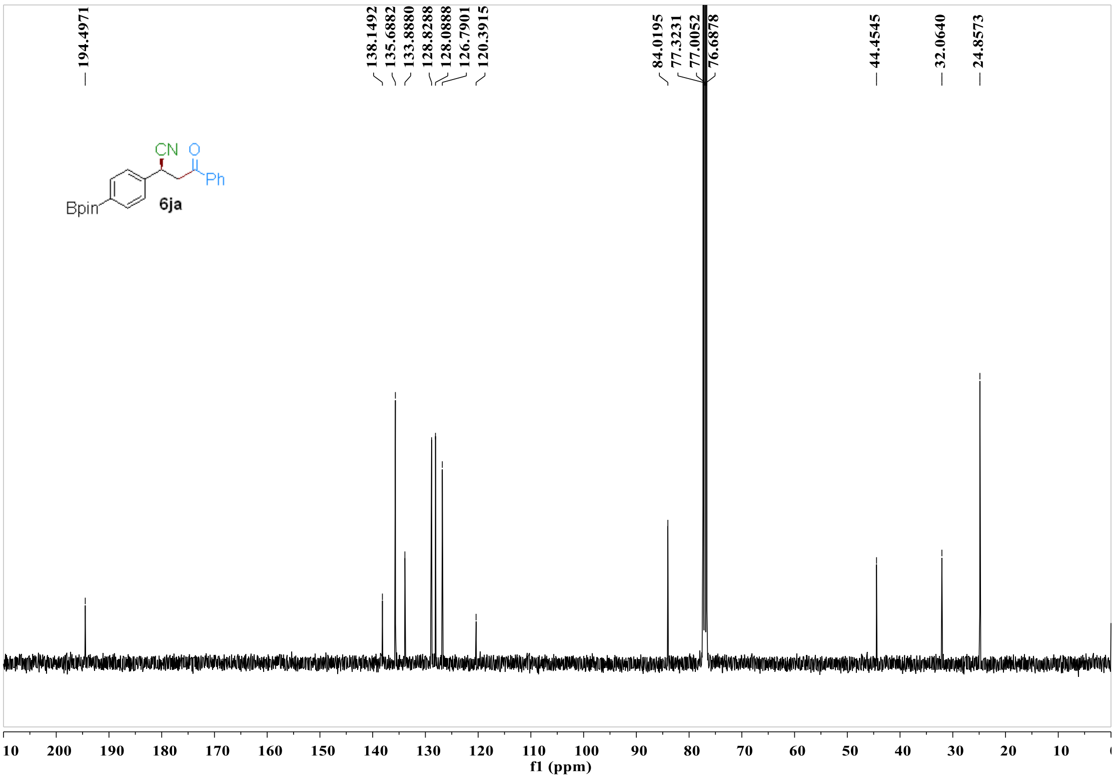

^{1}H NMR (400 MHz, $CDCl_3$) and ^{13}C NMR (100 MHz, $CDCl_3$) spectra of substrate 6ka

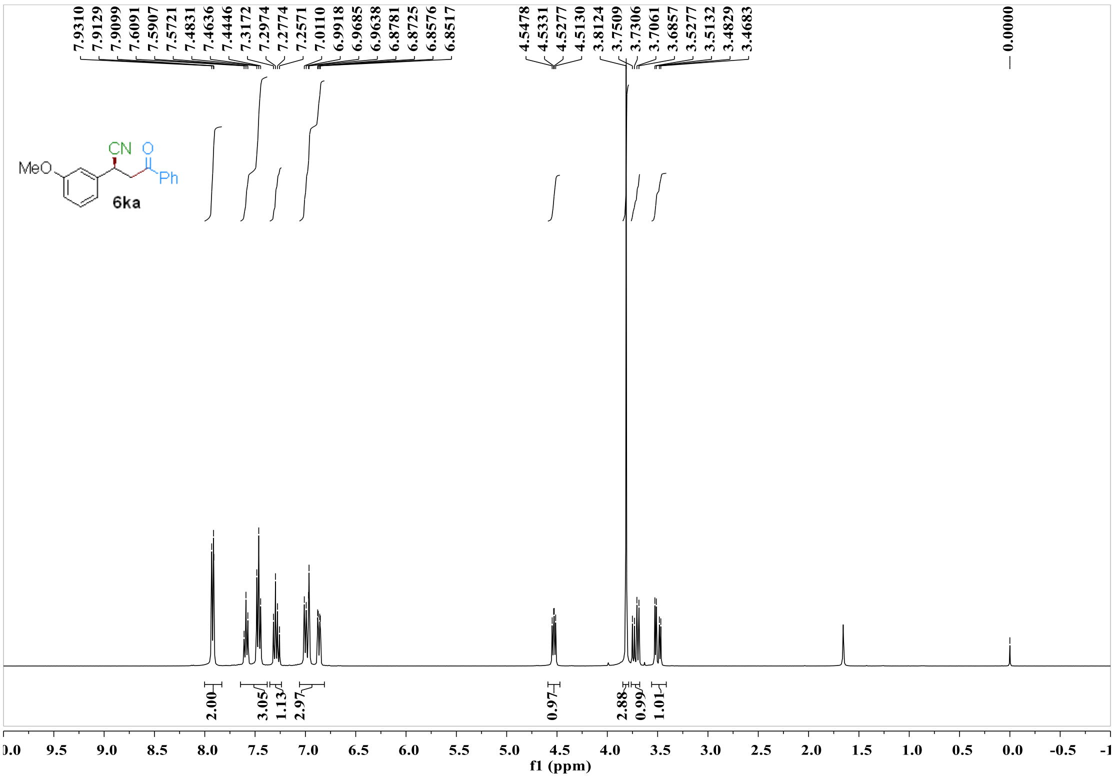

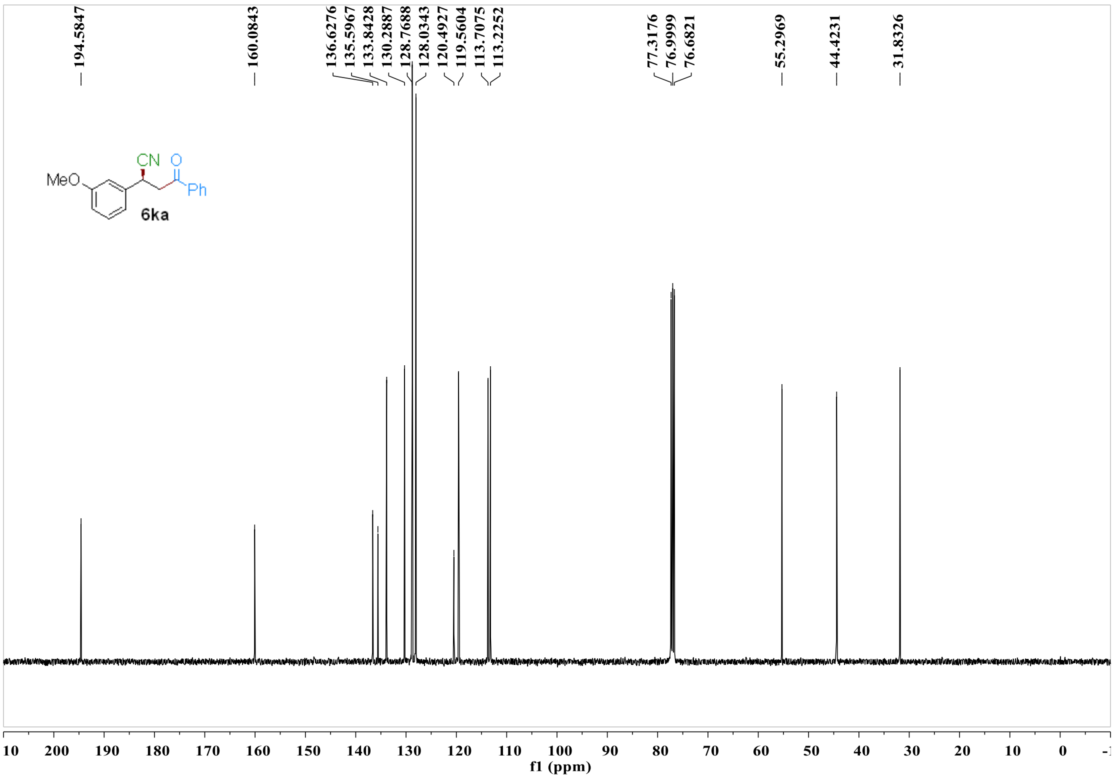

^{1}H NMR (400 MHz, CDCl$_3$) and ^{13}C NMR (100 MHz, CDCl$_3$) spectra of substrate 6la

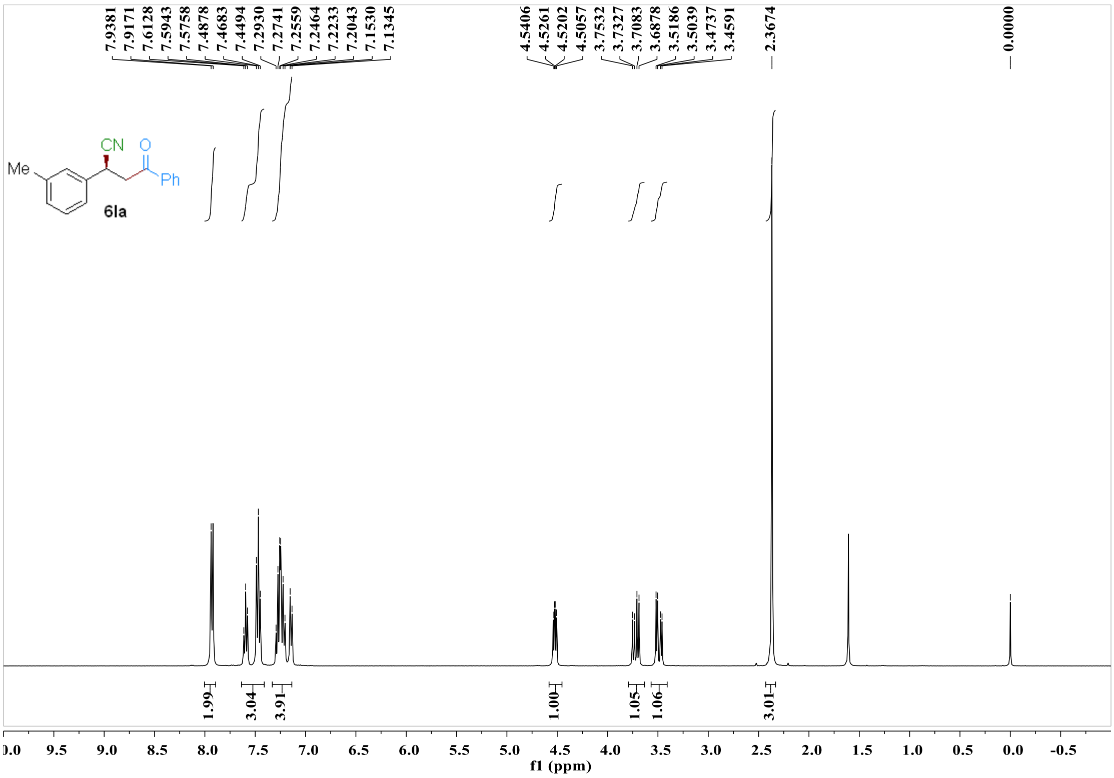

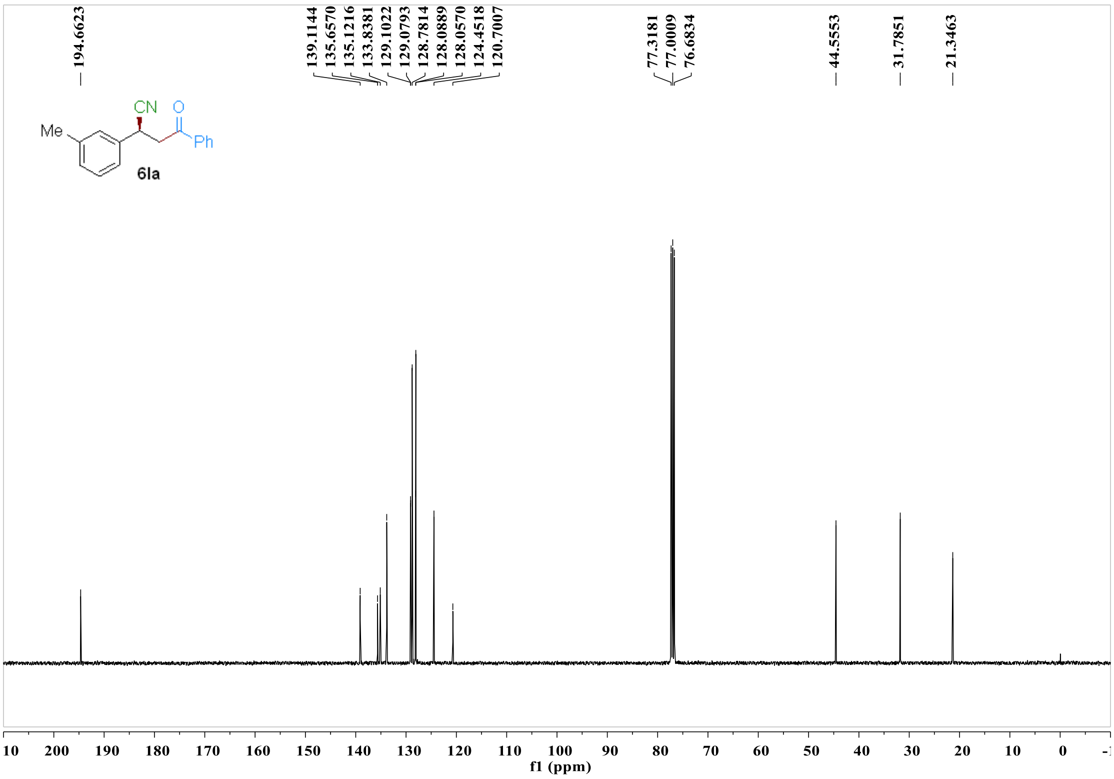

^{1}H NMR (400 MHz, CDCl3), ^{13}C NMR (100 MHz, CDCl3), ^{19}F NMR (376 MHz, CDCl3) spectra of substrate 6ma

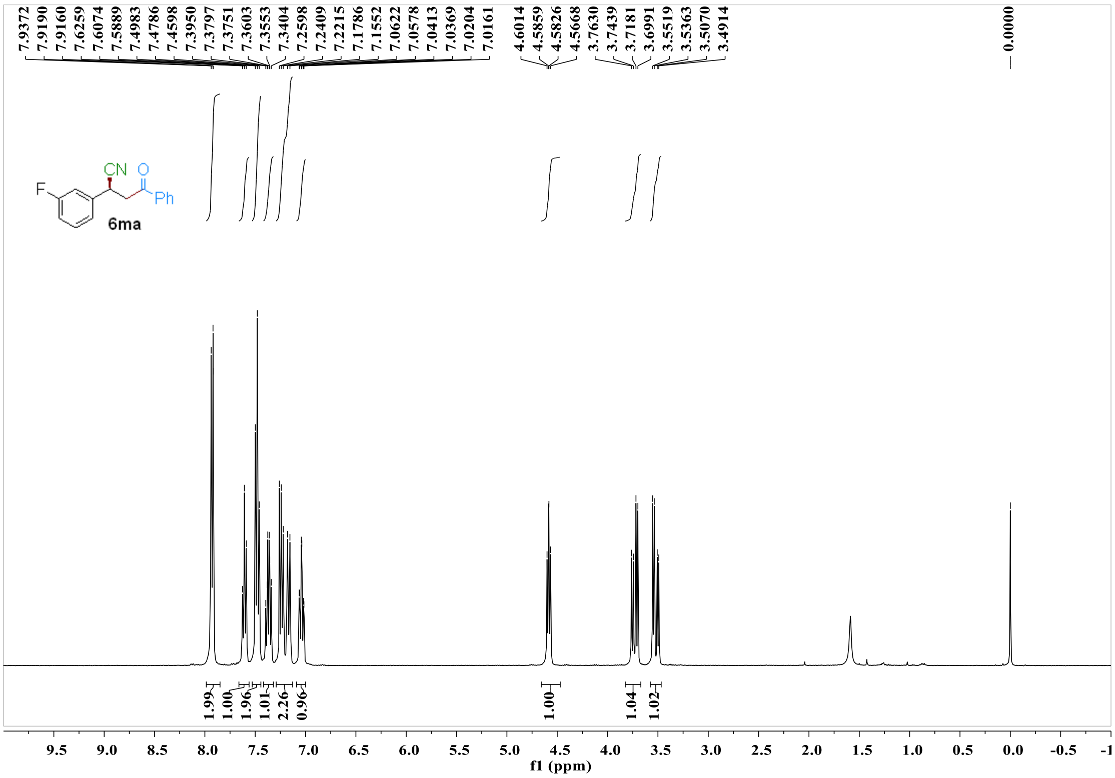

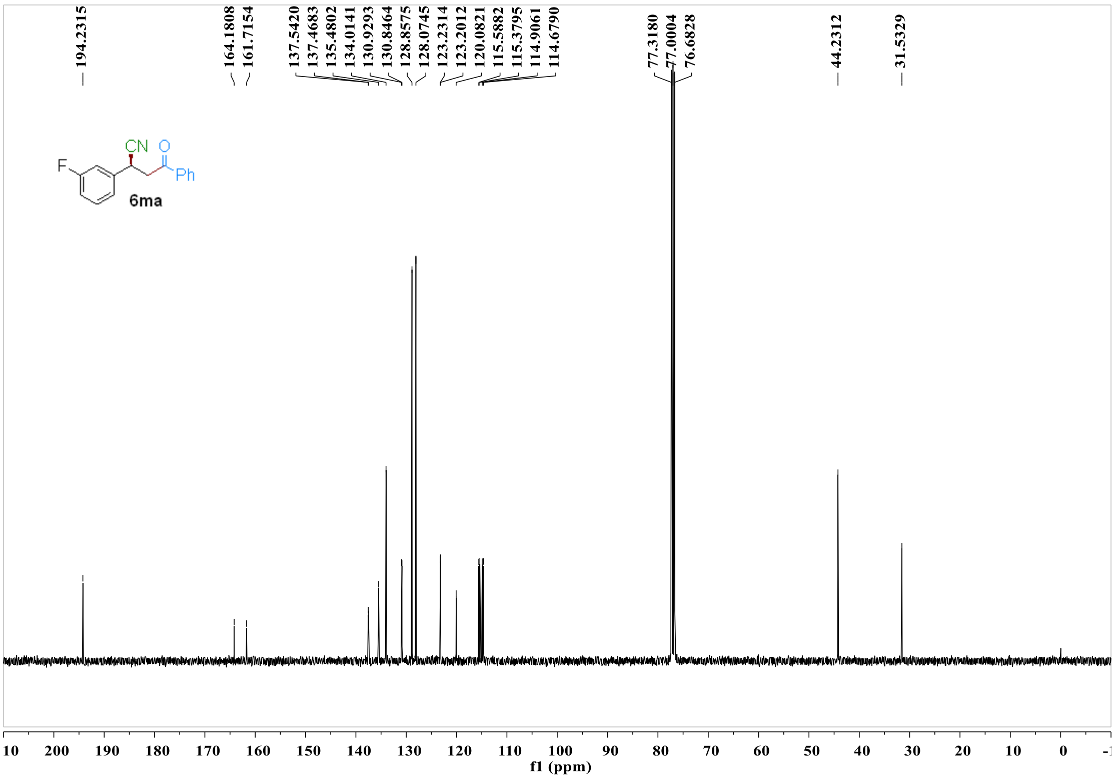

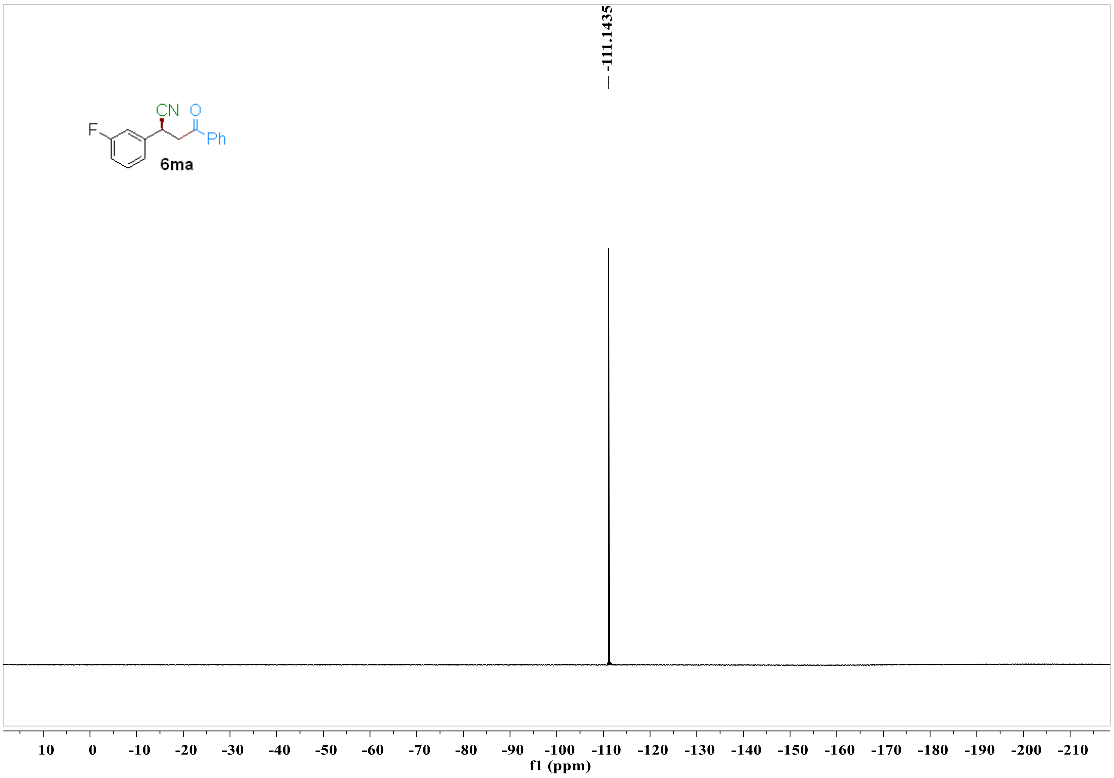
-111.1435
F
CN
O
Ph
6ma
10 0 -10 -20 -30 -40 -50 -60 -70 -80 -90 -100 -110 -120 -130 -140 -150 -160 -170 -180 -190 -200 -210
f1 (ppm)

^{1}H NMR (400 MHz, $CDCl_3$) and ^{13}C NMR (100 MHz, $CDCl_3$) spectra of substrate 6na

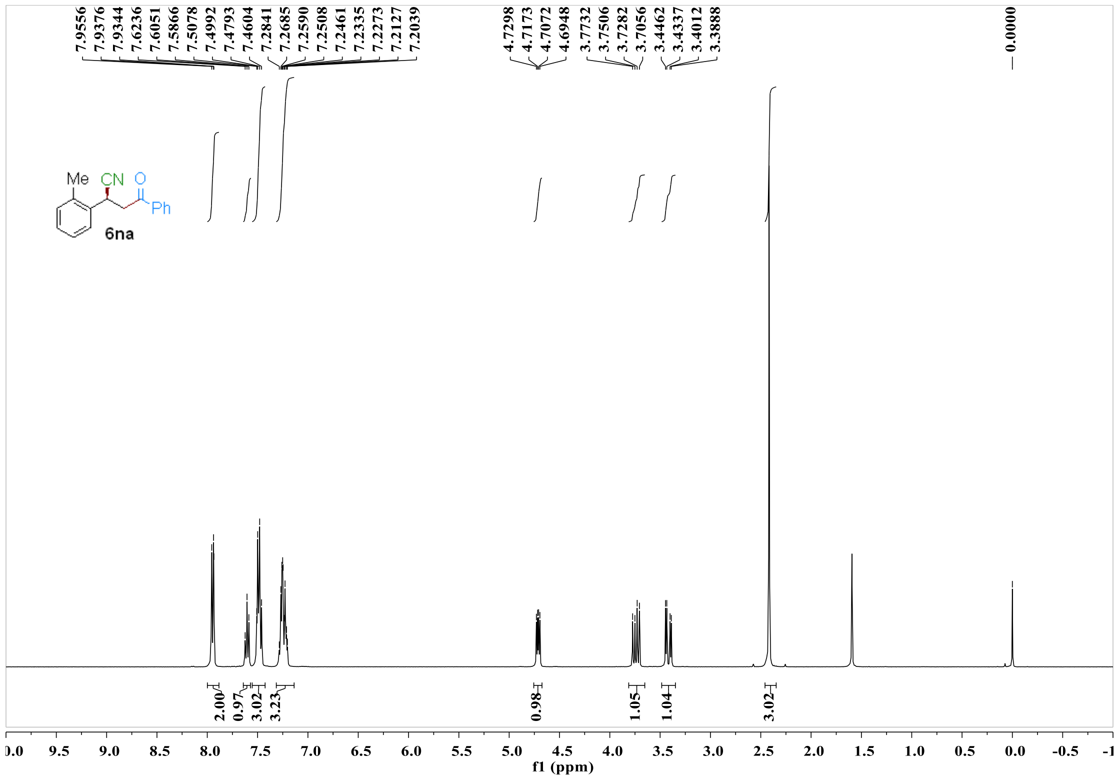

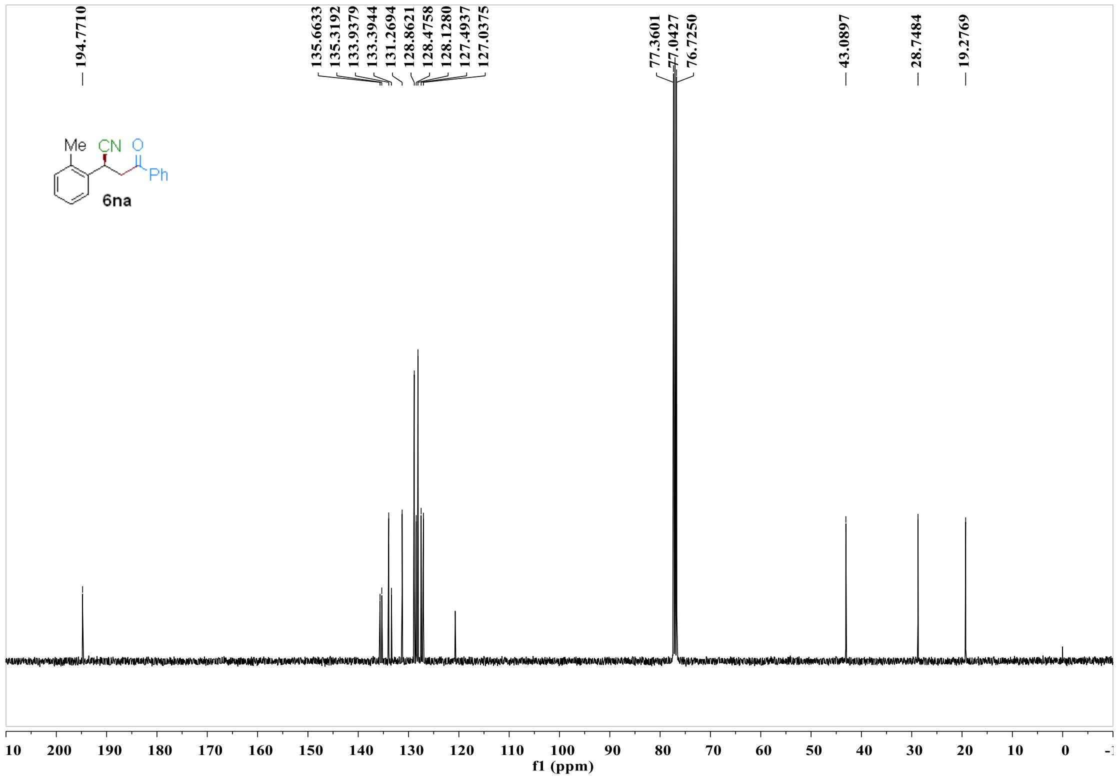

^{1}H NMR (400 MHz, $CDCl_3$) and ^{13}C NMR (100 MHz, $CDCl_3$) spectra of substrate 6oa

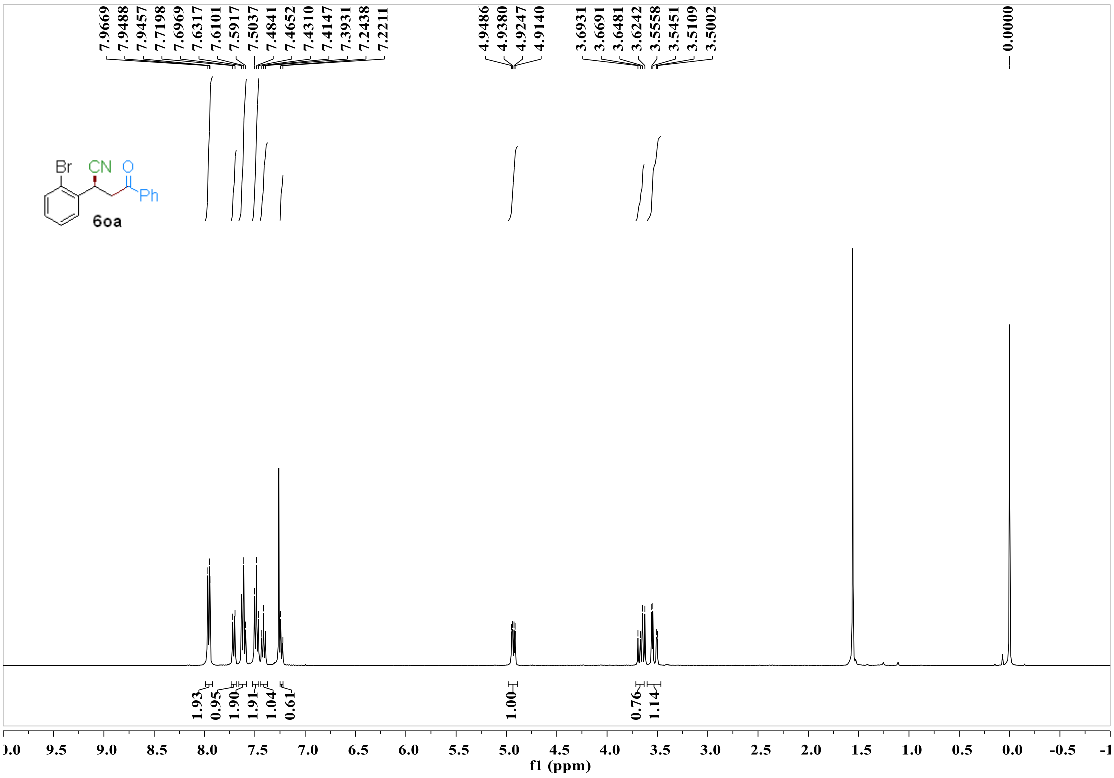

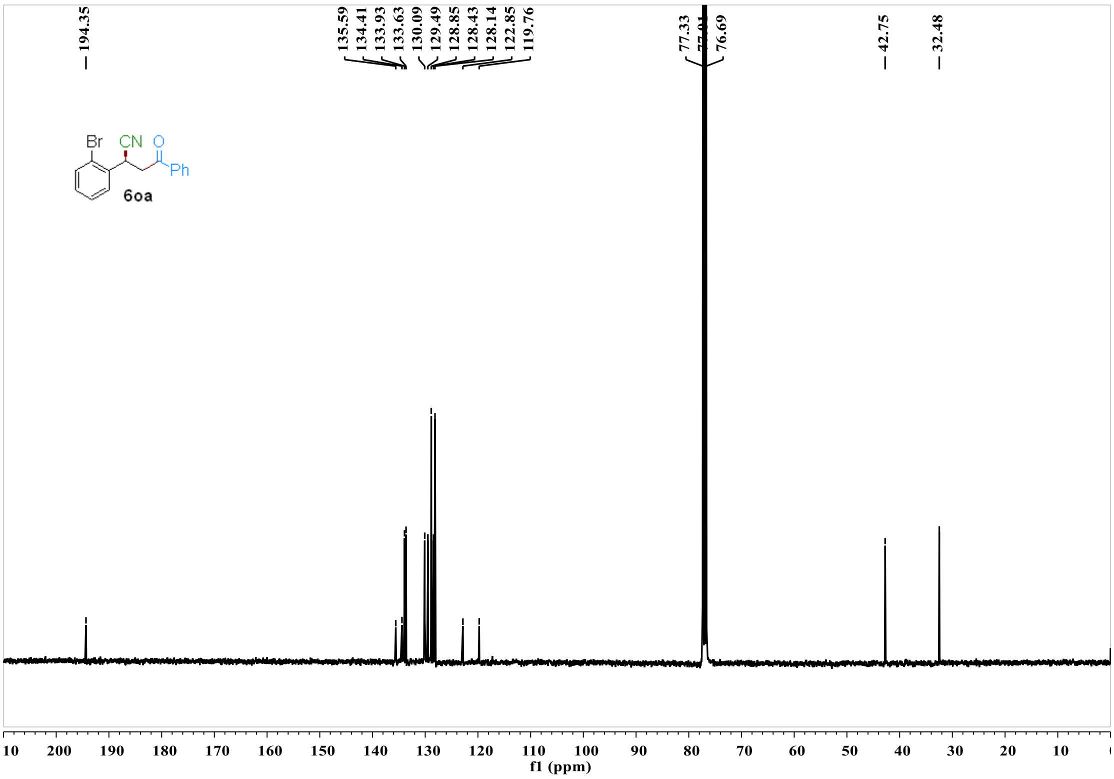

^{1}H NMR (400 MHz, CDCl3) and ^{13}C NMR (100 MHz, CDCl3) spectra of substrate 6pa

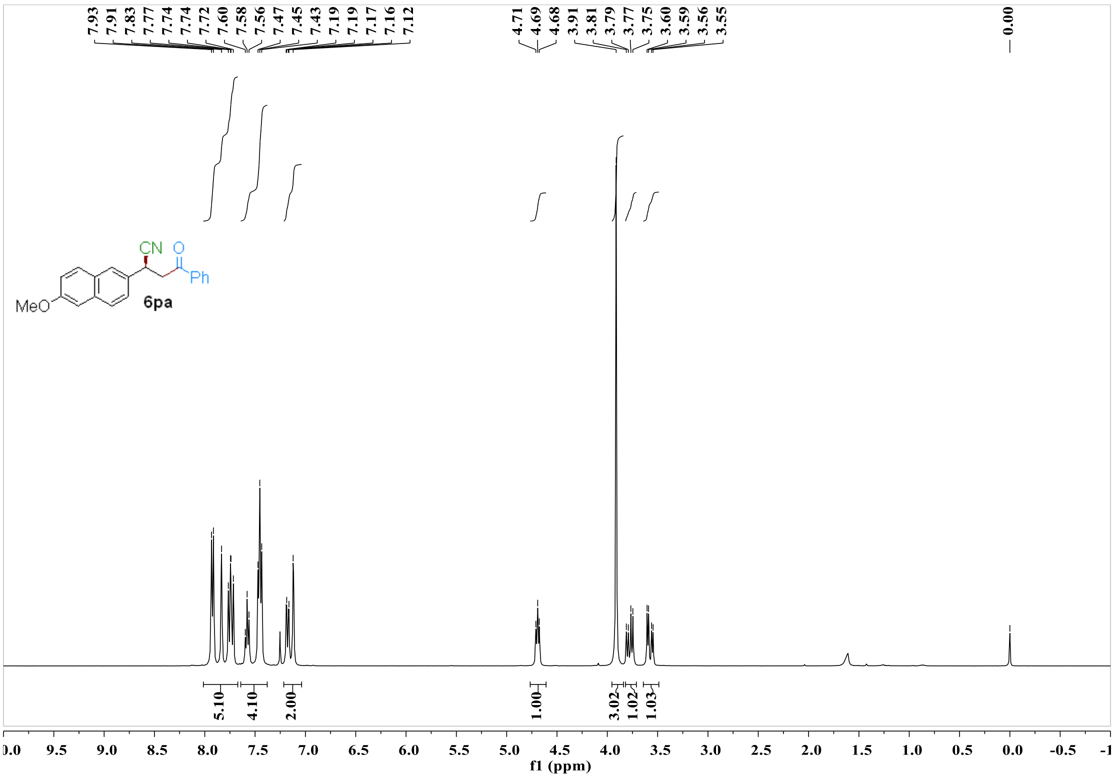

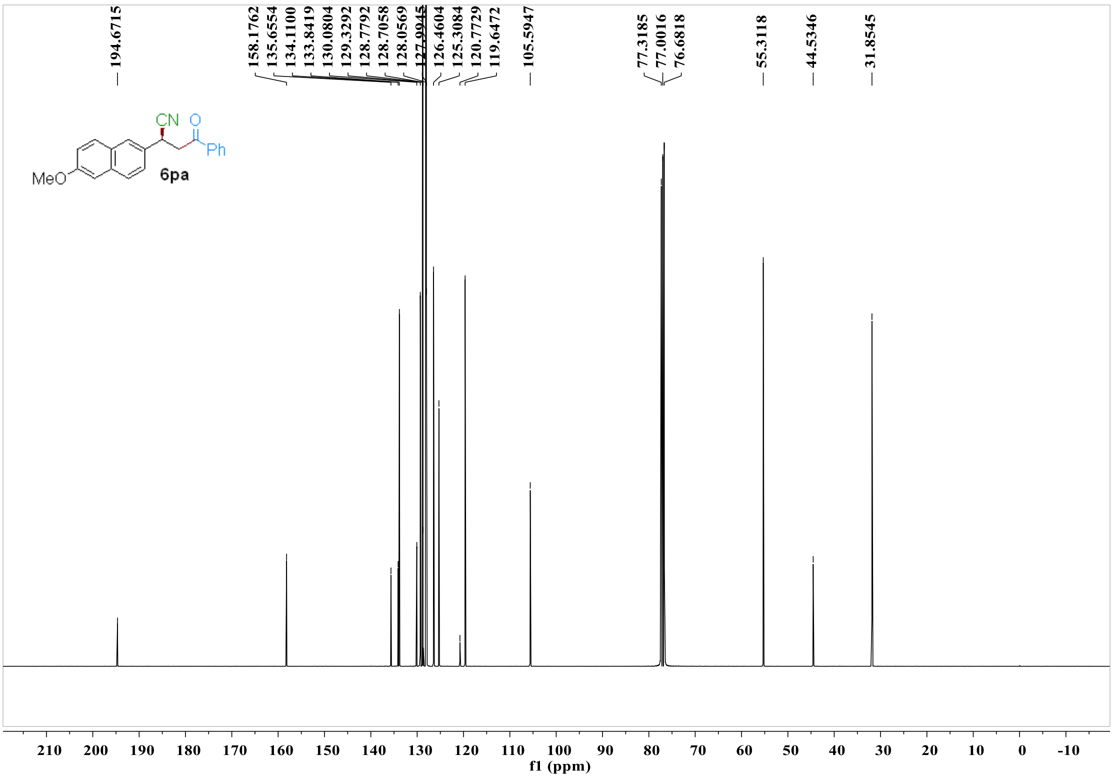

^{1}H NMR (400 MHz, $CDCl_3$) and ^{13}C NMR (100 MHz, $CDCl_3$) spectra of substrate 6qa

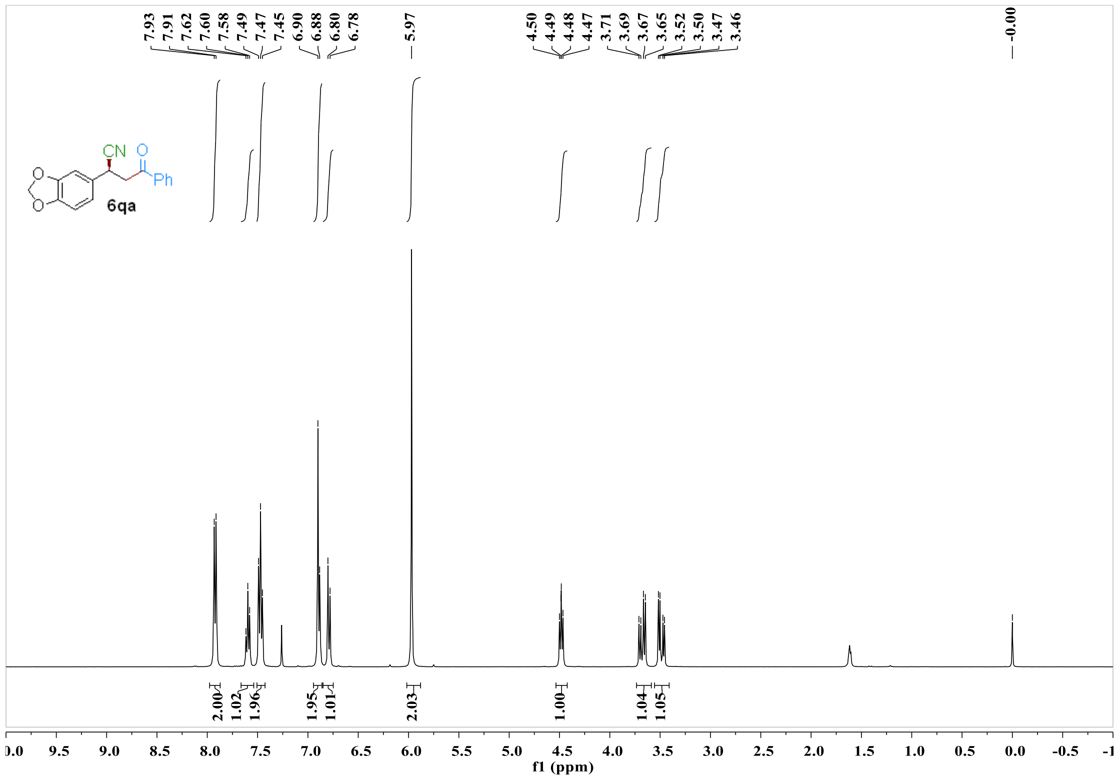

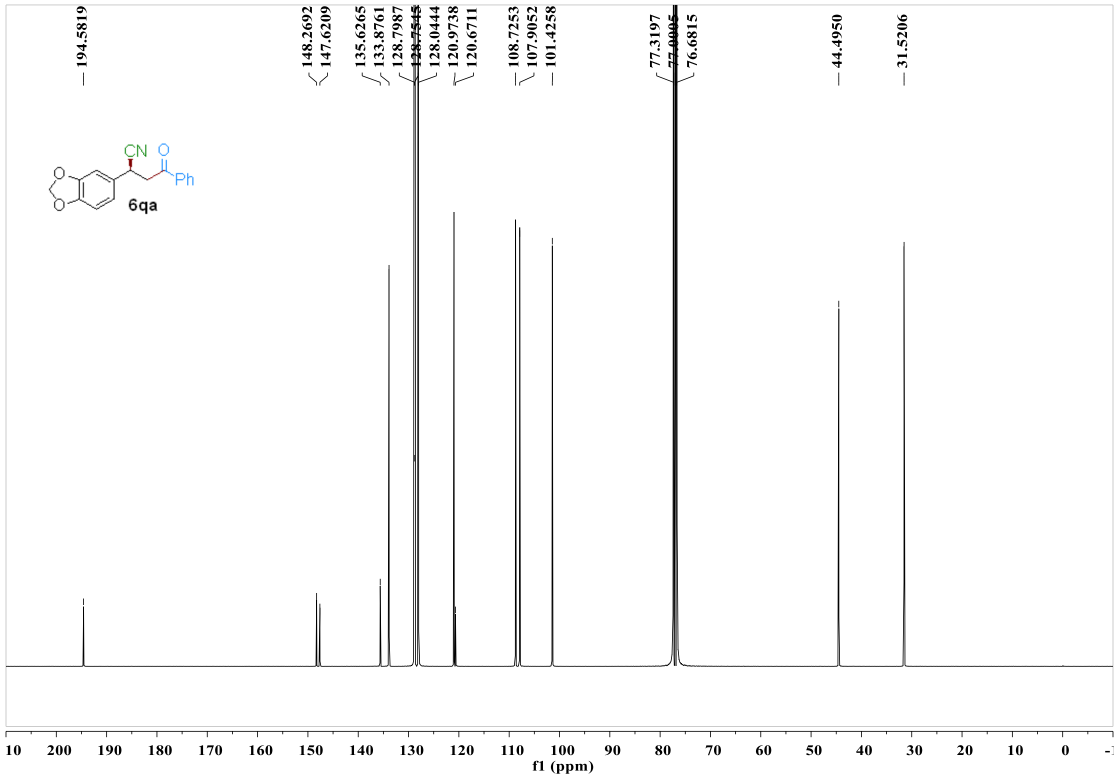

^{1}H NMR (400 MHz, CDCl3) and ^{13}C NMR (100 MHz, CDCl3) spectra of substrate 6ra

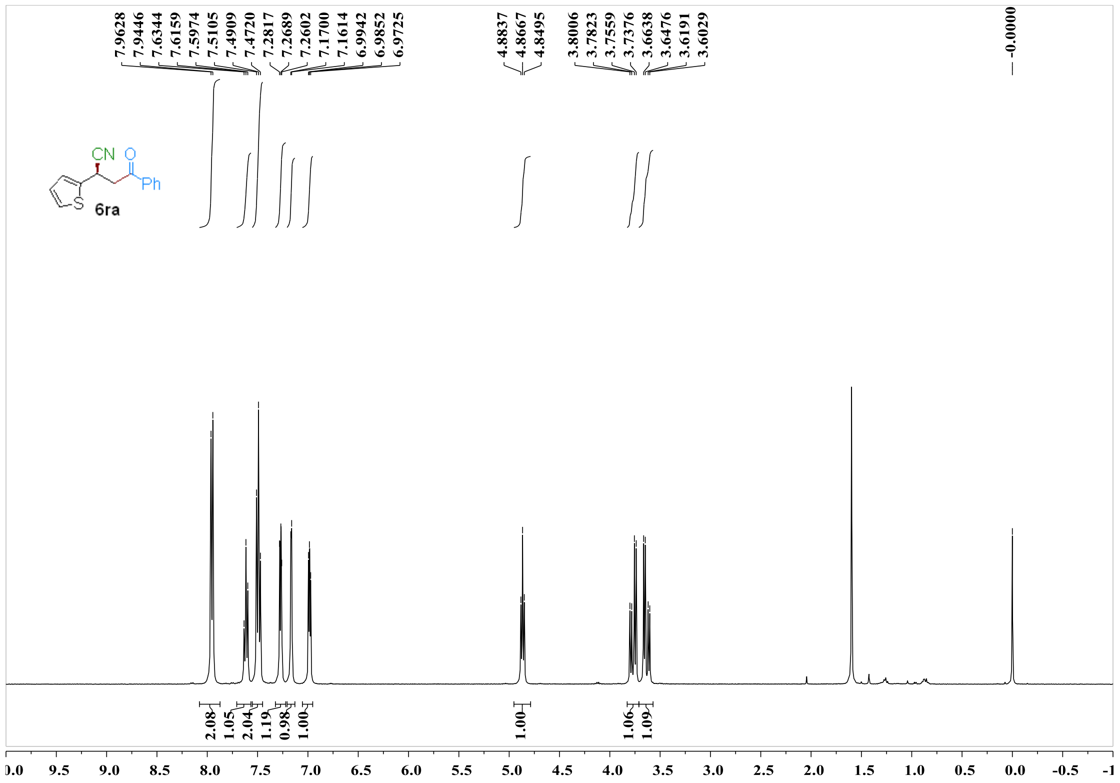

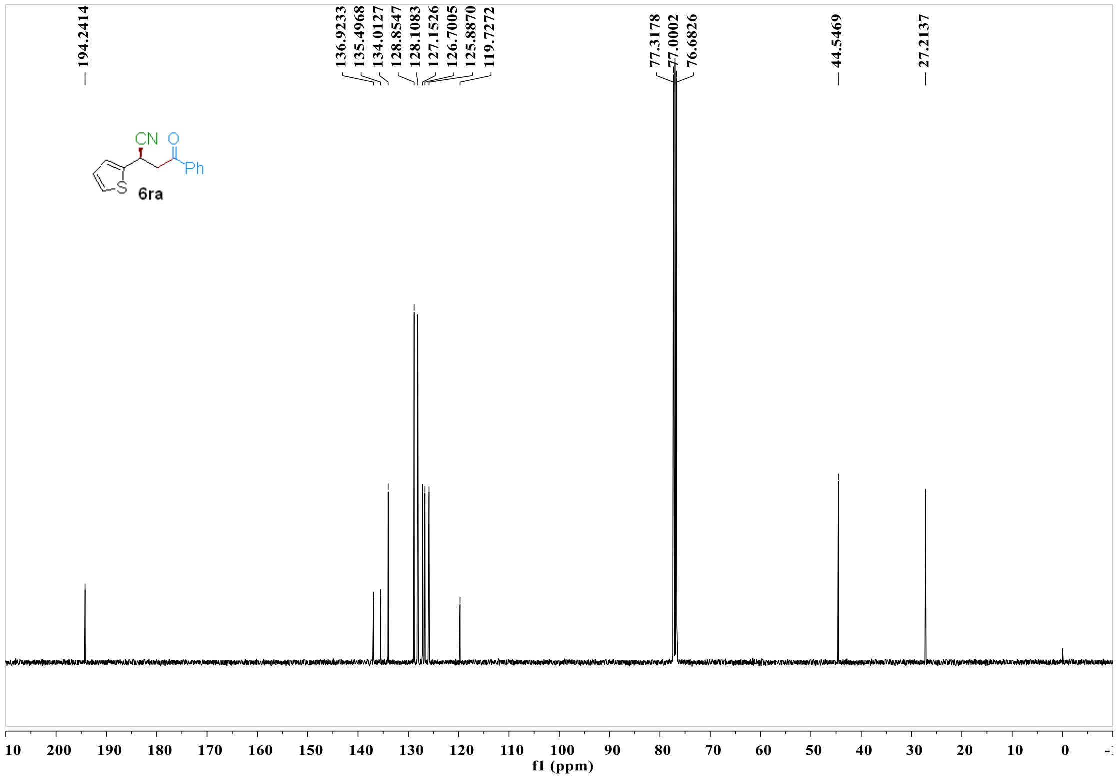

^{1}H NMR (400 MHz, CDCl3) and ^{13}C NMR (100 MHz, CDCl3) spectra of substrate 6sa

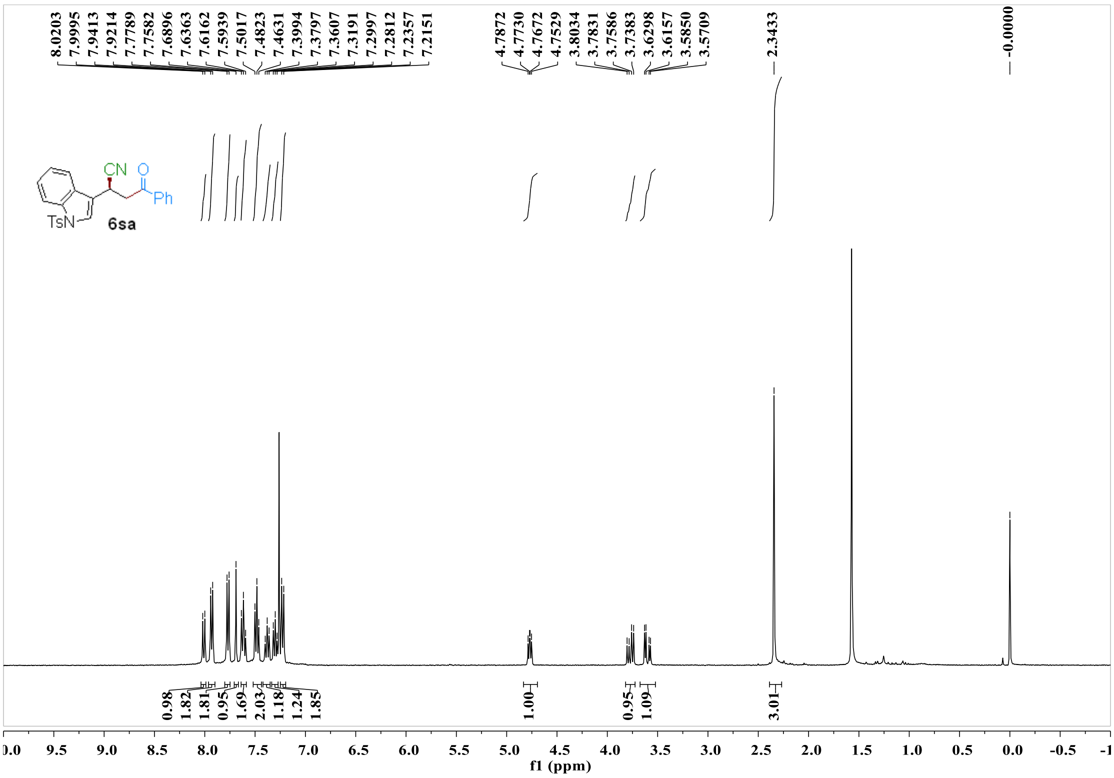

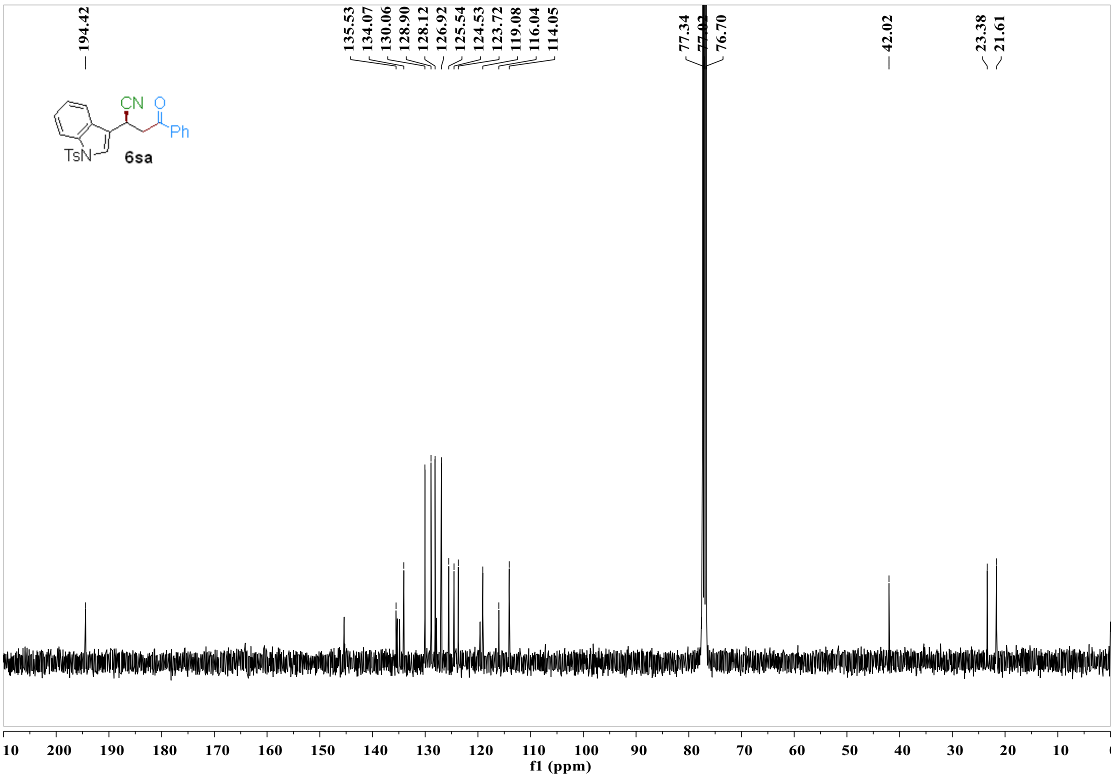

^{1}H NMR (400 MHz, $CDCl_3$) and ^{13}C NMR (100 MHz, $CDCl_3$) spectra of substrate 6ta

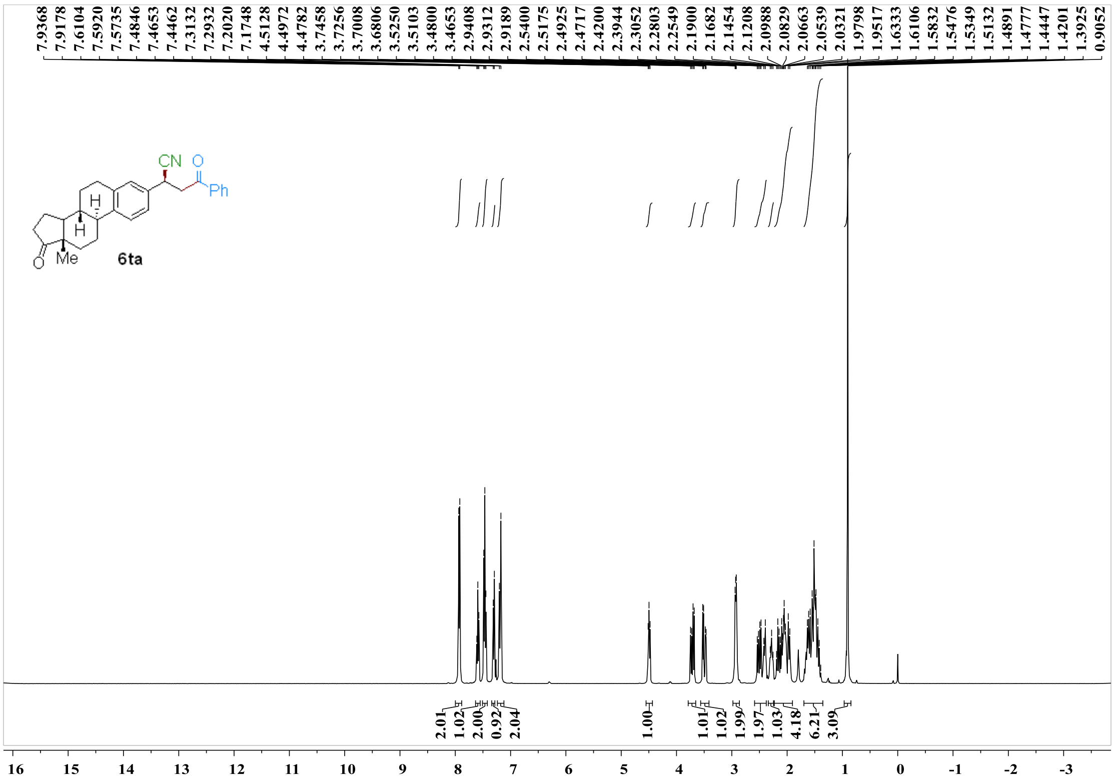

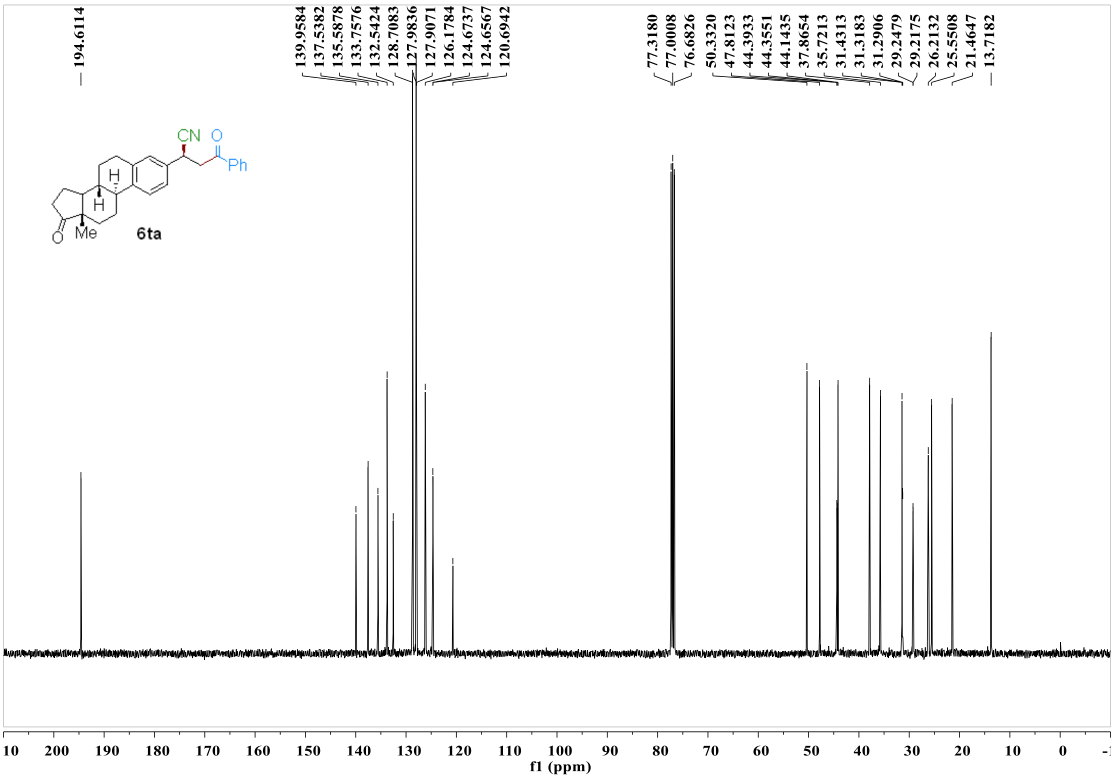

^{1}H NMR (400 MHz, CDCl3) and ^{13}C NMR (100 MHz, CDCl3) spectra of substrate 6ua

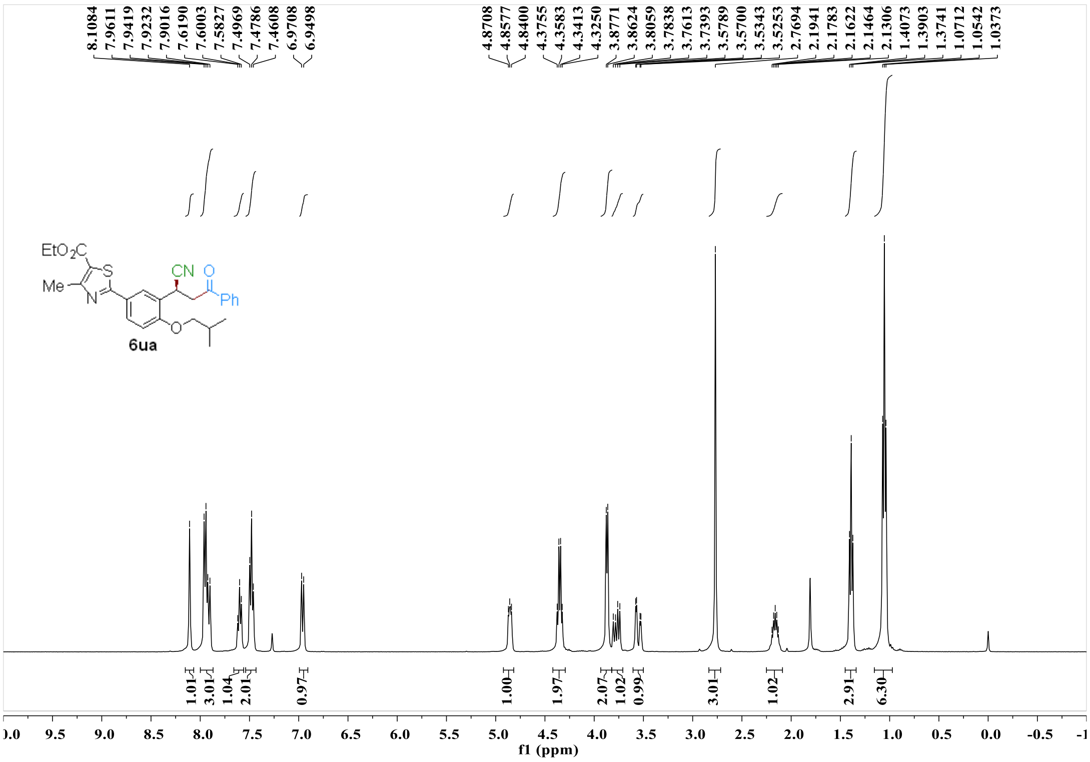

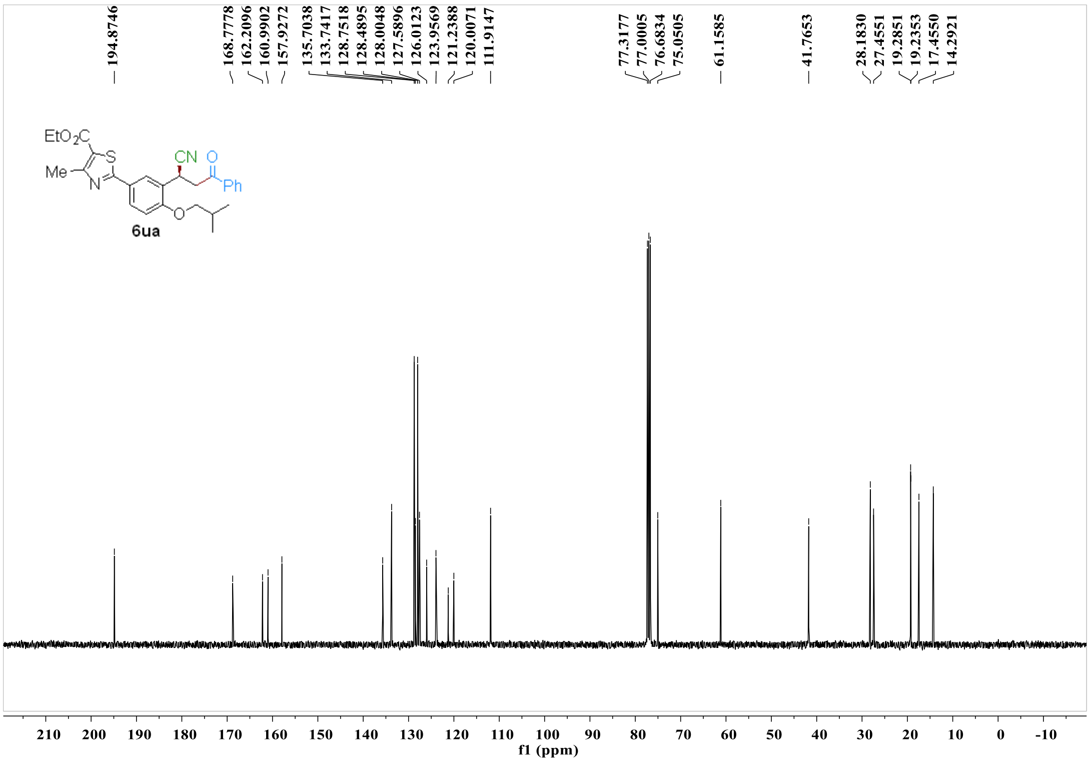

^{1}H NMR (400 MHz, CDCl3) and ^{13}C NMR (100 MHz, CDCl3) spectra of substrate 6va

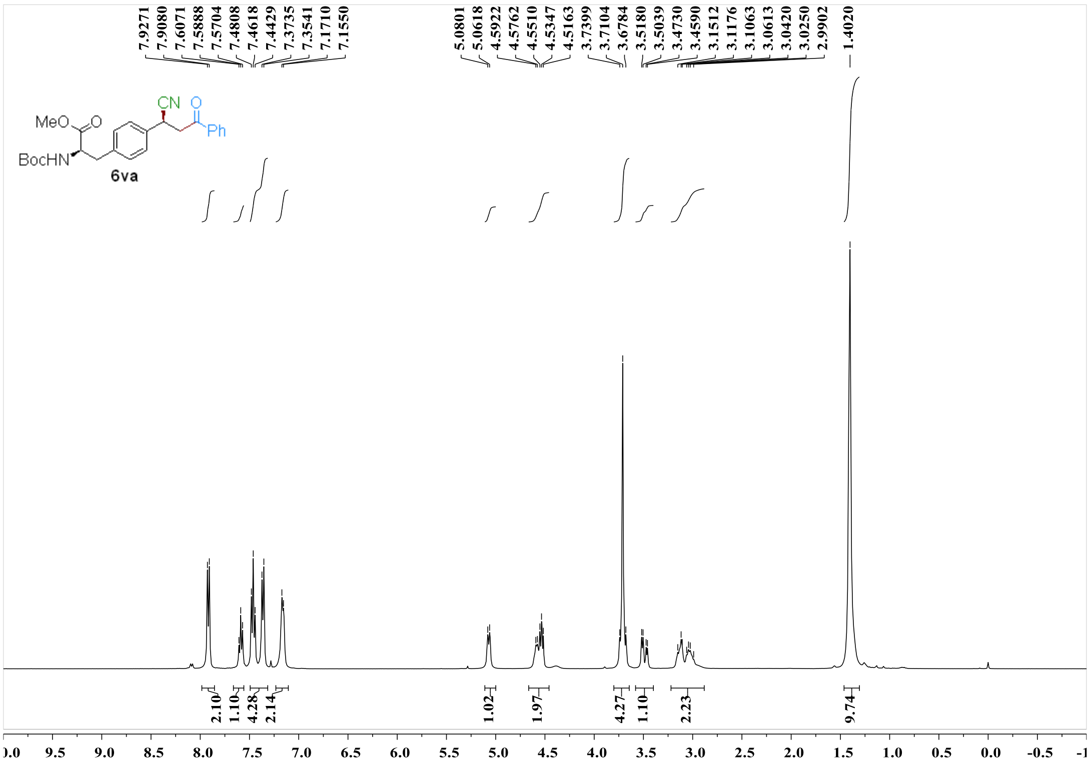

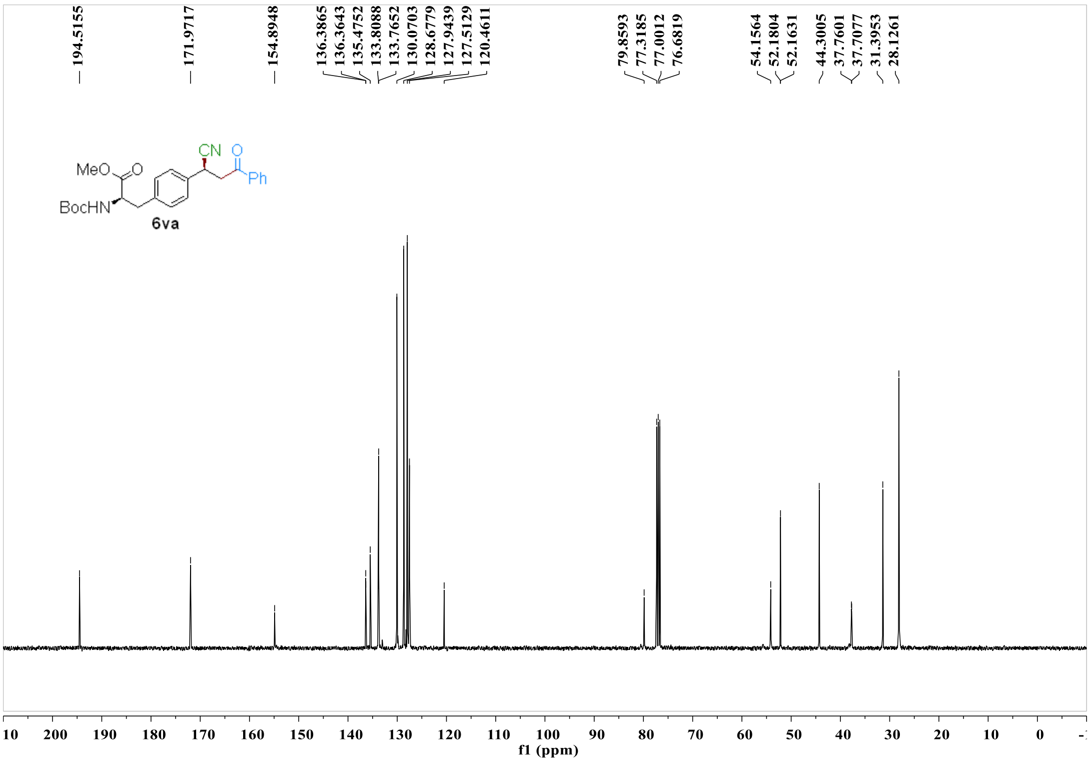

^{1}H NMR (400 MHz, $CDCl_3$) and ^{13}C NMR (100 MHz, $CDCl_3$) spectra of substrate 6bb

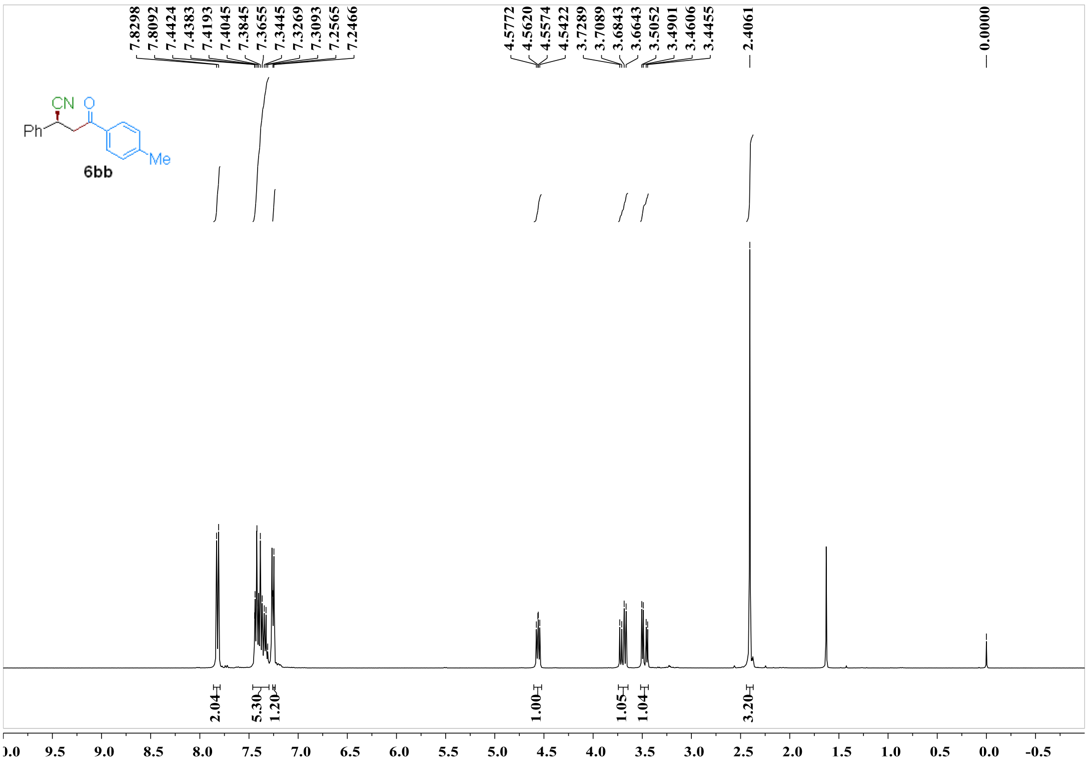

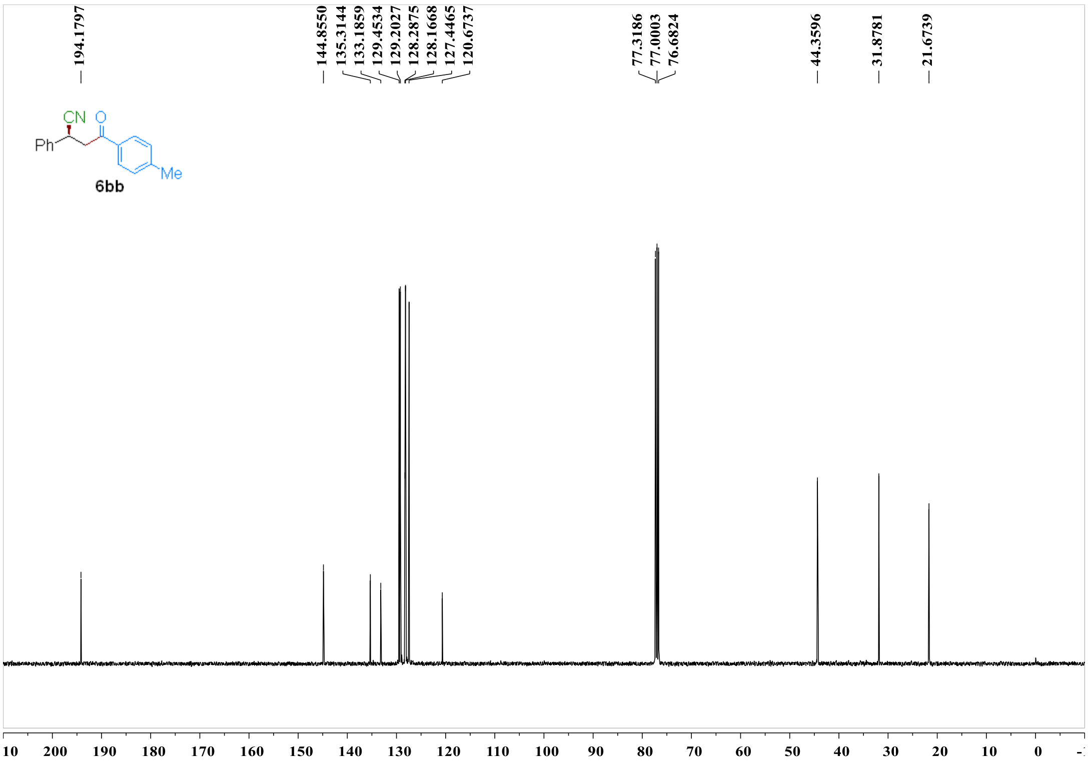

^{1}H NMR (400 MHz, CDCl3) and ^{13}C NMR (100 MHz, CDCl3) spectra of substrate 6bc

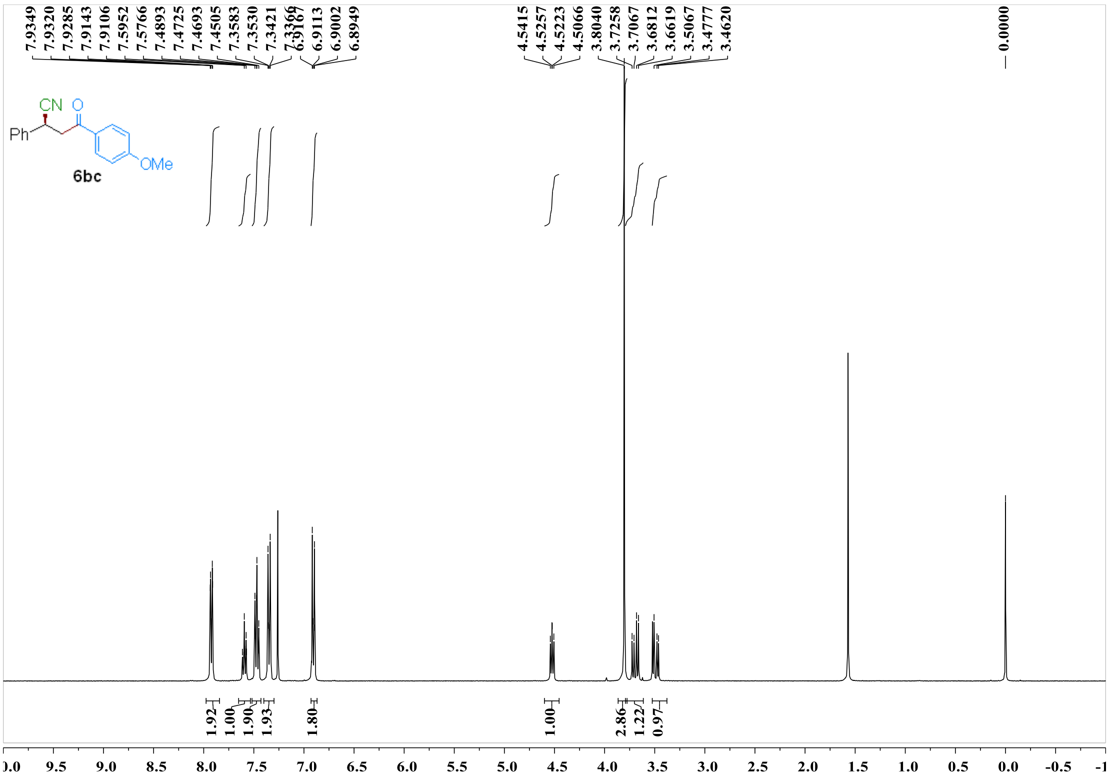

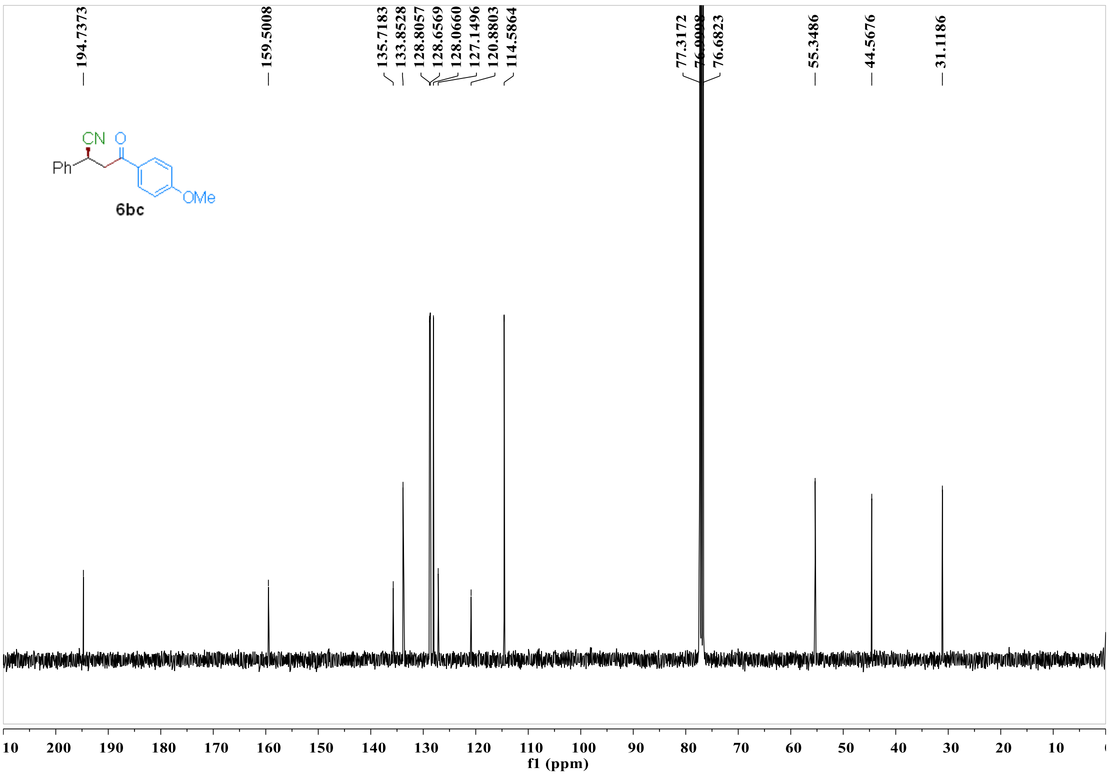

^{1}H NMR (400 MHz, $CDCl_3$) and ^{13}C NMR (100 MHz, $CDCl_3$) spectra of substrate 6bd

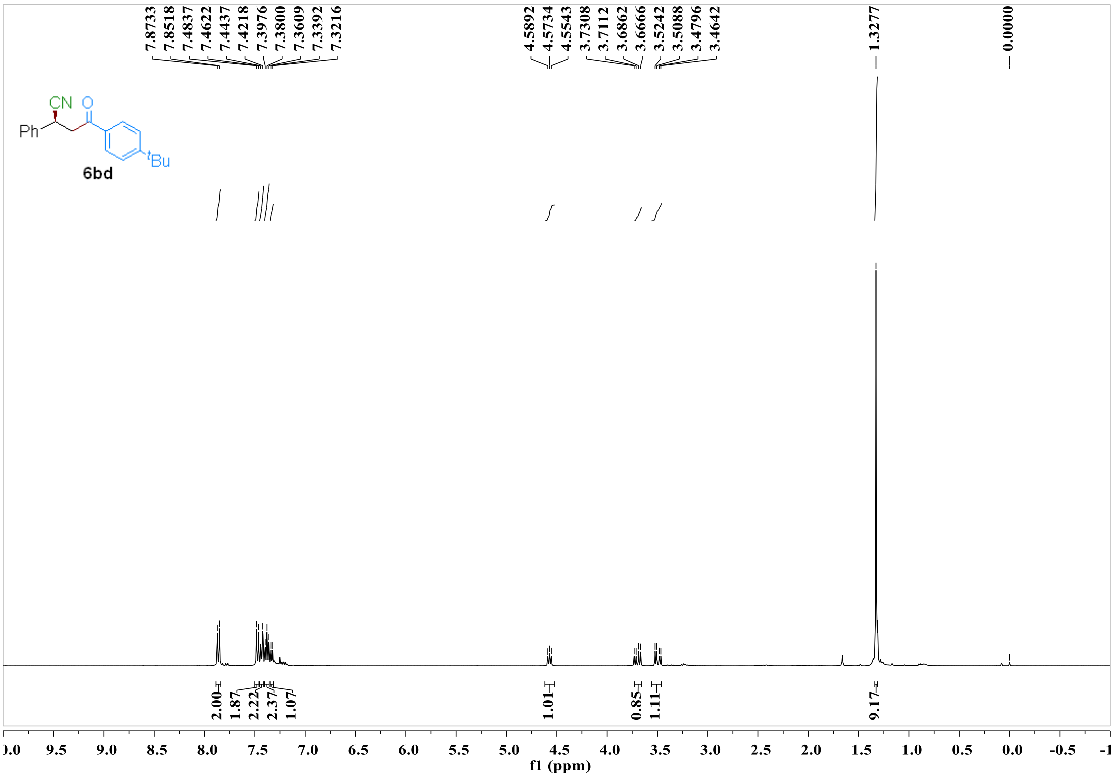

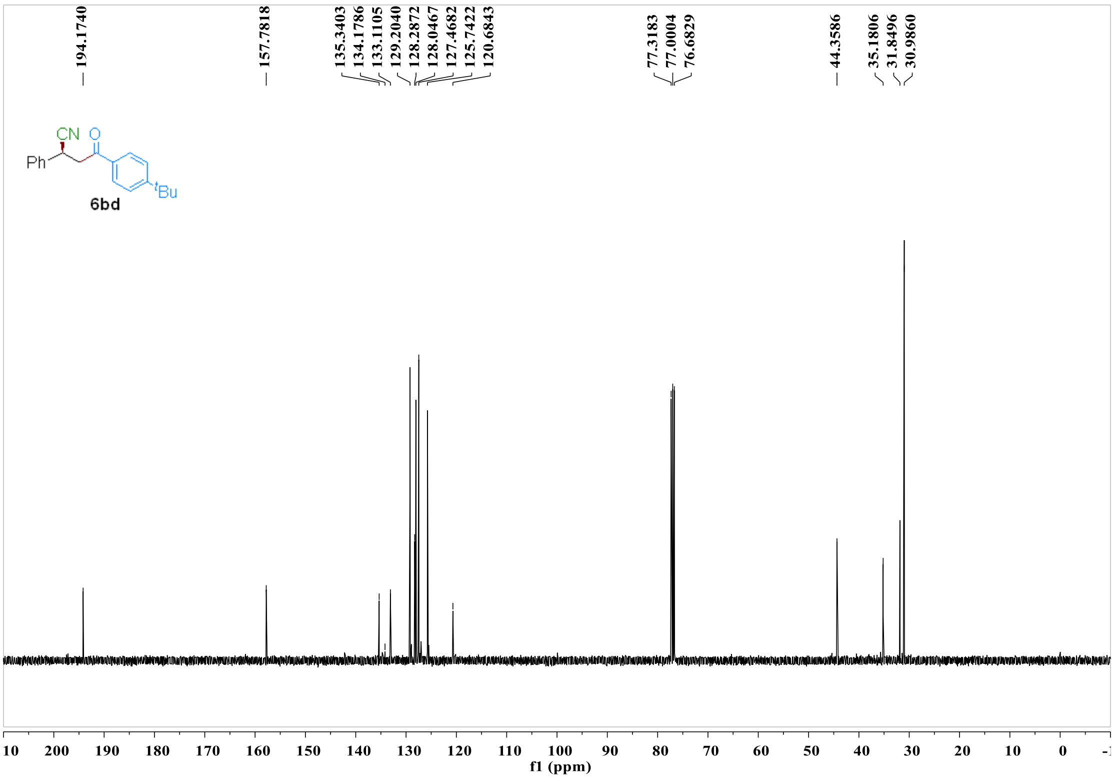

^{1}H NMR (400 MHz, $CDCl_3$), ^{13}C NMR (100 MHz, $CDCl_3$), ^{19}F NMR (376 MHz, $CDCl_3$) spectra of substrate 6be

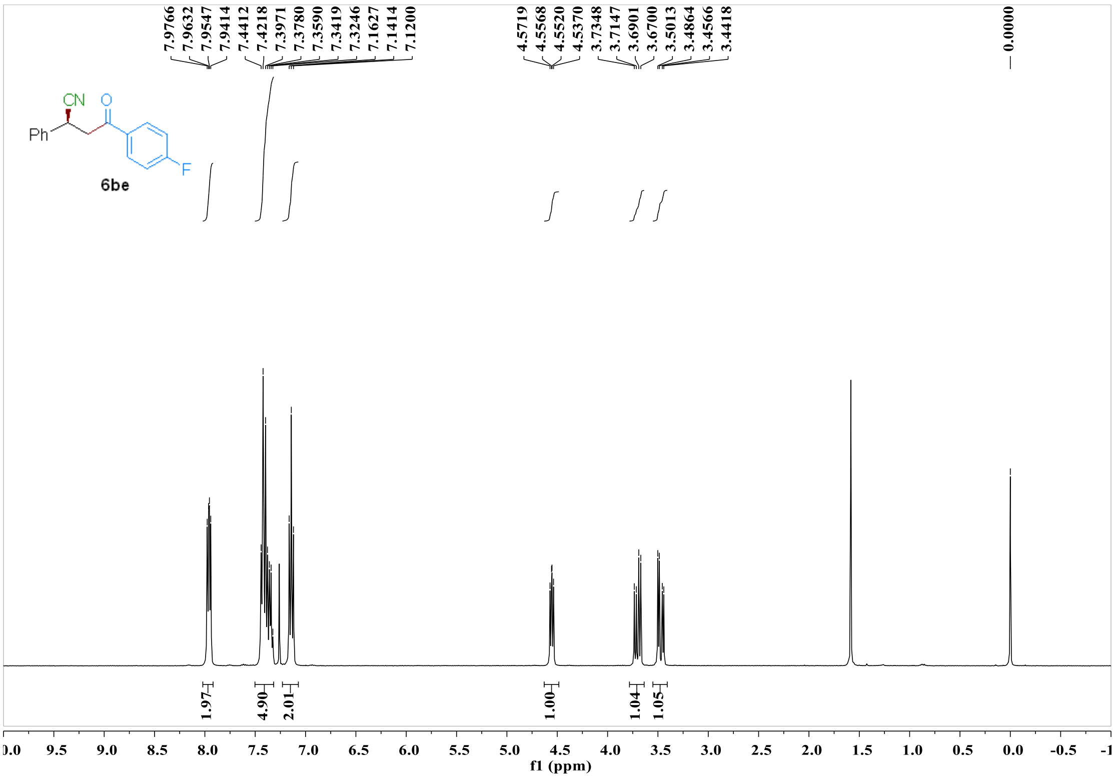

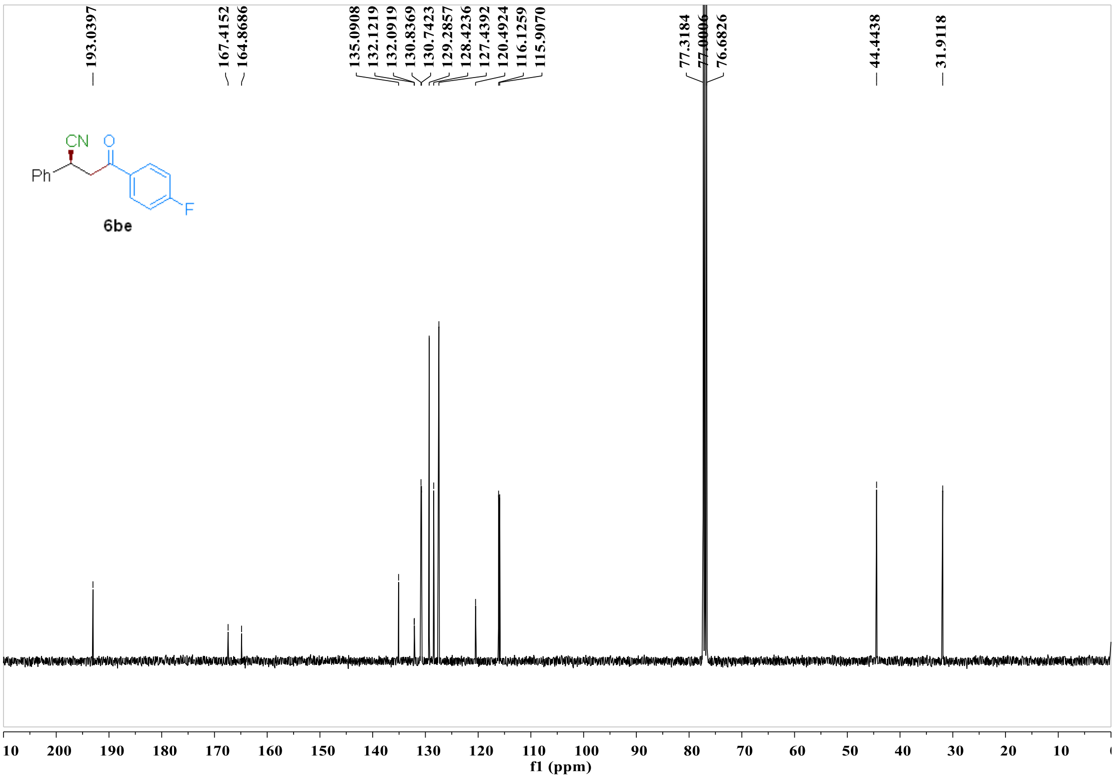

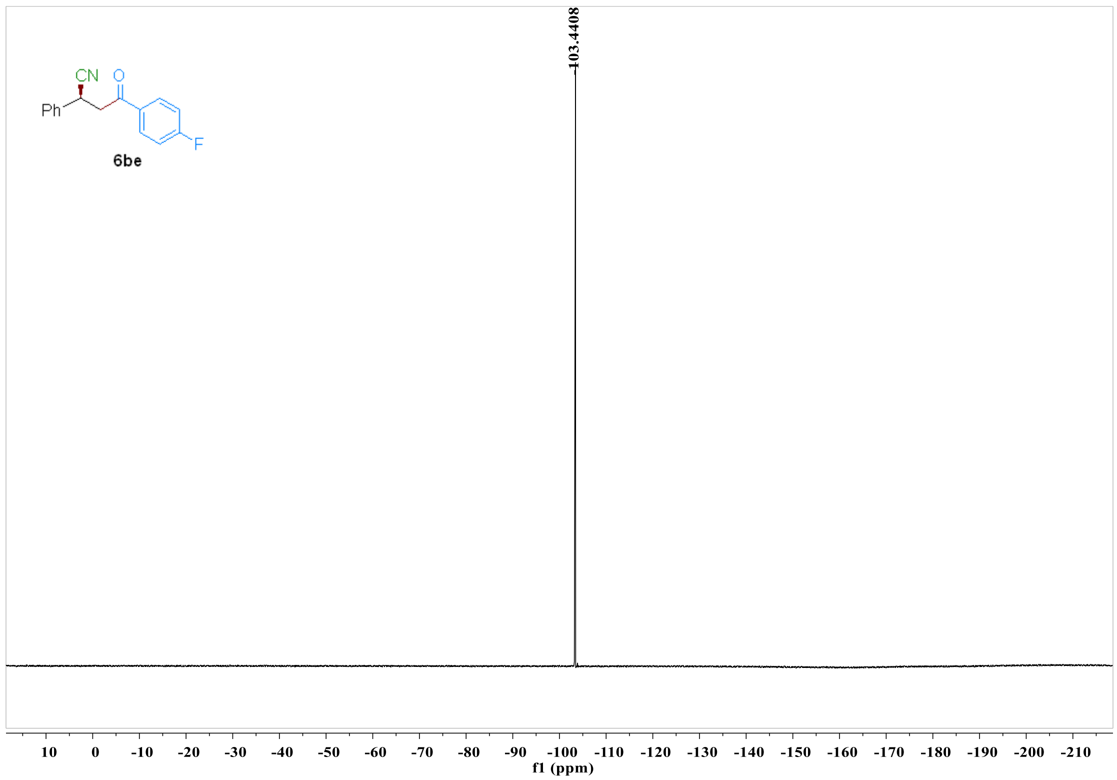
-103.4408
CN
O
Ph
F
6be
10
0
-10
-20
-30
-40
-50
-60
-70
-80
-90
-100
-110
-120
-130
-140
-150
-160
-170
-180
-190
-200
-210
f1 (ppm)

^{1}H NMR (400 MHz, $CDCl_3$) and ^{13}C NMR (100 MHz, $CDCl_3$) spectra of substrate 6bf

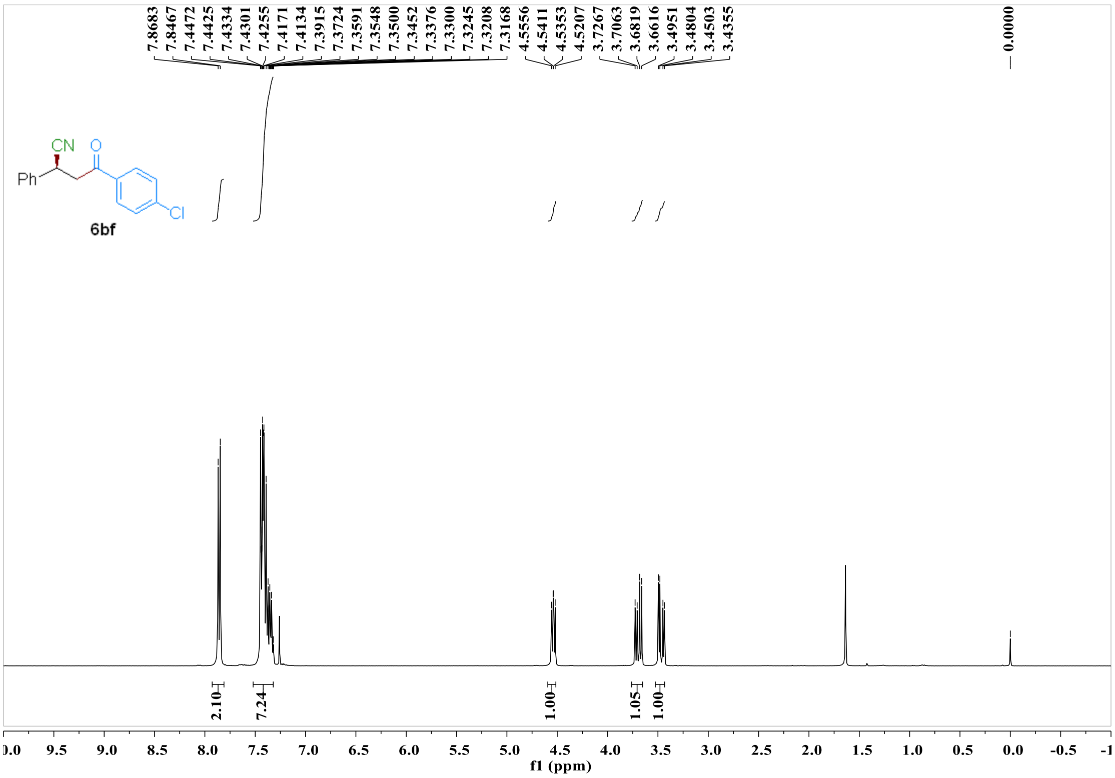

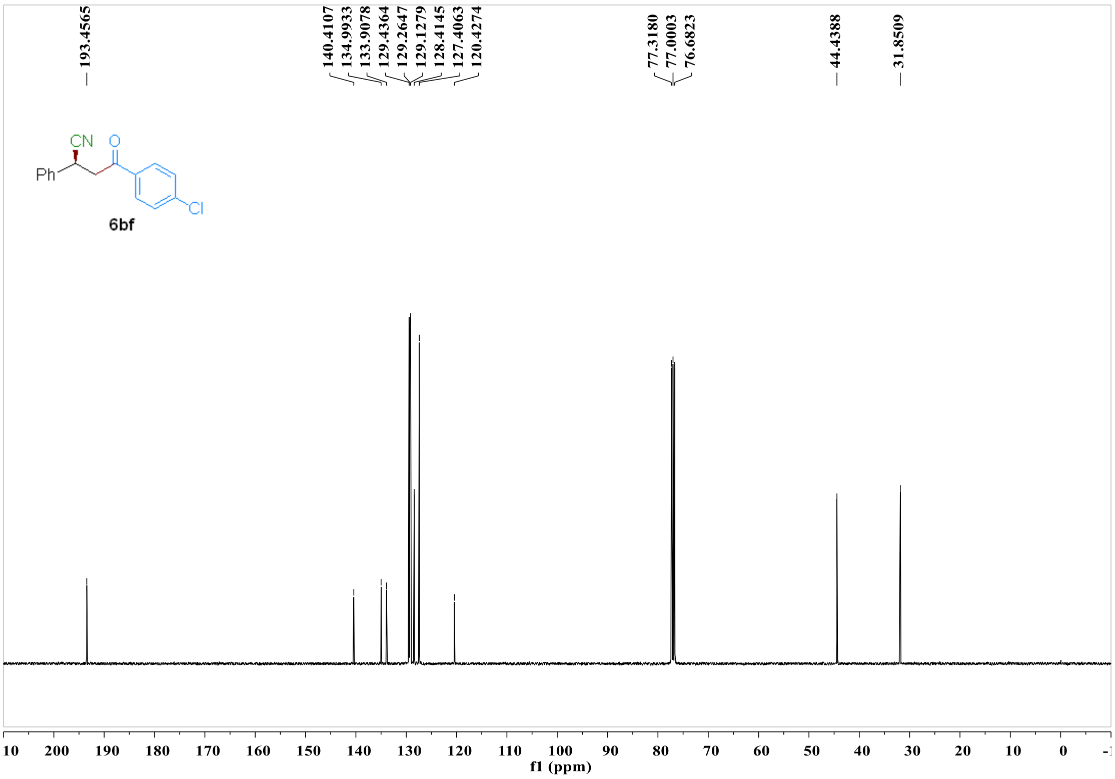

^{1}H NMR (400 MHz, $CDCl_3$) and ^{13}C NMR (100 MHz, $CDCl_3$) spectra of substrate 6bg

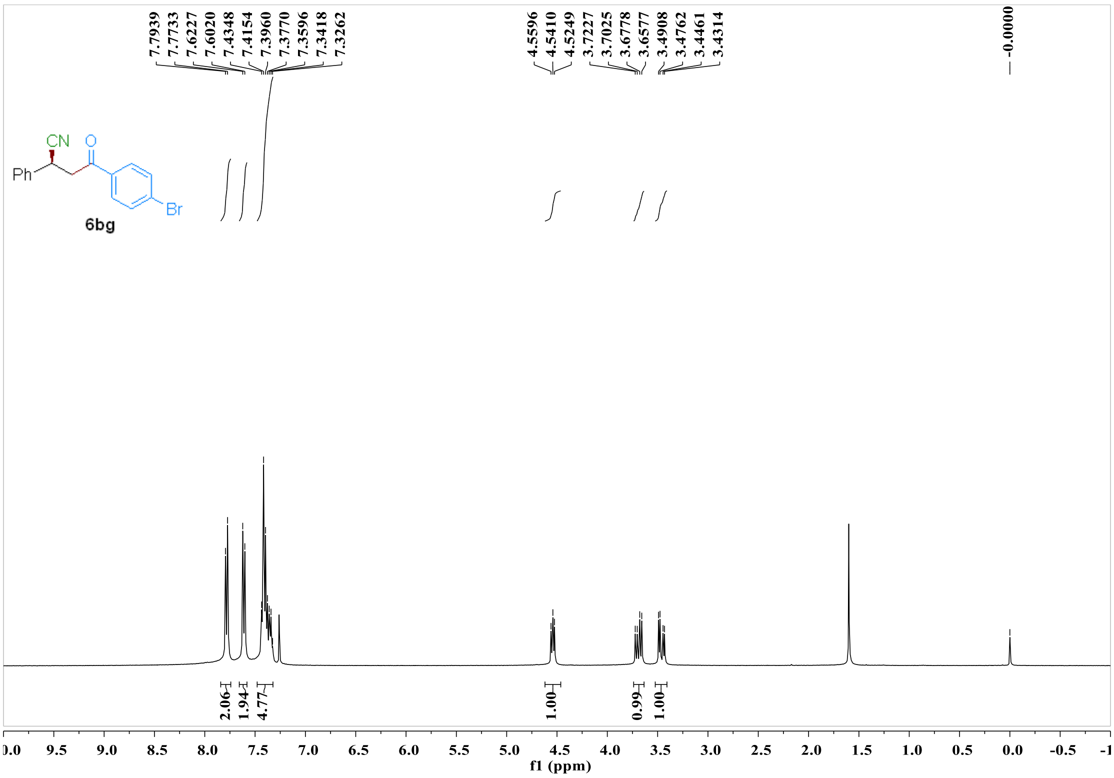

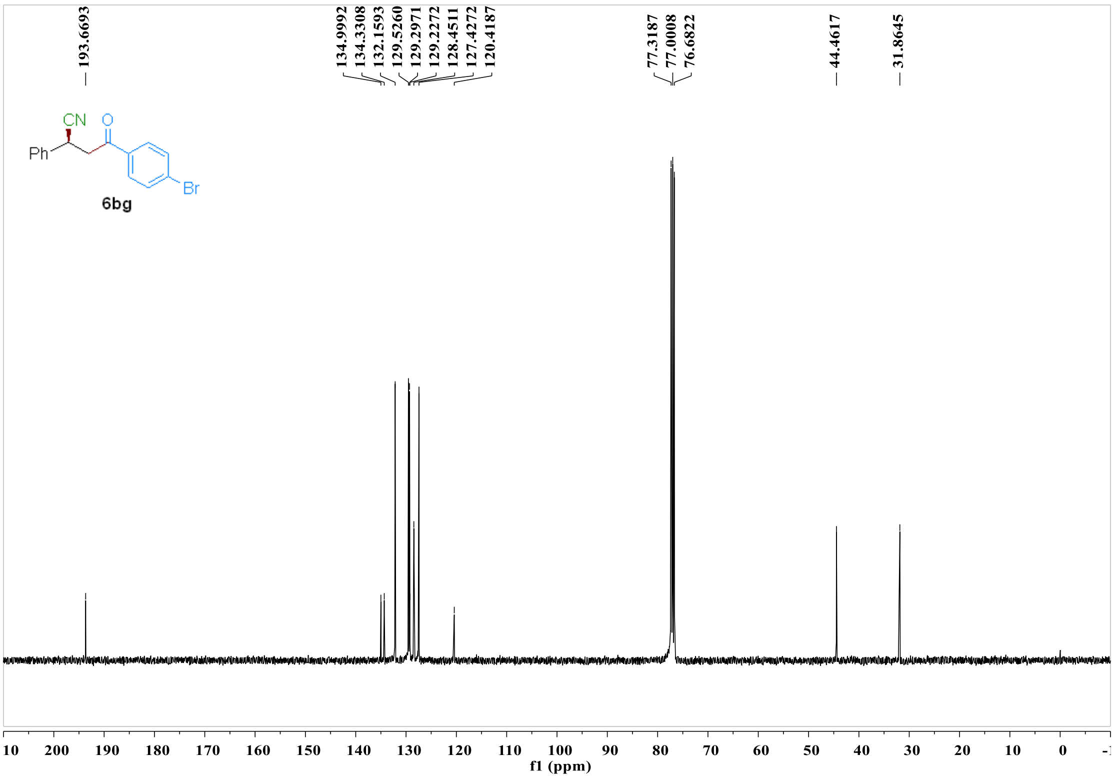

^{1}H NMR (400 MHz, CDCl3), ^{13}C NMR (100 MHz, CDCl3), ^{19}F NMR (376 MHz, CDCl3) spectra of substrate 6bh

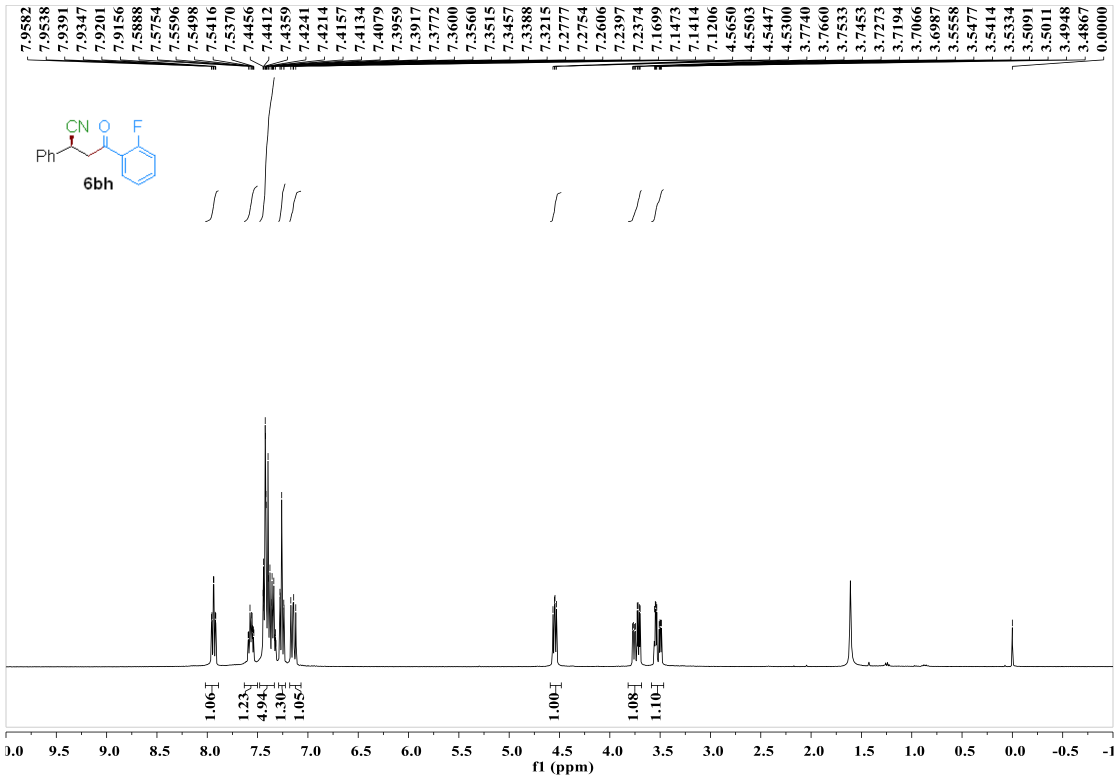

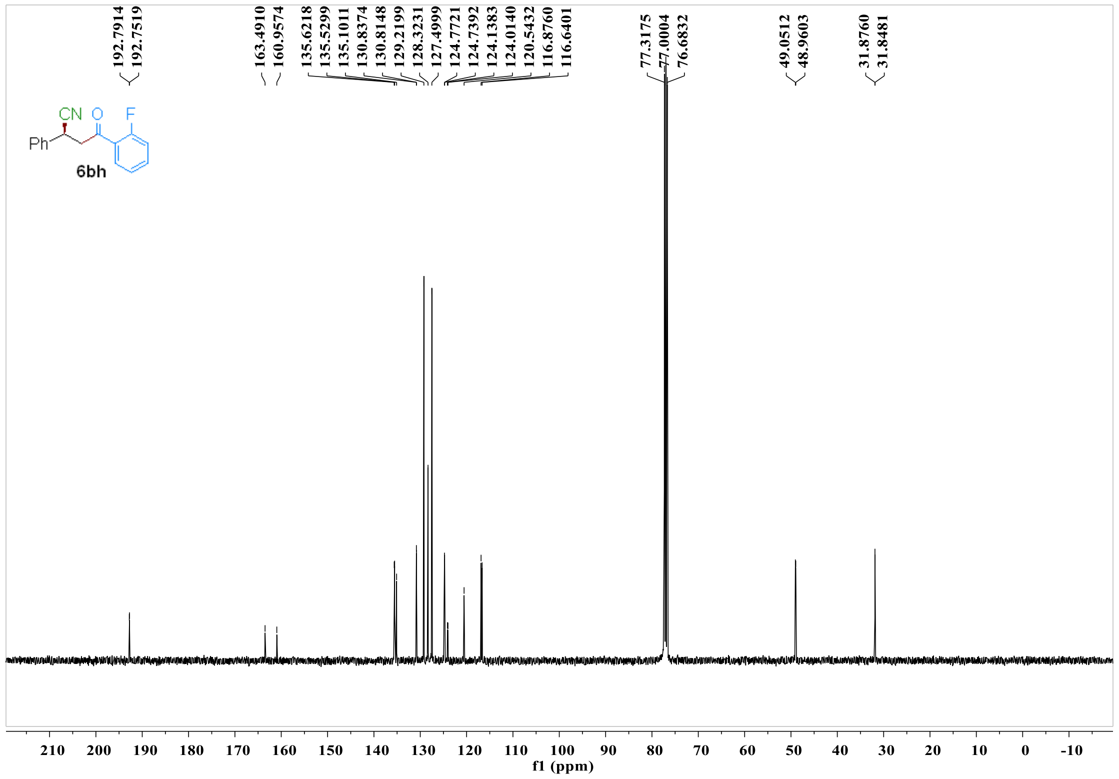

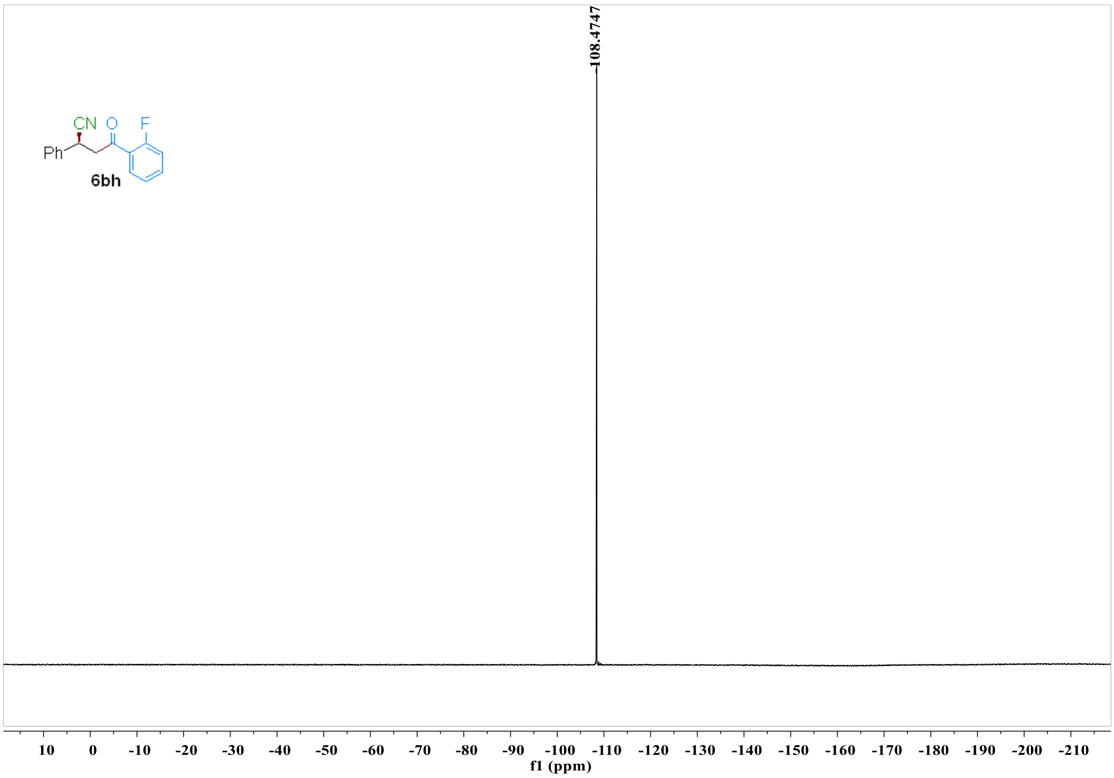
-108.4747
CN
O
F
Ph
6bh
10 0 -10 -20 -30 -40 -50 -60 -70 -80 -90 -100 -110 -120 -130 -140 -150 -160 -170 -180 -190 -200 -210
f1 (ppm)

^{1}H NMR (400 MHz, CDCl3) and ^{13}C NMR (100 MHz, CDCl3) spectra of substrate 6bi

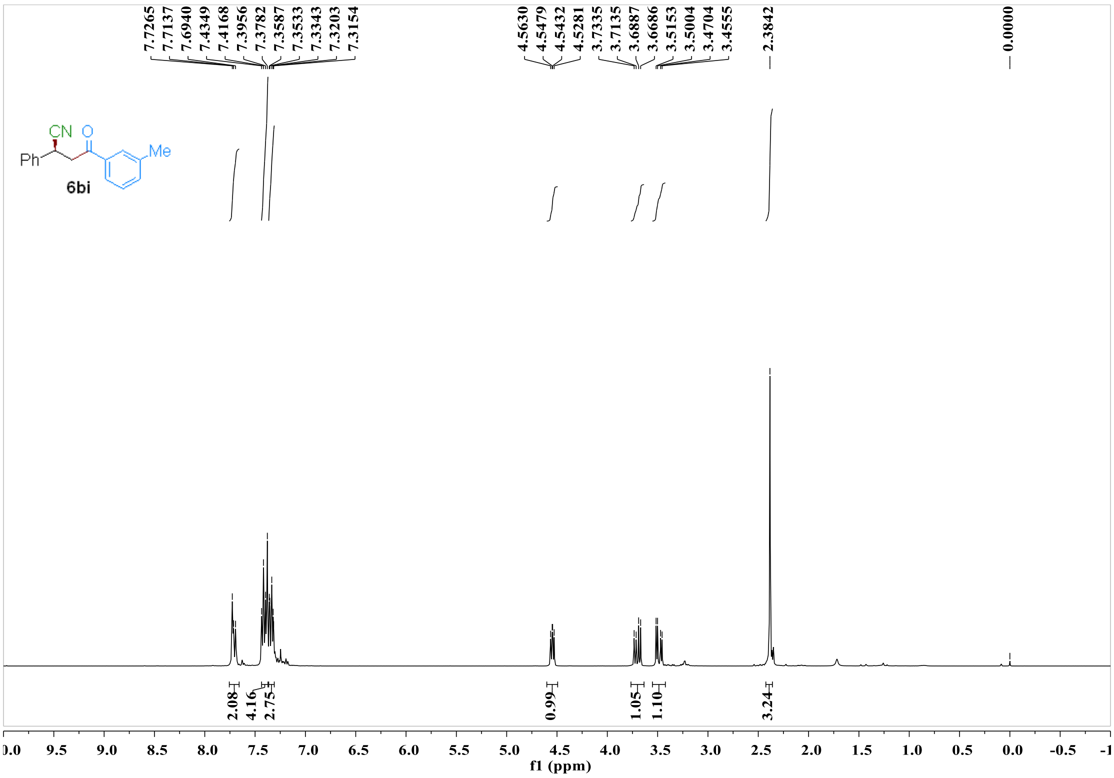

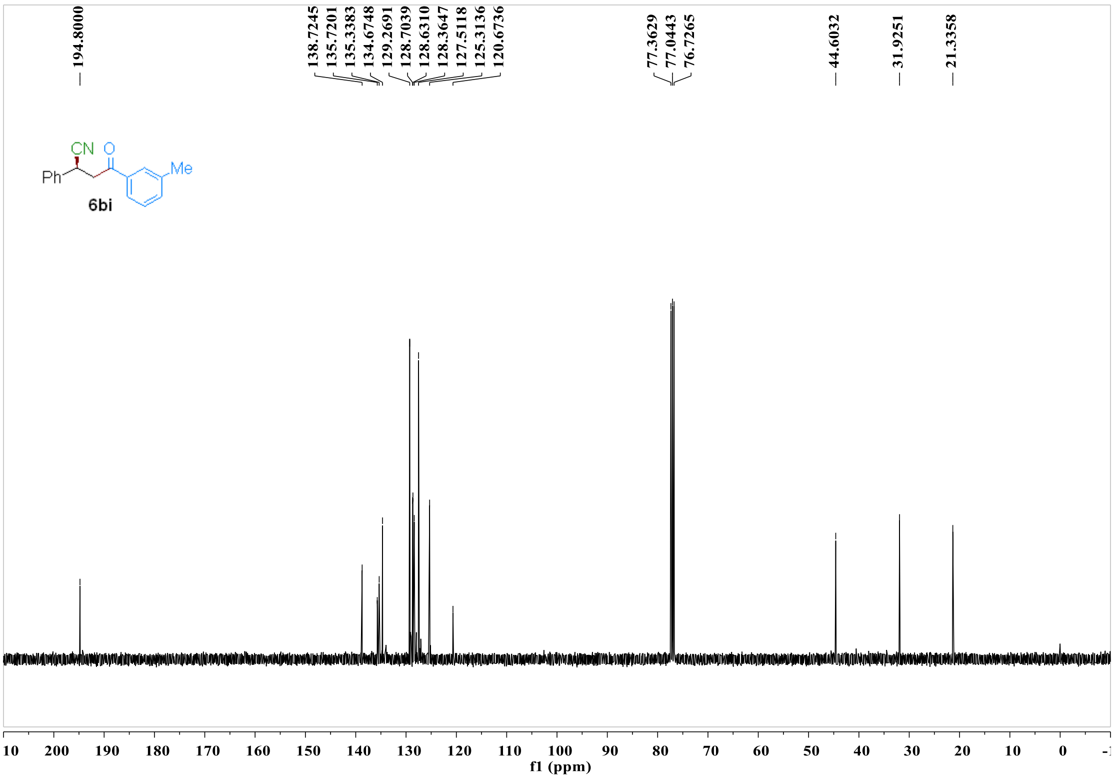

^{1}H NMR (400 MHz, $CDCl_3$) and ^{13}C NMR (100 MHz, $CDCl_3$) spectra of substrate 6bj

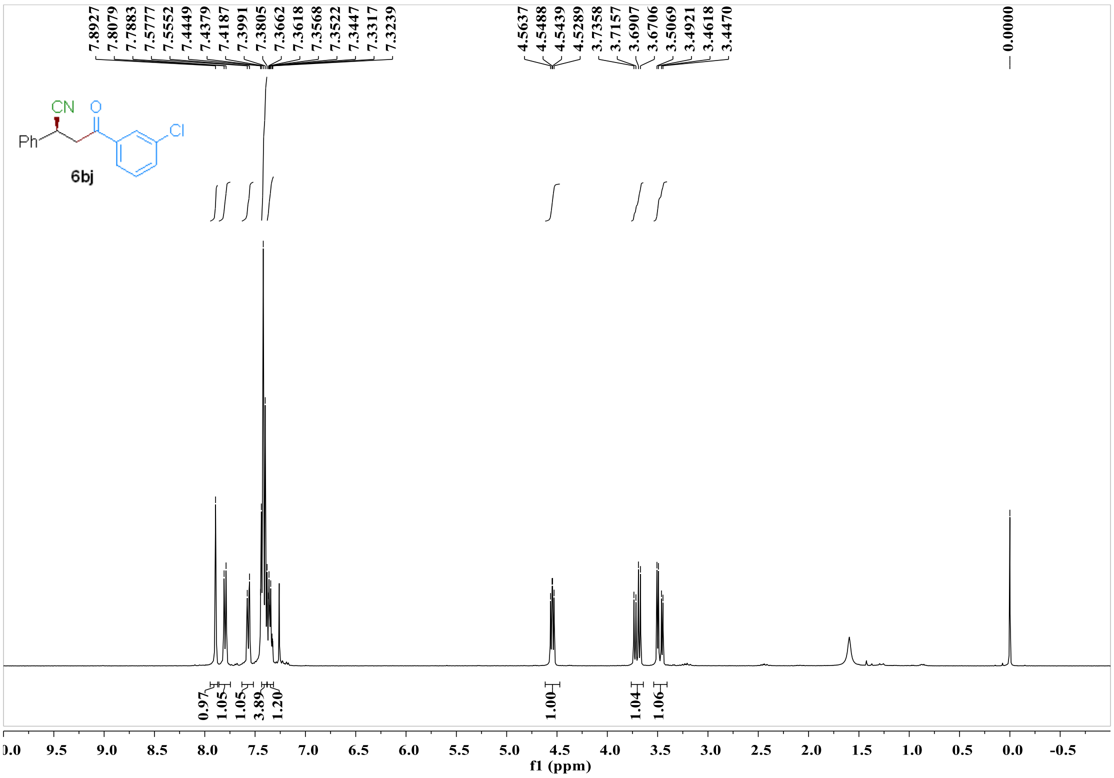

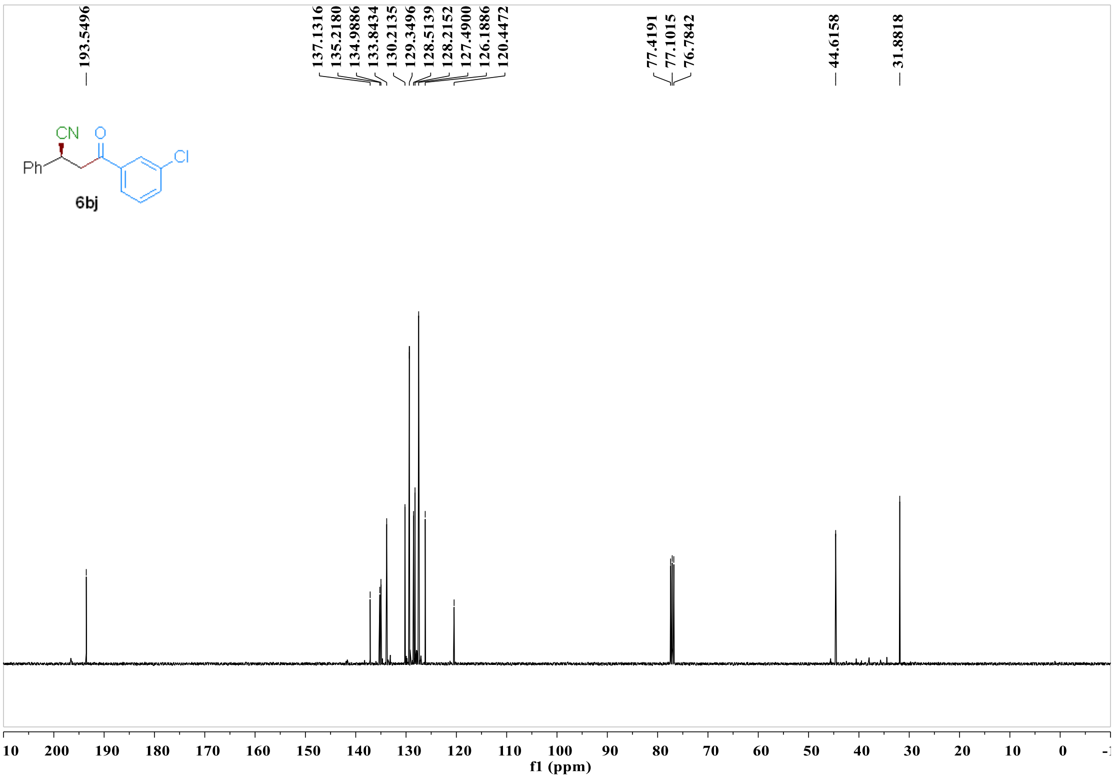

^{1}H NMR (400 MHz, $CDCl_3$) and ^{13}C NMR (100 MHz, $CDCl_3$) spectra of substrate 6bk

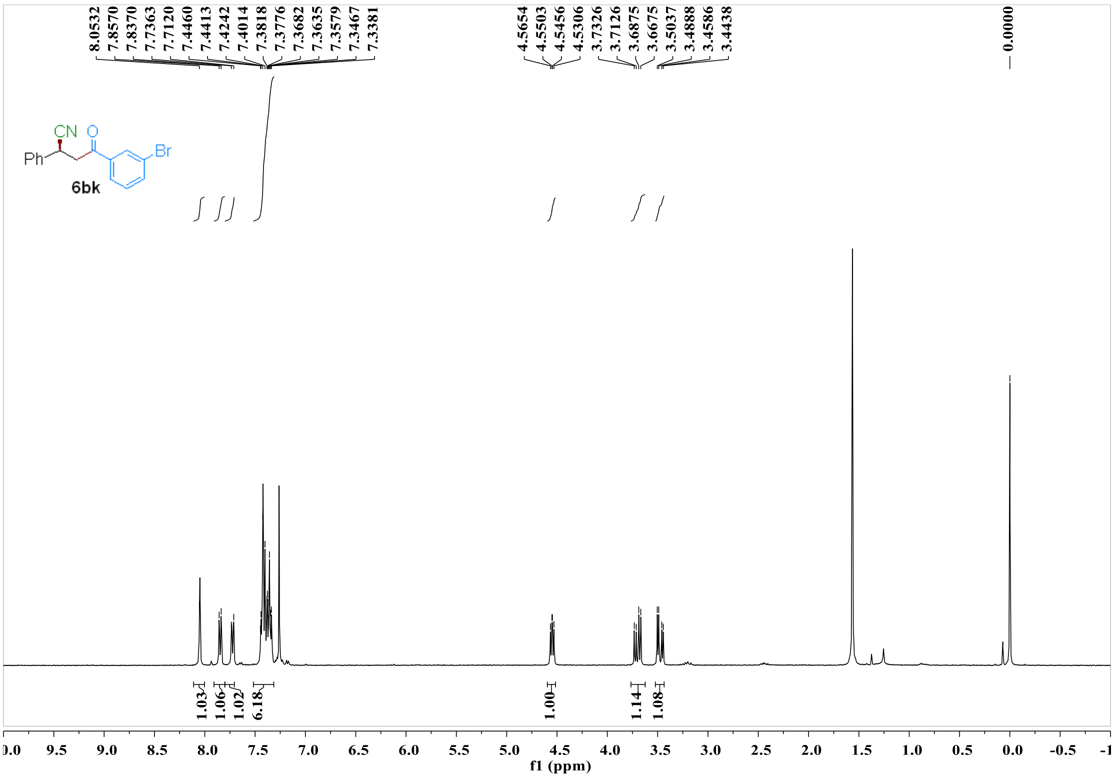

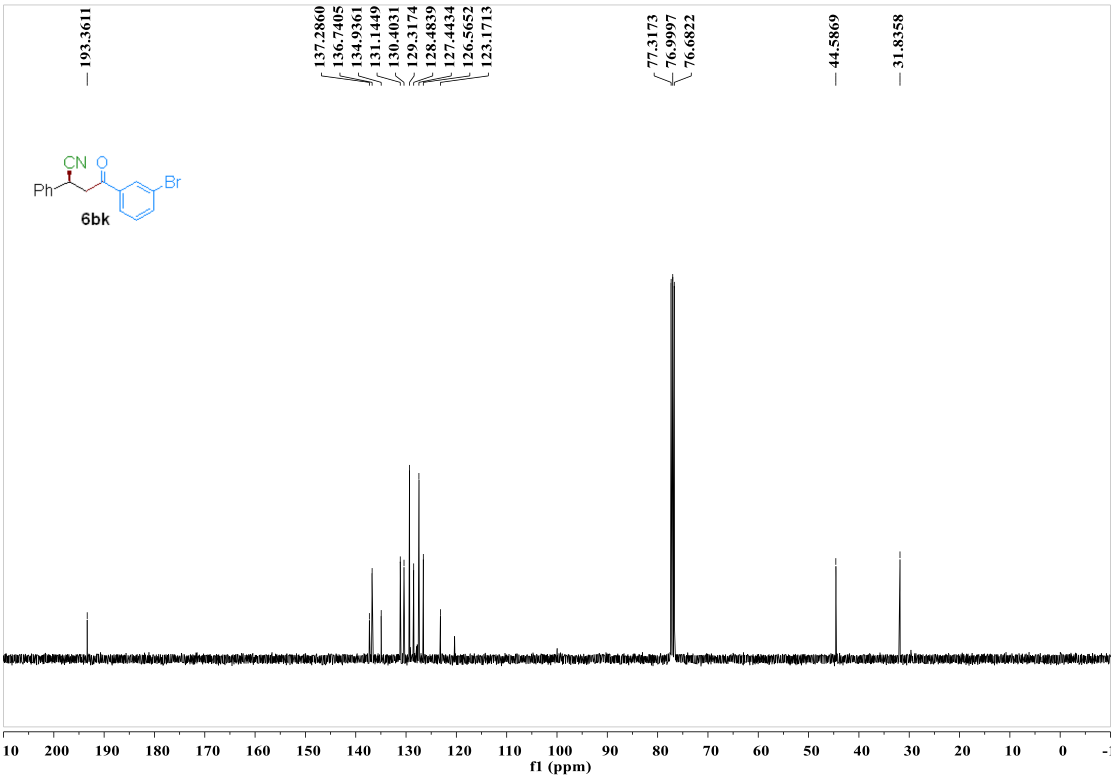

^{1}H NMR (400 MHz, CDCl$_3$) and ^{13}C NMR (100 MHz, CDCl$_3$) spectra of substrate 6bl

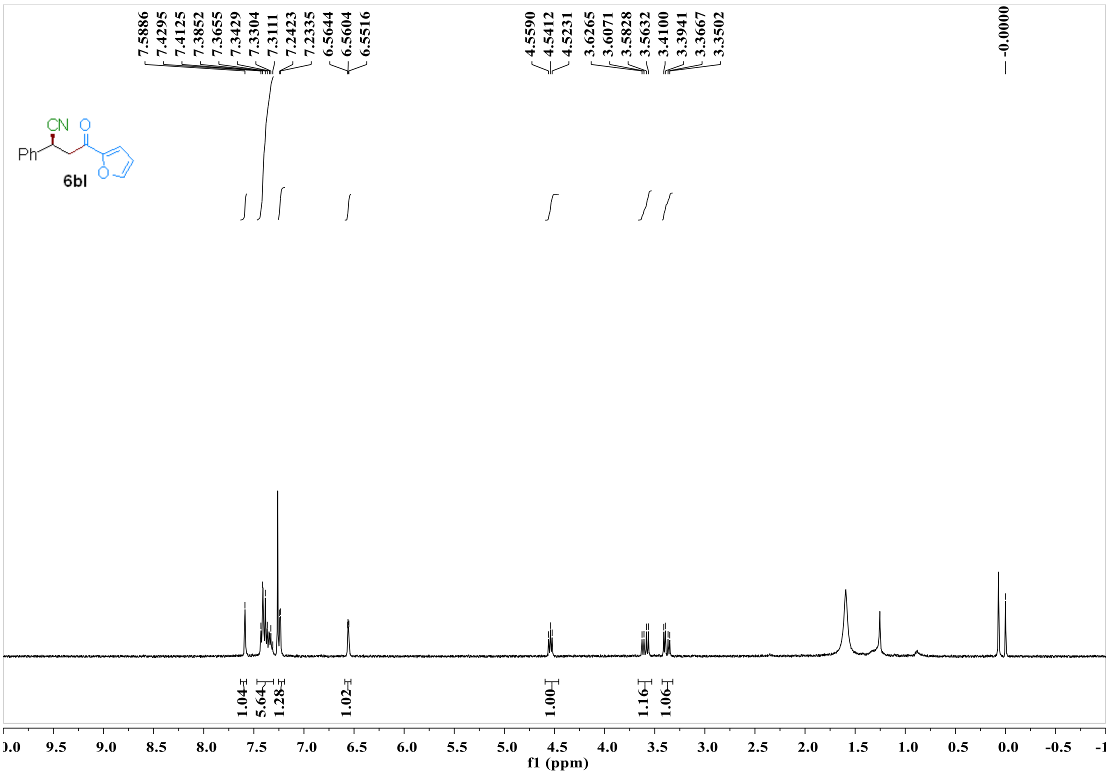

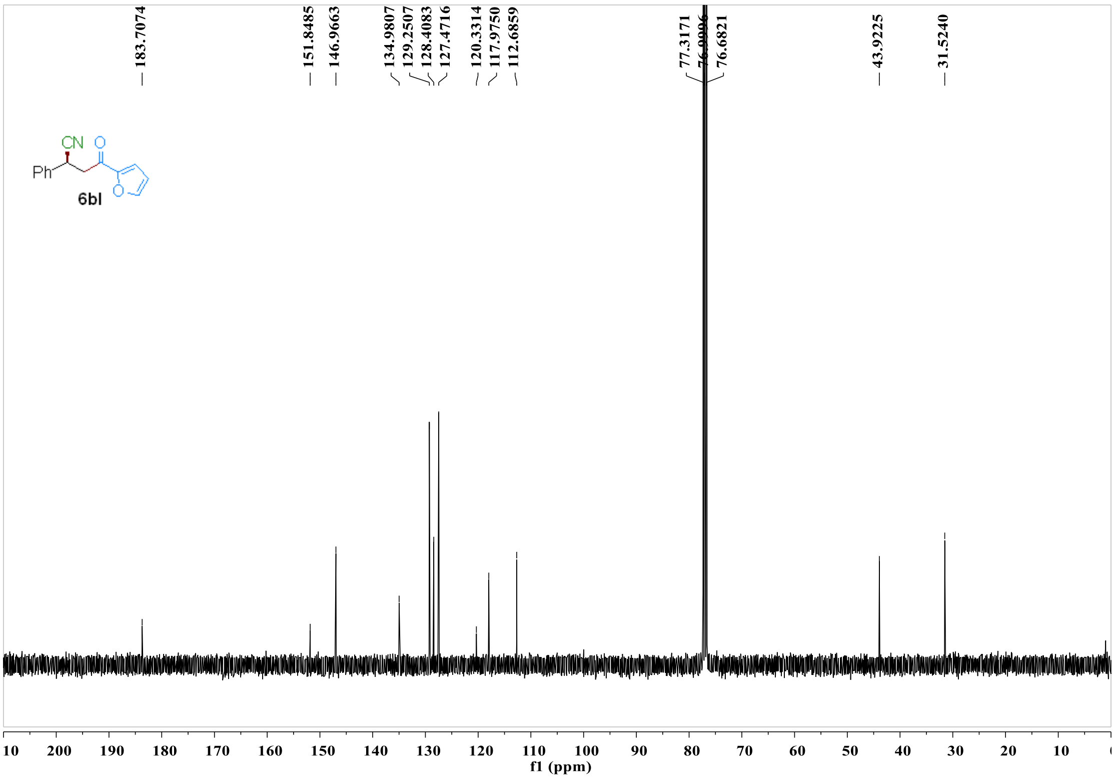

^{1}H NMR (400 MHz, CDCl$_3$) and ^{13}C NMR (100 MHz, CDCl$_3$) spectra of substrate of 6am

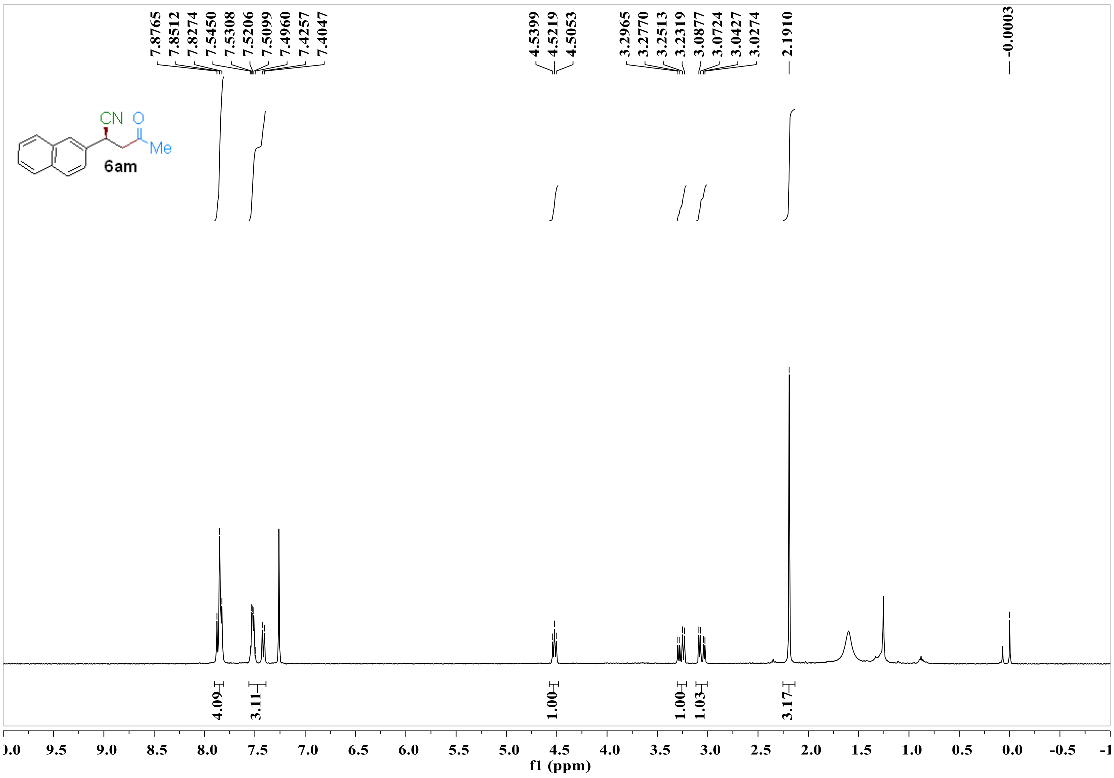

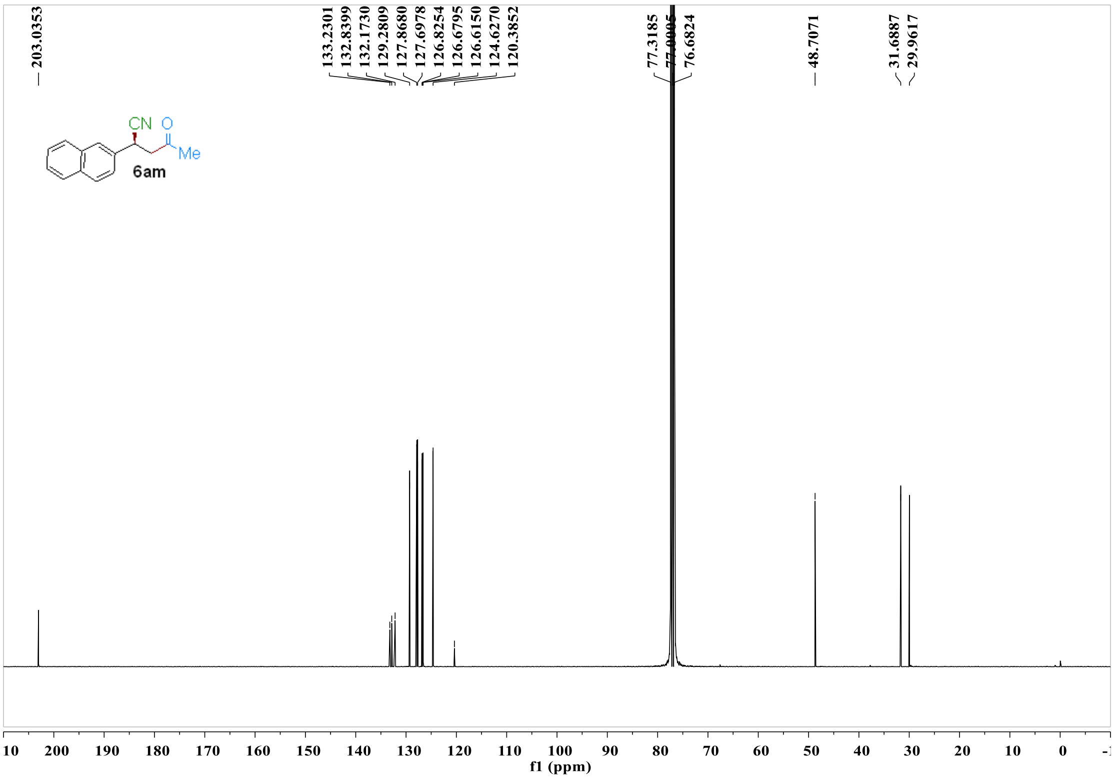

^{1}H NMR (400 MHz, CDCl$_3$) and ^{13}C NMR (100 MHz, CDCl$_3$) spectra of substrate 6an

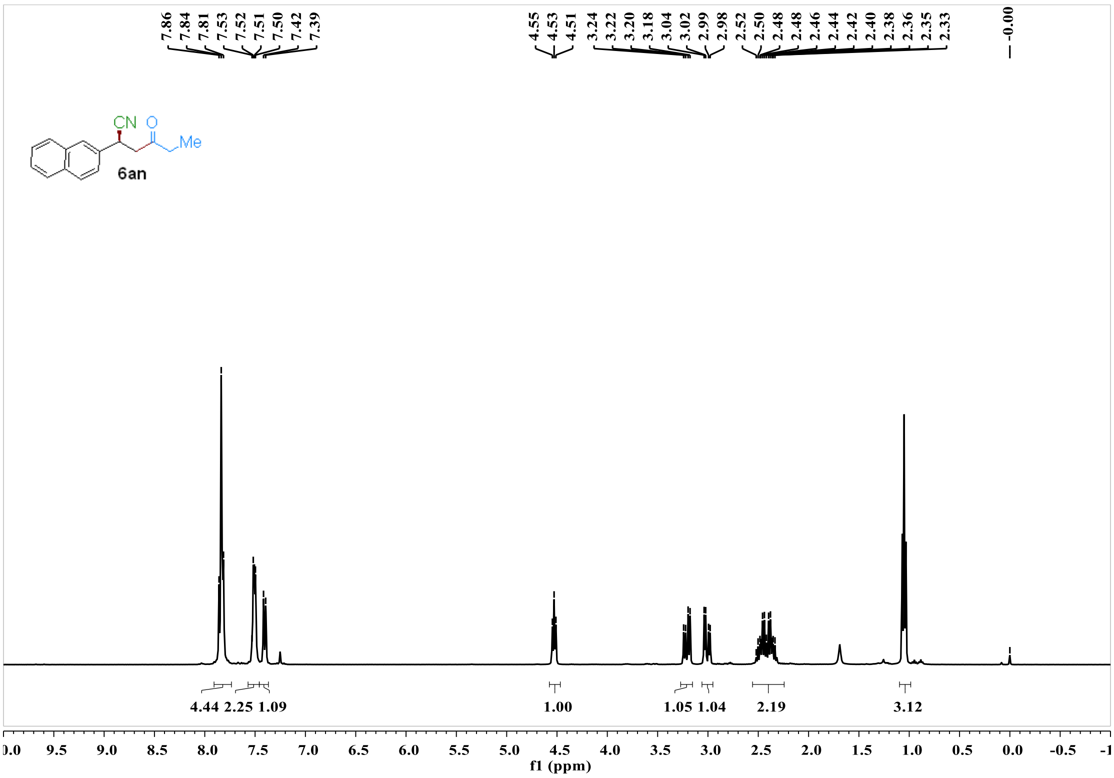

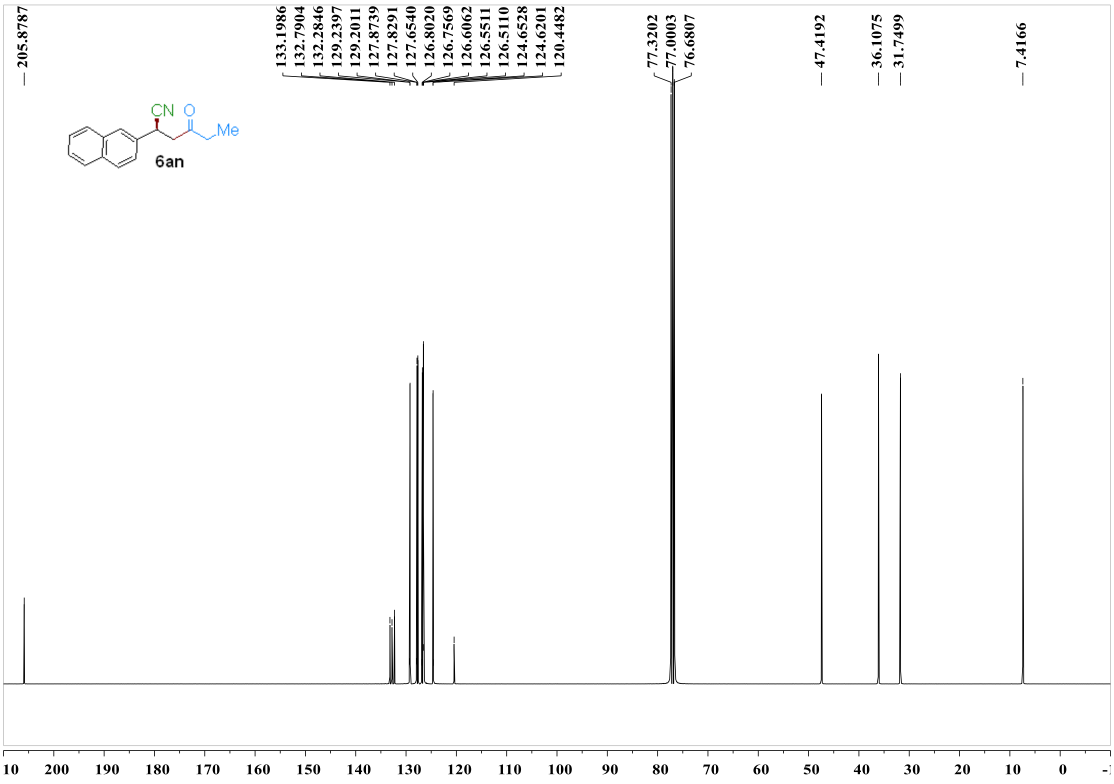

^{1}H NMR (400 MHz, CDCl$_3$) and ^{13}C NMR (100 MHz, CDCl$_3$) spectra of substrate 6ao

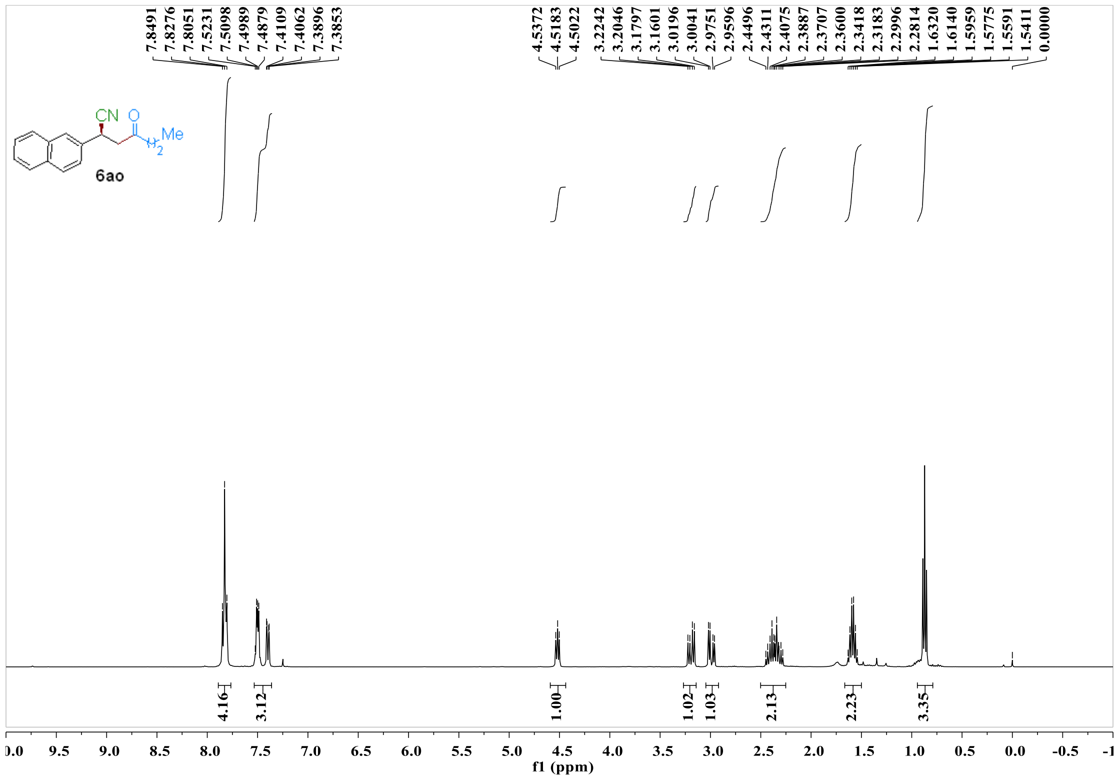

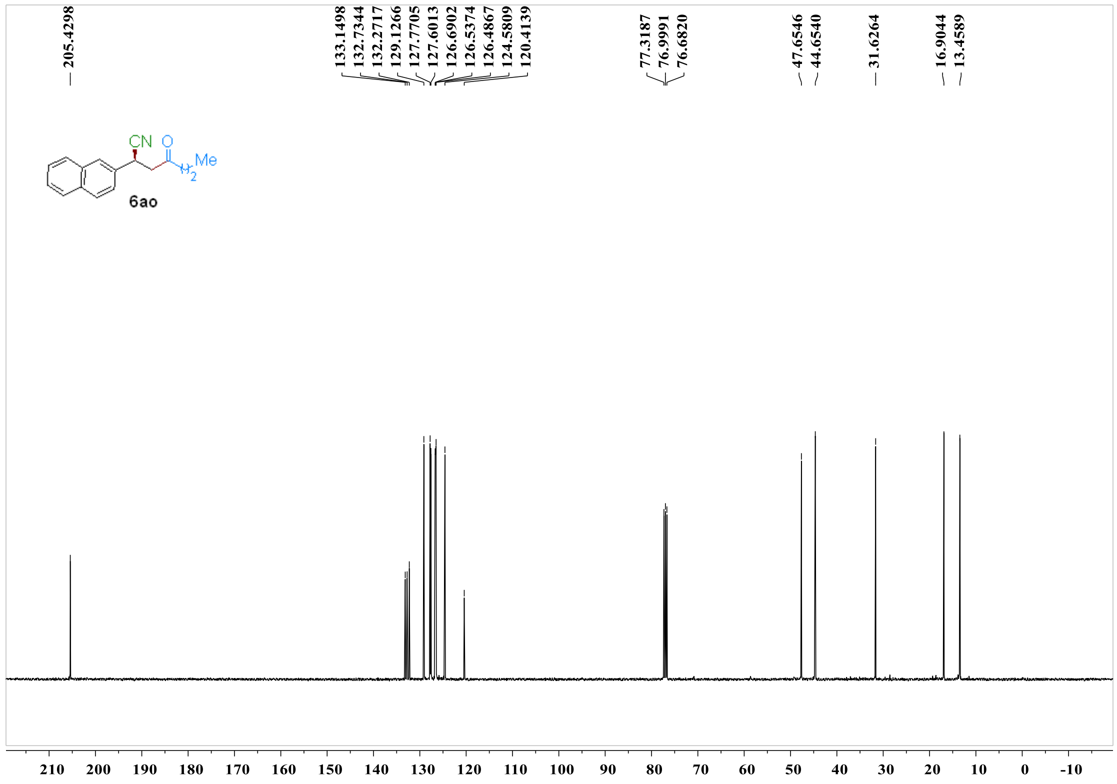

^{1}H NMR (400 MHz, $CDCl_3$) and ^{13}C NMR (100 MHz, $CDCl_3$) spectra of substrate 6ap

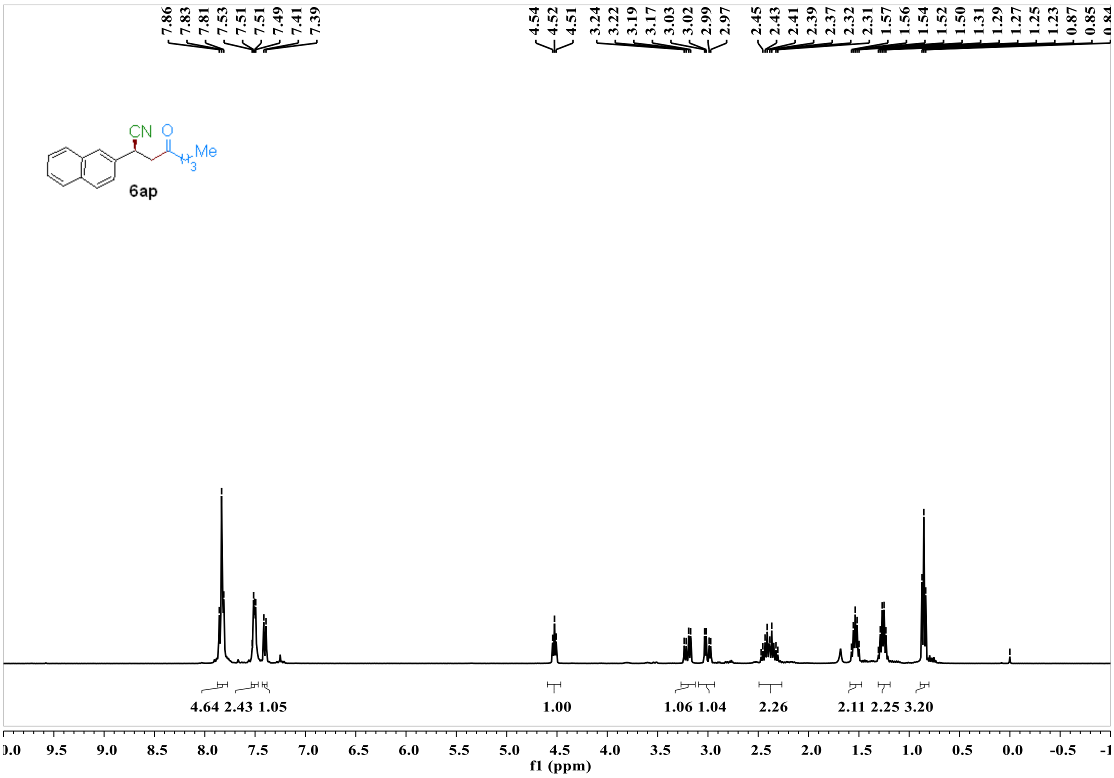

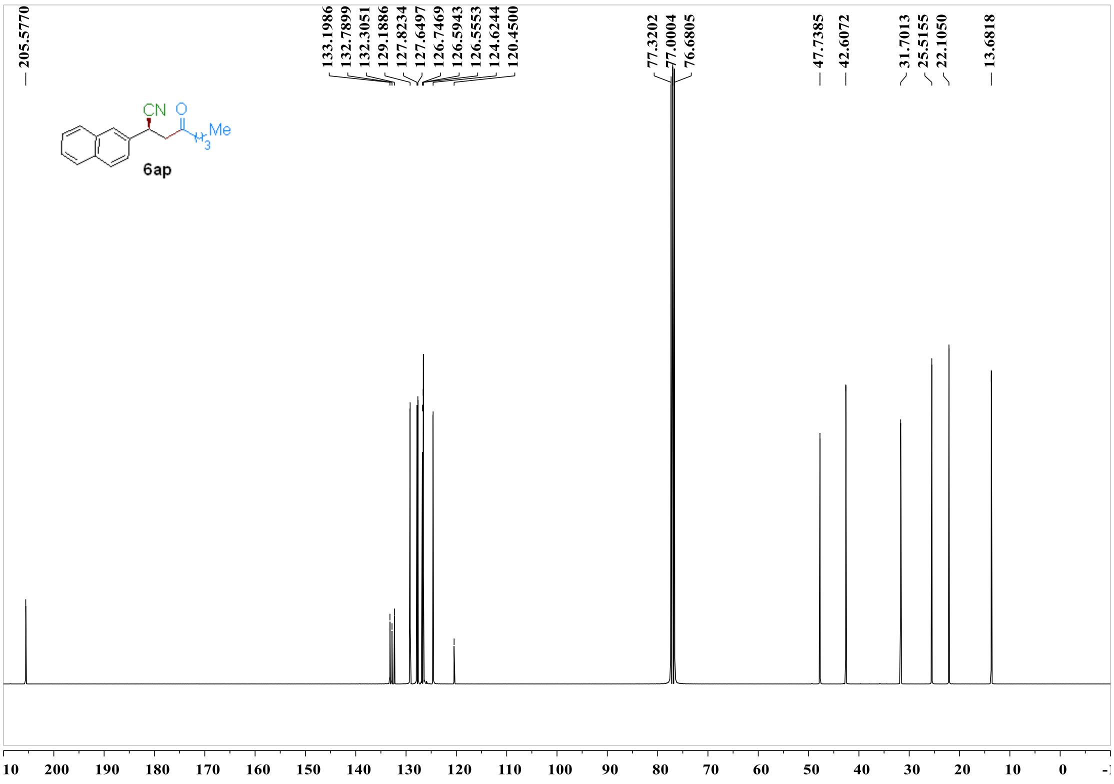

^{1}H NMR (400 MHz, $CDCl_3$) and ^{13}C NMR (100 MHz, $CDCl_3$) spectra of substrate 7aa

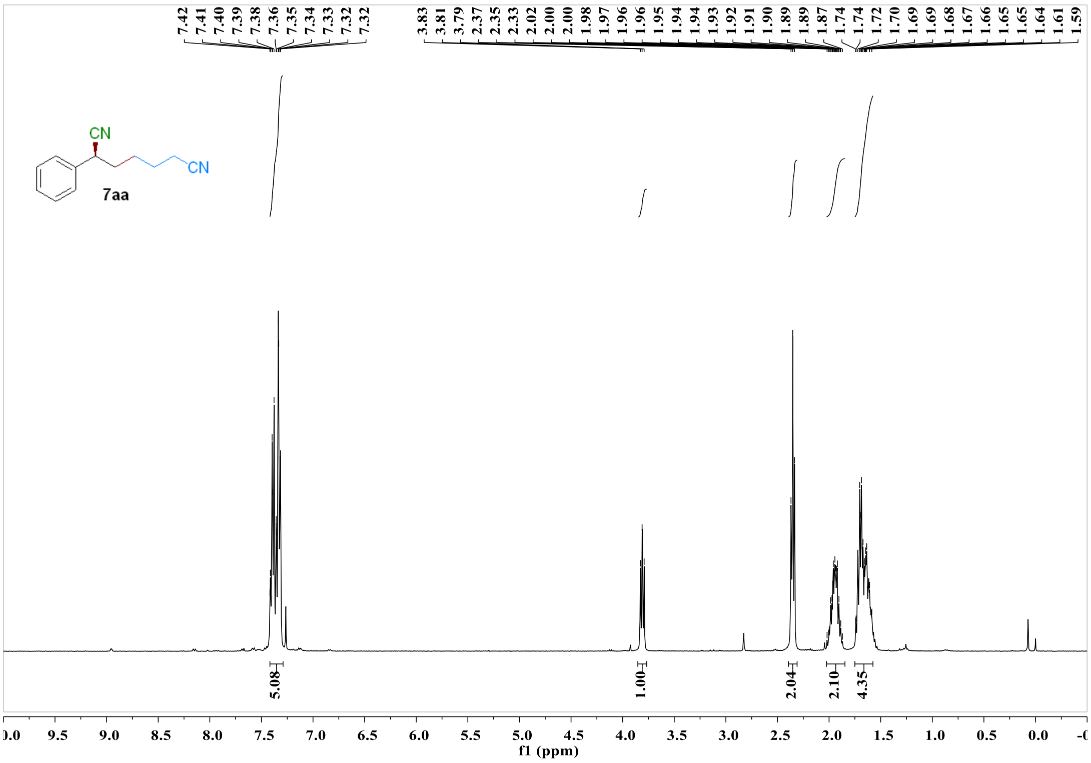

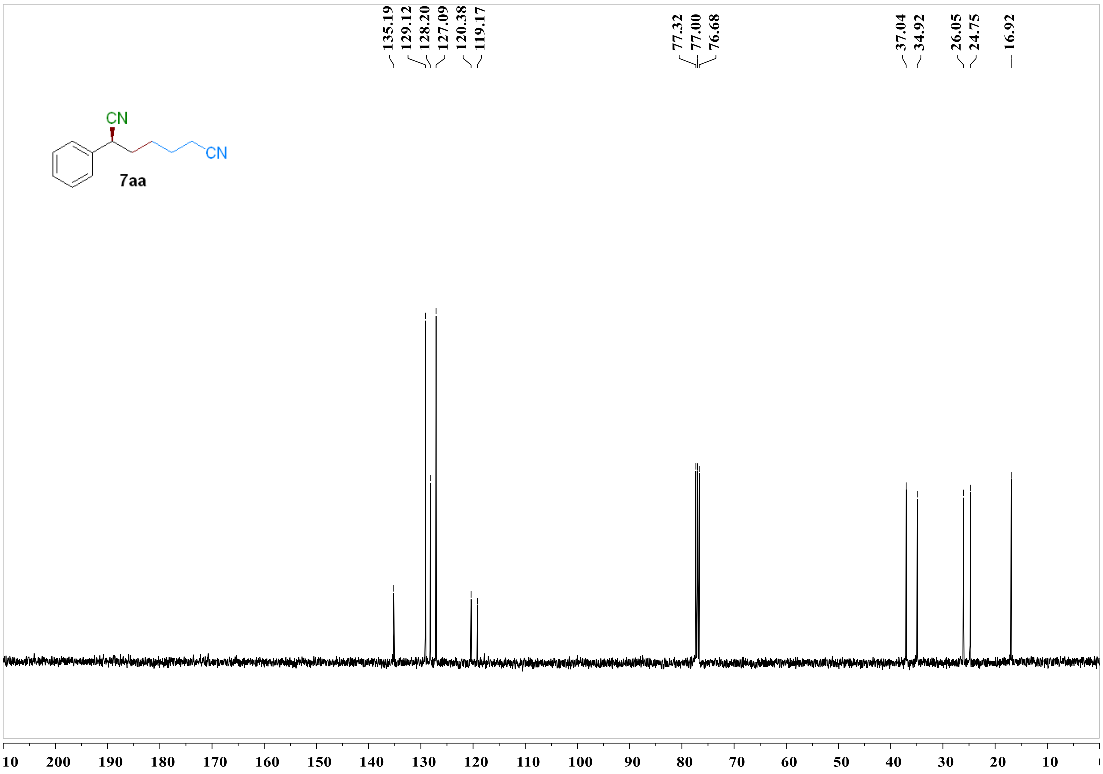

^{1}H NMR (400 MHz, CDCl3) and ^{13}C NMR (100 MHz, CDCl3) spectra of substrate 7ba

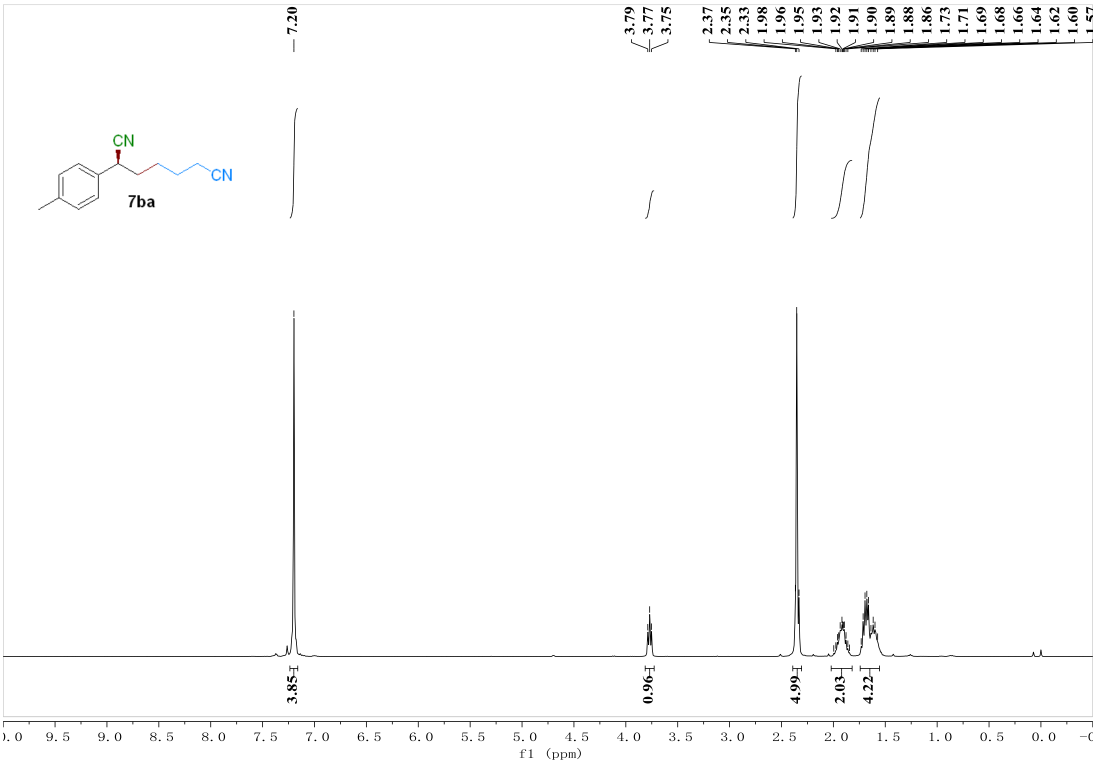

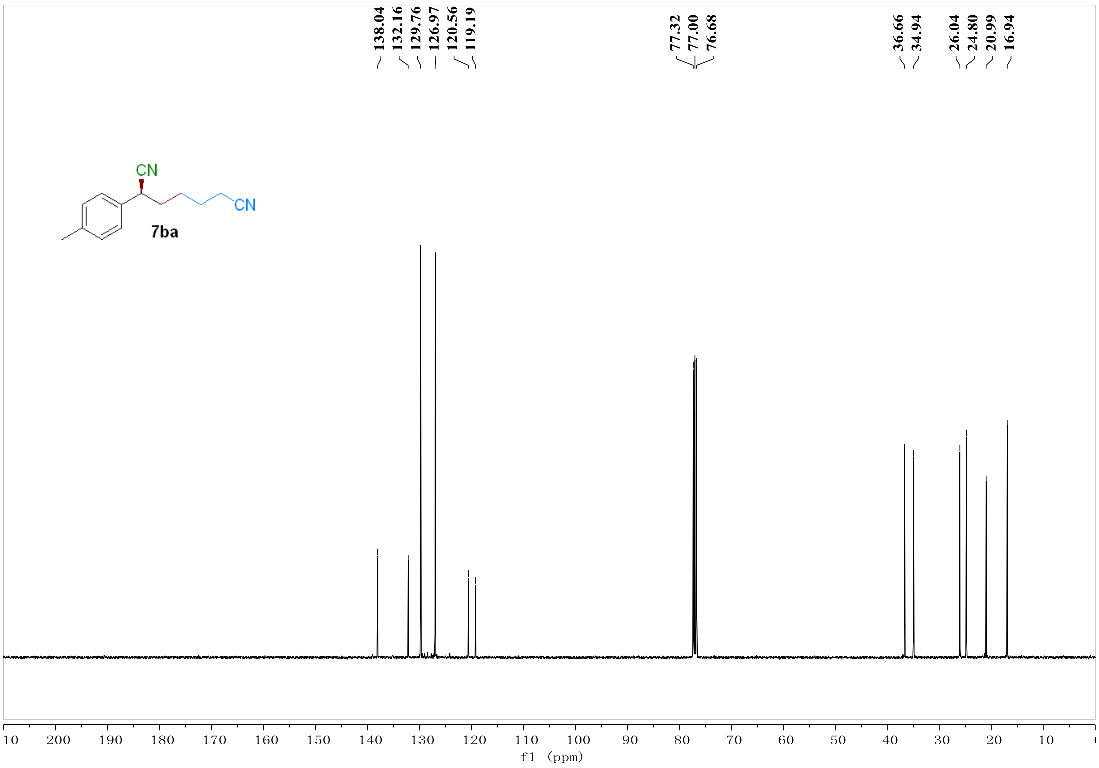

^{1}H NMR (400 MHz, $CDCl_3$) and ^{13}C NMR (100 MHz, $CDCl_3$) spectra of substrate 7ca

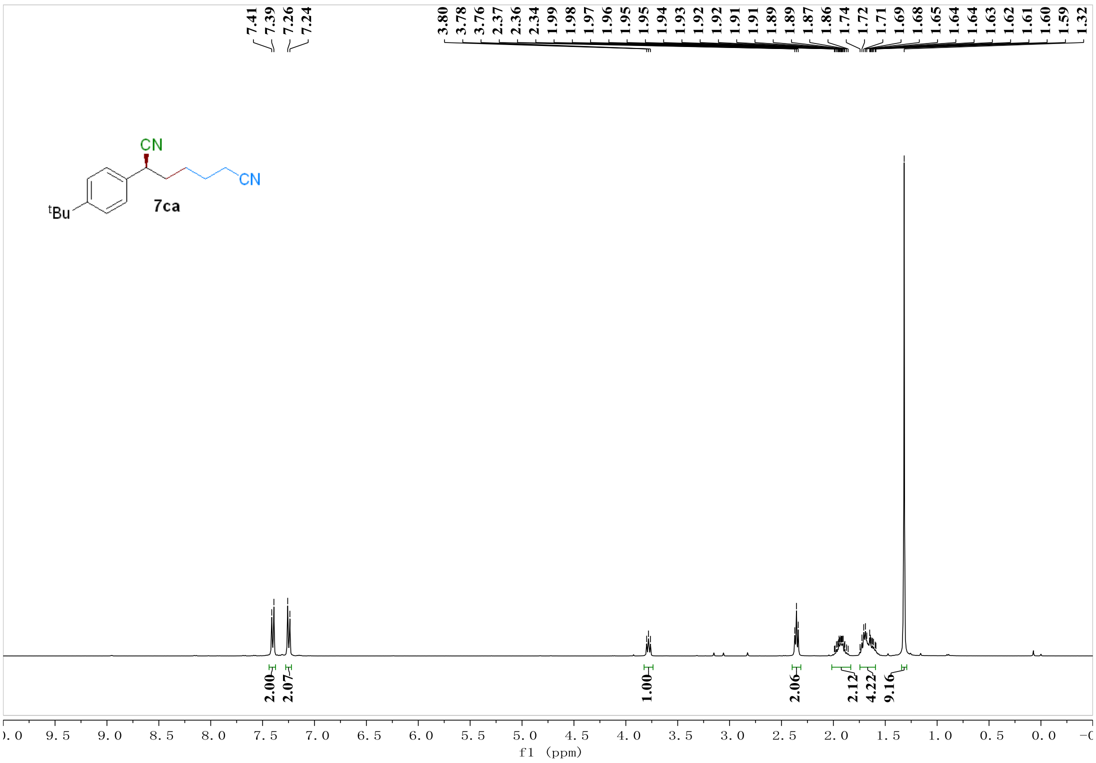

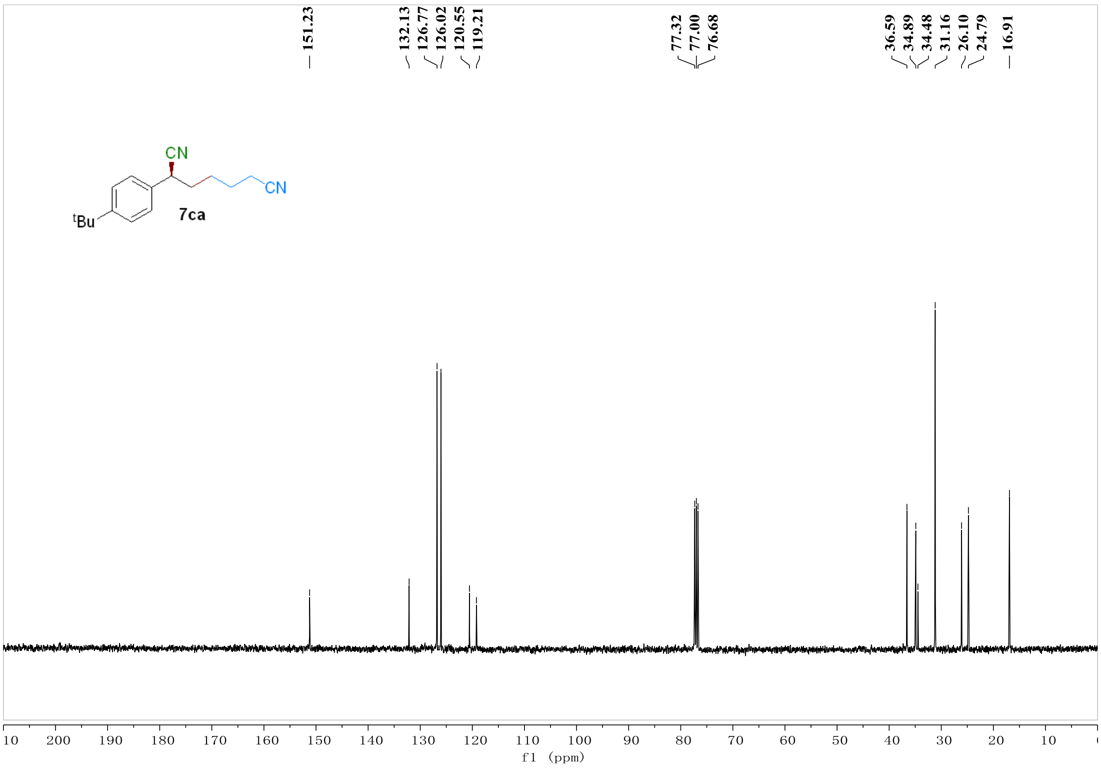

^{1}H NMR (400 MHz, $CDCl_3$) and ^{13}C NMR (100 MHz, $CDCl_3$) spectra of substrate 7da

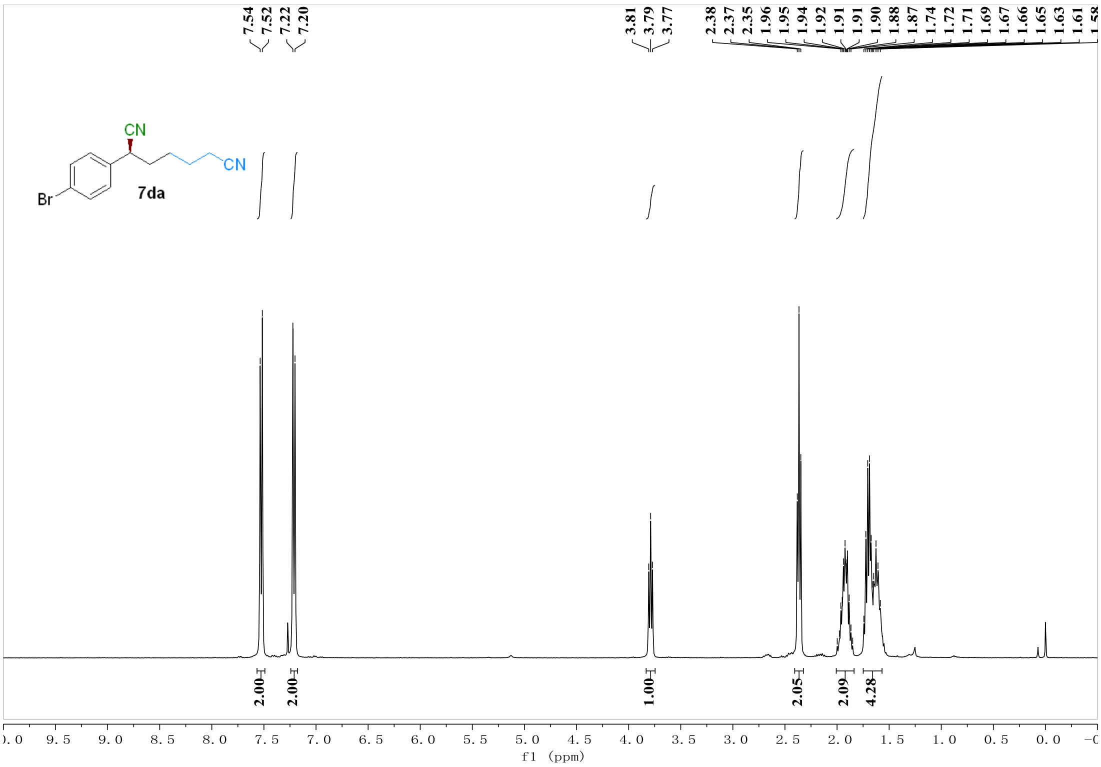

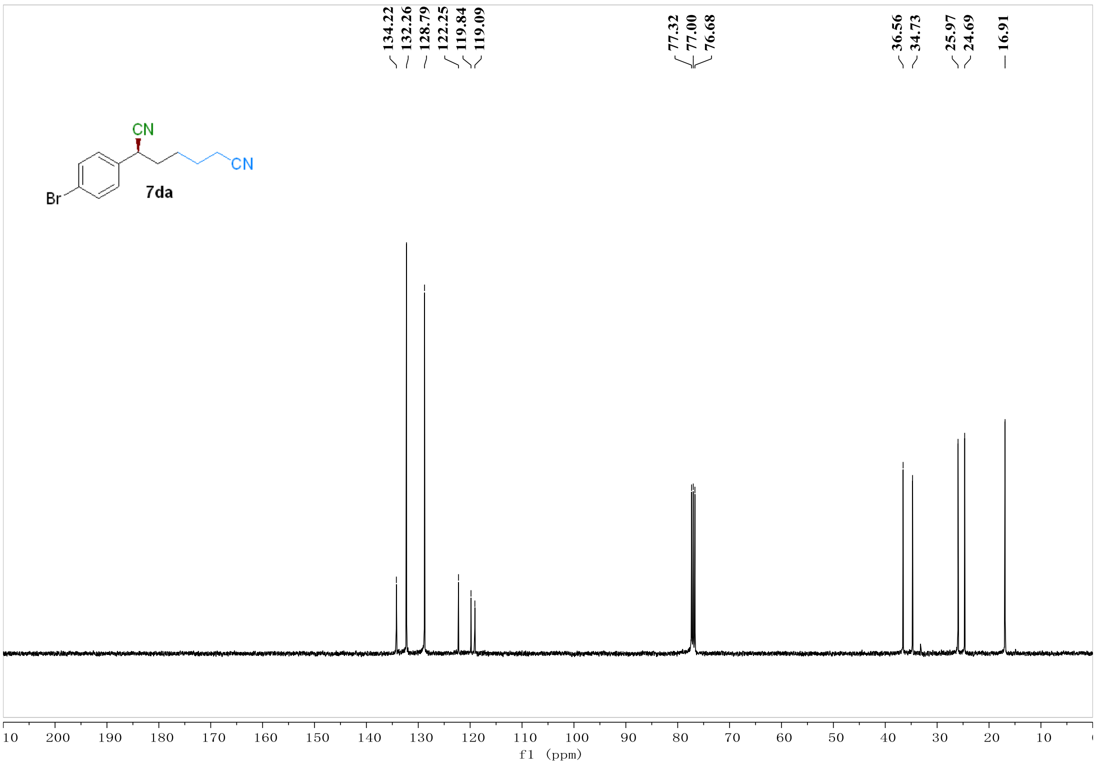

^{1}H NMR (400 MHz, CDCl3) and ^{13}C NMR (100 MHz, CDCl3) spectra of substrate 7ea

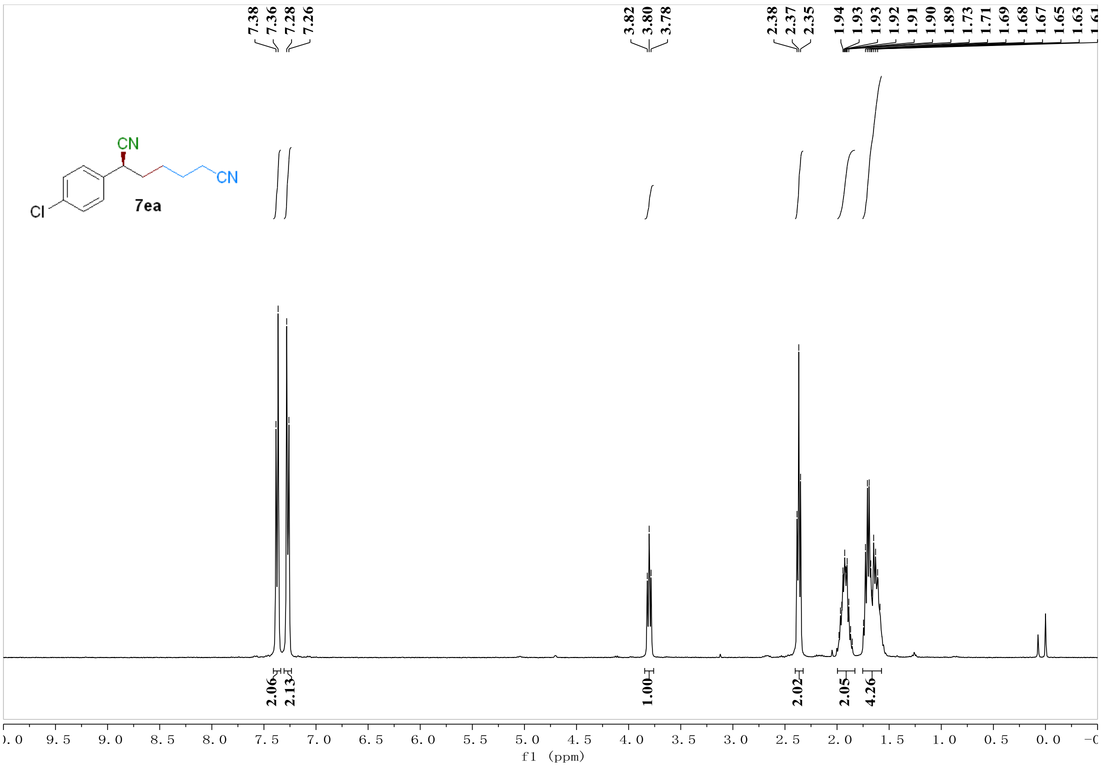

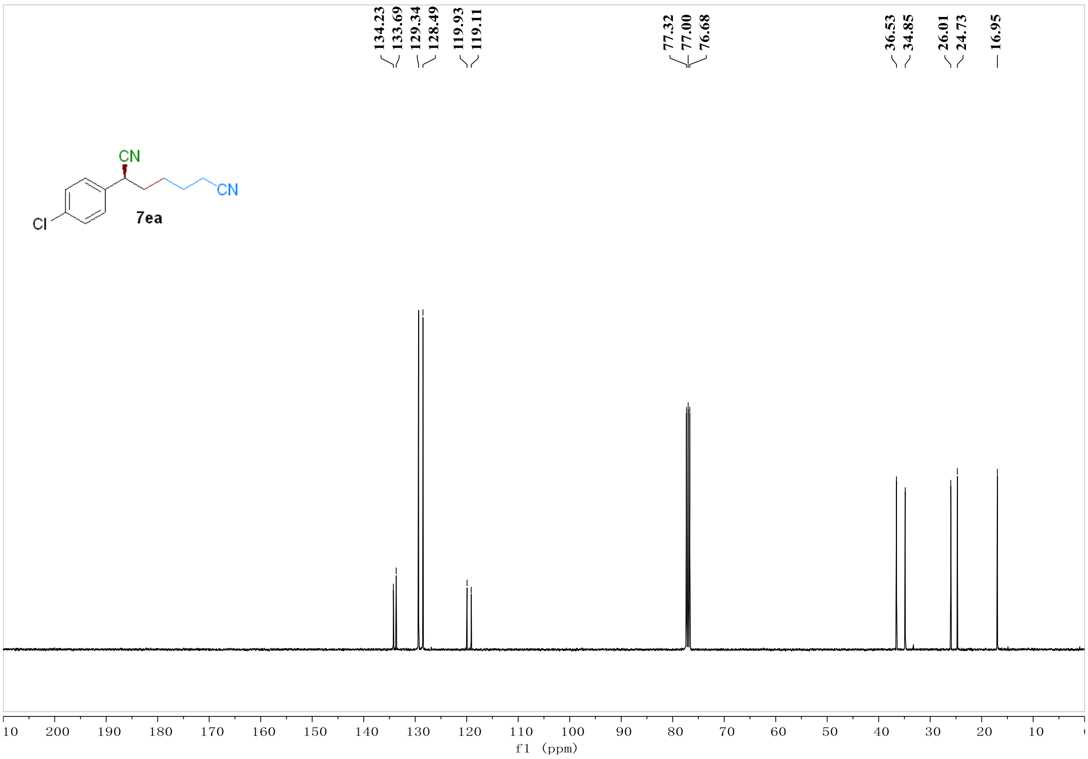

^{1}H NMR (400 MHz, $CDCl_3$), ^{13}C NMR (100 MHz, $CDCl_3$), ^{19}F NMR (376 MHz, $CDCl_3$) spectra of substrate 7fa

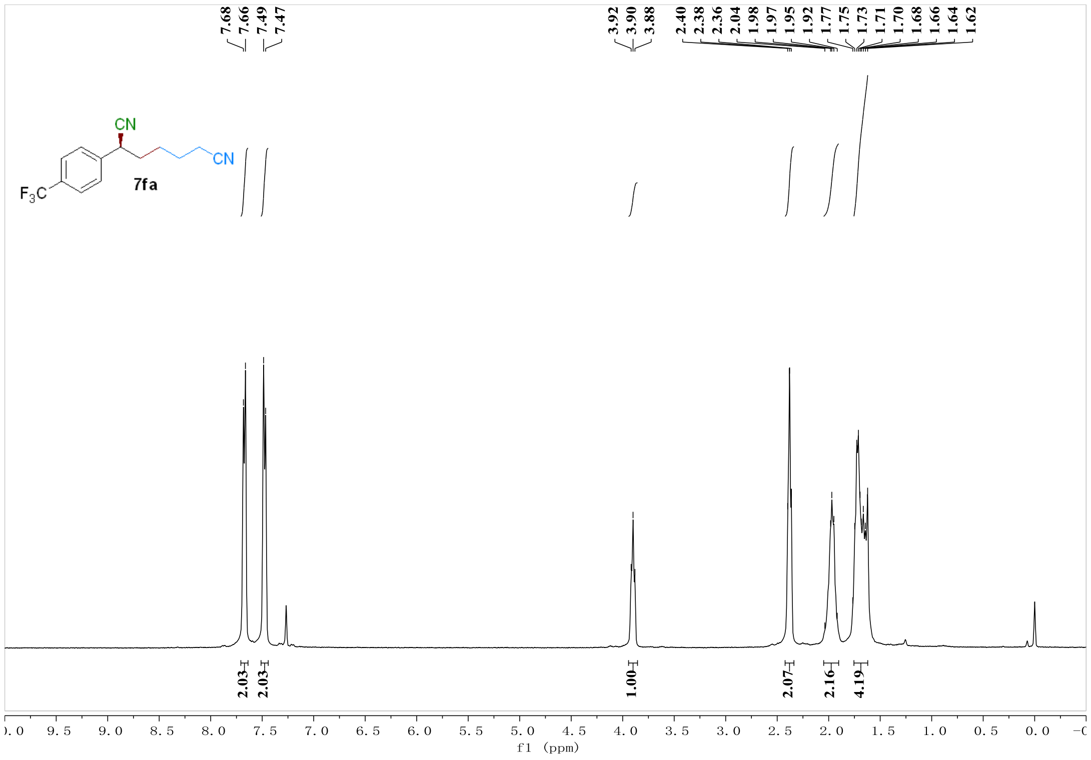

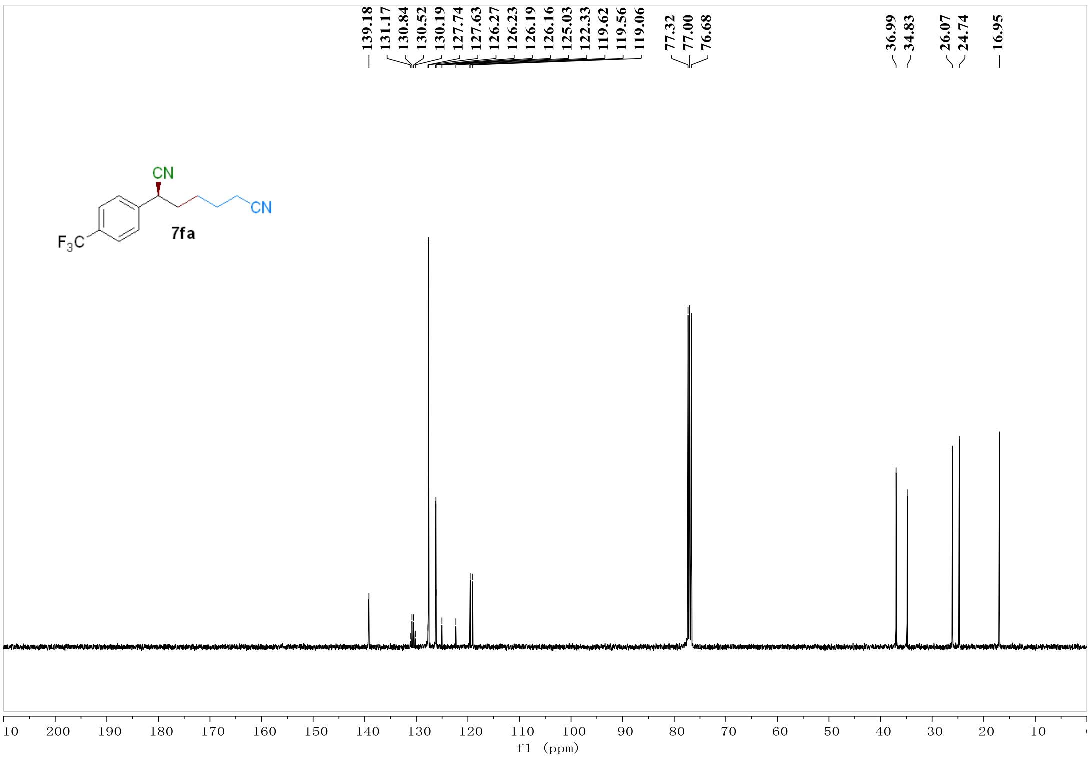

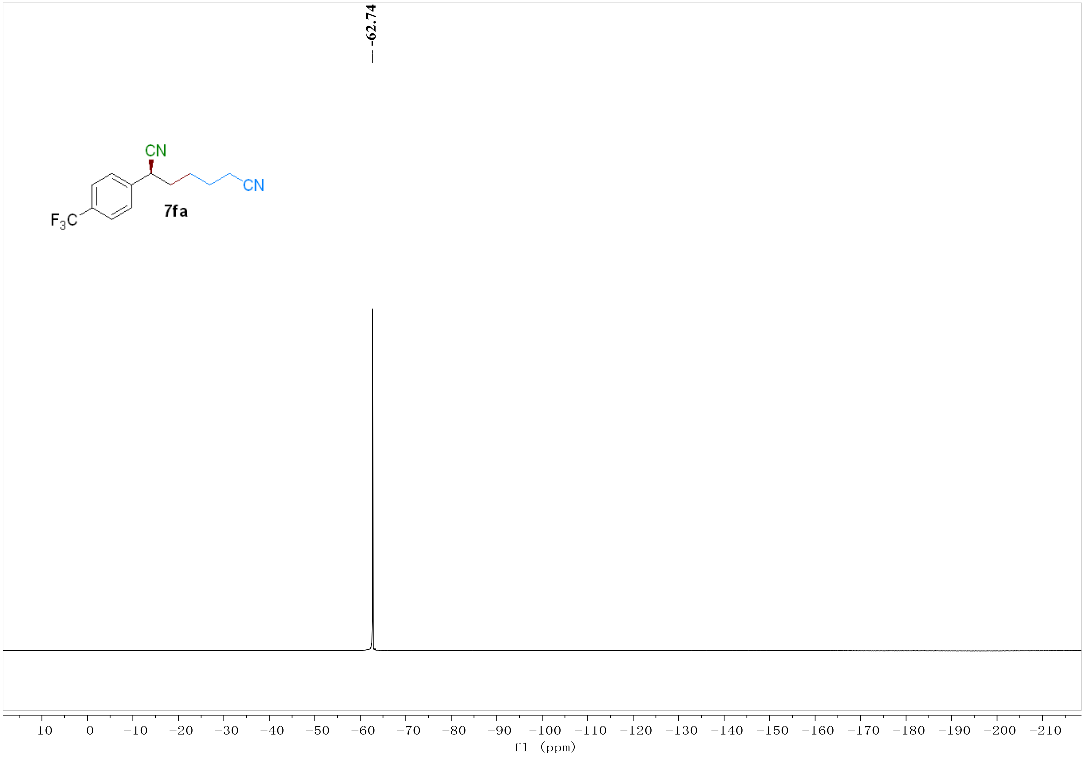
-62.74
CN
CN
F_3C
7fa
10 0 −10 −20 −30 −40 −50 −60 −70 −80 −90 −100 −110 −120 −130 −140 −150 −160 −170 −180 −190 −200 −210
f1 (ppm)

^{1}H NMR (400 MHz, CDCl$_3$) and ^{13}C NMR (100 MHz, CDCl$_3$) spectra of substrate 7ga

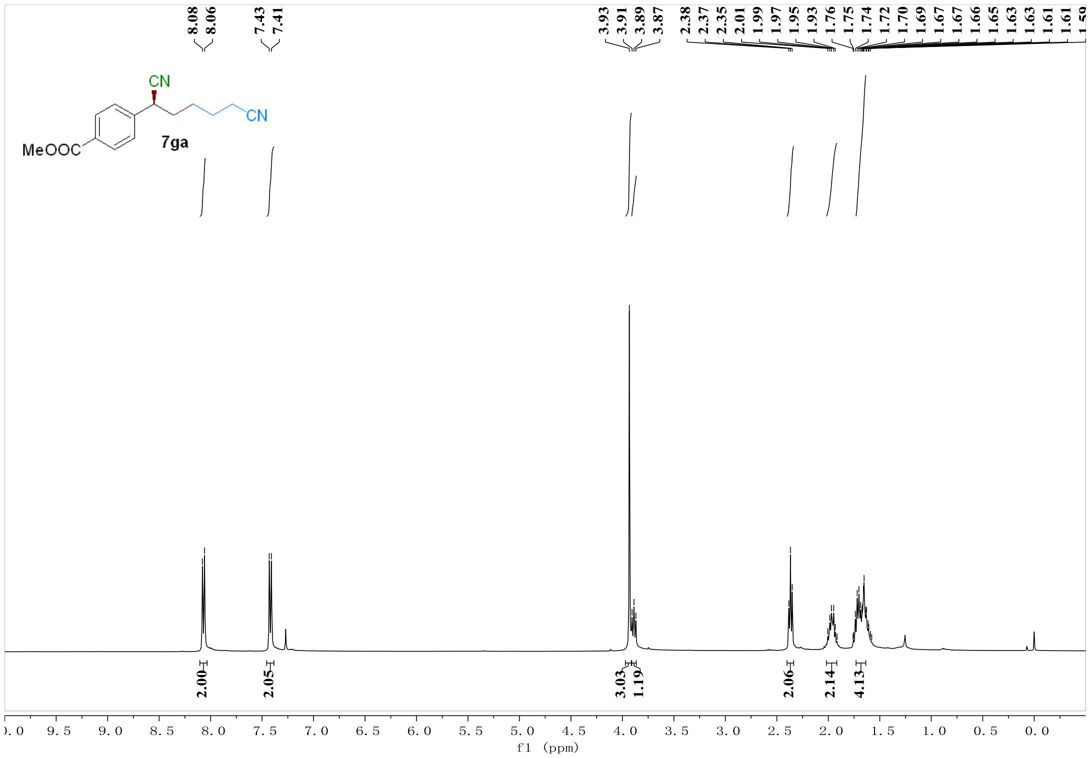

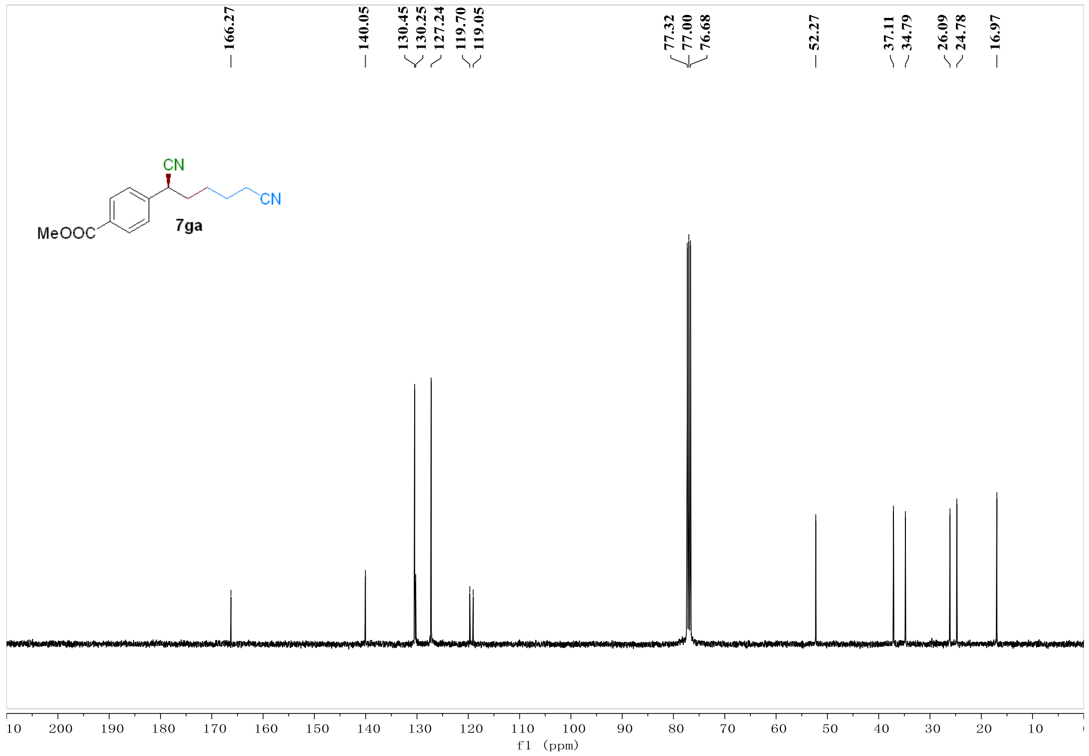

^{1}H NMR (400 MHz, $CDCl_3$) and ^{13}C NMR (100 MHz, $CDCl_3$) spectra of substrate 7ha

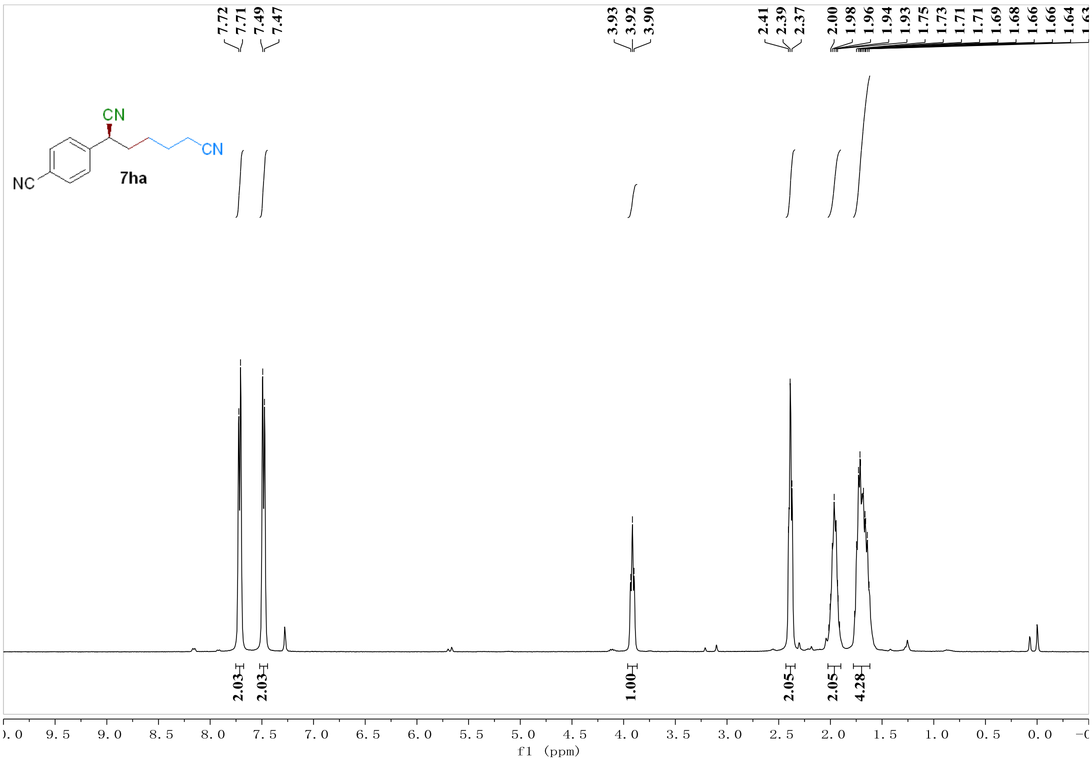

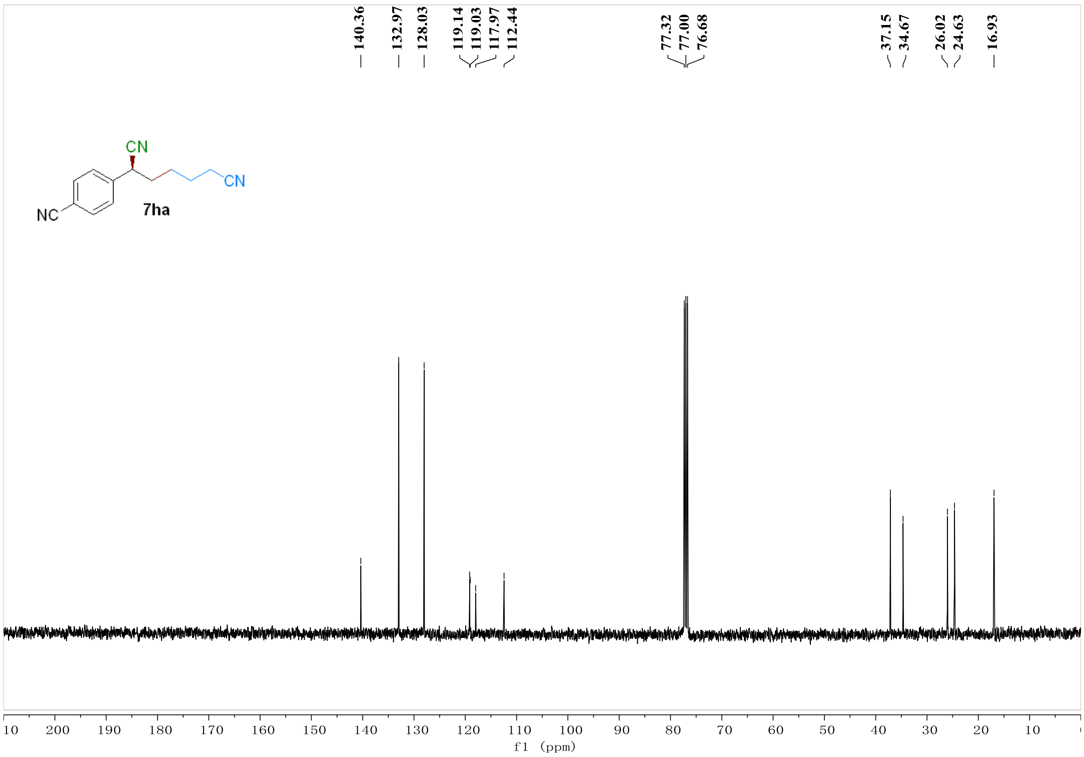

^{1}H NMR (400 MHz, CDCl3) and ^{13}C NMR (100 MHz, CDCl3) spectra of substrate 7ia

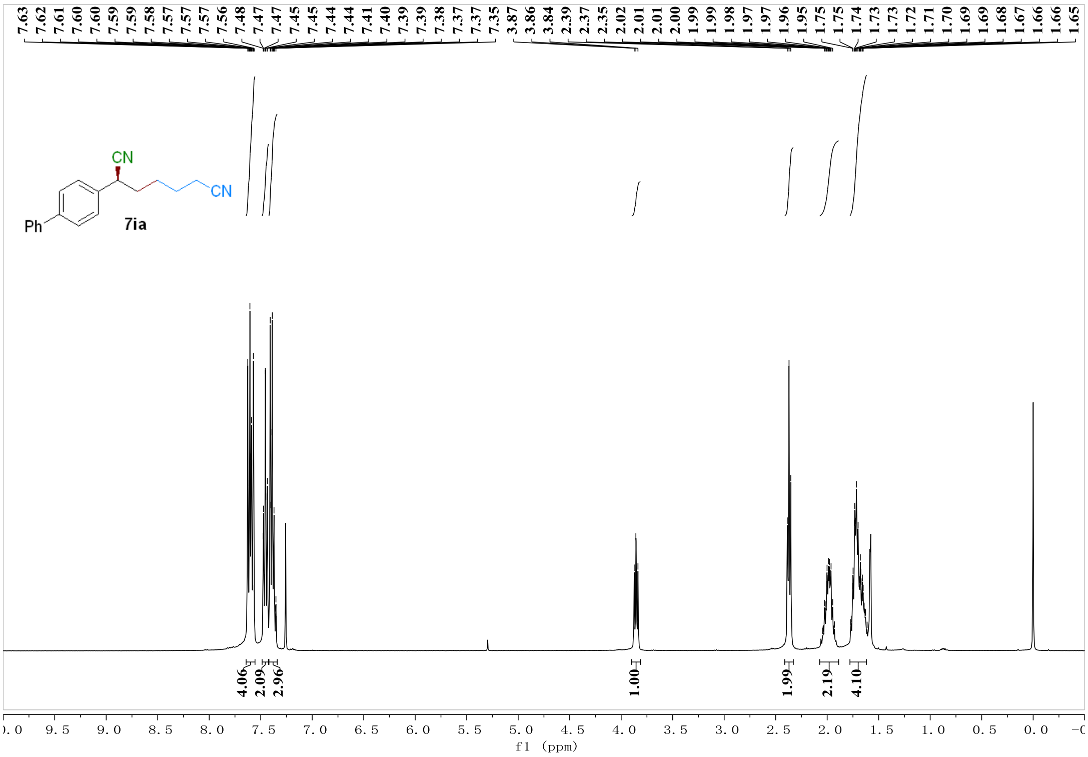

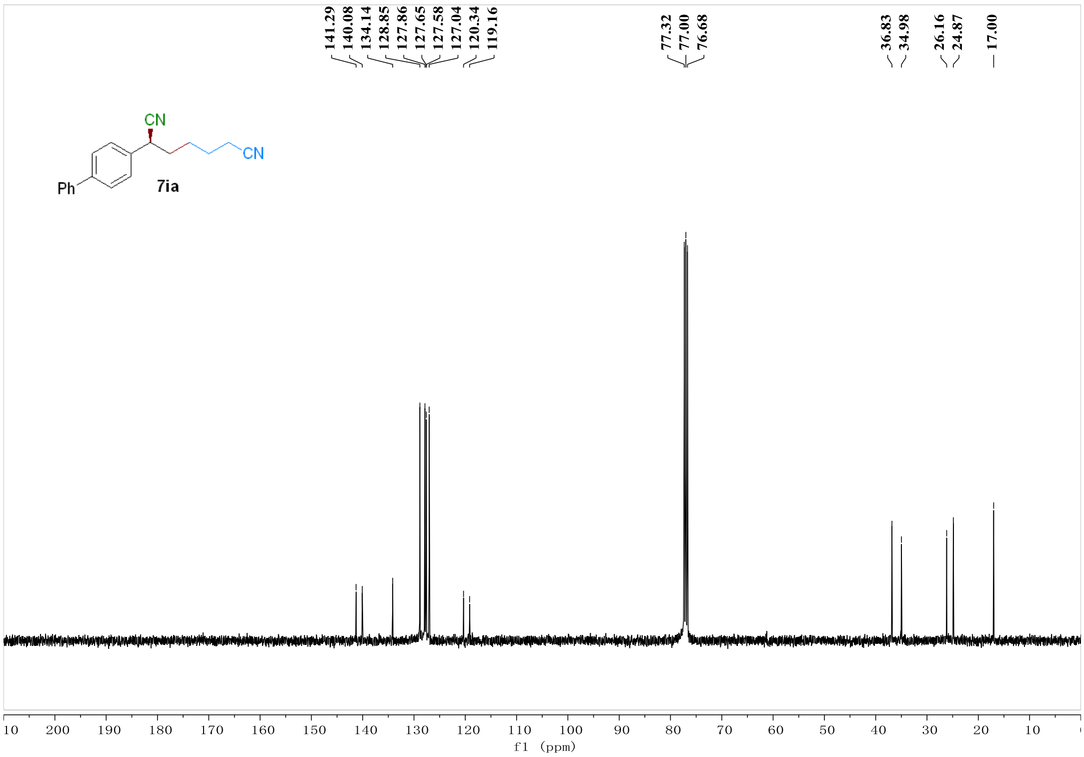

^1H NMR (400 MHz, $CDCl_3$) and ^{13}C NMR (100 MHz, $CDCl_3$) spectra of substrate 7ja

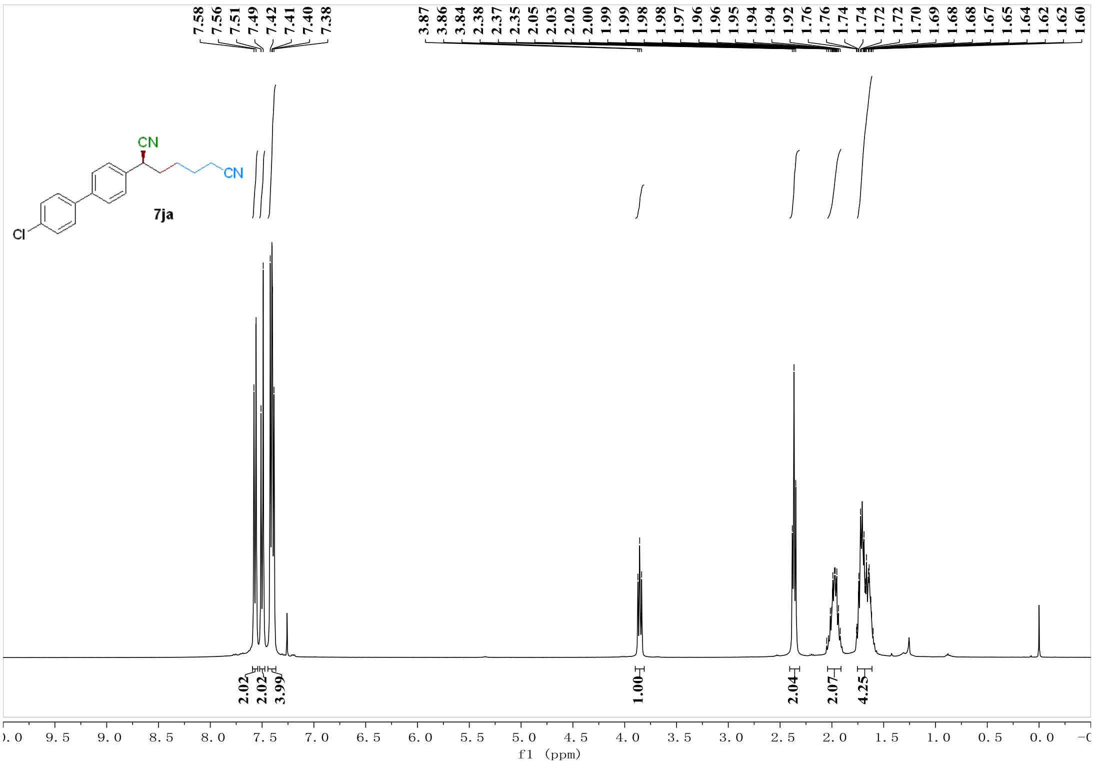

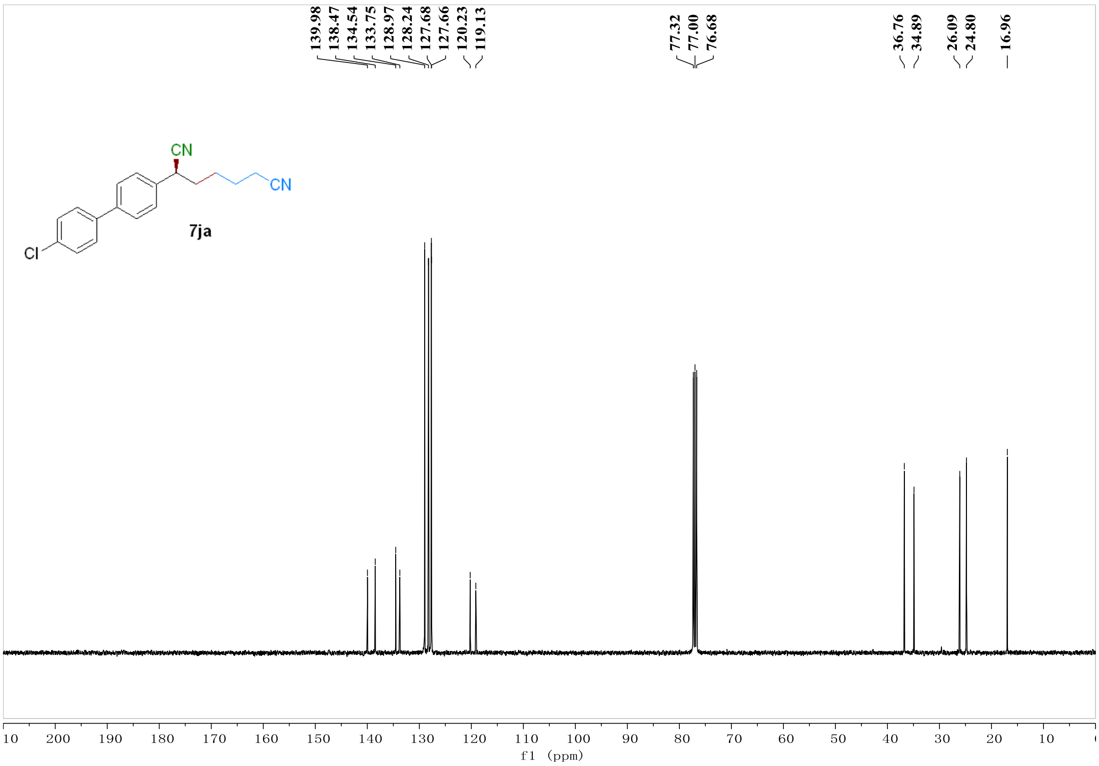

^{1}H NMR (400 MHz, $CDCl_3$) and ^{13}C NMR (100 MHz, $CDCl_3$) spectra of substrate 7ka

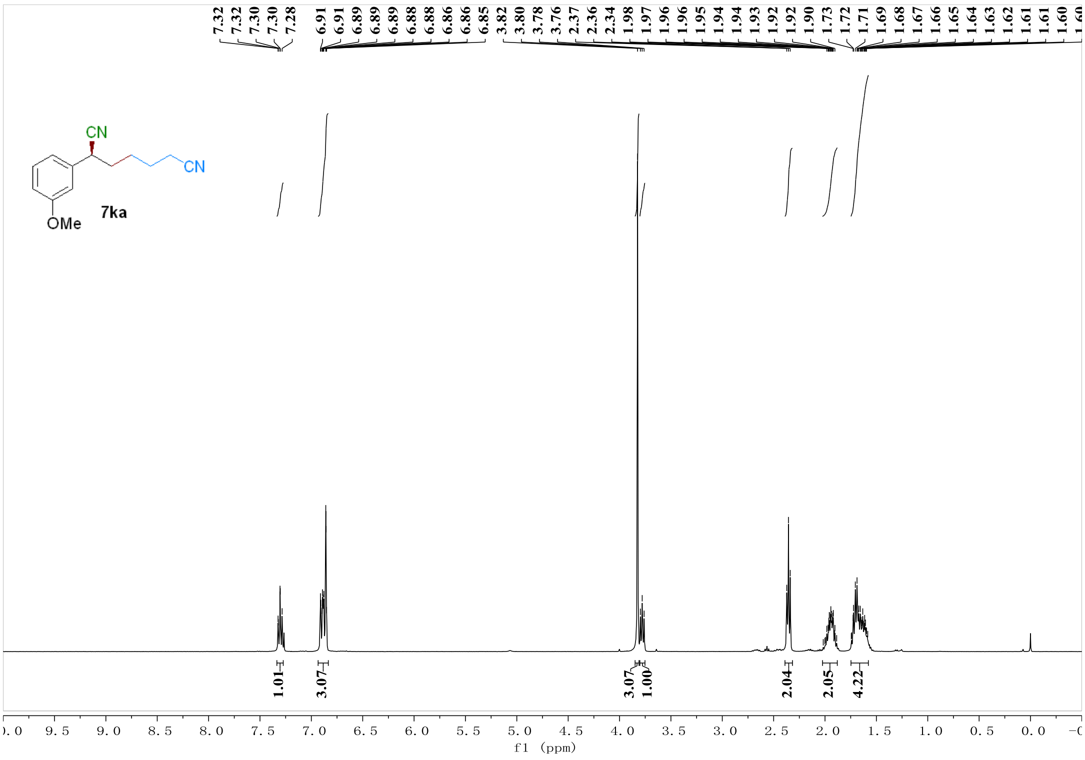

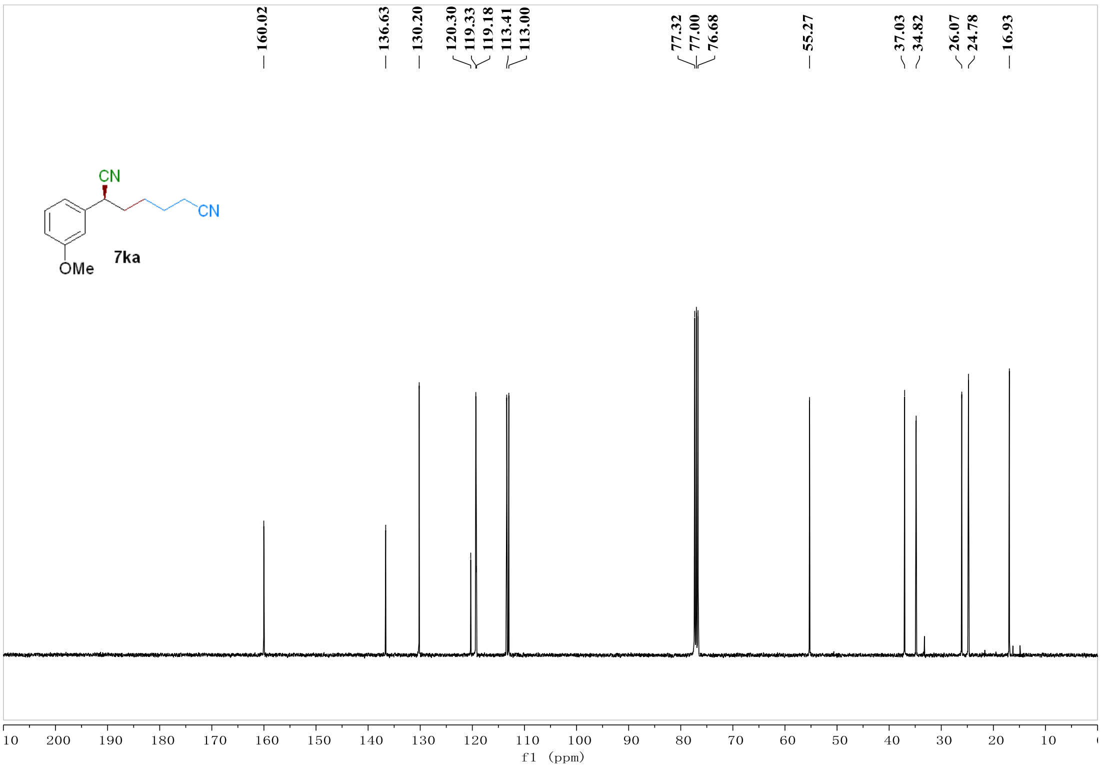

^{1}H NMR (400 MHz, CDCl3), ^{13}C NMR (100 MHz, CDCl3), ^{19}F NMR (376 MHz, CDCl3) spectra of substrate 7la

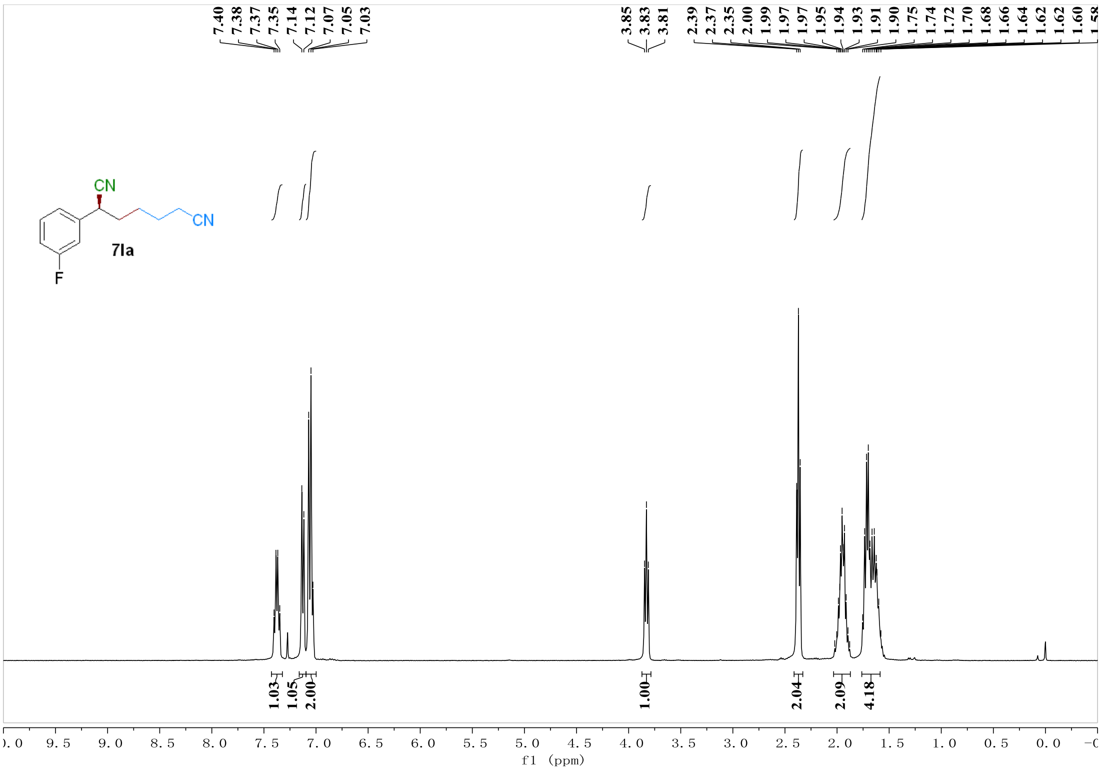

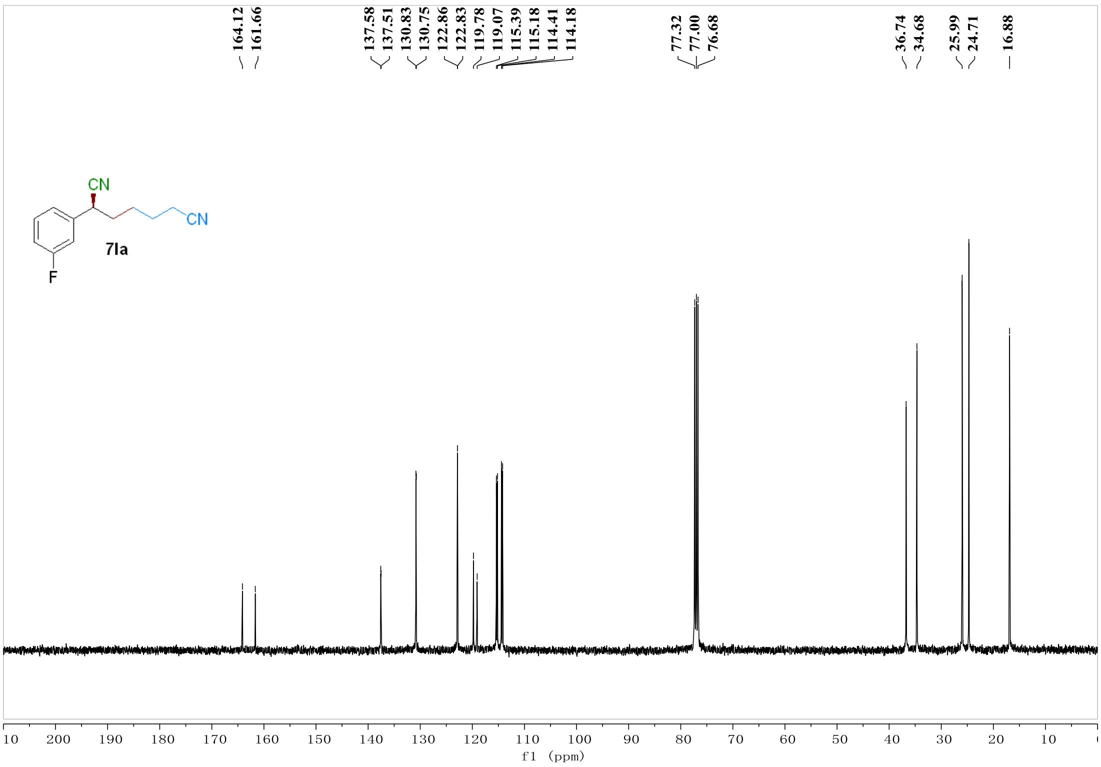

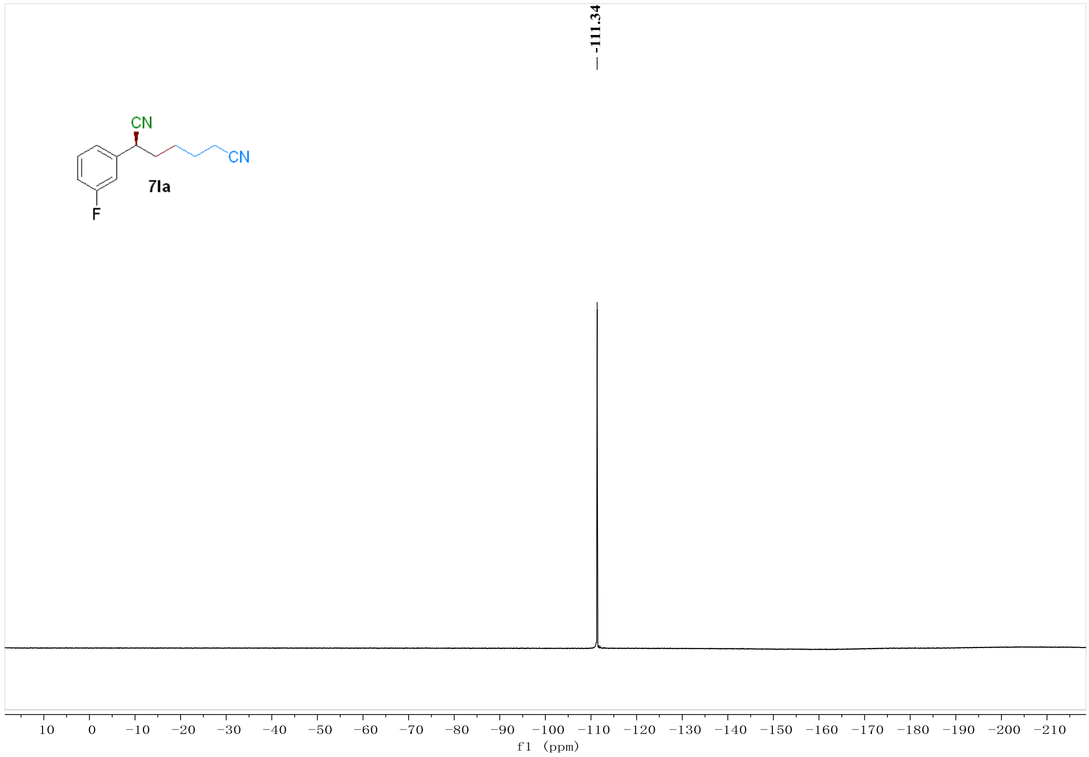
-111.34
CN
CN
F
7la
10 0 −10 −20 −30 −40 −50 −60 −70 −80 −90 −100 −110 −120 −130 −140 −150 −160 −170 −180 −190 −200 −210
f1 (ppm)

^{1}H NMR (400 MHz, $CDCl_3$) and ^{13}C NMR (100 MHz, $CDCl_3$) spectra of substrate 7ma

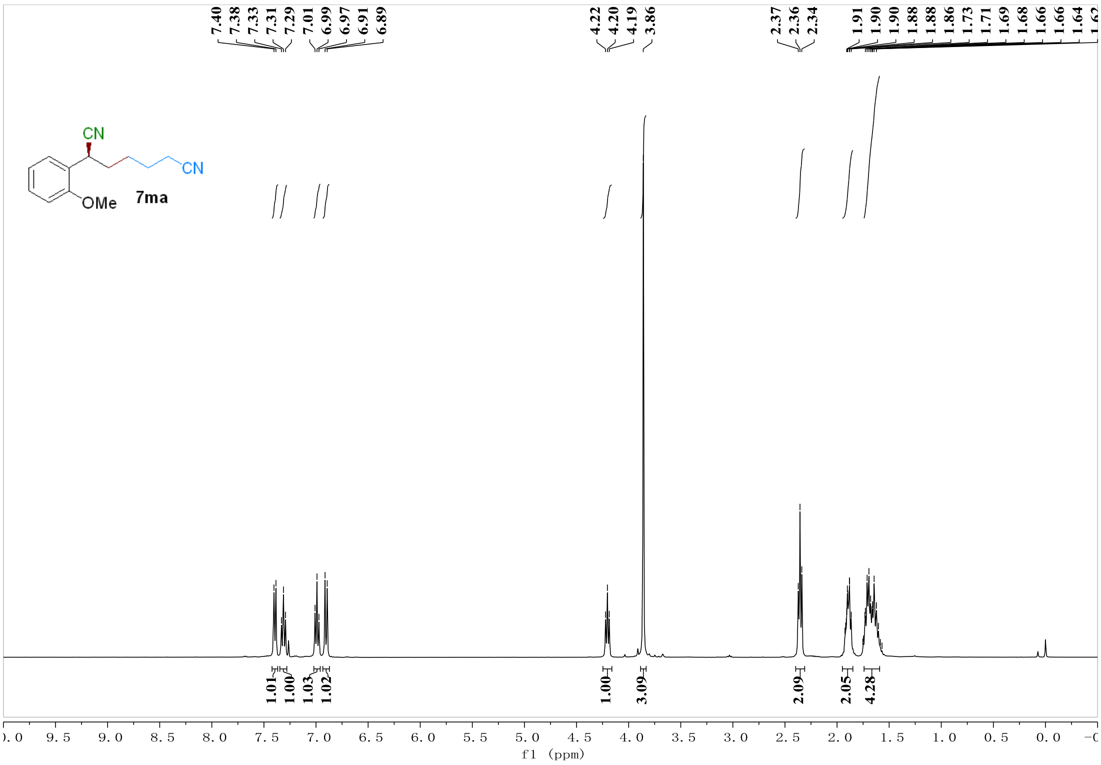

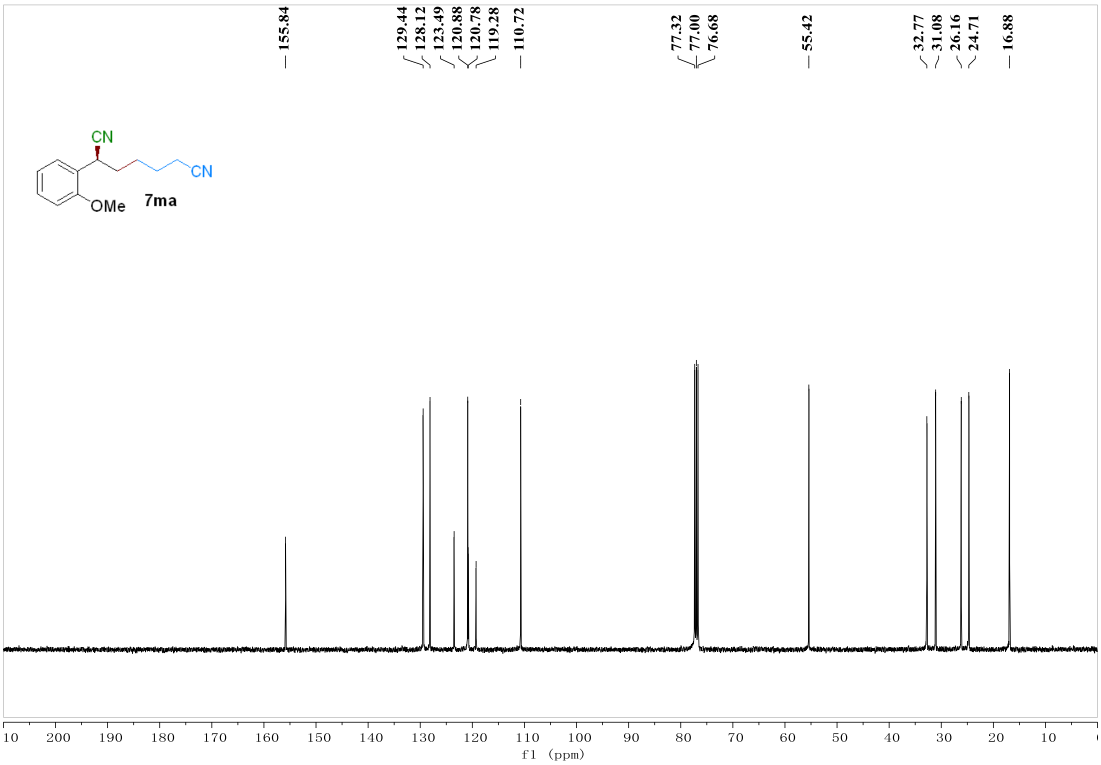

^{1}H NMR (400 MHz, $CDCl_3$) and ^{13}C NMR (100 MHz, $CDCl_3$) spectra of substrate 7na

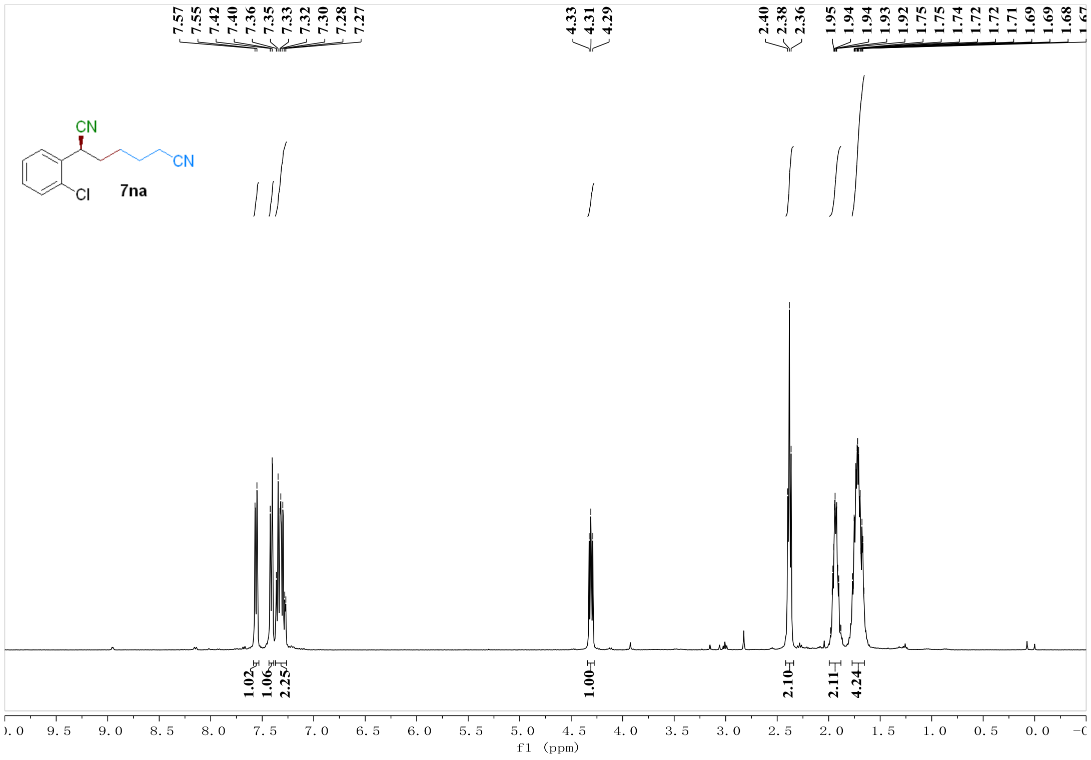

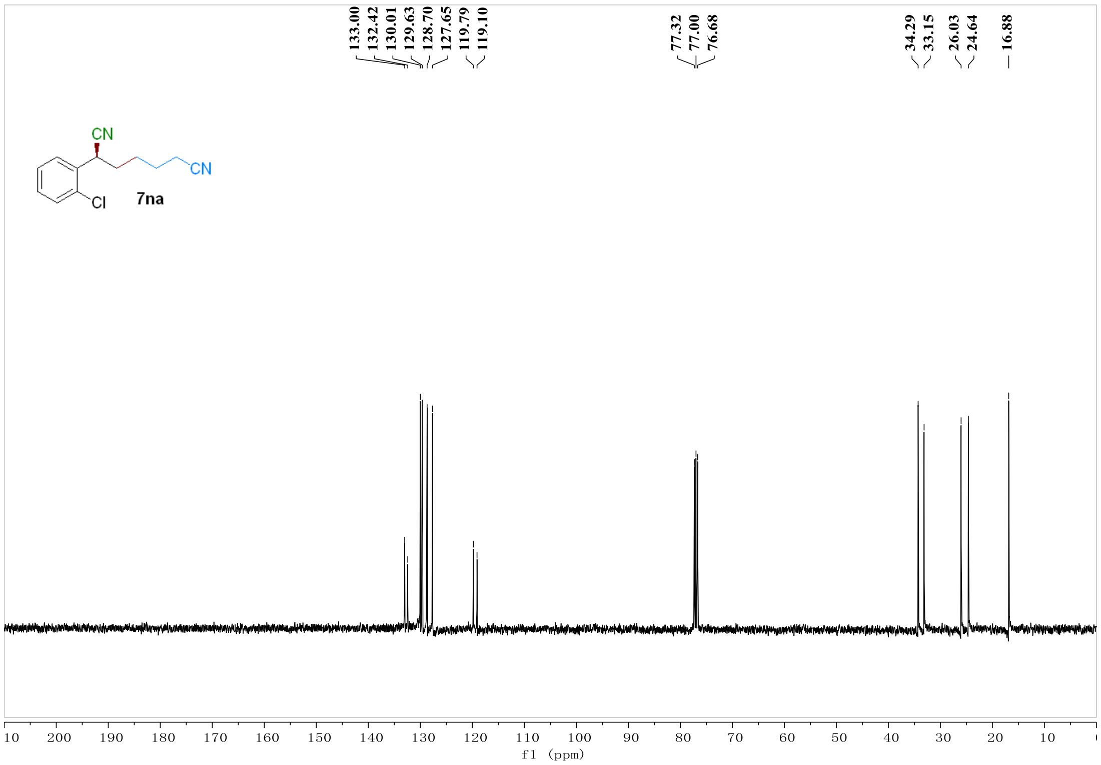

^{1}H NMR (400 MHz, $CDCl_3$) and ^{13}C NMR (100 MHz, $CDCl_3$) spectra of substrate 7oa

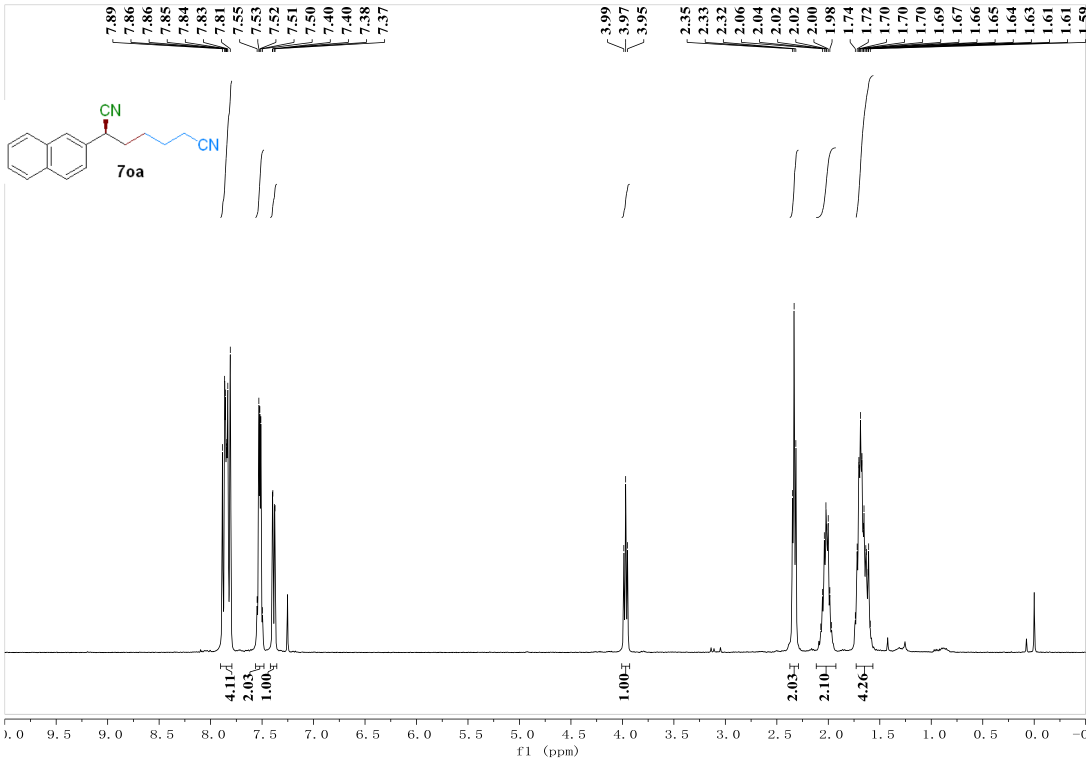

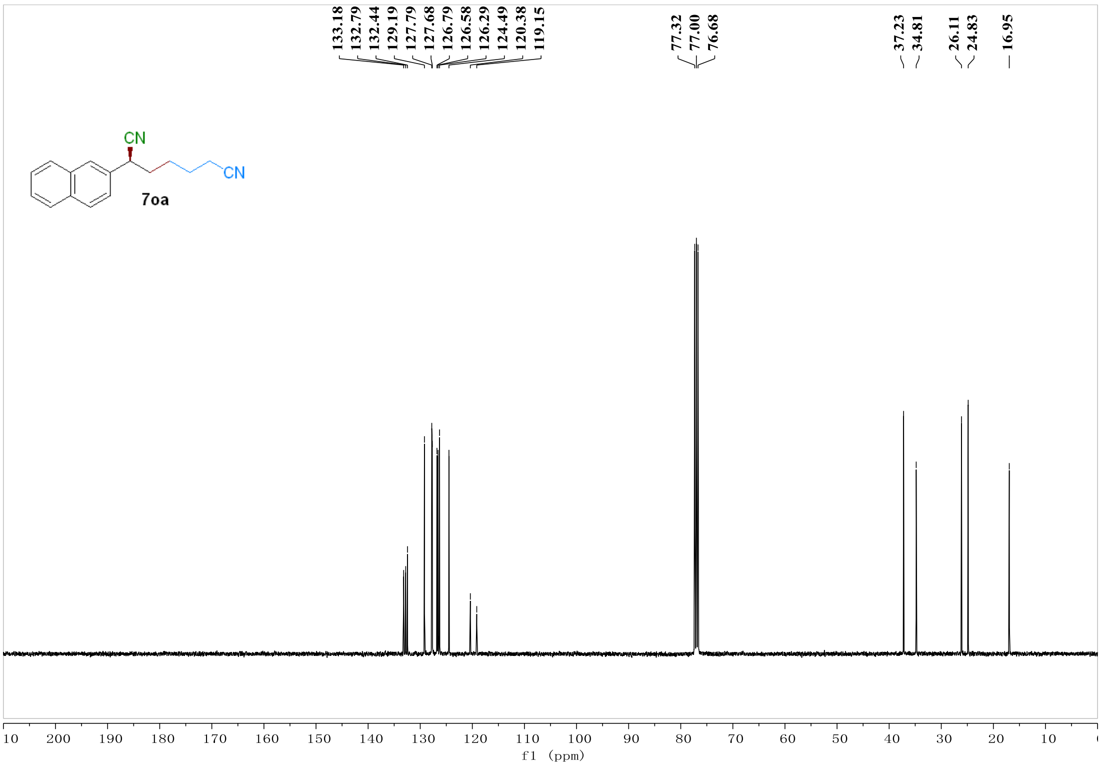

^{1}H NMR (400 MHz, $CDCl_3$) and ^{13}C NMR (100 MHz, $CDCl_3$) spectra of substrate 7pa

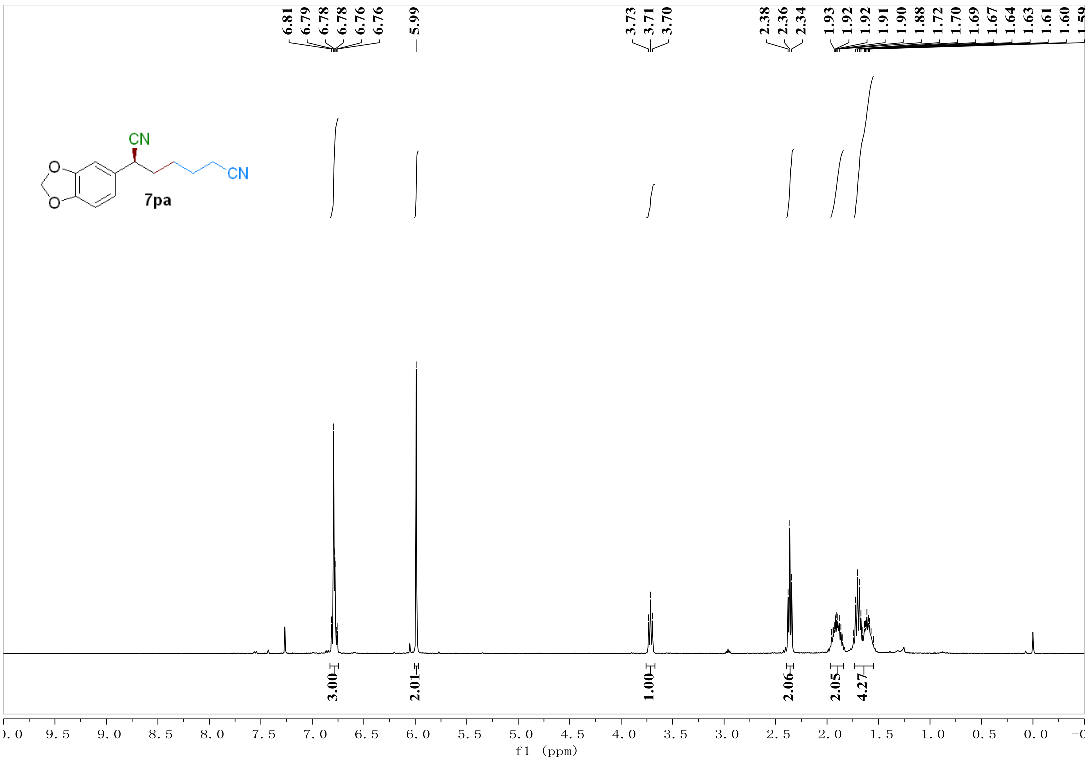

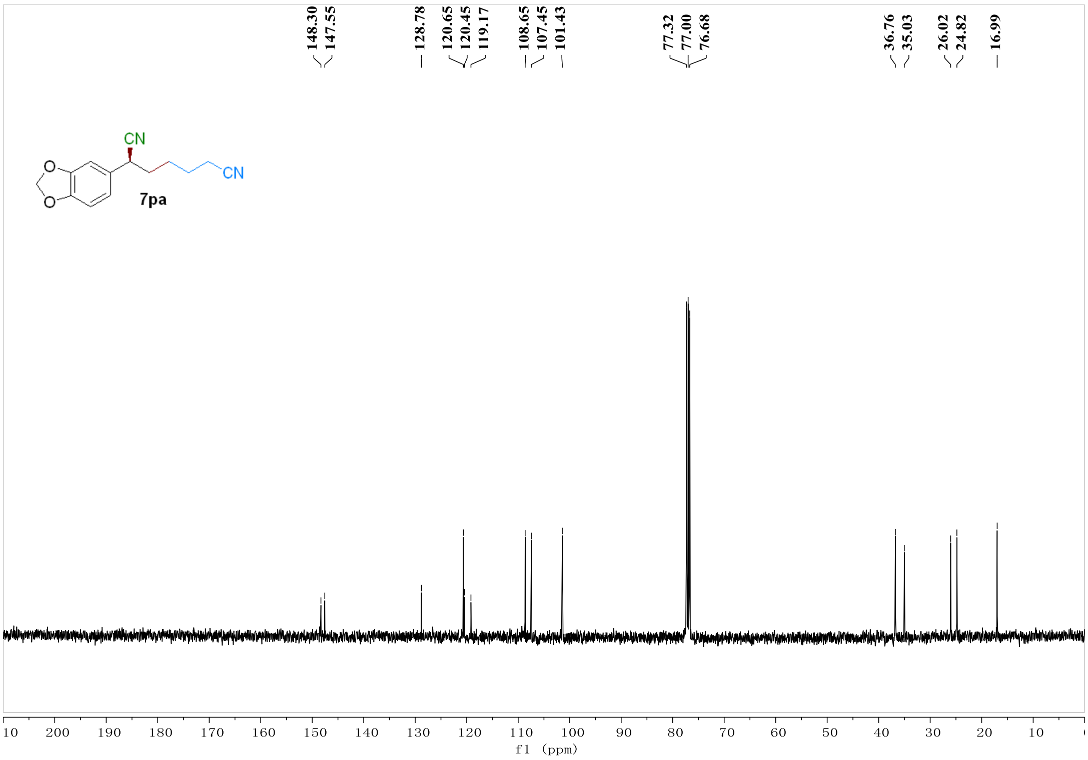

^{1}H NMR (400 MHz, CDCl3) and ^{13}C NMR (100 MHz, CDCl3) spectra of substrate 7qa

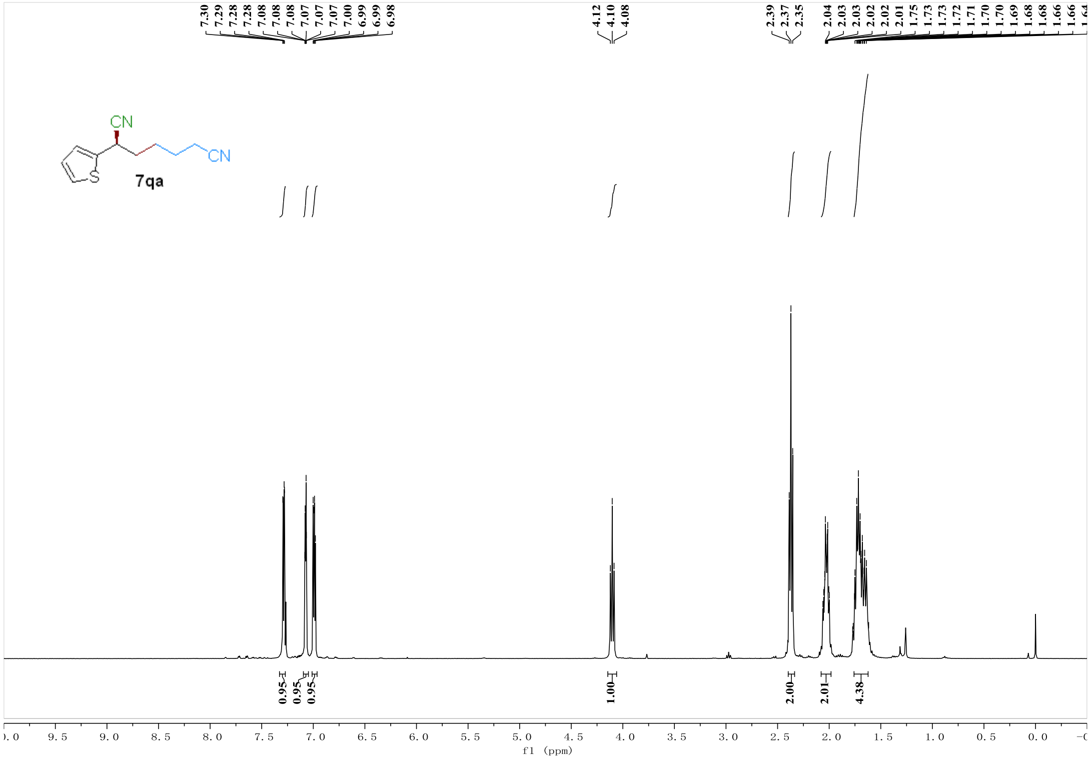

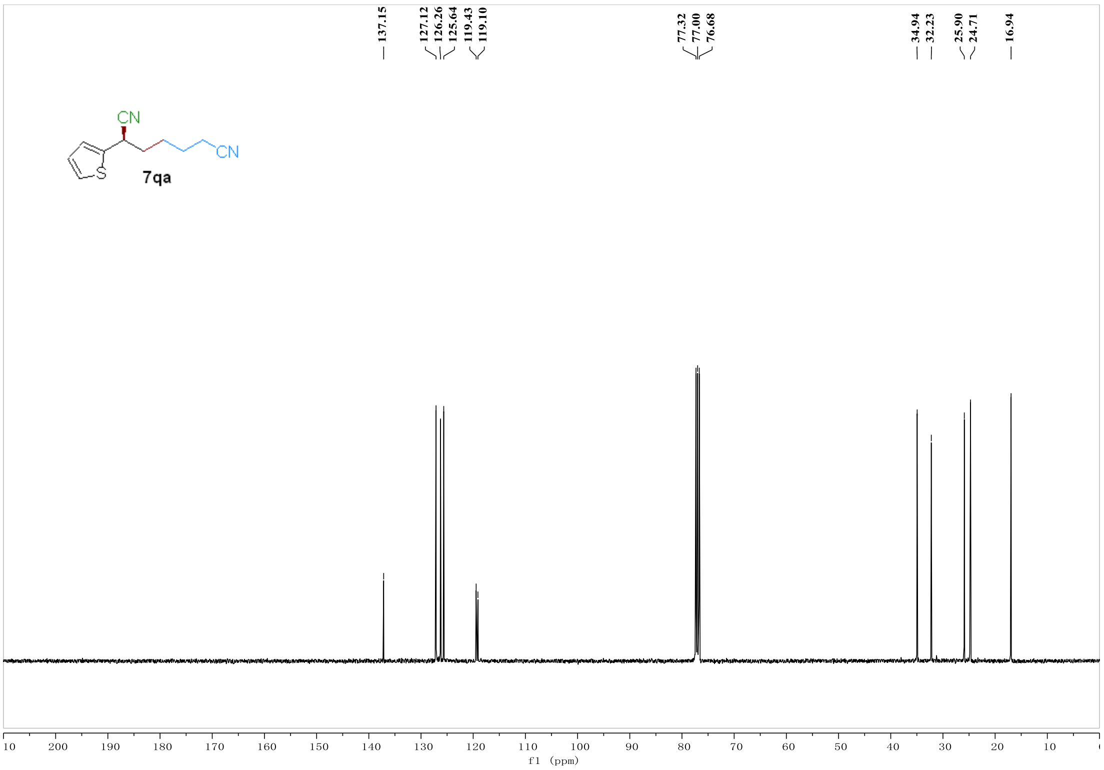

^{1}H NMR (400 MHz, CDCl$_3$) and ^{13}C NMR (100 MHz, CDCl$_3$) spectra of substrate 7ra

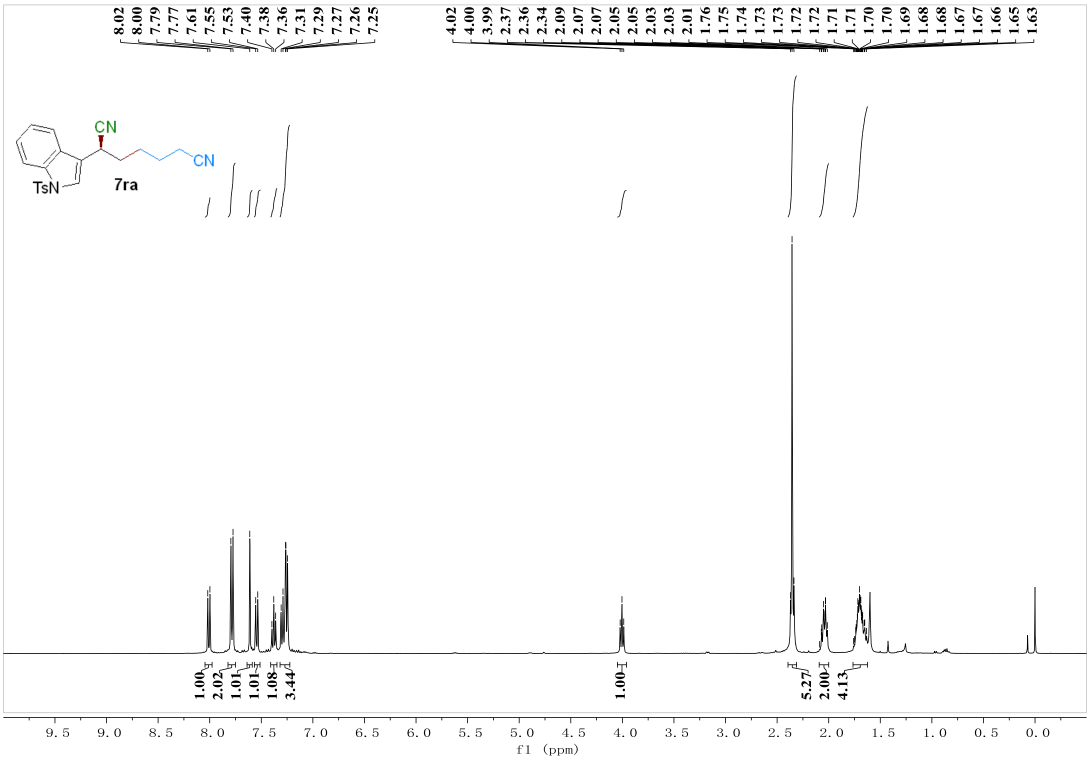

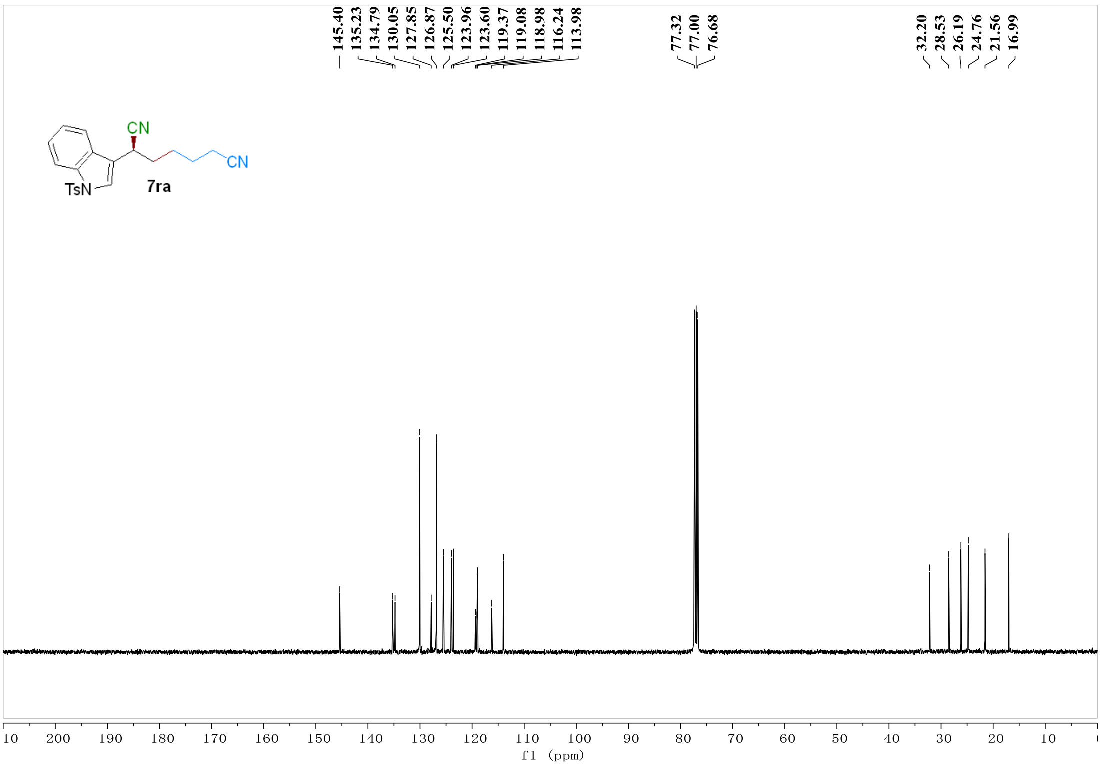

^{1}H NMR (400 MHz, CDCl$_3$) and ^{13}C NMR (100 MHz, CDCl$_3$) spectra of substrate 7sa

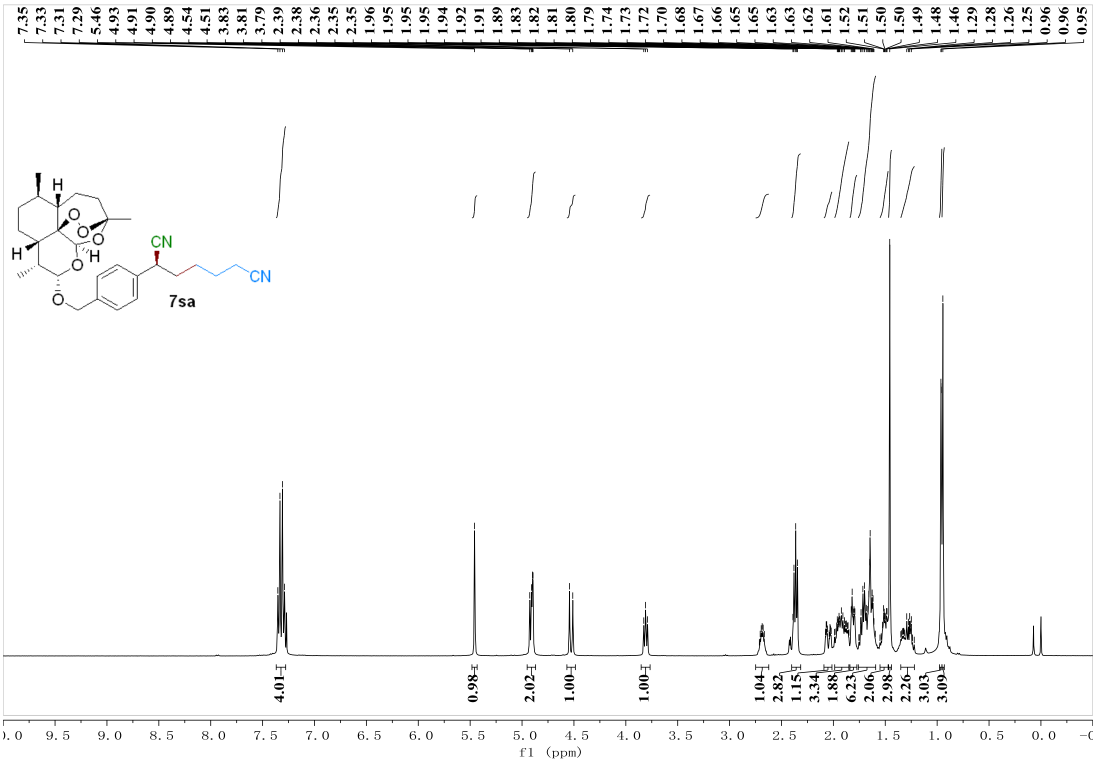

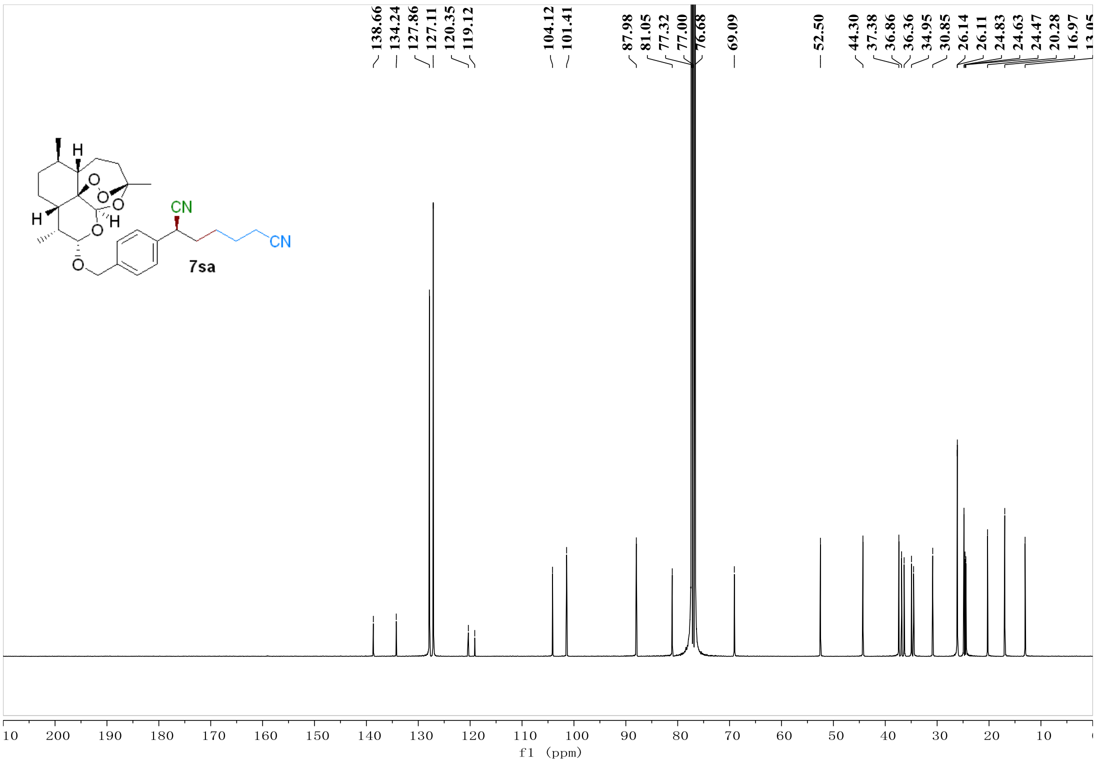

^{1}H NMR (400 MHz, CDCl3) and ^{13}C NMR (100 MHz, CDCl3) spectra of substrate 7ta

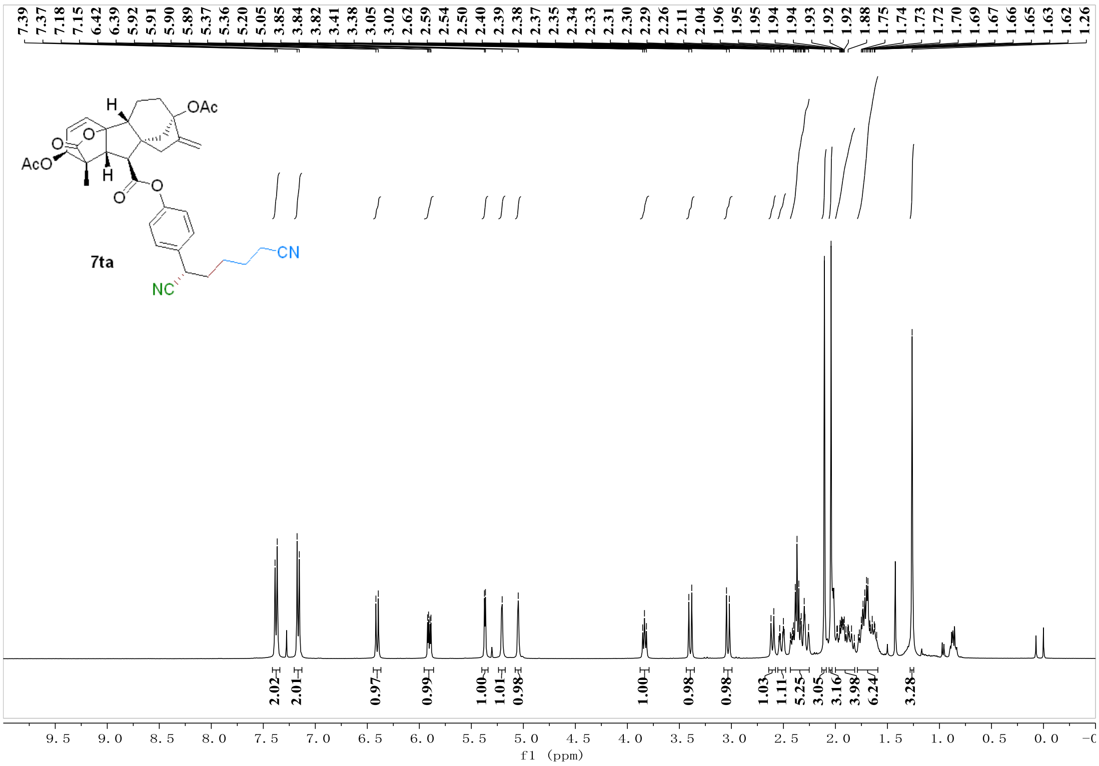

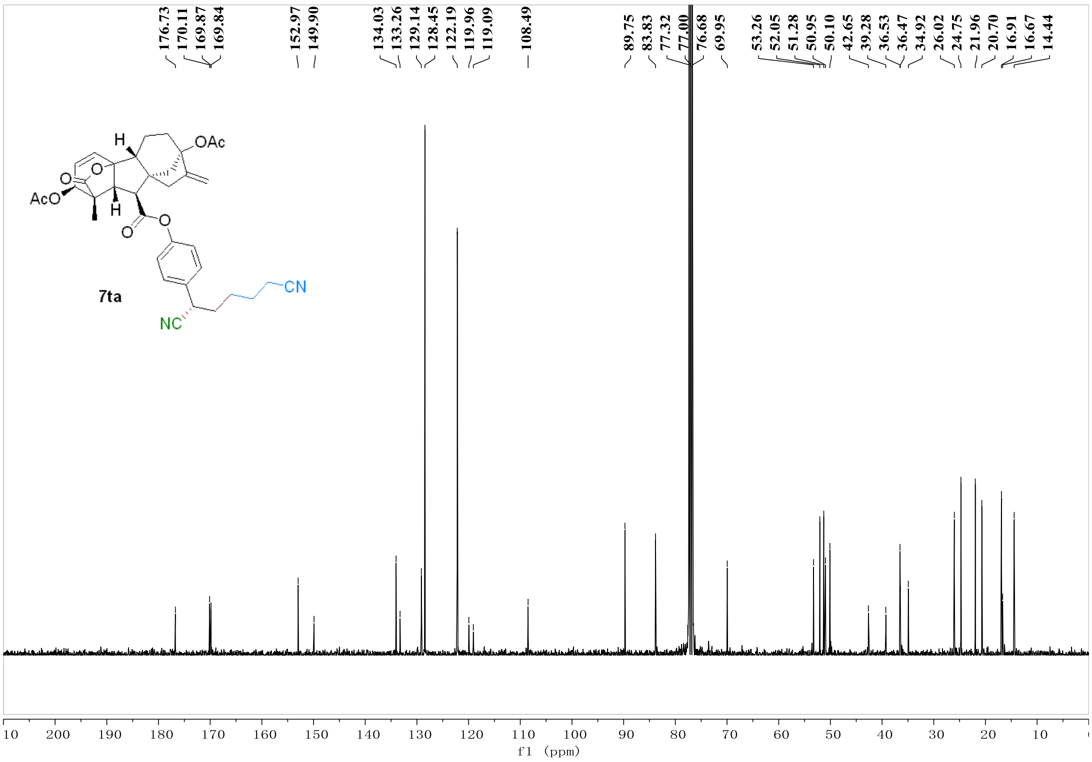

^{1}H NMR (400 MHz, $CDCl_3$) and ^{13}C NMR (100 MHz, $CDCl_3$) spectra of substrate 7ib

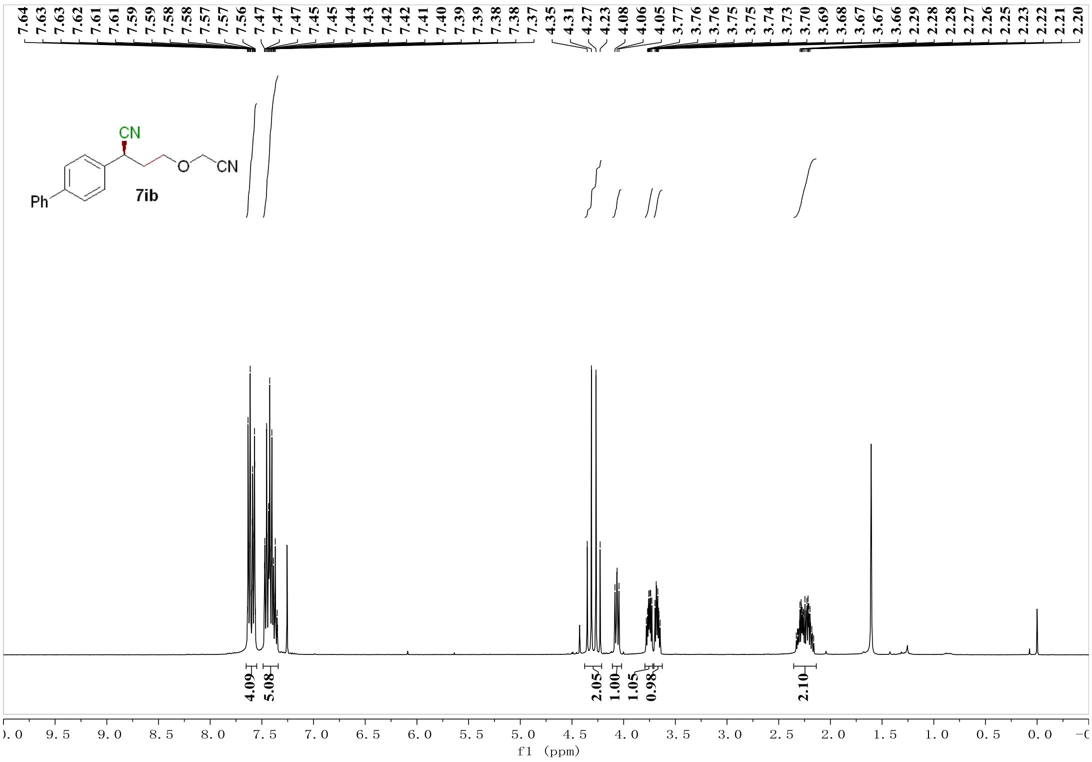

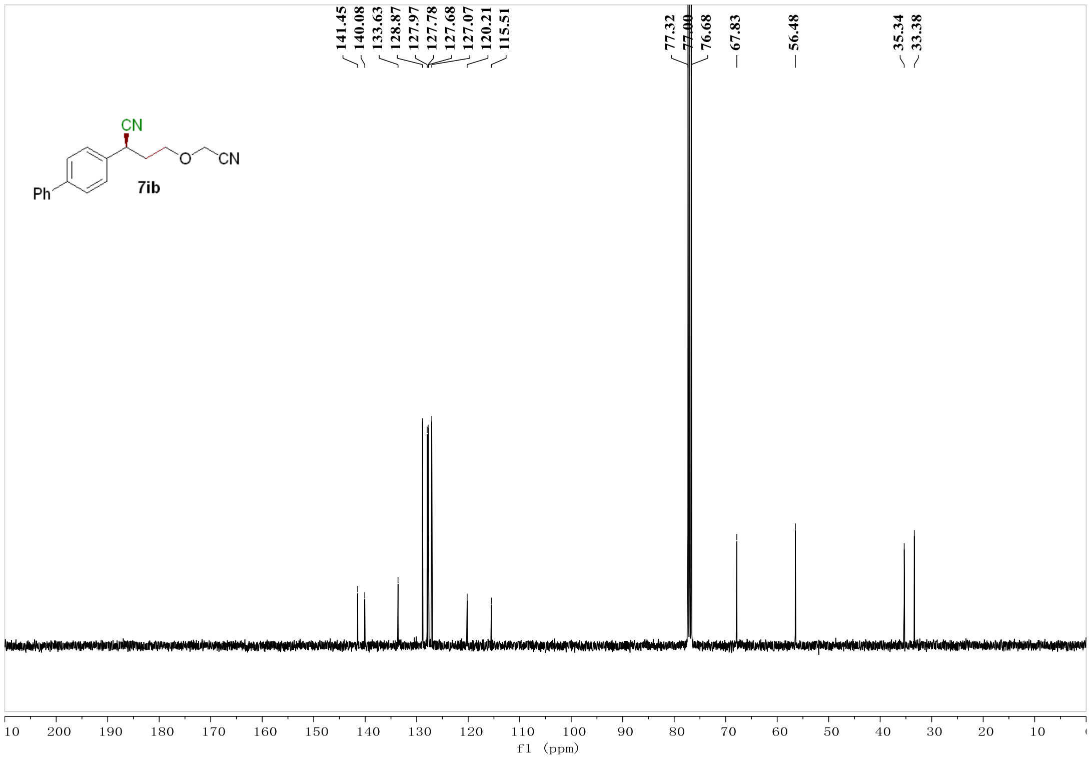

^{1}H NMR (400 MHz, CDCl₃) and ^{13}C NMR (100 MHz, CDCl₃) spectra of substrate 7ic

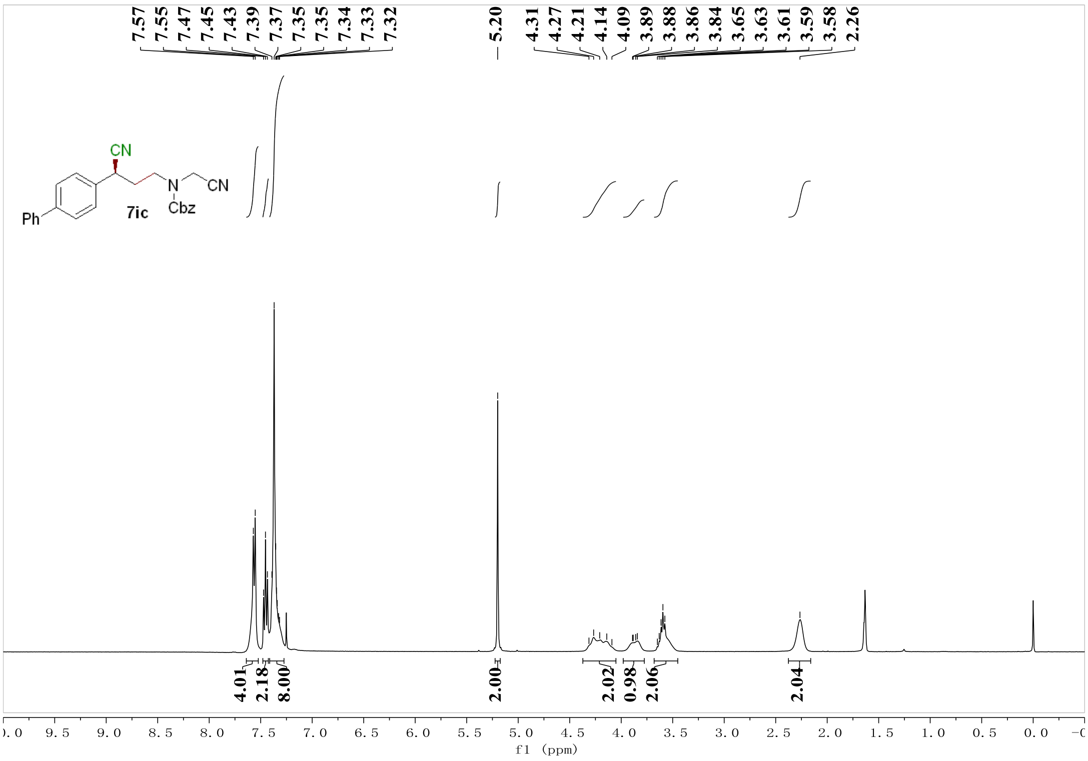

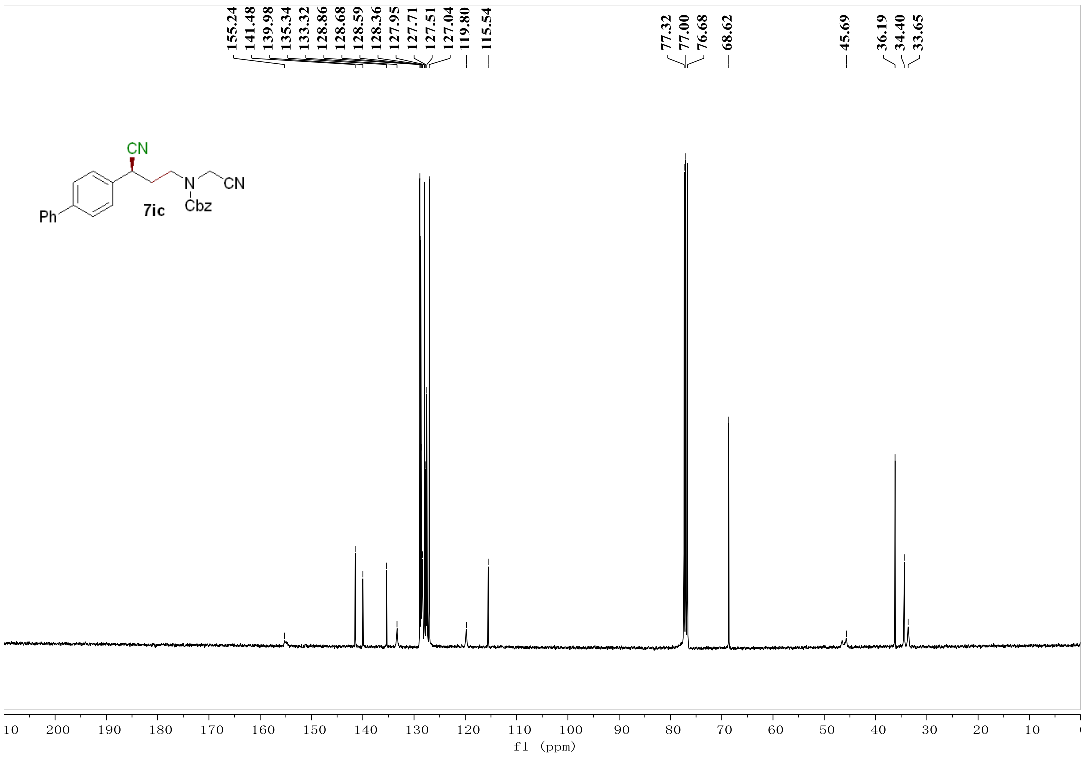

^{1}H NMR (400 MHz, CDCl3) and ^{13}C NMR (100 MHz, CDCl3) spectra of substrate 7id

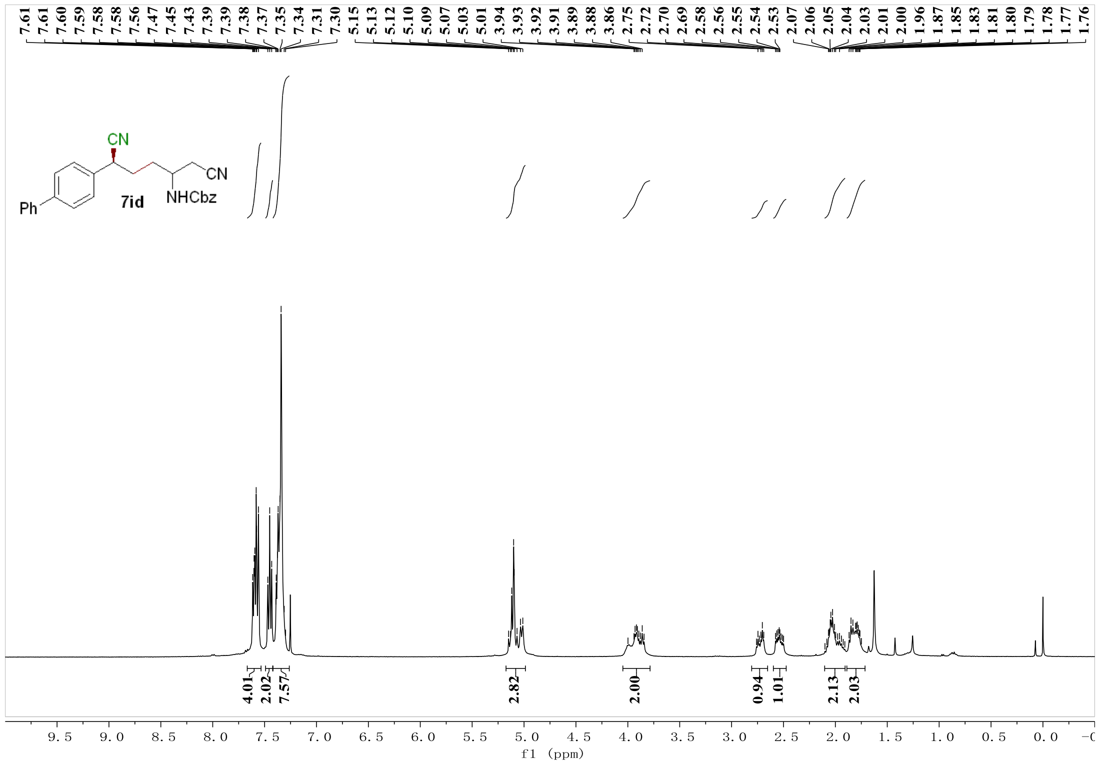

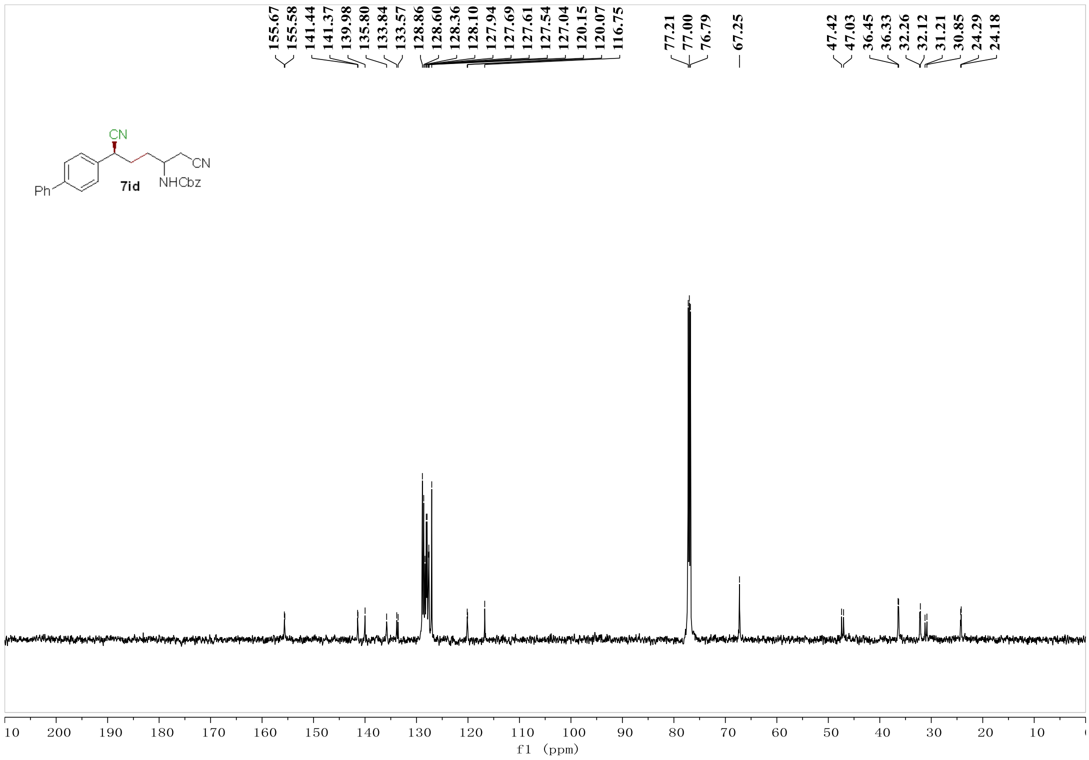

^{1}H NMR (400 MHz, $CDCl_3$) and ^{13}C NMR (100 MHz, $CDCl_3$) spectra of substrate 7ie

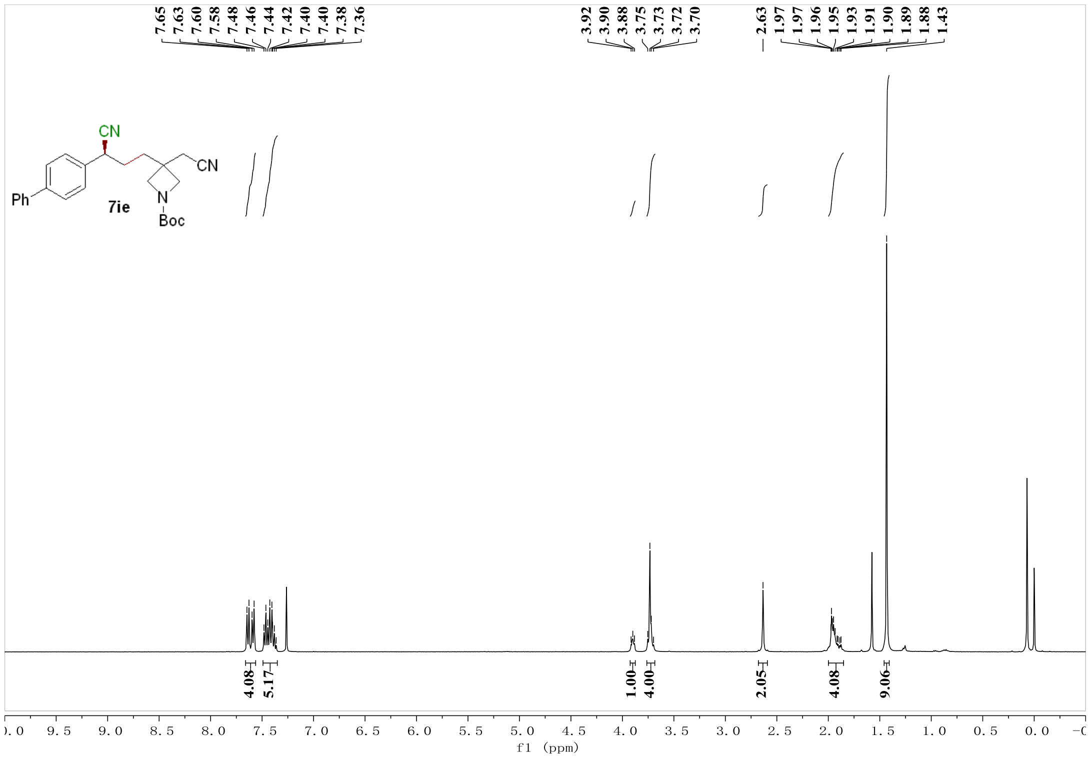

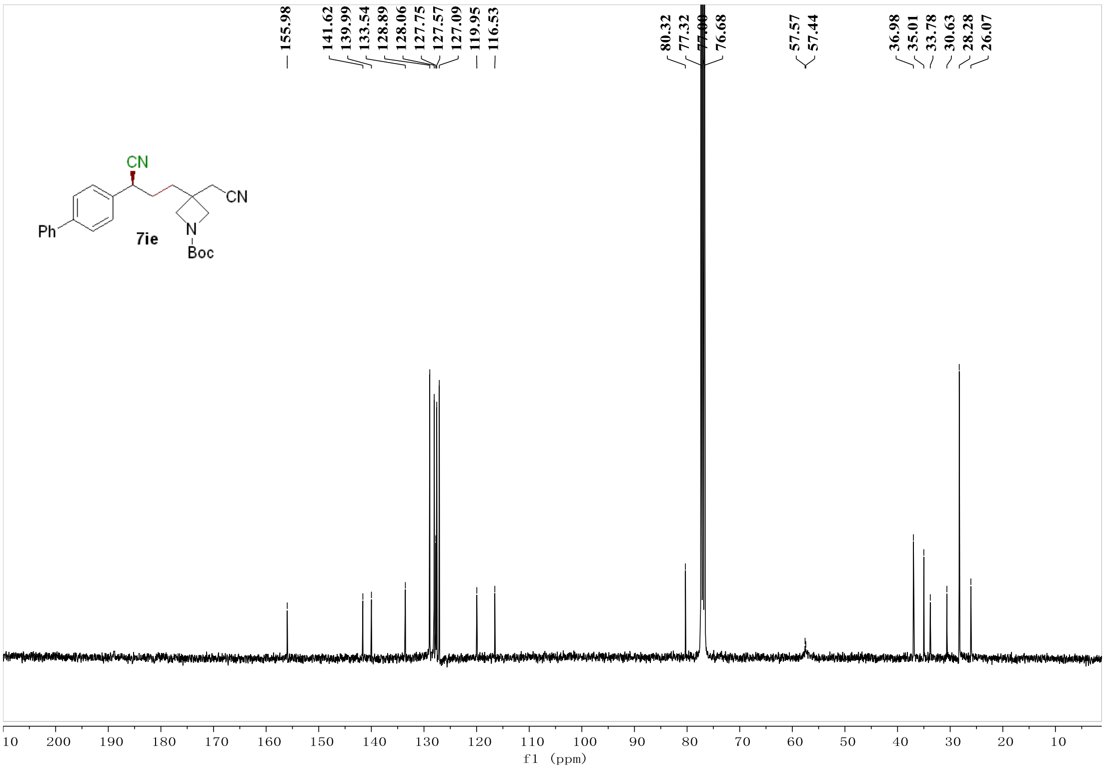

^{1}H NMR (400 MHz, $CDCl_3$) and ^{13}C NMR (100 MHz, $CDCl_3$) spectra of substrate 7if

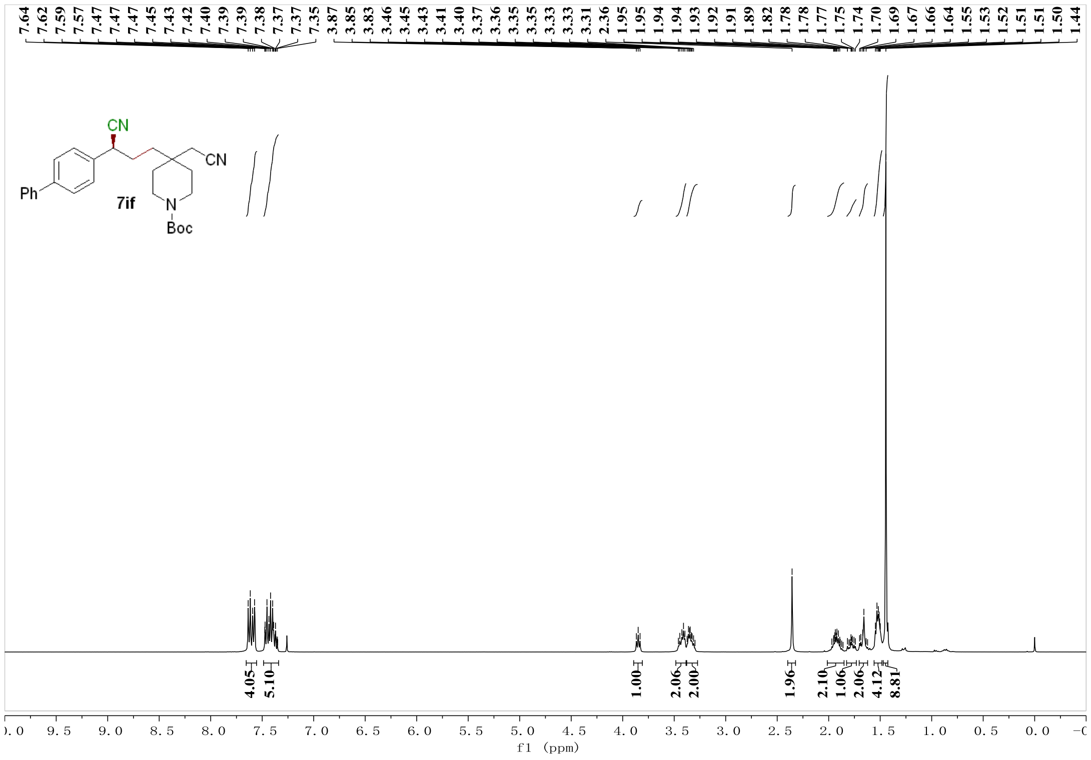

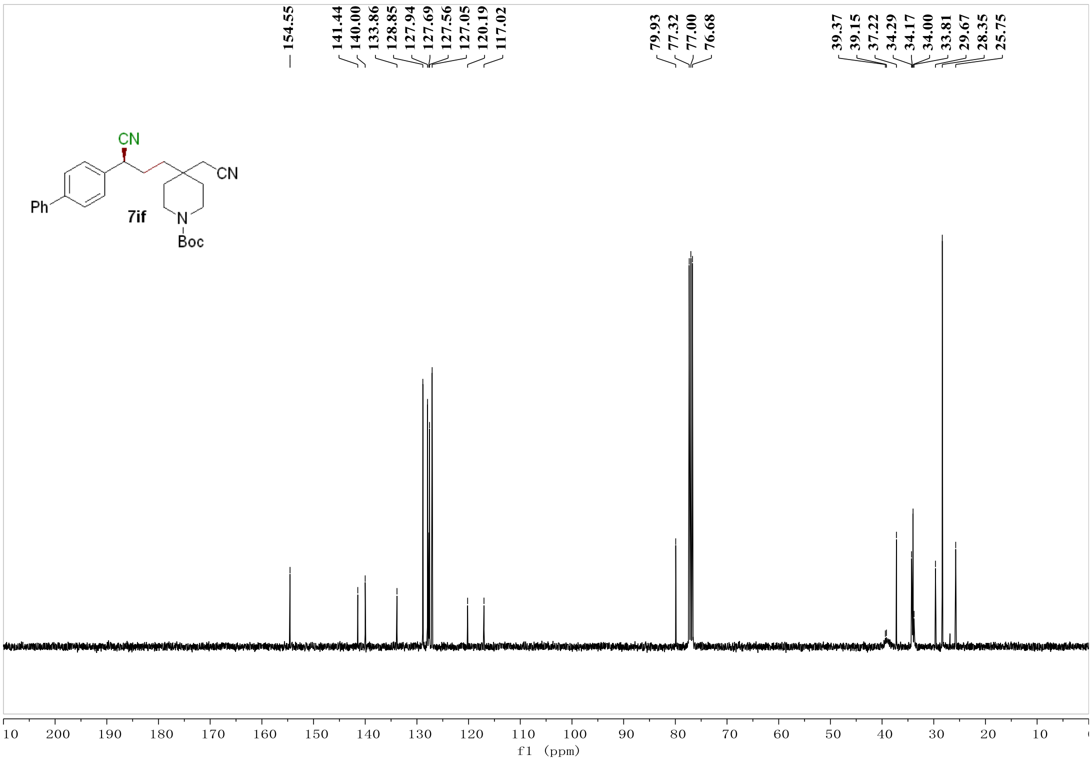

^{1}H NMR (400 MHz, CDCl$_3$) and ^{13}C NMR (100 MHz, CDCl$_3$) spectra of substrate 7ig

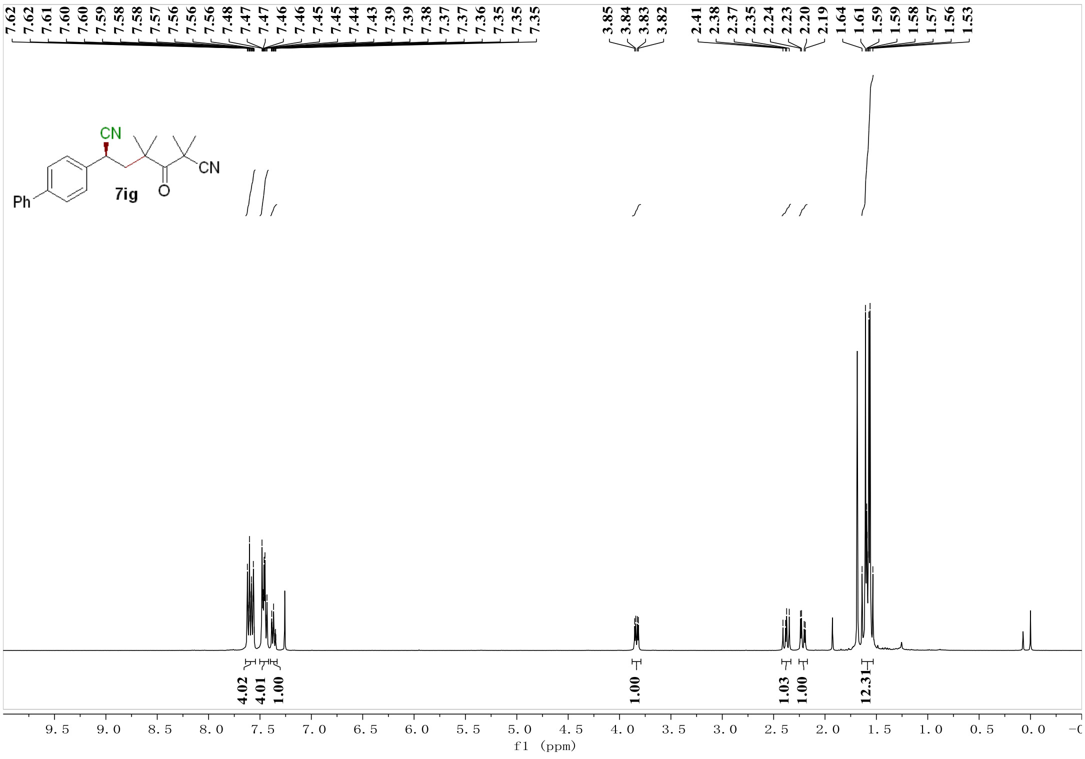

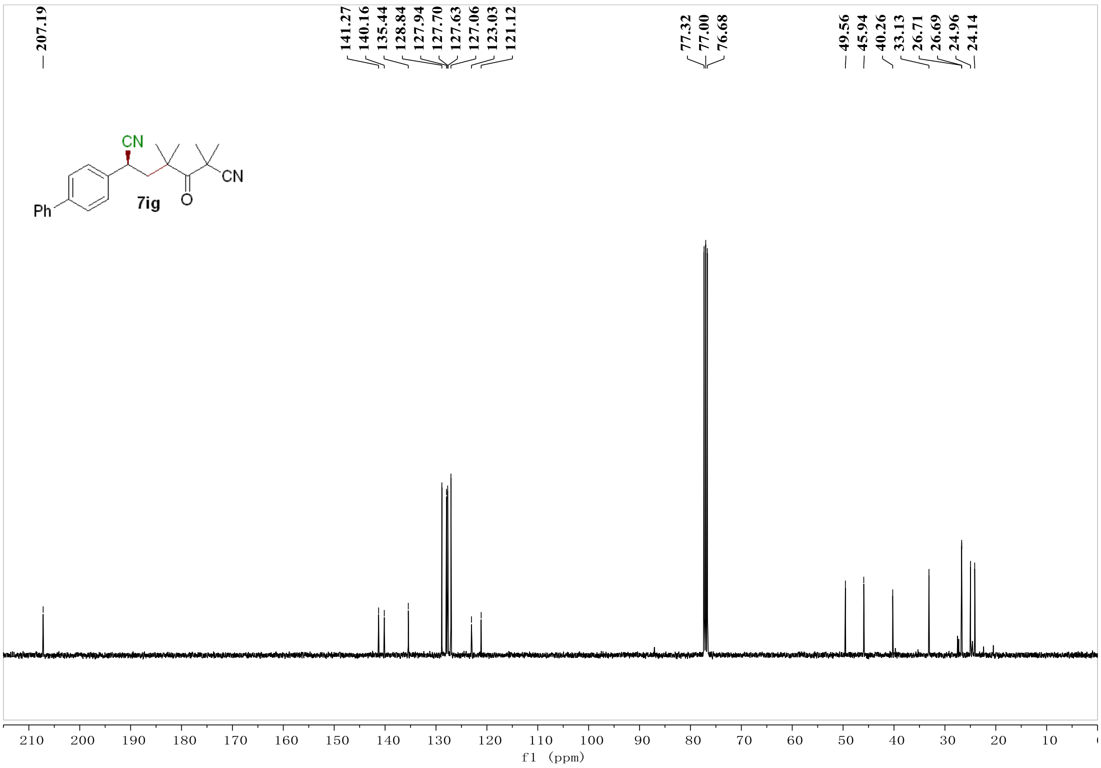

^{1}H NMR (400 MHz, $CDCl_3$) and ^{13}C NMR (100 MHz, $CDCl_3$) spectra of substrate 8

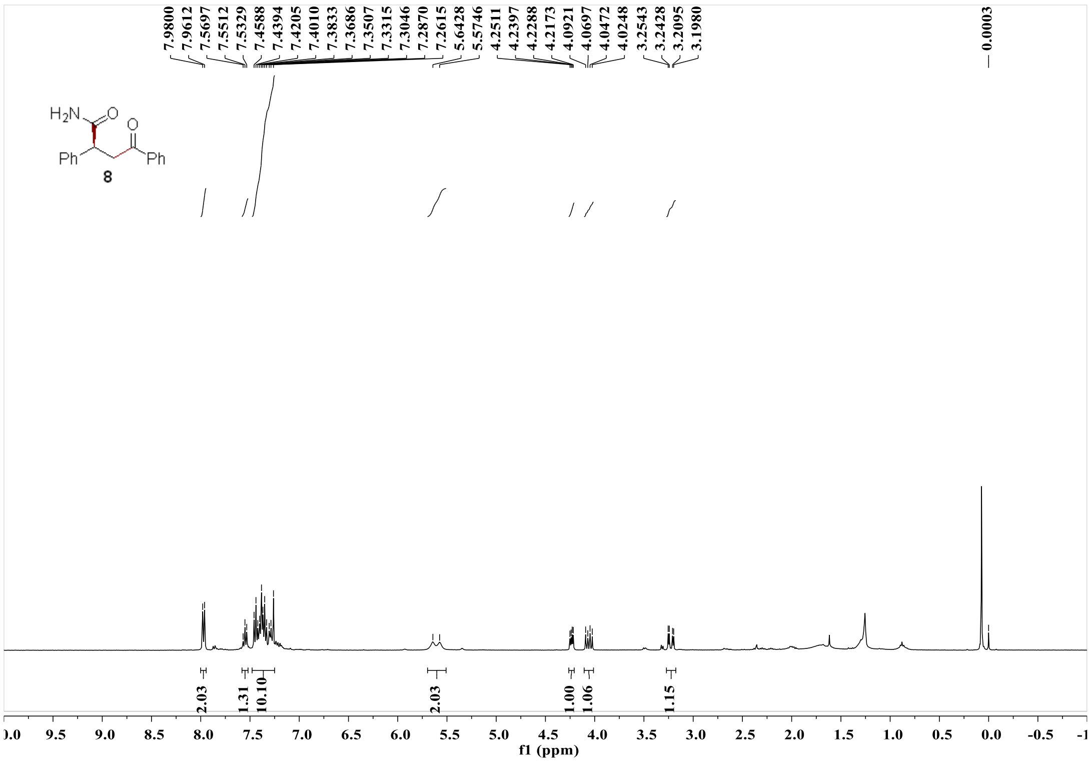

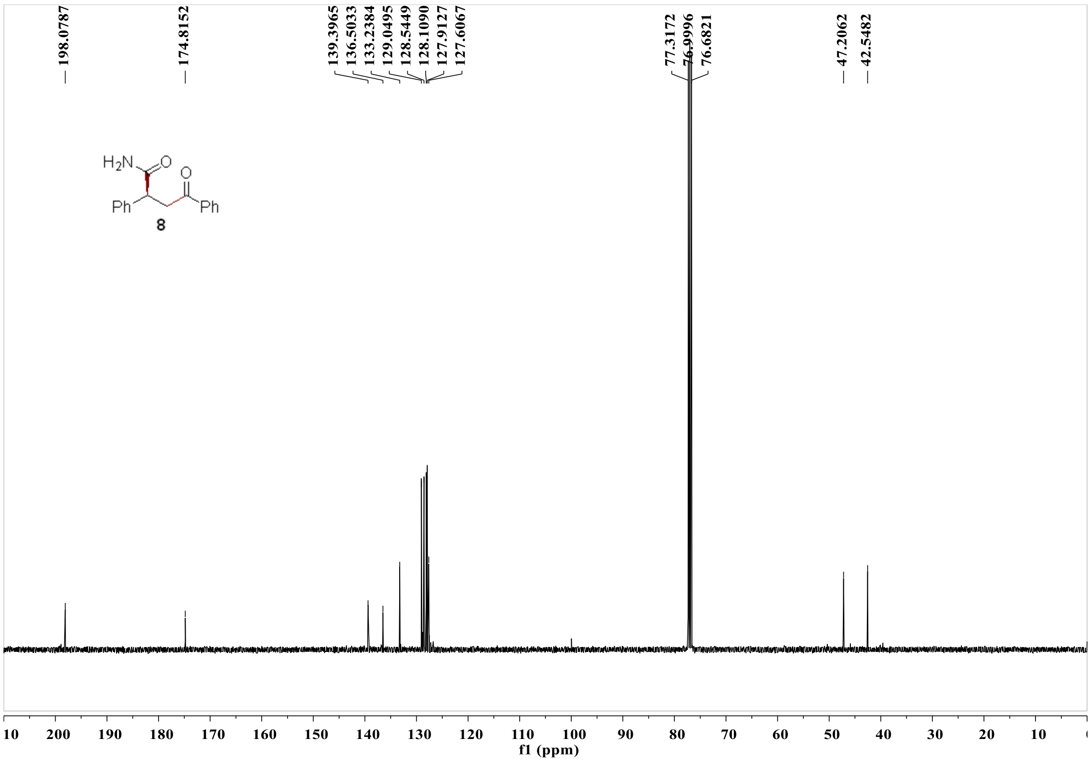

^{1}H NMR (400 MHz, DMSO-d$_6$) and ^{13}C NMR (100 MHz, DMSO-d$_6$) spectra of substrate 9

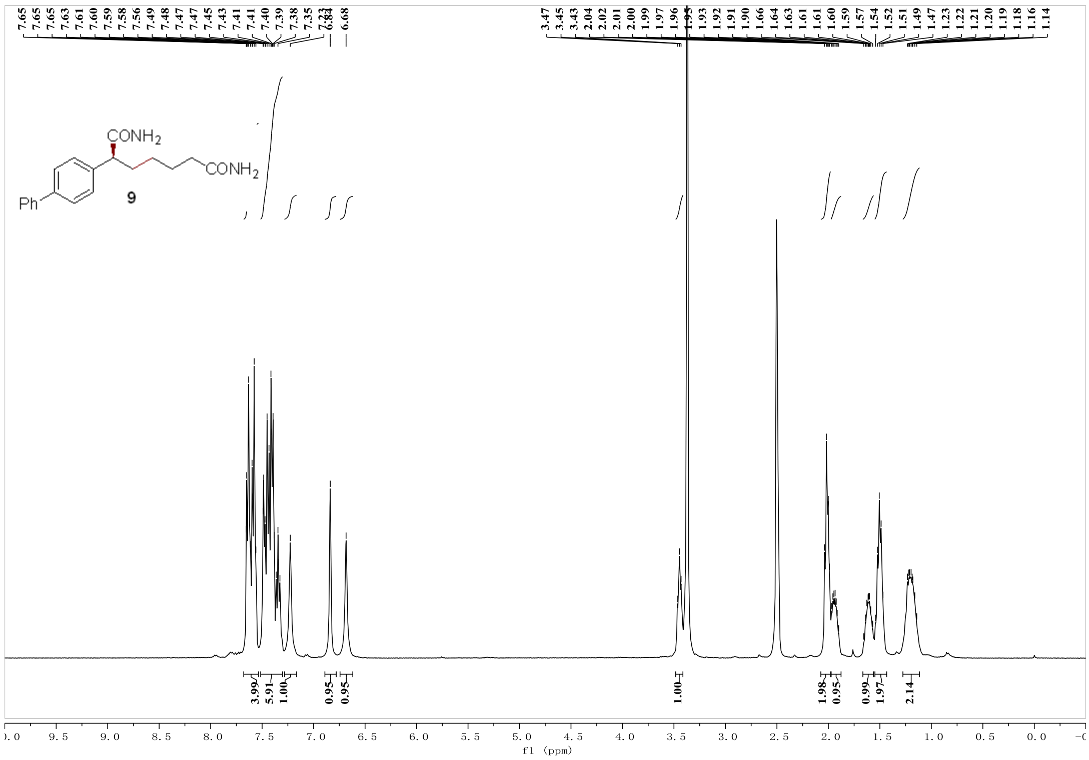

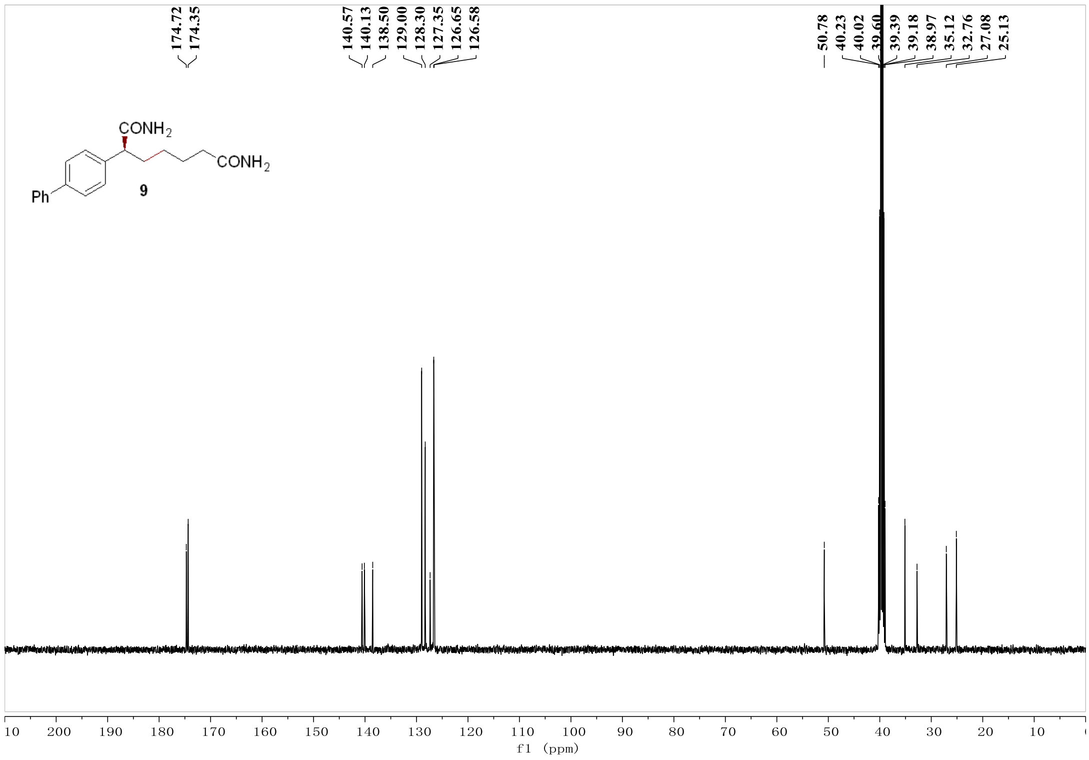

^{1}H NMR (400 MHz, CDCl$_3$) and ^{13}C NMR (100 MHz, CDCl$_3$) spectra of substrate 10

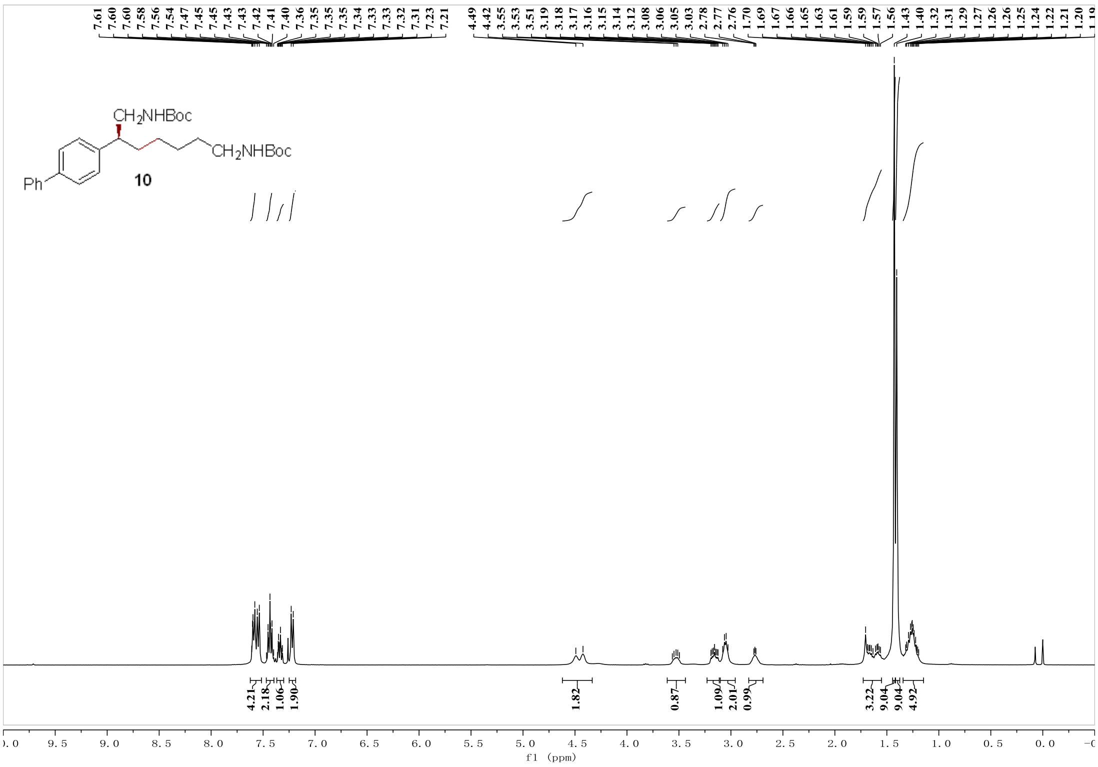

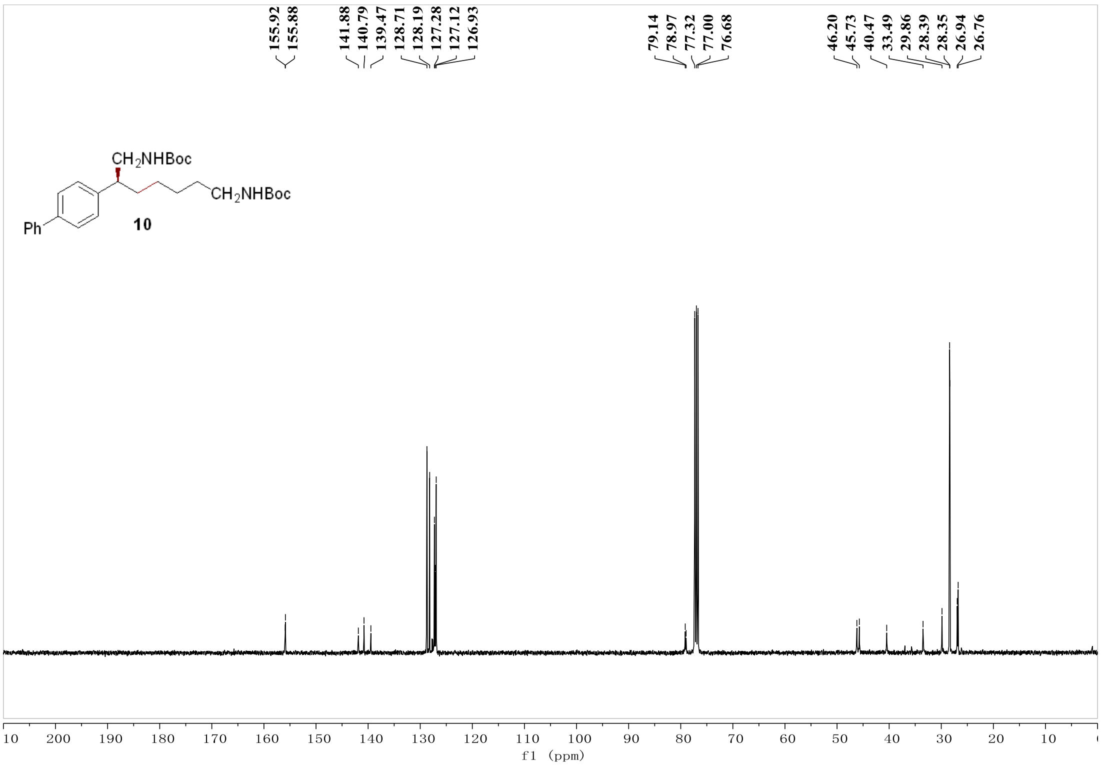

^{1}H NMR (400 MHz, $CDCl_3$) and ^{13}C NMR (100 MHz, $CDCl_3$) spectra of substrate 11

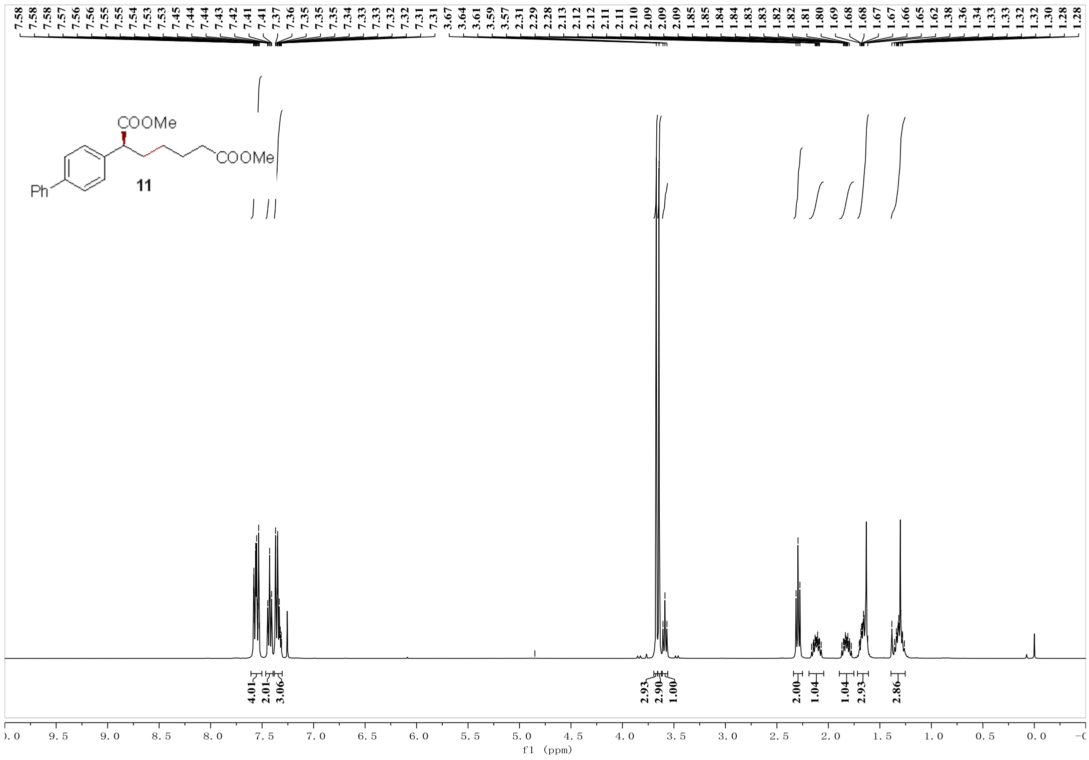

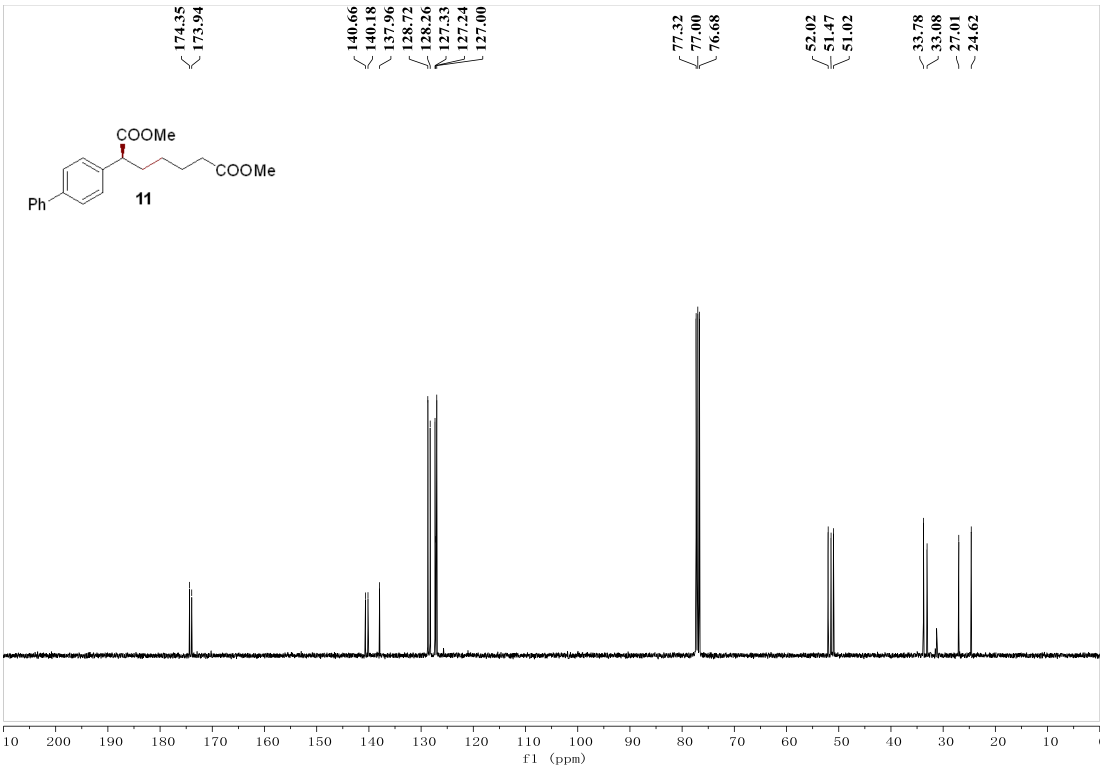

^{1}H NMR (400 MHz, $CDCl_3$) and ^{13}C NMR (100 MHz, $CDCl_3$) spectra of substrate 12

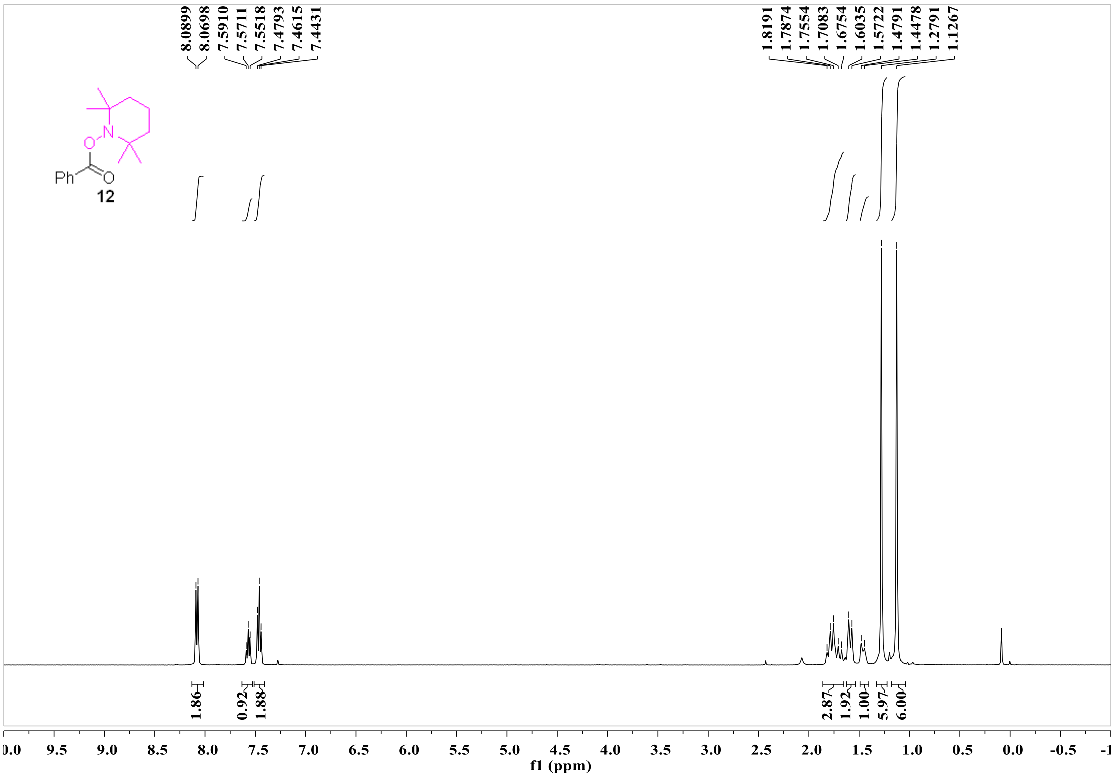

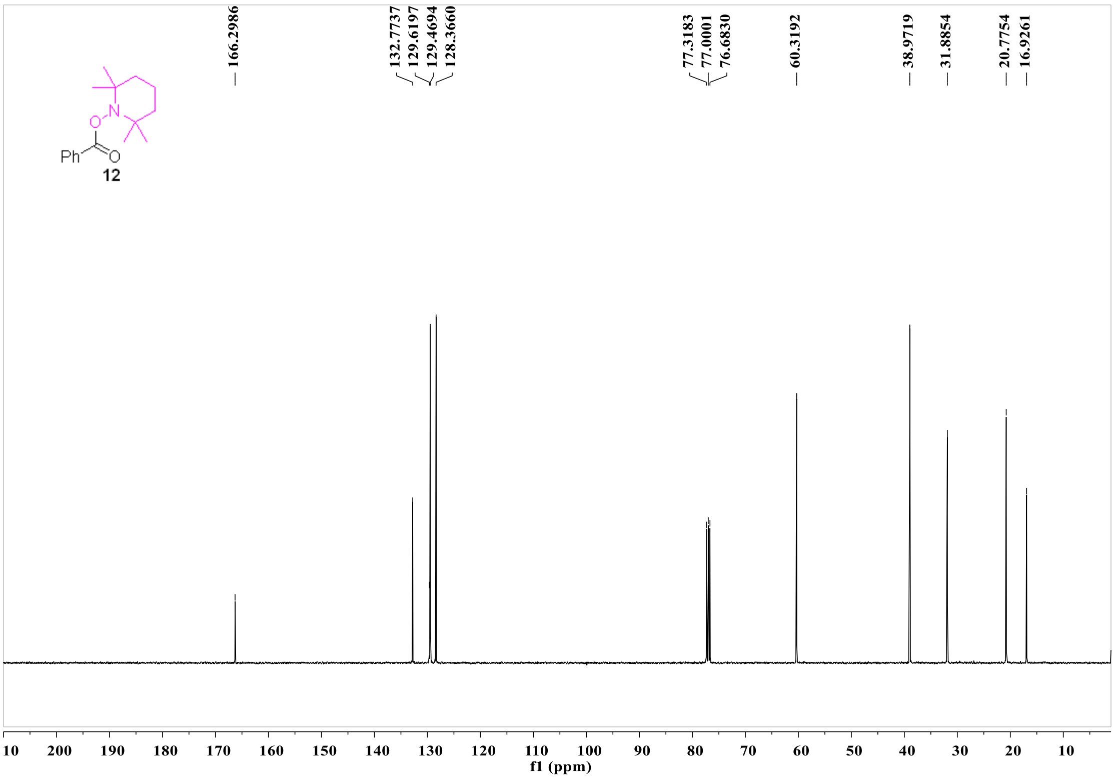

^{1}H NMR (400 MHz, $CDCl_3$) and ^{13}C NMR (100 MHz, $CDCl_3$) spectra of substrate 14

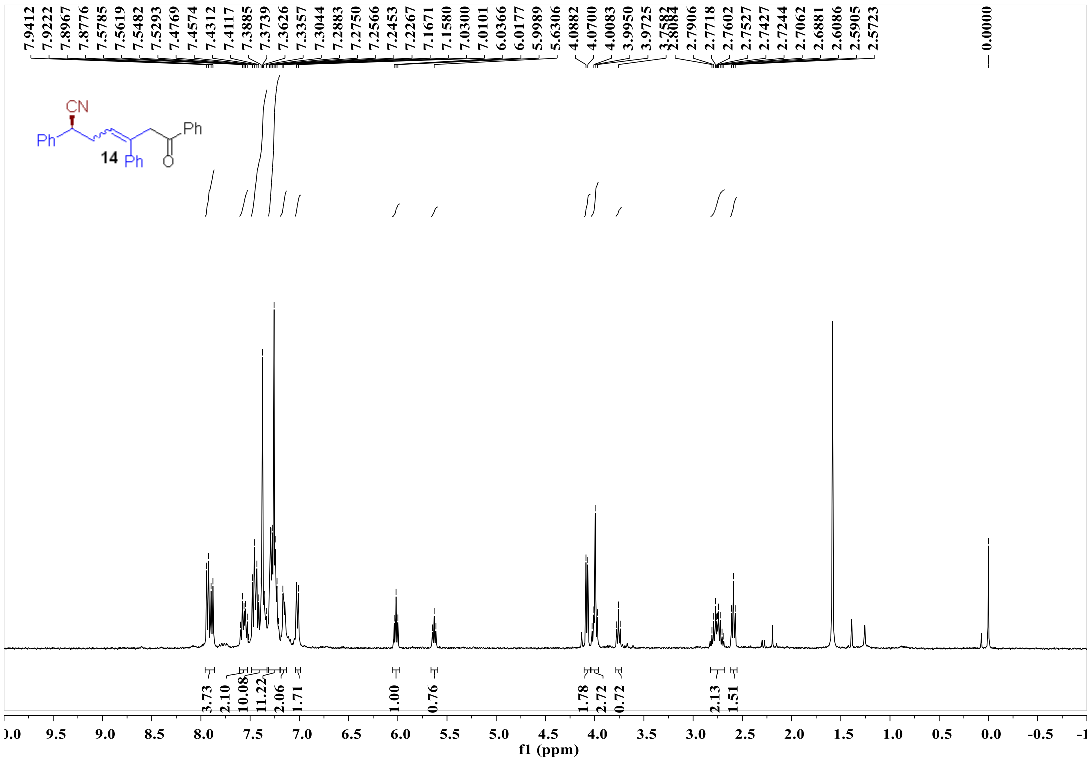

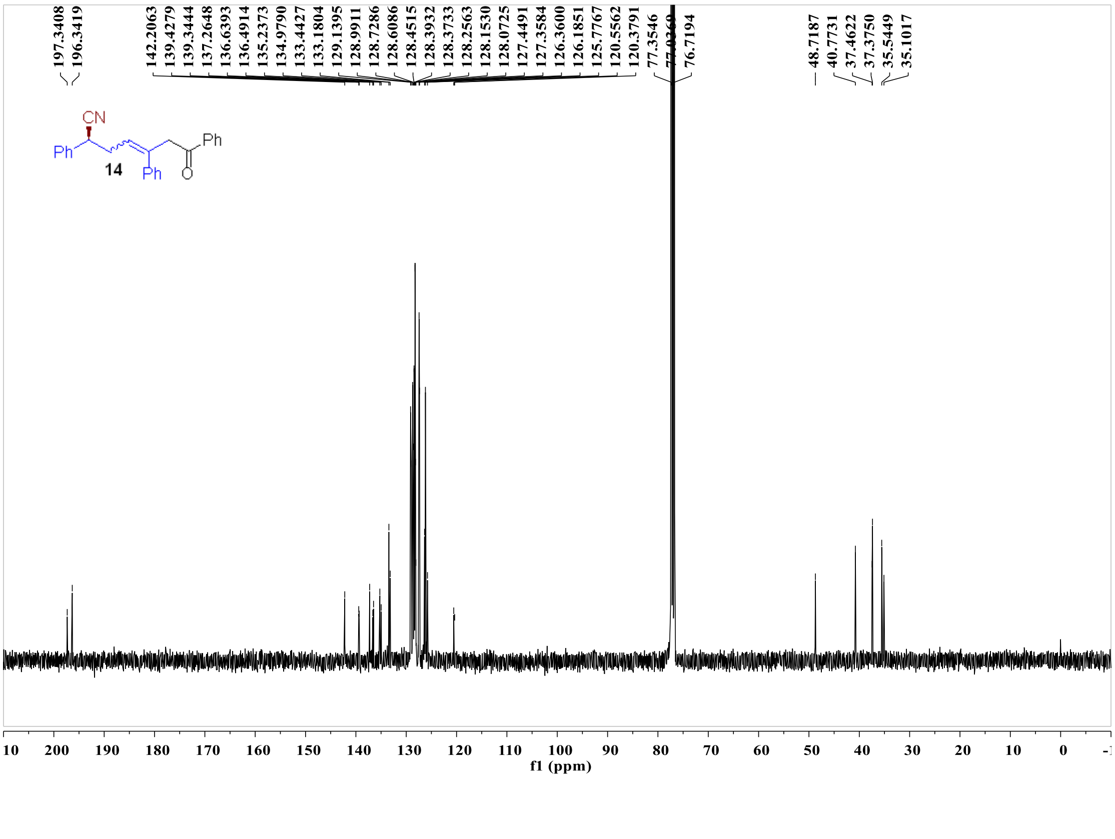

^{1}H-^{1}H NOESY of compound 14

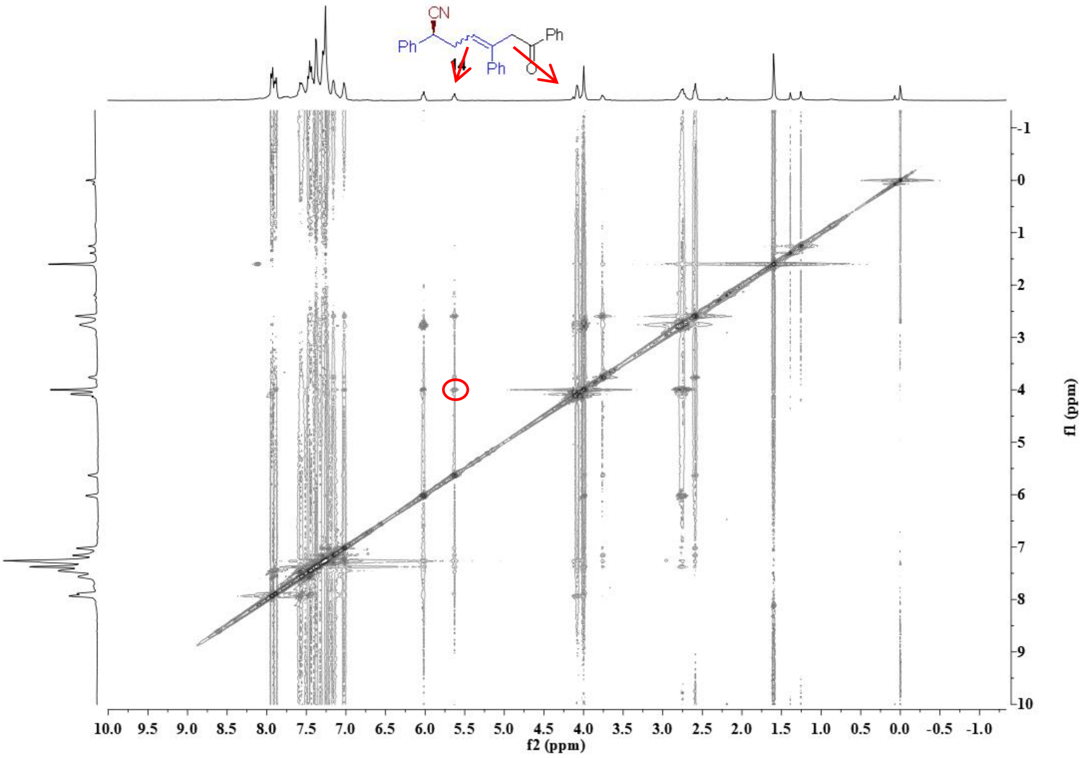

^{1}H NMR (400 MHz, $CDCl_3$) and ^{13}C NMR (100 MHz, $CDCl_3$) spectra of substrate 15

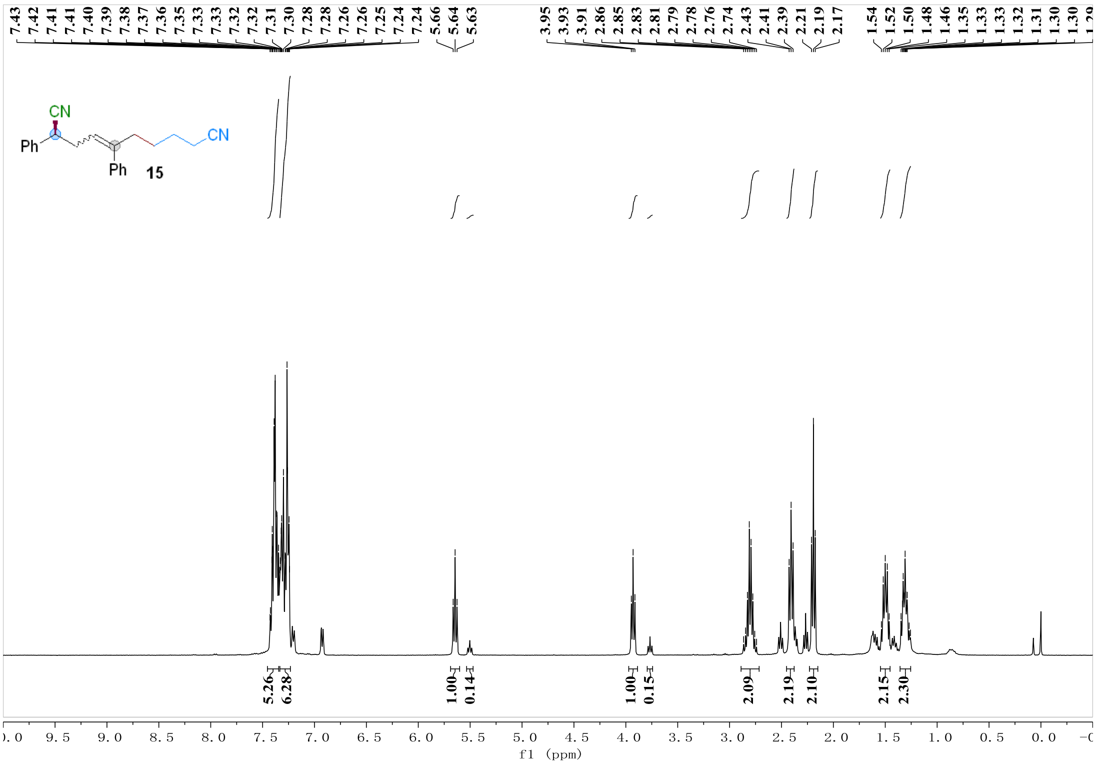

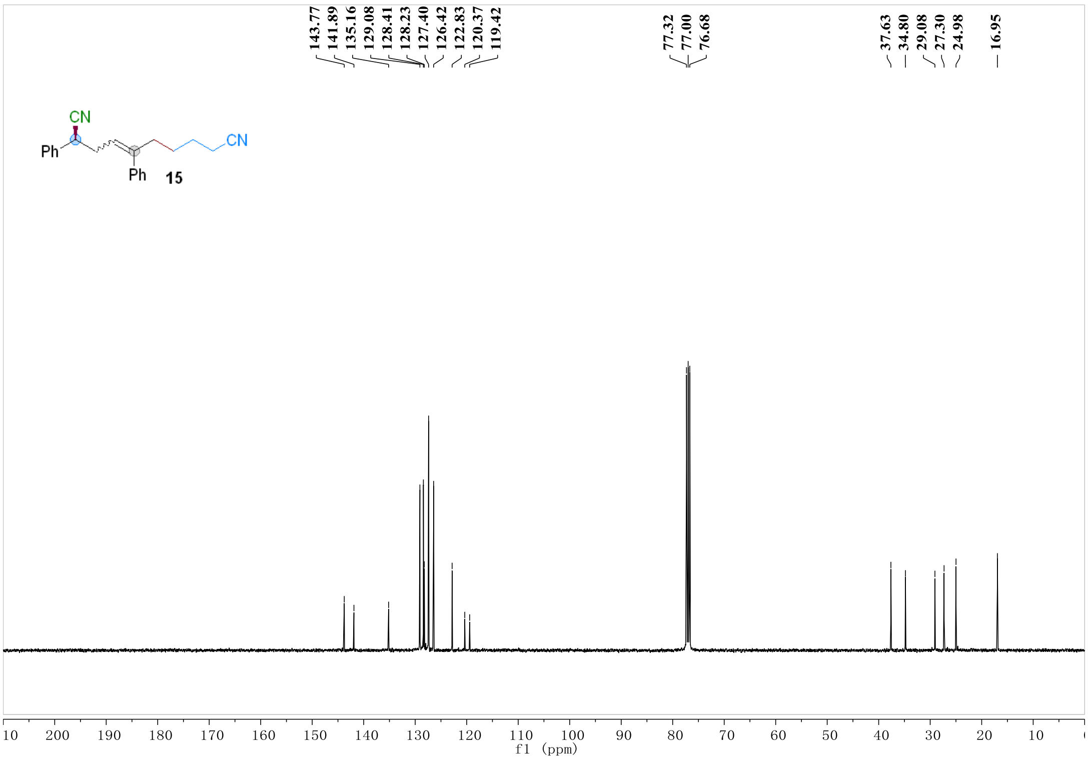

^{1}H-^{1}H NOESY of compound 15

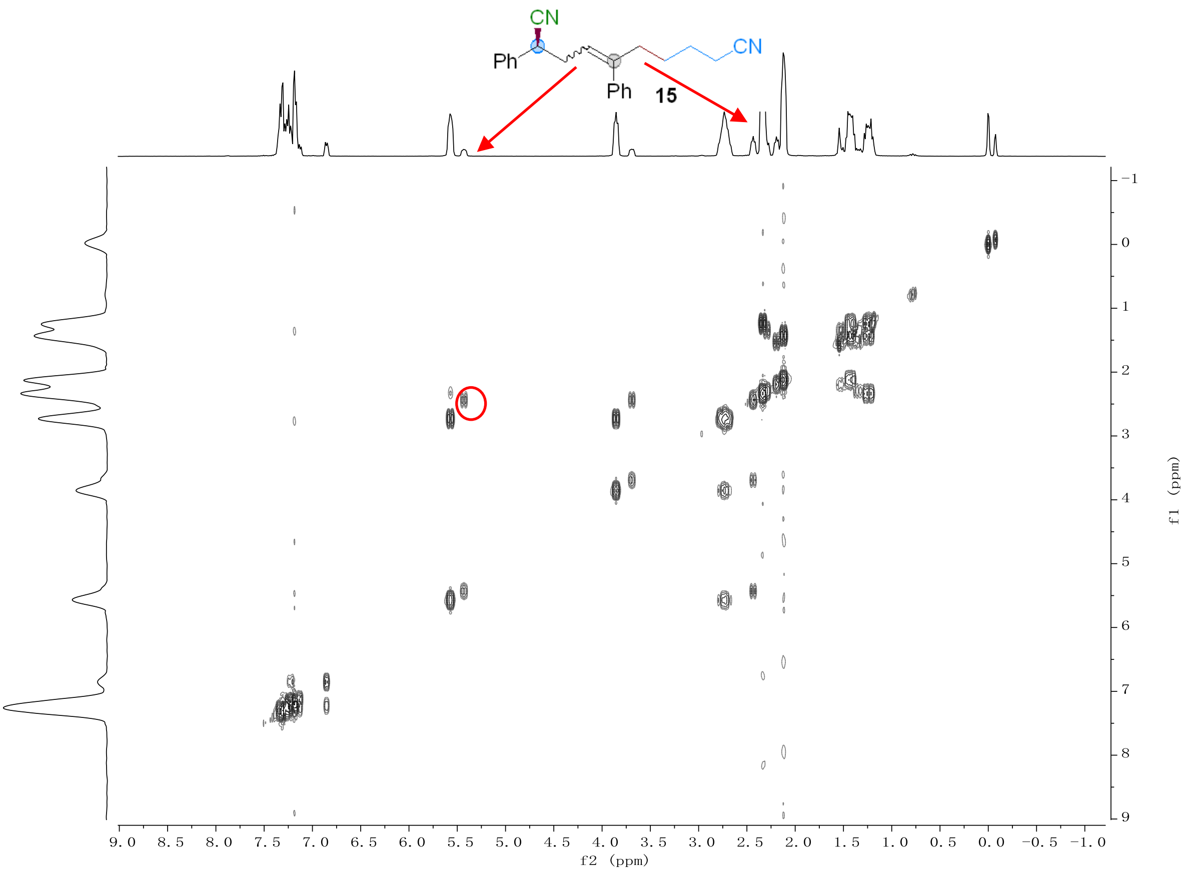

9. Copies of HPLC Spectra

Chiral HPLC spectrum of racemic 6aa

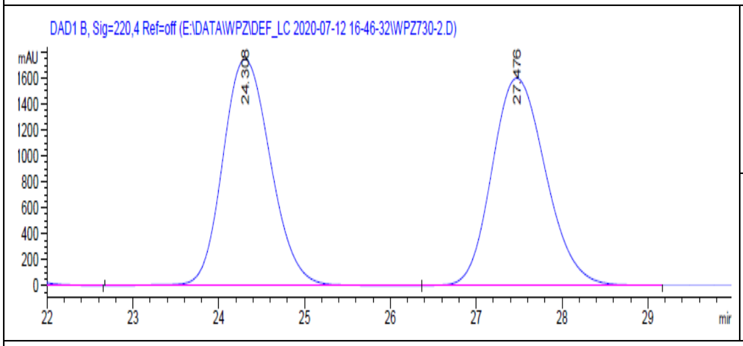

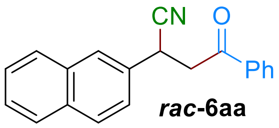

rac-6aa

Peak #	RetTime [min]	Type	Width [min]	Area mAU *s	Height [mAU]	Area %
1	24.308	BB	0.5740	9554.77930	258.05469	49.5939
2	27.476	BB	0.6532	9711.25098	230.66913	50.4061

Chiral HPLC spectrum of 6aa

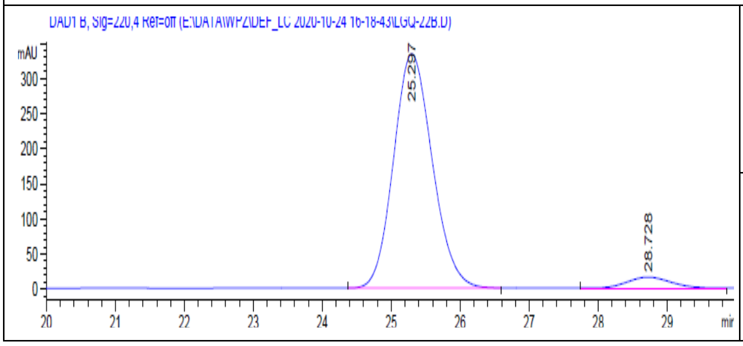

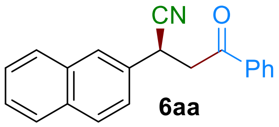

6aa

Peak #	RetTime [min]	Type	Width [min]	Area mAU *s	Height [mAU]	Area %
1	25.297	BB	0.6050	1.30047e4	333.43539	94.9117
2	28.728	BB	0.5663	697.18970	15.81400	5.0883

Chiral HPLC spectrum of racemic 6ba

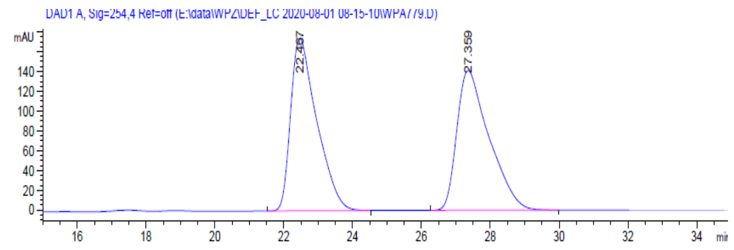

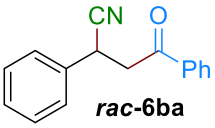

rac-6ba

Peak #	RetTime [min]	Type	Width [min]	Area mAU *s	Height [mAU]	Area %
1	22.467	BB	0.7664	9167.88672	174.07697	49.9435
2	27.359	BB	0.9482	9188.63574	141.02007	50.0565

Chiral HPLC spectrum of racemic 6ba

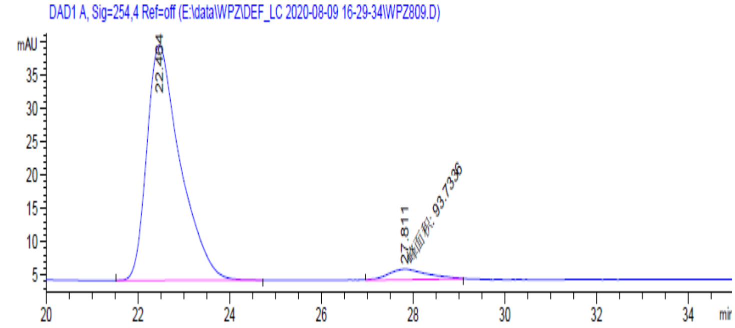

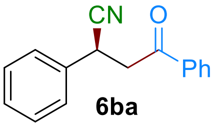

6ba

Peak #	RetTime [min]	Type	Width [min]	Area mAU *s	Height [mAU]	Area %
1	22.464	BB	0.7462	1782.92896	35.24311	95.0053
2	27.811	MM	0.9926	93.73359	1.57389	4.9947

Chiral HPLC spectrum of racemic 6ca

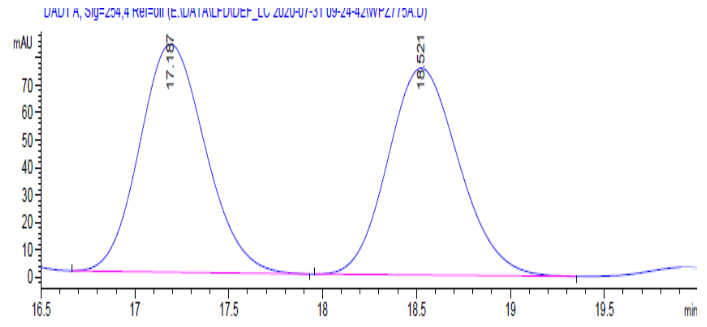

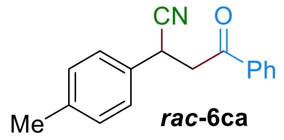

***rac*-6ca**

Peak #	RetTime [min]	Type	Width [min]	Area mAU *s	Height [mAU]	Area %
1	17.187	BB	0.3809	2027.86230	83.04923	50.5308
2	18.521	BB	0.4094	1985.26172	75.34254	49.4692

Chiral HPLC spectrum of 6ca

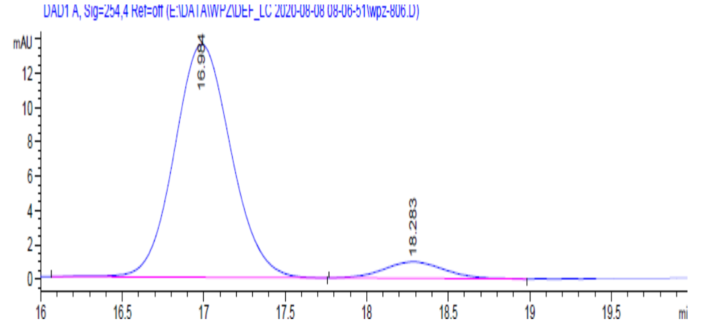

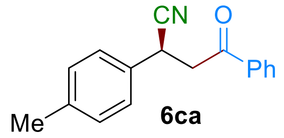

6ca

Peak #	RetTime [min]	Type	Width [min]	Area mAU *s	Height [mAU]	Area %
1	16.984	BB	0.3751	327.63681	13.59715	93.0152
2	18.283	BB	0.4019	24.60327	9.63685e-1	6.9848

Chiral HPLC spectrum of racemic 6da

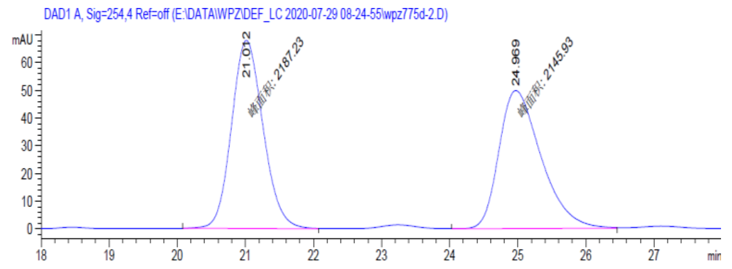

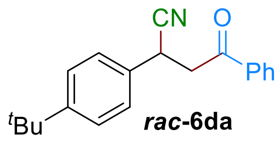

***rac*-6da**

Peak #	RetTime [min]	Type	Width [min]	Area mAU *s	Height [mAU]	Area %
1	21.012	MM	0.5366	2187.23486	67.93749	50.4766
2	24.969	MM	0.7170	2145.93164	49.87918	49.5234

Chiral HPLC spectrum of 6da

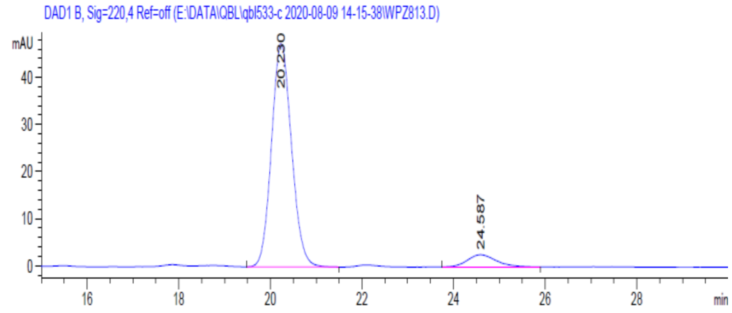

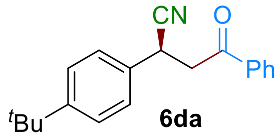

6da

Peak #	RetTime [min]	Type	Width [min]	Area mAU *s	Height [mAU]	Area %
1	20.230	BB	0.4707	1438.43433	47.31816	92.8126
2	24.587	BB	0.5564	111.39236	2.56609	7.1874

Chiral HPLC spectrum of racemic 6ea

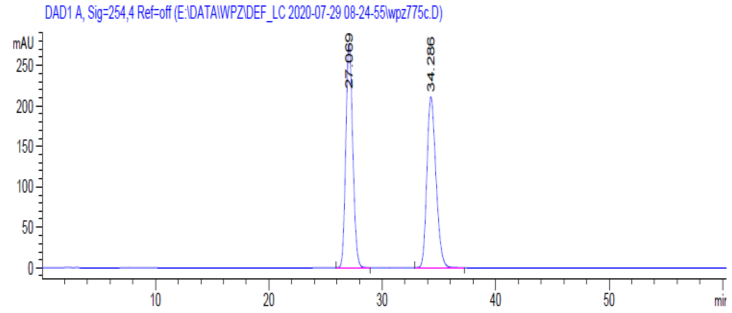

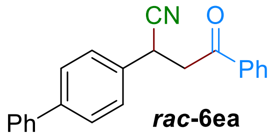

***rac*-6ea**

Peak #	RetTime [min]	Type	Width [min]	Area mAU *s	Height [mAU]	Area %
1	27.069	BB	0.6636	1.18071e4	276.83301	50.0169
2	34.286	BB	0.8641	1.17991e4	211.09738	49.9831

Chiral HPLC spectrum of 6ea

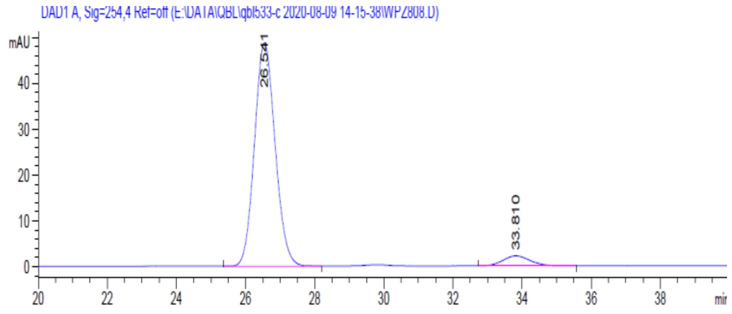

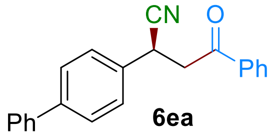

6ea

Peak #	RetTime [min]	Type	Width [min]	Area mAU *s	Height [mAU]	Area %
1	26.541	BB	0.6464	2020.57935	48.66517	94.4165
2	33.810	BB	0.7489	119.49132	2.16157	5.5835

Chiral HPLC spectrum of racemic 6fa

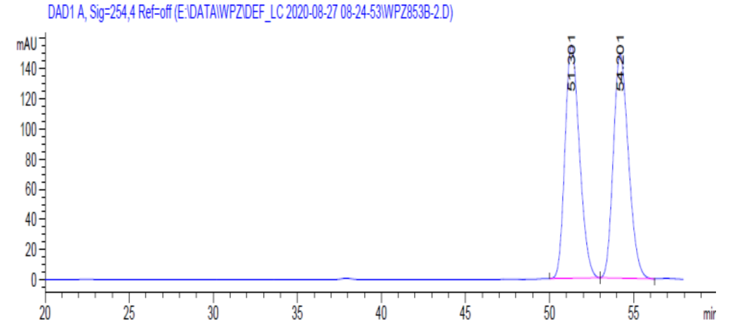

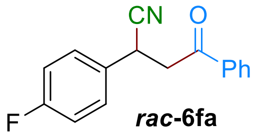

***rac*-6fa**

Peak #	RetTime [min]	Type	Width [min]	Area mAU *s	Height [mAU]	Area %
1	51.301	BB	0.9269	9265.74805	154.66864	49.9441
2	54.201	BB	0.9726	9286.48145	148.28609	50.0559

Chiral HPLC spectrum of 6fa

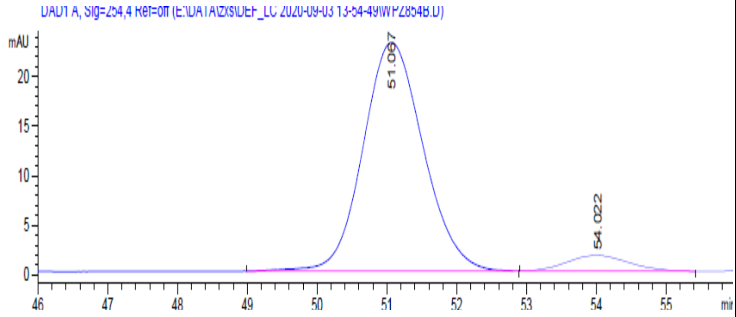

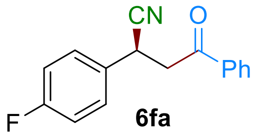

6fa

Peak #	RetTime [min]	Type	Width [min]	Area mAU *s	Height [mAU]	Area %
1	51.067	BB	0.9193	1381.23779	23.04244	93.4257
2	54.022	BB	0.7303	97.19601	1.58744	6.5743

Chiral HPLC spectrum of racemic 6ga

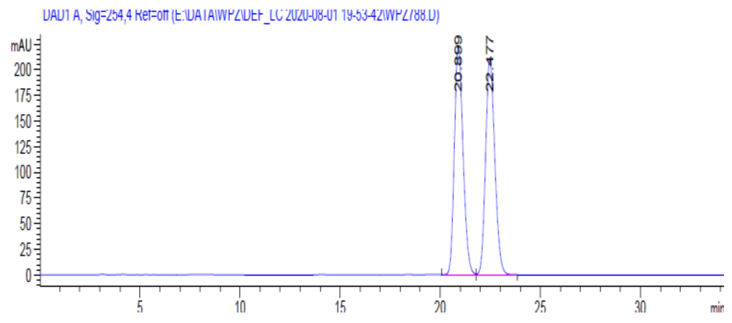

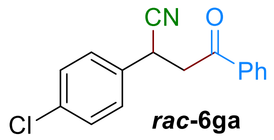

***rac*-6ga**

Peak #	RetTime [min]	Type	Width [min]	Area mAU *s	Height [mAU]	Area %
1	20.899	BV	0.4678	6727.12744	223.07492	49.9916
2	22.477	VB	0.4971	6729.39697	210.42232	50.0084

Chiral HPLC spectrum of racemic 6ga

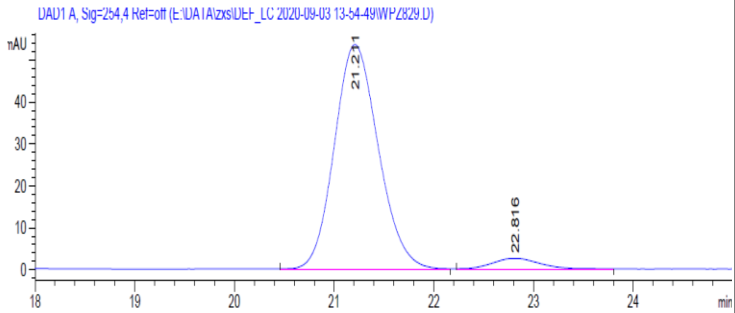

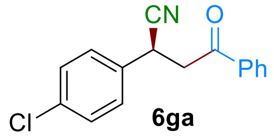

6ga

Peak #	RetTime [min]	Type	Width [min]	Area mAU *s	Height [mAU]	Area %
1	21.211	BB	0.4751	1638.82446	53.54733	95.1997
2	22.816	BB	0.4636	82.63541	2.53999	4.8003

Chiral HPLC spectrum of racemic 6ha

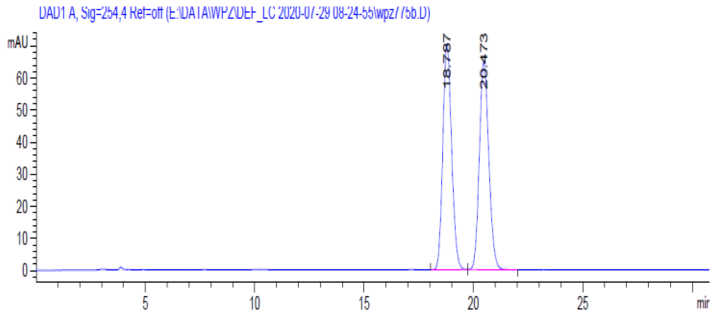

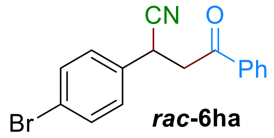

***rac*-6ha**

Peak #	RetTime [min]	Type	Width [min]	Area mAU *s	Height [mAU]	Area %
1	18.787	BB	0.4310	1962.59094	70.44279	50.0166
2	20.473	BB	0.4709	1961.28845	64.83901	49.9834

Chiral HPLC spectrum of 6ha

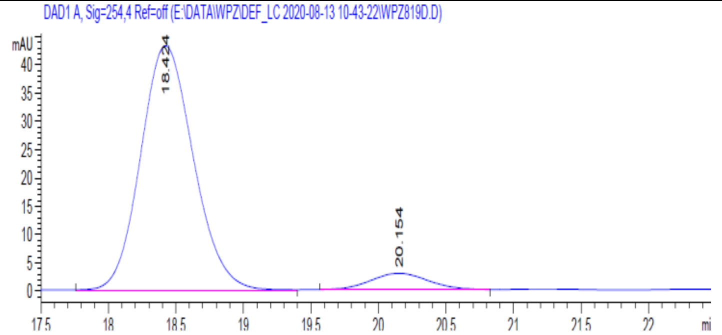

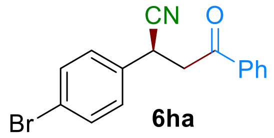

6ha

Peak #	RetTime [min]	Type	Width [min]	Area mAU *s	Height [mAU]	Area %
1	18.424	BB	0.4201	1168.89392	43.13783	93.2233
2	20.154	BB	0.4096	84.97124	2.88500	6.7767

Chiral HPLC spectrum of racemic 6ia

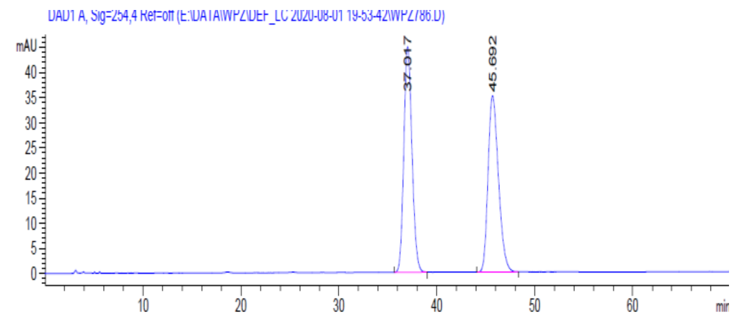

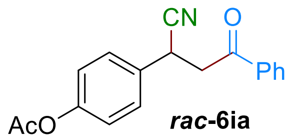

Peak #	RetTime [min]	Type	Width [min]	Area mAU *s	Height [mAU]	Area %
1	37.017	BB	0.8860	2594.39844	44.90539	50.0131
2	45.692	BB	1.1278	2593.04004	35.10540	49.9869

Chiral HPLC spectrum of 6ia

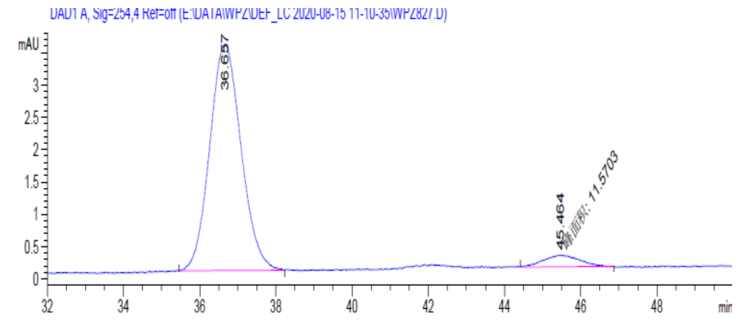

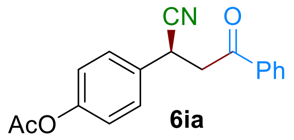

Peak #	RetTime [min]	Type	Width [min]	Area mAU *s	Height [mAU]	Area %
1	36.657	BB	0.6838	200.02803	3.49470	94.5319
2	45.464	MM	1.0669	11.57034	1.80746e-1	5.4681

Chiral HPLC spectrum of racemic 6ja

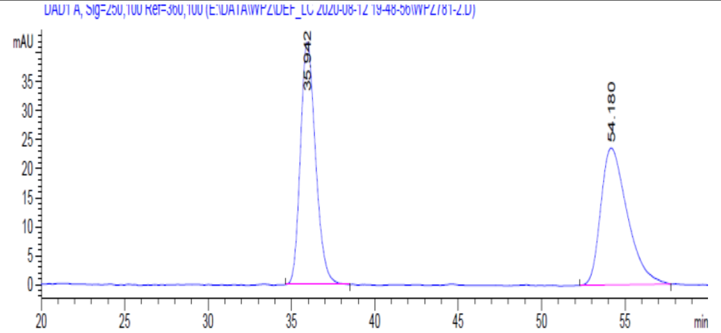

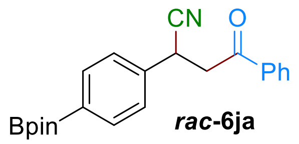

Peak #	RetTime [min]	Type	Width [min]	Area mAU *s	Height [mAU]	Area %
1	35.942	BB	0.9691	2643.64136	41.84273	50.2745
2	54.180	BB	1.3690	2614.77539	23.62958	49.7255

Chiral HPLC spectrum of 6ja

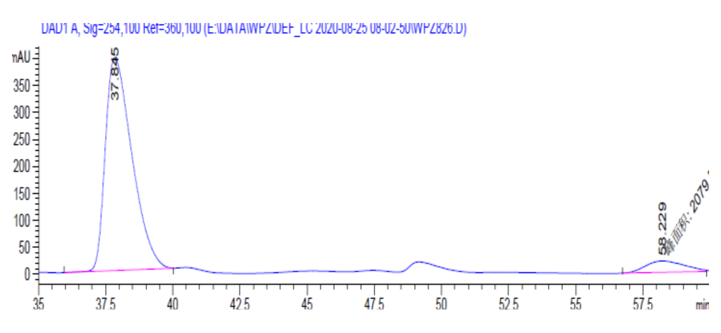

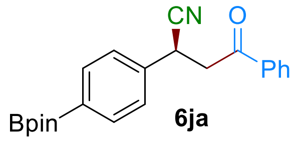

Peak #	RetTime [min]	Type	Width [min]	Area mAU *s	Height [mAU]	Area %
1	37.845	BB	1.0458	2.72401e4	395.36768	92.9072
2	58.229	MM	1.6643	2079.57471	20.82593	7.0928

Chiral HPLC spectrum of racemic 6ka

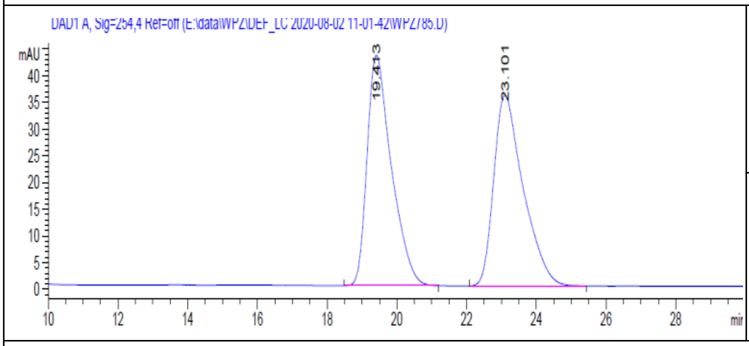

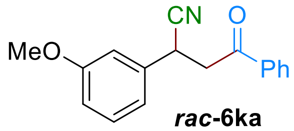

***rac*-6ka**

Peak #	RetTime [min]	Type	Width [min]	Area mAU *s	Height [mAU]	Area %
1	19.413	BB	0.7198	2093.12061	43.15891	49.9546
2	23.101	BB	0.8624	2096.92456	35.74659	50.0454

Chiral HPLC spectrum of 6ka

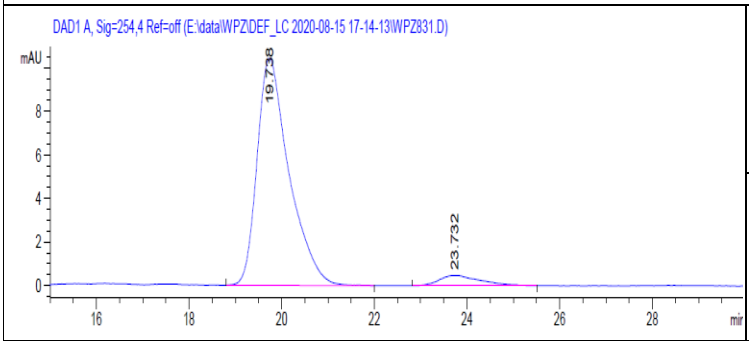

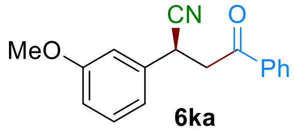

6ka

Peak #	RetTime [min]	Type	Width [min]	Area mAU *s	Height [mAU]	Area %
1	19.738	BB	0.7009	499.11975	10.41907	94.3487
2	23.732	BB	0.7379	29.89604	4.79176e-1	5.6513

Chiral HPLC spectrum of racemic 6la

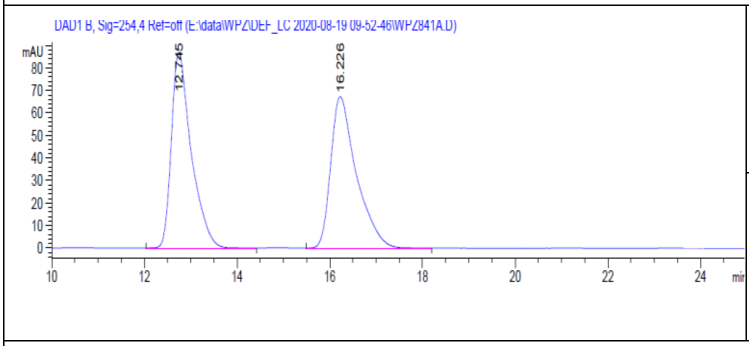

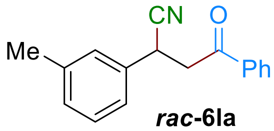

***rac*-6la**

Peak #	RetTime [min]	Type	Width [min]	Area mAU *s	Height [mAU]	Area %
1	12.745	BB	0.4388	2584.83765	86.94409	50.0087
2	16.226	BB	0.5640	2583.94116	67.28686	49.9913

Chiral HPLC spectrum of 6la

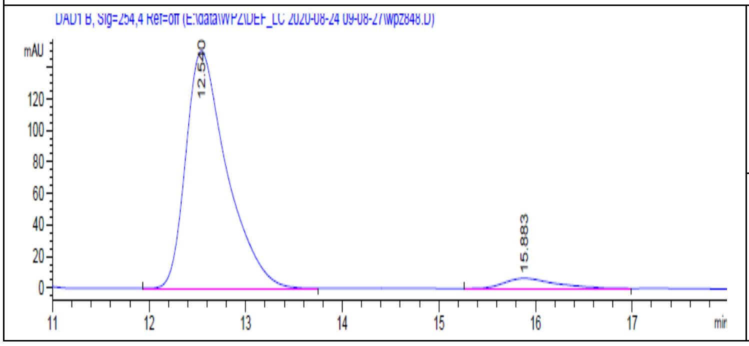

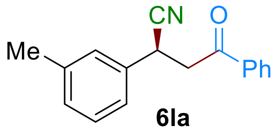

6la

Peak #	RetTime [min]	Type	Width [min]	Area mAU *s	Height [mAU]	Area %
1	12.540	BB	0.4343	4433.42969	150.23985	94.9005
2	15.883	BB	0.5362	238.23175	6.46012	5.0995

Chiral HPLC spectrum of racemic 6ma

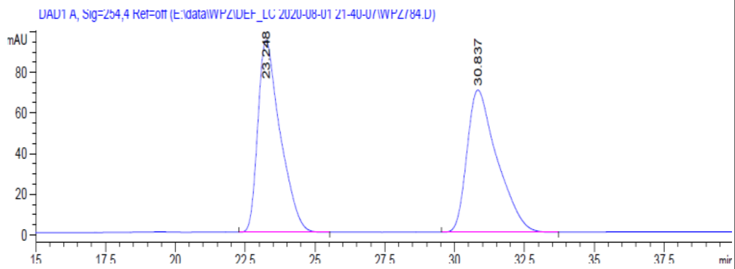

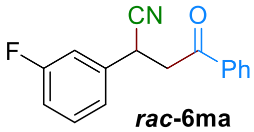

***rac*-6ma**

Peak #	RetTime [min]	Type	Width [min]	Area mAU *s	Height [mAU]	Area %
1	23.248	BB	0.8073	5242.74854	94.49631	50.0024
2	30.837	BB	1.0846	5242.24854	69.92482	49.9976

Chiral HPLC spectrum of 6ma

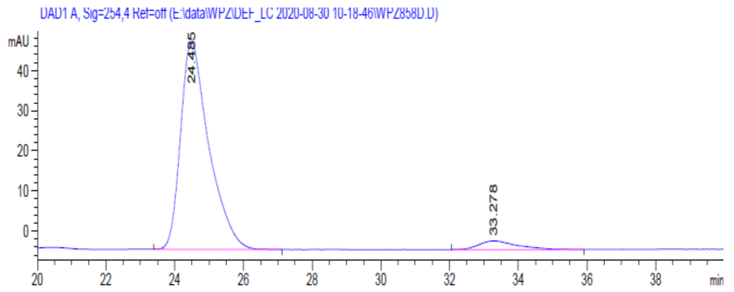

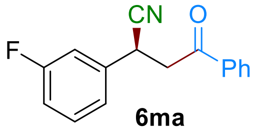

6ma

Peak #	RetTime [min]	Type	Width [min]	Area mAU *s	Height [mAU]	Area %
1	24.485	BB	0.8435	2958.71509	51.71067	94.5972
2	33.278	BB	0.9207	168.98447	2.16948	5.4028

Chiral HPLC spectrum of racemic 6na

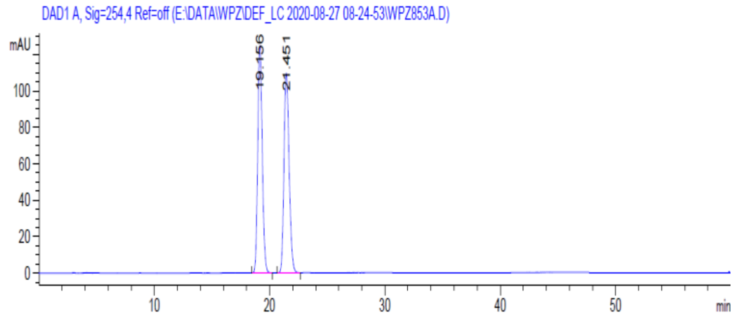

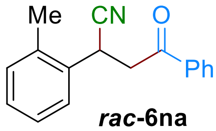

***rac*-6na**

Peak #	RetTime [min]	Type	Width [min]	Area mAU *s	Height [mAU]	Area %
1	19.156	BB	0.4246	3407.54053	124.76900	50.0613
2	21.451	BB	0.4820	3399.19043	109.54051	49.9387

Chiral HPLC spectrum of 6na

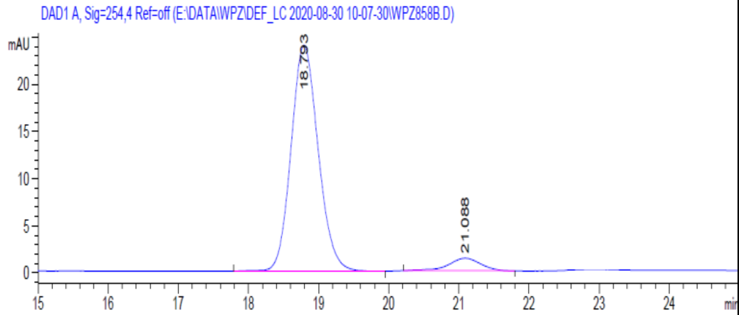

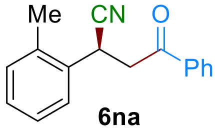

6na

Peak #	RetTime [min]	Type	Width [min]	Area mAU *s	Height [mAU]	Area %
1	18.793	BB	0.4153	643.68085	23.96768	93.8364
2	21.088	BB	0.4229	42.27982	1.32284	6.1636

Chiral HPLC spectrum of racemic 6oa

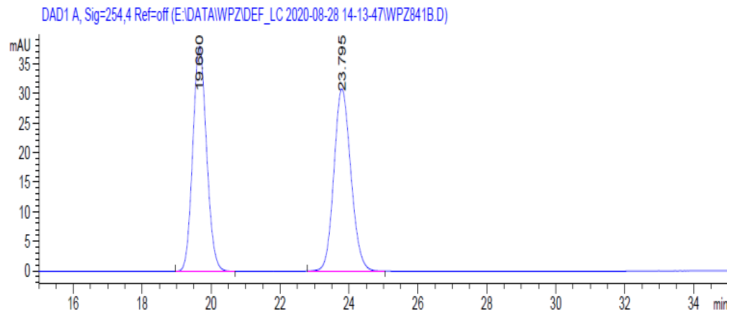

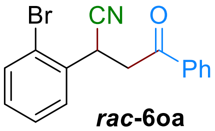

***rac*-6oa**

Peak #	RetTime [min]	Type	Width [min]	Area mAU *s	Height [mAU]	Area %
1	19.660	BB	0.4297	1054.87537	38.02231	50.0220
2	23.795	BB	0.5334	1053.94666	30.78683	49.9780

Chiral HPLC spectrum of 6oa

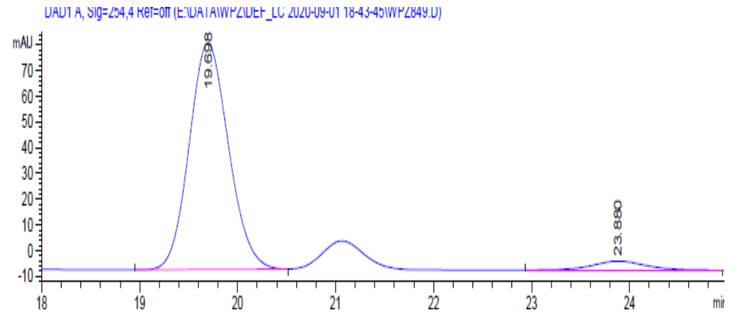

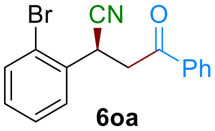

6oa

Peak #	RetTime [min]	Type	Width [min]	Area mAU *s	Height [mAU]	Area %
1	19.698	BB	0.4331	2454.32349	88.07878	94.9377
2	23.880	BB	0.5214	130.86972	3.45935	5.0623

Chiral HPLC spectrum of racemic 6pa

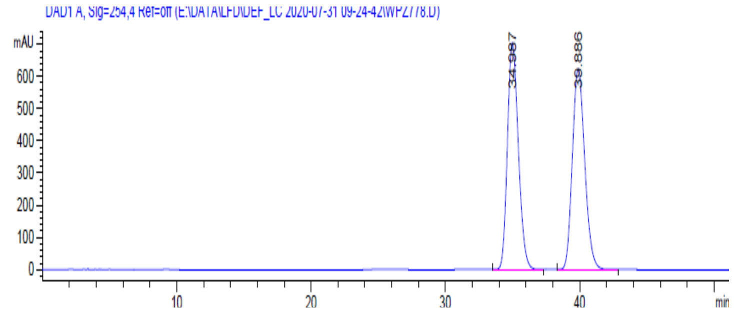

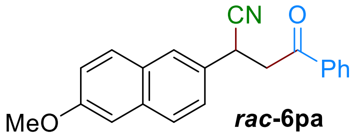

***rac*-6pa**

Peak #	RetTime [min]	Type	Width [min]	Area mAU *s	Height [mAU]	Area %
1	34.987	BB	0.8766	3.97663e4	704.41791	49.9275
2	39.886	BB	0.9974	3.98817e4	622.58167	50.0725

Chiral HPLC spectrum of 6pa

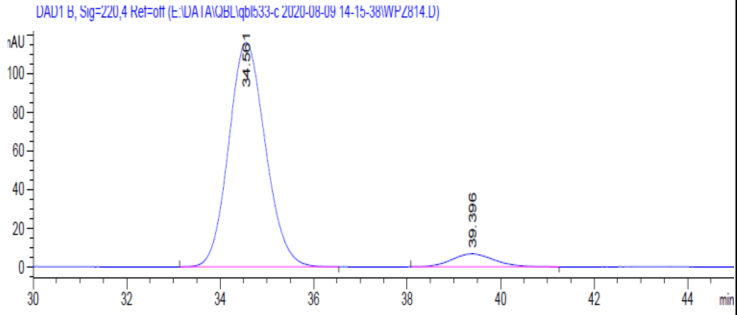

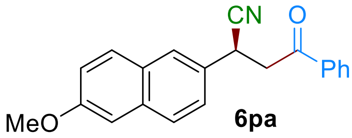

6pa

Peak #	RetTime [min]	Type	Width [min]	Area mAU *s	Height [mAU]	Area %
1	34.561	BB	0.8407	6305.37646	115.90758	93.7865
2	39.396	BB	0.7620	417.73740	6.62172	6.2135

Chiral HPLC spectrum of racemic 6qa

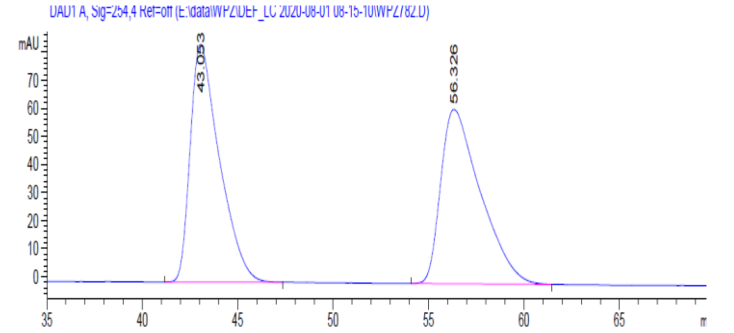

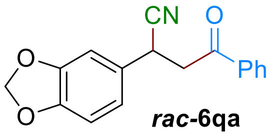

***rac*-6qa**

Peak #	RetTime [min]	Type	Width [min]	Area mAU *s	Height [mAU]	Area %
1	43.053	BB	1.5551	8994.69434	84.55399	50.0039
2	56.326	BB	1.9827	8993.29883	62.05772	49.9961

Chiral HPLC spectrum of 6qa

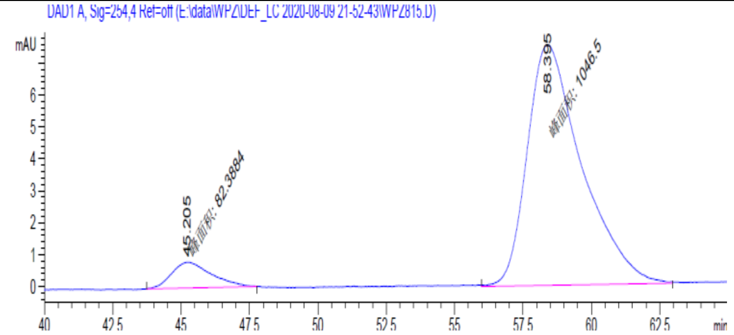

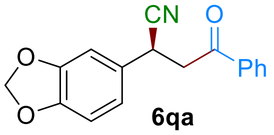

6qa

Peak #	RetTime [min]	Type	Width [min]	Area mAU *s	Height [mAU]	Area %
1	45.205	MM	1.6888	82.38841	8.13105e-1	7.2982
2	58.395	MM	2.3142	1046.50452	7.53673	92.7018

Chiral HPLC spectrum of racemic 6ra

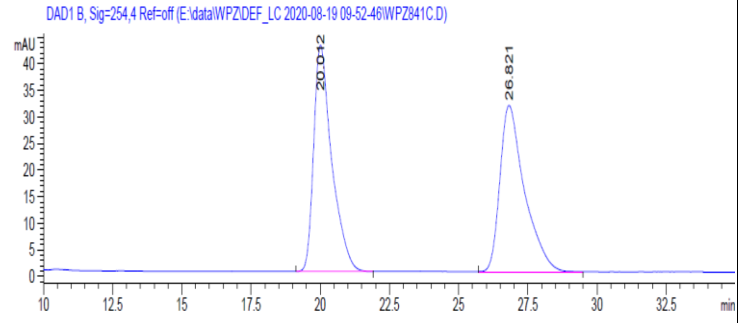

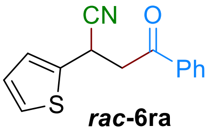

***rac*-6ra**

Peak #	RetTime [min]	Type	Width [min]	Area mAU *s	Height [mAU]	Area %
1	20.012	BB	0.6618	1922.83557	42.50033	49.9282
2	26.821	BB	0.8816	1928.36670	31.26552	50.0718

Chiral HPLC spectrum of 6ra

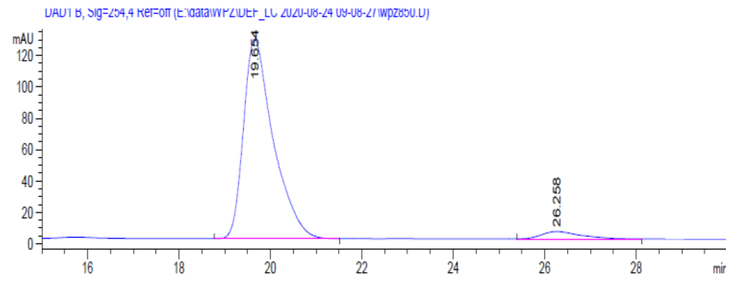

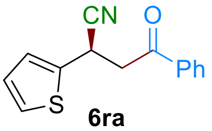

6ra

Peak #	RetTime [min]	Type	Width [min]	Area mAU *s	Height [mAU]	Area %
1	19.654	BB	0.6653	5748.60742	127.17993	95.2910
2	26.258	BB	0.7263	284.08176	4.82922	4.7090

Chiral HPLC spectrum of racemic 6sa

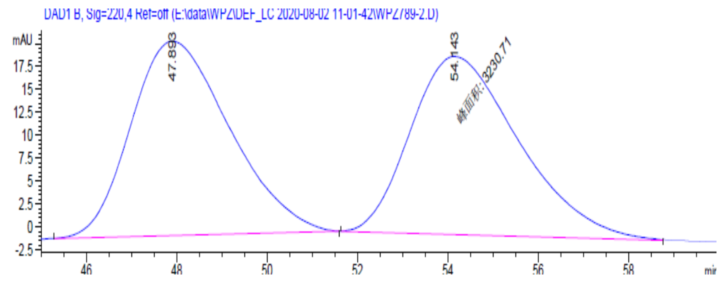

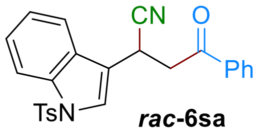

***rac*-6sa**

Peak #	RetTime [min]	Type	Width [min]	Area mAU *s	Height [mAU]	Area %
1	47.893	BB	1.7839	3211.95337	21.19602	49.8544
2	54.143	MM	2.7733	3230.71460	19.41527	50.1456

Chiral HPLC spectrum of 6sa

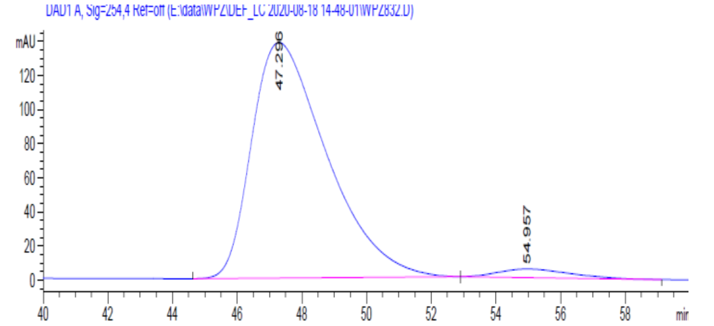

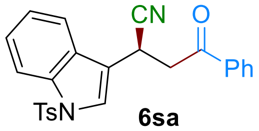

6sa

Peak #	RetTime [min]	Type	Width [min]	Area mAU *s	Height [mAU]	Area %
1	47.296	BB	2.4347	2.22691e4	137.99545	96.6207
2	54.957	BB	1.9206	778.85175	5.02409	3.3793

Chiral HPLC spectrum of racemic 6ta

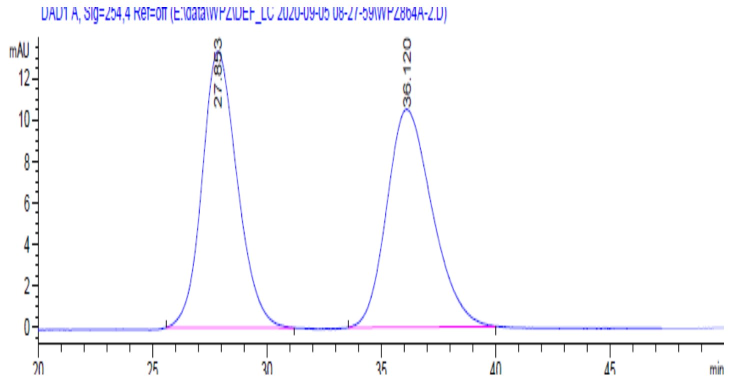

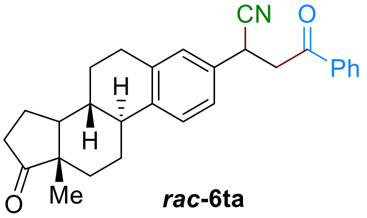

***rac*-6ta**

Peak #	RetTime [min]	Type	Width [min]	Area mAU *s	Height [mAU]	Area %
1	27.853	BB	1.2738	1428.58423	13.33506	49.5366
2	36.120	BB	1.6247	1455.31396	10.51474	50.4634

Chiral HPLC spectrum of 6ta

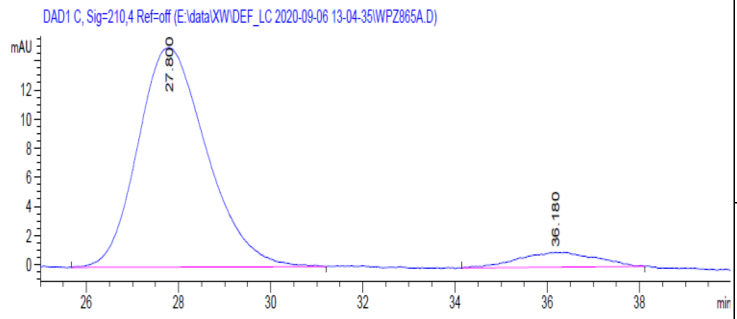

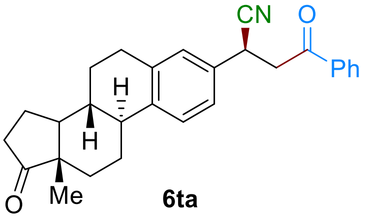

6ta

Peak #	RetTime [min]	Type	Width [min]	Area mAU *s	Height [mAU]	Area %
1	27.800	BB	1.3182	1565.38306	15.09093	92.9140
2	36.180	BB	1.3837	119.38286	1.01541	7.0860

Chiral HPLC spectrum of racemic 6ua

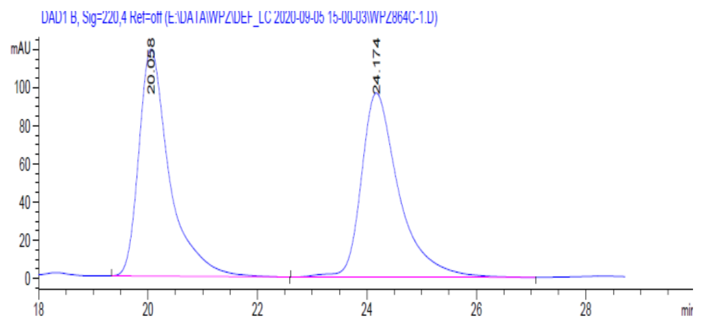

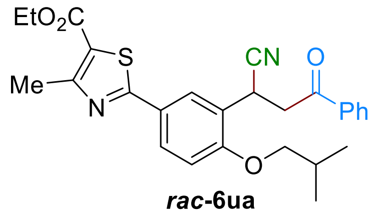

rac-6ua

Peak #	RetTime [min]	Type	Width [min]	Area mAU *s	Height [mAU]	Area %
1	20.058	BB	0.5755	4624.28174	118.96624	50.1075
2	24.174	BB	0.7065	4604.43555	96.52800	49.8925

Chiral HPLC spectrum of 6ua

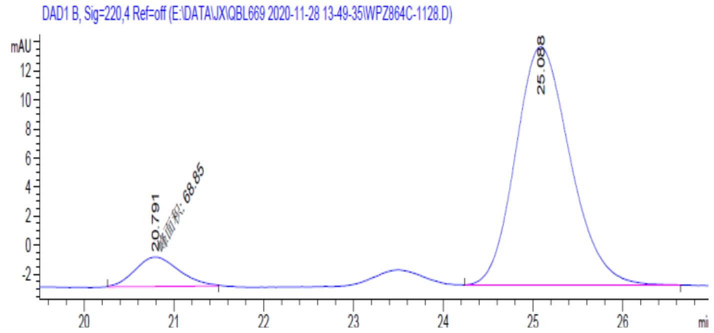

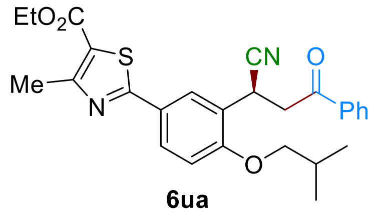

6ua

Peak #	RetTime [min]	Type	Width [min]	Area mAU *s	Height [mAU]	Area %
1	20.791	MM	0.5677	68.85000	2.02138	9.0588
2	25.088	BB	0.6428	691.18433	16.36437	90.9412

Chiral HPLC spectrum of racemic 6va

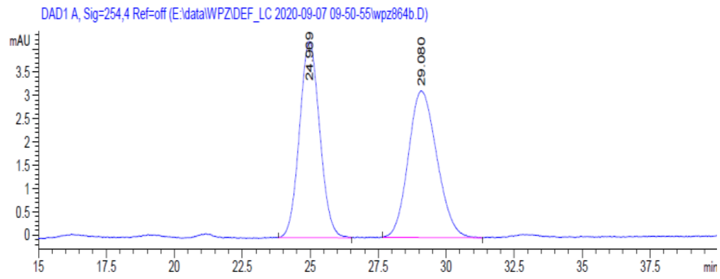

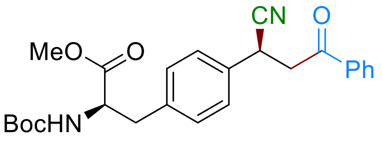

rac-6va

Peak #	RetTime [min]	Type	Width [min]	Area mAU *s	Height [mAU]	Area %
1	24.969	BB	0.6126	481.90442	9.45481	49.9730
2	29.081	BB	0.8345	482.42493	6.92709	50.0270

Chiral HPLC spectrum of 6va

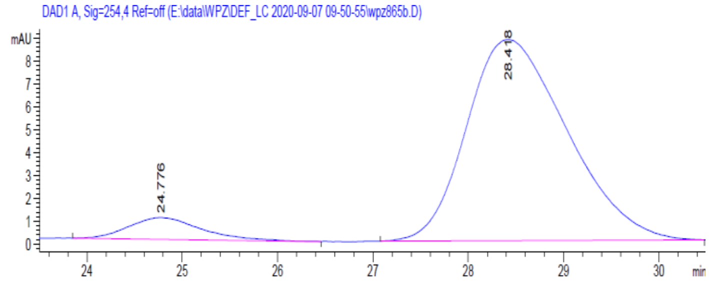

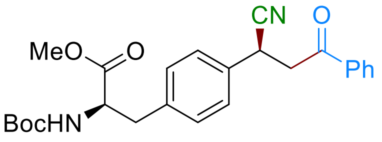

6va

Peak #	RetTime [min]	Type	Width [min]	Area mAU *s	Height [mAU]	Area %
1	24.776	BB	0.6557	50.44384	9.46587e-1	7.2698
2	28.418	BB	1.0603	643.43494	8.78749	92.7302

Chiral HPLC spectrum of racemic 6bb

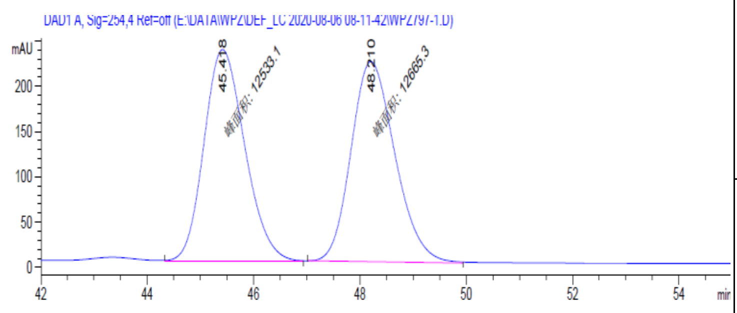

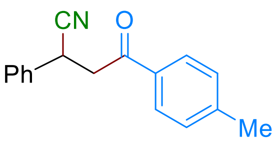

***rac*-6bb**

Peak #	RetTime [min]	Type	Width [min]	Area mAU *s	Height [mAU]	Area %
1	45.418	MM	0.8923	1.25331e4	234.09033	49.7377
2	48.210	MM	0.9551	1.26653e4	221.00250	50.2623

Chiral HPLC spectrum of 6bb

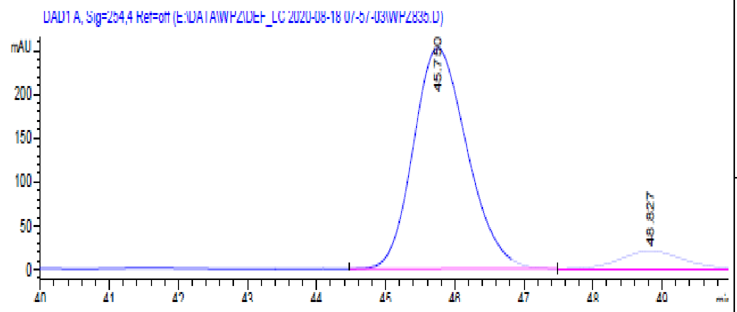

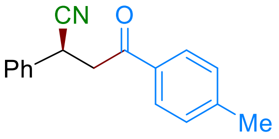

6bb

Peak #	RetTime [min]	Type	Width [min]	Area mAU *s	Height [mAU]	Area %
1	45.750	BB	0.8361	1.36163e4	252.11067	91.5543
2	48.827	BB	0.9078	1256.07886	20.24701	8.4457

Chiral HPLC spectrum of racemic 6bc

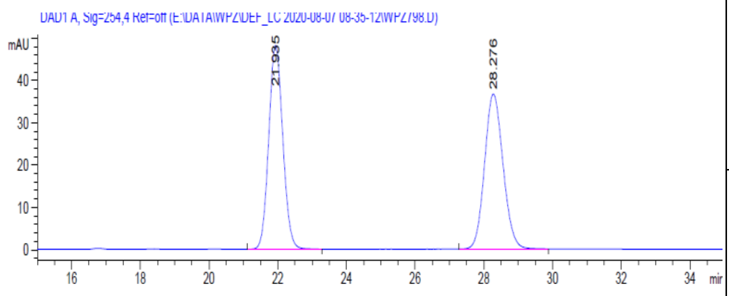

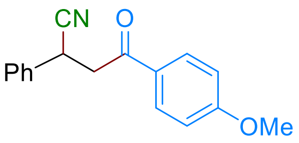

***rac*-6bc**

Peak #	RetTime [min]	Type	Width [min]	Area mAU *s	Height [mAU]	Area %
1	21.935	BB	0.4394	1367.86023	48.14893	50.0042
2	28.276	BB	0.5777	1367.63220	36.61932	49.9958

Chiral HPLC spectrum of 6bc

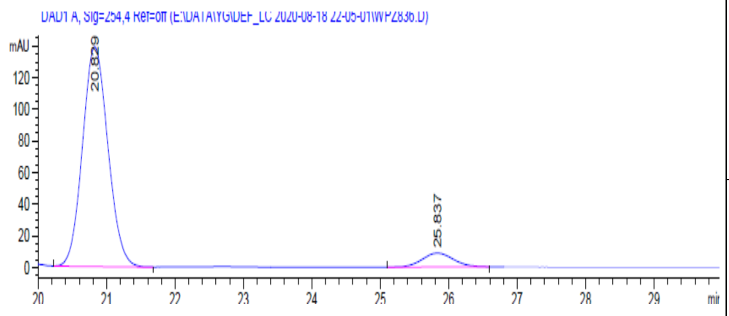

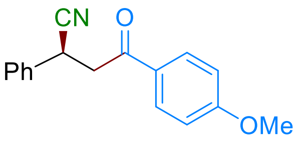

6bc

Peak #	RetTime [min]	Type	Width [min]	Area mAU *s	Height [mAU]	Area %
1	20.829	BB	0.4029	3583.14917	138.94525	92.9221
2	25.837	BB	0.4722	272.92938	8.74192	7.0779

Chiral HPLC spectrum of racemic 6bd

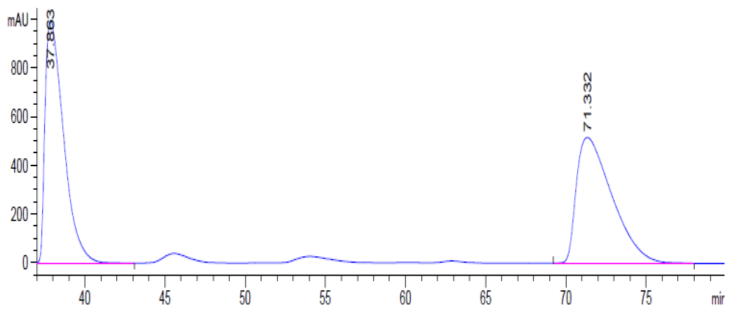

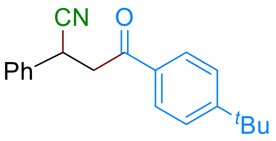

***rac*-6bd**

Peak #	RetTime [min]	Type	Width [min]	Area mAU *s	Height [mAU]	Area %
1	37.863	VB	1.2633	8.02013e4	996.12976	50.1394
2	71.332	BB	2.2113	7.97555e4	515.98645	49.8606

Chiral HPLC spectrum of 6bd

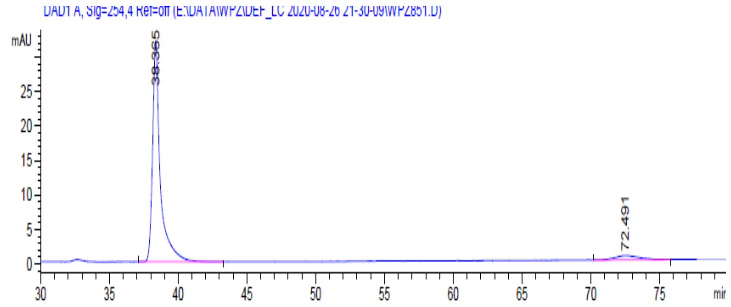

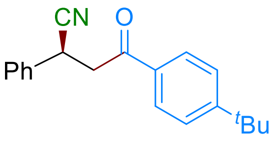

6bd

Peak #	RetTime [min]	Type	Width [min]	Area mAU *s	Height [mAU]	Area %
1	38.365	BB	0.5542	1252.53955	31.89255	94.2642
2	72.491	BB	1.7110	76.21436	6.11105e-1	5.7358

Chiral HPLC spectrum of racemic 6be

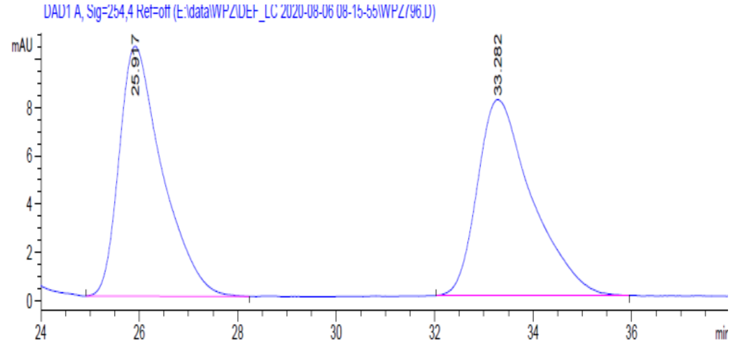

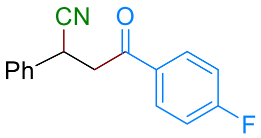

***rac*-6be**

Peak #	RetTime [min]	Type	Width [min]	Area mAU *s	Height [mAU]	Area %
1	25.917	BB	0.8556	630.00488	10.35826	50.1229
2	33.282	BB	0.9896	626.91638	8.13066	49.8771

Chiral HPLC spectrum of 6be

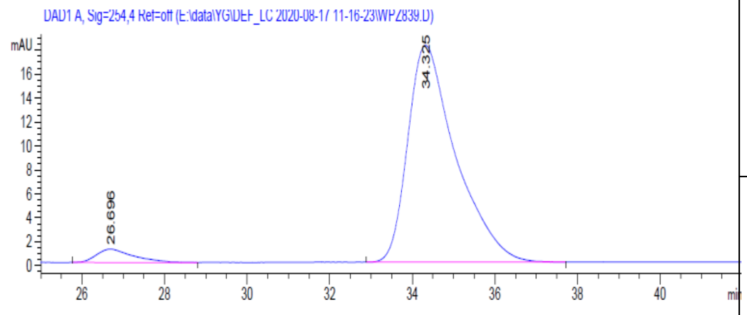

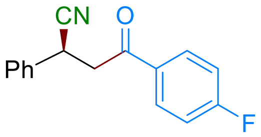

6be

Peak #	RetTime [min]	Type	Width [min]	Area mAU *s	Height [mAU]	Area %
1	26.696	BB	0.7875	70.29843	1.12113	4.5864
2	34.325	BB	1.1740	1462.46875	18.11232	95.4136

Chiral HPLC spectrum of racemic 6bf

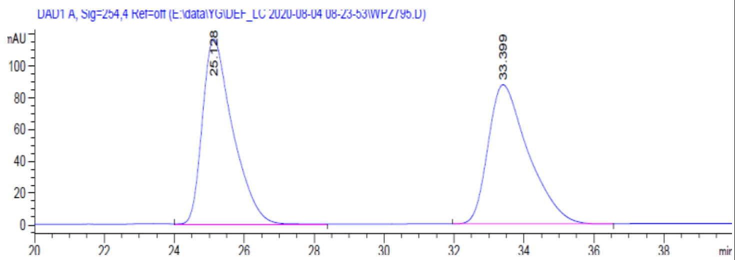

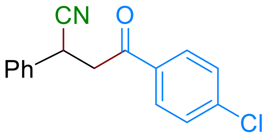

***rac*-6bf**

Peak #	RetTime [min]	Type	Width [min]	Area mAU *s	Height [mAU]	Area %
1	25.128	BB	0.8880	6992.50146	116.20441	50.0880
2	33.399	BB	1.1684	6967.93701	87.37325	49.9120

Chiral HPLC spectrum of 6bf

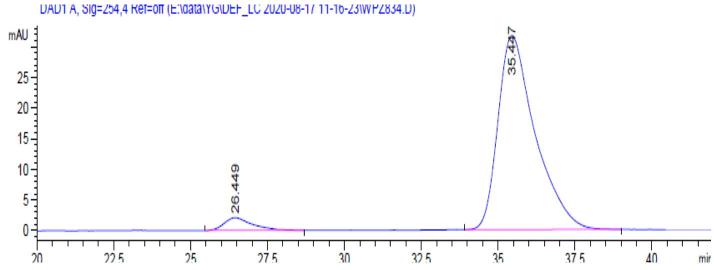

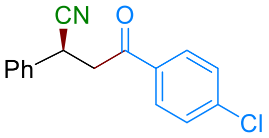

6bf

Peak #	RetTime [min]	Type	Width [min]	Area mAU *s	Height [mAU]	Area %
1	26.449	BB	0.7992	129.06351	2.05922	4.6288
2	35.447	BB	1.2363	2659.21094	31.64689	95.3712

Chiral HPLC spectrum of racemic 6bg

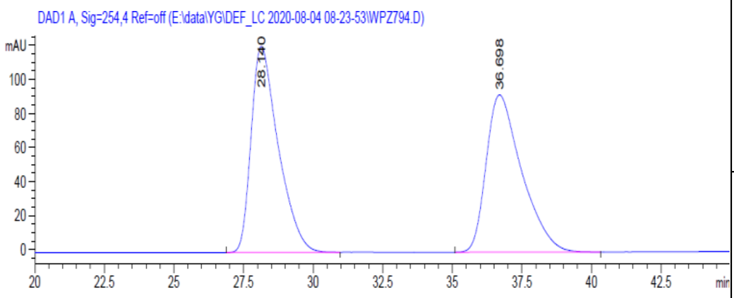

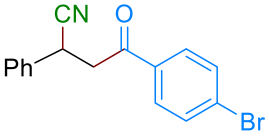

***rac*-6bg**

Peak #	RetTime [min]	Type	Width [min]	Area mAU *s	Height [mAU]	Area %
1	28.140	BB	1.0110	8199.75684	120.94640	50.0097
2	36.698	BB	1.3053	8196.56738	92.36240	49.9903

Chiral HPLC spectrum of 6bg

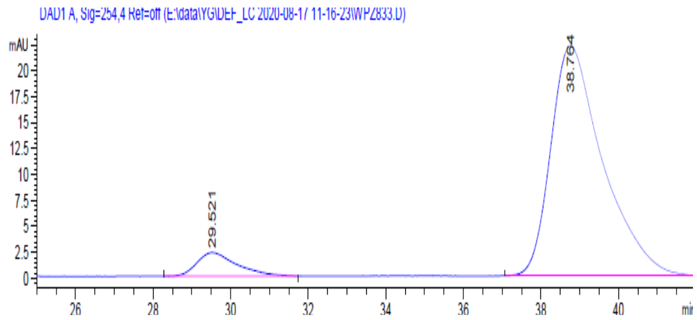

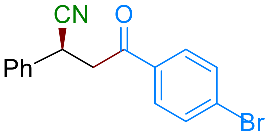

6bg

Peak #	RetTime [min]	Type	Width [min]	Area mAU *s	Height [mAU]	Area %
1	29.521	BB	0.8550	161.99908	2.26341	7.2964
2	38.764	BB	1.3497	2058.25244	22.11783	92.7036

Chiral HPLC spectrum of racemic 6bh

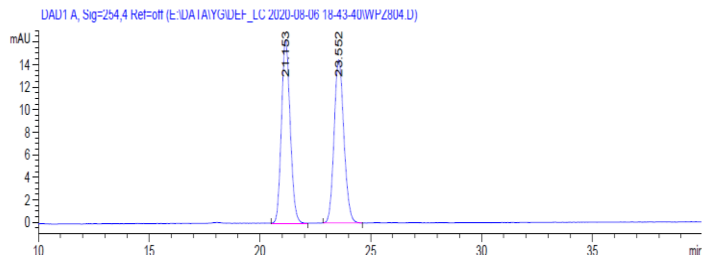

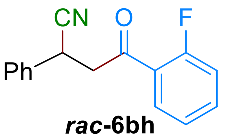

***rac*-6bh**

Peak #	RetTime [min]	Type	Width [min]	Area mAU *s	Height [mAU]	Area %
1	21.153	BB	0.4208	444.58609	16.26992	50.0109
2	23.552	BB	0.4765	444.39249	14.46220	49.9891

Chiral HPLC spectrum of 6bh

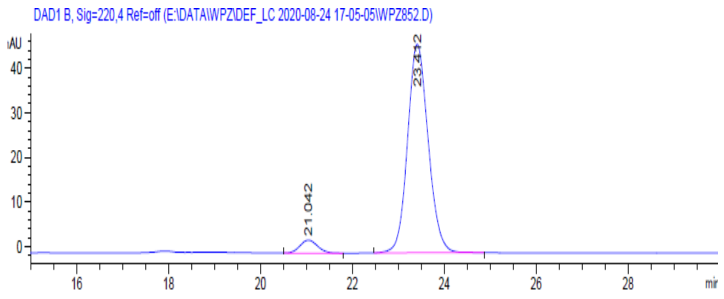

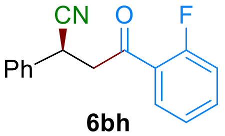

6bh

Peak #	RetTime [min]	Type	Width [min]	Area mAU *s	Height [mAU]	Area %
1	21.042	BB	0.3871	78.52249	2.92634	5.1136
2	23.412	BB	0.4821	1457.03589	46.68394	94.8864

Chiral HPLC spectrum of racemic 6bi

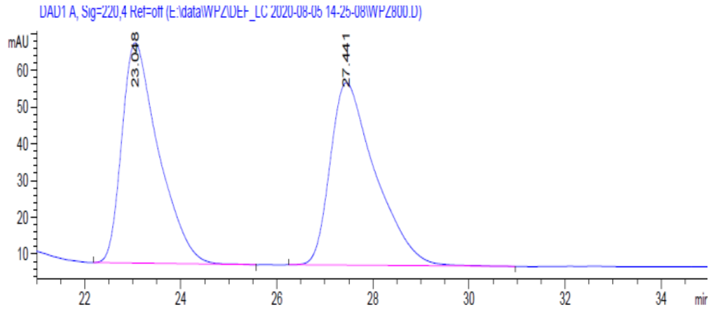

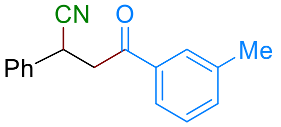

***rac*-6bi**

Peak #	RetTime [min]	Type	Width [min]	Area mAU *s	Height [mAU]	Area %
1	23.048	BB	0.7767	3176.02441	59.89371	49.5240
2	27.441	BB	0.9427	3237.08203	49.64606	50.4760

Chiral HPLC spectrum of 6bi

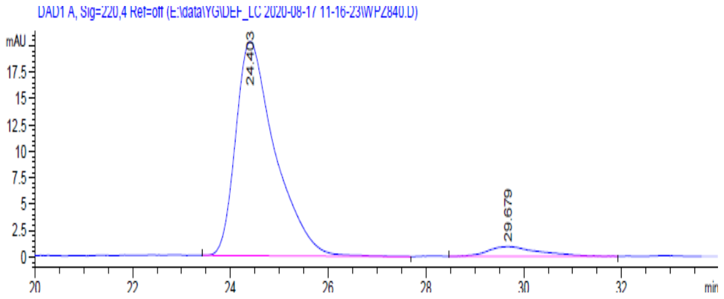

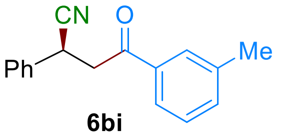

6bi

Peak #	RetTime [min]	Type	Width [min]	Area mAU *s	Height [mAU]	Area %
1	24.403	BB	0.8234	1131.67456	20.26764	94.4204
2	29.679	BB	0.8582	66.87450	9.30812e-1	5.5796

Chiral HPLC spectrum of racemic 6bj

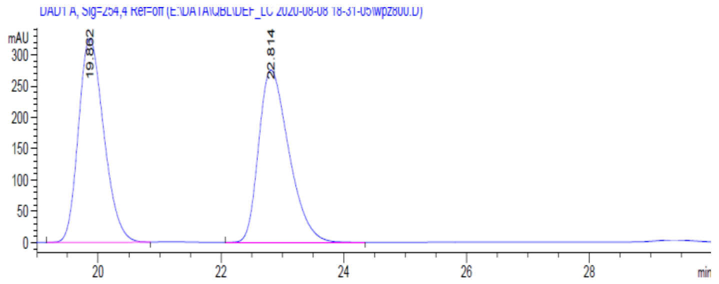

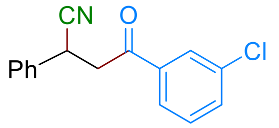

rac-**6bj**

Peak #	RetTime [min]	Type	Width [min]	Area mAU *s	Height [mAU]	Area %
1	19.862	BB	0.4470	9348.61816	325.60077	49.8397
2	22.814	BB	0.5267	9408.73828	275.31244	50.1603

Chiral HPLC spectrum of 6bj

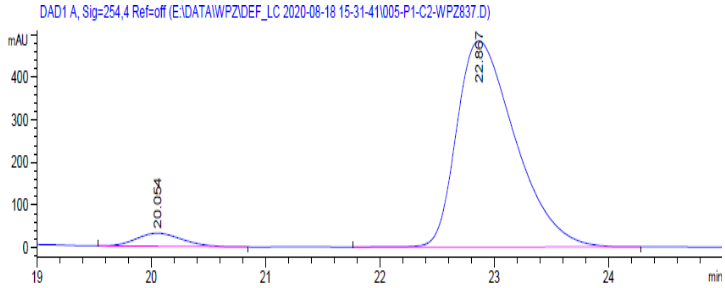

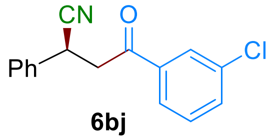

6bj

Peak #	RetTime [min]	Type	Width [min]	Area mAU *s	Height [mAU]	Area %
1	20.054	BB	0.4359	856.19867	30.65477	4.7437
2	22.867	BB	0.5461	1.71928e4	484.38821	95.2563

Chiral HPLC spectrum of racemic 6bk

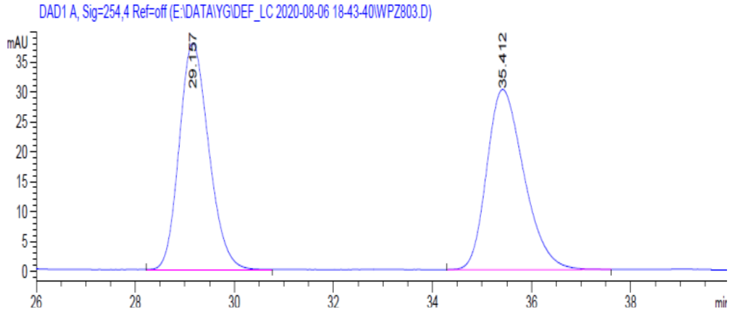

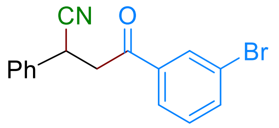

rac-**6bk**

Peak #	RetTime [min]	Type	Width [min]	Area mAU *s	Height [mAU]	Area %
1	29.157	BB	0.6353	1567.01404	37.82694	49.9372
2	35.412	BB	0.7888	1570.95300	30.10307	50.0628

Chiral HPLC spectrum of 6bk

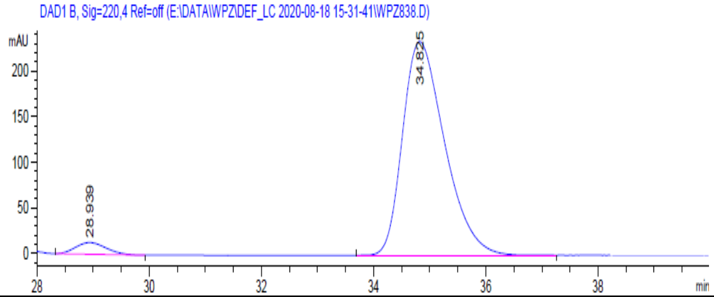

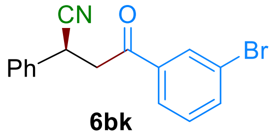

6bk

Peak #	RetTime [min]	Type	Width [min]	Area mAU *s	Height [mAU]	Area %
1	28.939	BB	0.5739	488.93558	12.85030	3.8531
2	34.825	BB	0.7995	1.22003e4	234.31572	96.1469

Chiral HPLC spectrum of racemic 6bl

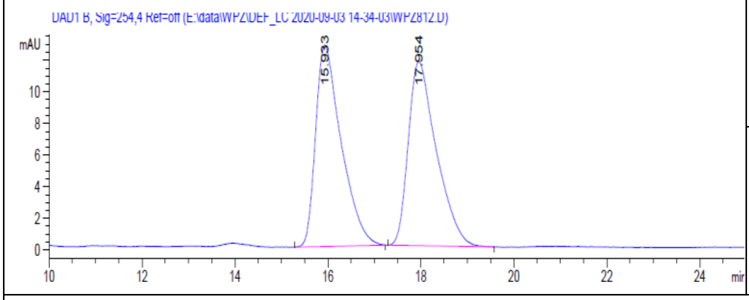

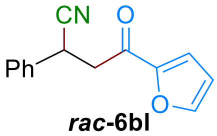

***rac*-6bl**

Peak #	RetTime [min]	Type	Width [min]	Area mAU *s	Height [mAU]	Area %
1	15.933	BB	0.5567	487.38211	12.73058	49.9013
2	17.954	BB	0.6042	489.31027	11.64459	50.0987

Chiral HPLC spectrum of 6bl

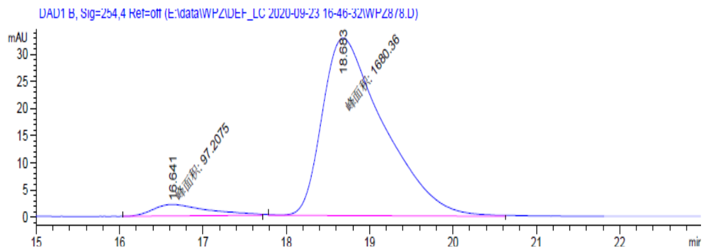

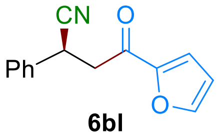

6bl

Peak #	RetTime [min]	Type	Width [min]	Area mAU *s	Height [mAU]	Area %
1	16.641	MM	0.7628	97.20747	2.12378	5.4686
2	18.683	MM	0.8561	1680.35864	32.71356	94.5314

Chiral HPLC spectrum of racemic 6am

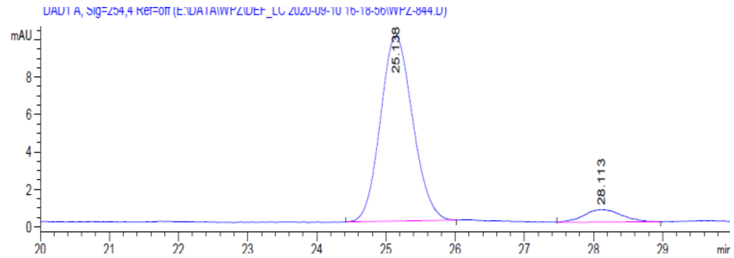

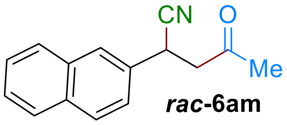

***rac*-6am**

Peak #	RetTime [min]	Type	Width [min]	Area mAU *s	Height [mAU]	Area %
1	25.324	BB	0.5007	690.34271	21.15656	49.9909
2	28.225	BB	0.5524	690.59387	18.54740	50.0091

Chiral HPLC spectrum of 6am

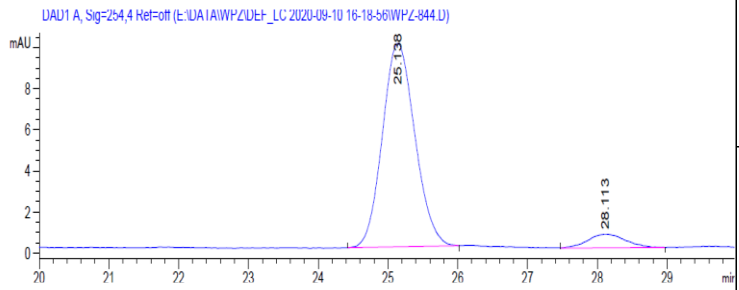

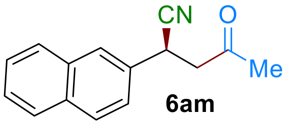

6am

Peak #	RetTime [min]	Type	Width [min]	Area mAU *s	Height [mAU]	Area %
1	25.138	BB	0.4898	320.46442	9.89632	92.7039
2	28.113	BB	0.4473	25.22163	6.75990e-1	7.2961

Chiral HPLC spectrum of racemic 6an

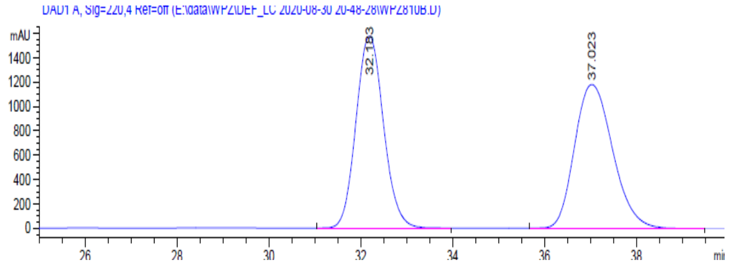

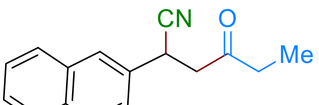

rac-**6an**

Peak #	RetTime [min]	Type	Width [min]	Area mAU *s	Height [mAU]	Area %
1	32.183	BB	0.6492	6.54987e4	1574.90320	49.1585
2	37.023	BB	0.9064	6.77411e4	1178.94141	50.8415

Chiral HPLC spectrum of 6an

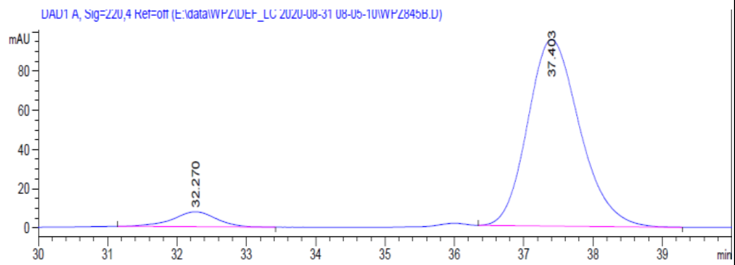

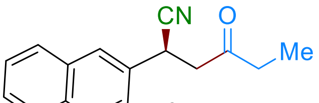

6an

Peak #	RetTime [min]	Type	Width [min]	Area mAU *s	Height [mAU]	Area %
1	32.270	BB	0.6387	337.51010	7.65032	6.3759
2	37.403	BB	0.8016	4956.05273	95.16989	93.6241

Chiral HPLC spectrum of racemic 6ao

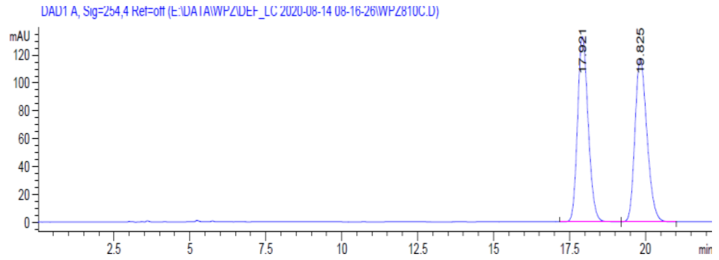

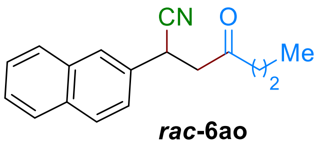

rac-**6ao**

Peak #	RetTime [min]	Type	Width [min]	Area mAU *s	Height [mAU]	Area %
1	17.921	BB	0.3769	3225.17285	132.99915	50.0148
2	19.825	BB	0.4263	3223.26245	117.40053	49.9852

Chiral HPLC spectrum of 6ao

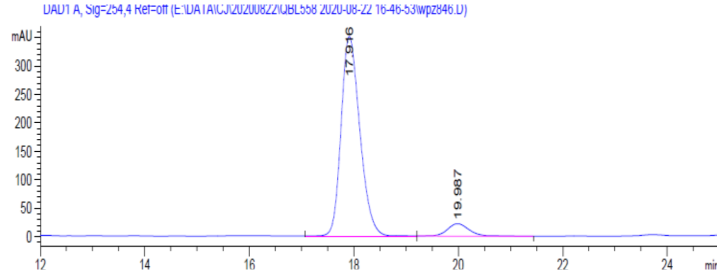

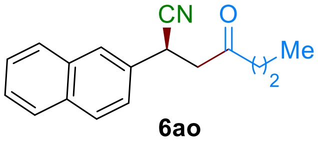

6ao

Peak #	RetTime [min]	Type	Width [min]	Area mAU *s	Height [mAU]	Area %
1	17.916	BB	0.3847	8753.75879	351.34348	93.1458
2	19.987	BB	0.4490	644.15625	22.04073	6.8542

Chiral HPLC spectrum of racemic 6ap

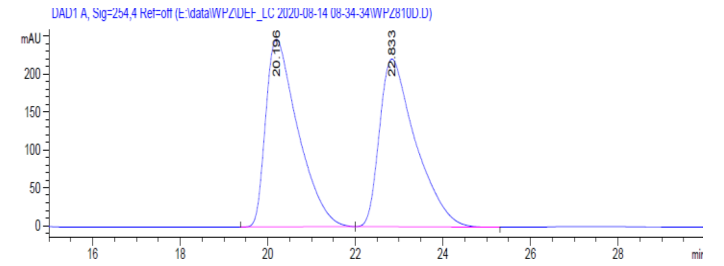

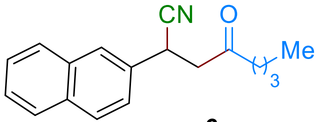

***rac*-6ap**

Peak #	RetTime [min]	Type	Width [min]	Area mAU *s	Height [mAU]	Area %
1	20.196	BB	0.7592	1.26158e4	247.27063	49.9361
2	22.833	BB	0.8394	1.26481e4	221.09612	50.0639

Chiral HPLC spectrum of 6ap

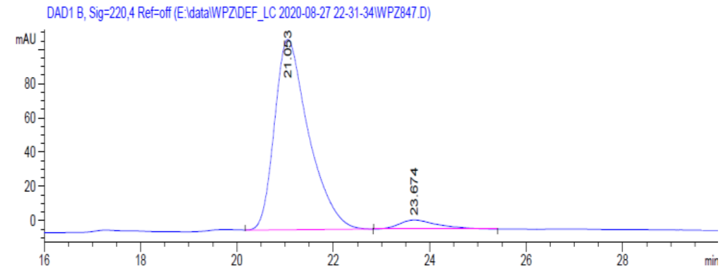

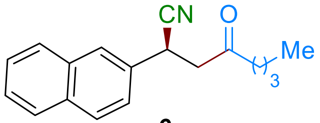

6ap

Peak #	RetTime [min]	Type	Width [min]	Area mAU *s	Height [mAU]	Area %
1	21.053	BB	0.7282	5450.28271	111.13112	94.9564
2	23.674	BB	0.7069	289.49121	5.26983	5.0436

Chiral HPLC spectrum of racemic 7aa

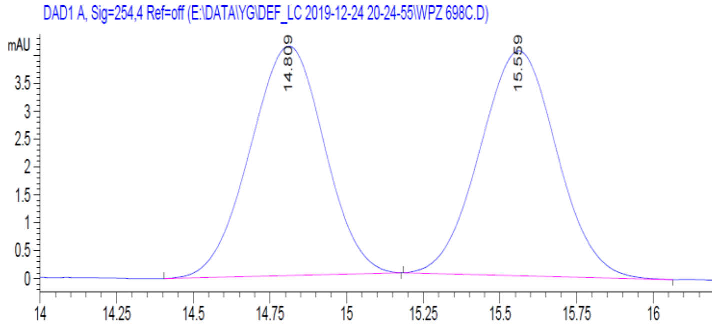

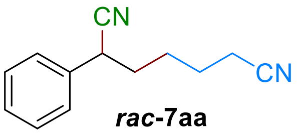

***rac*-7aa**

Peak #	RetTime [min]	Type	Width [min]	Area mAU *s	Height [mAU]	Area %
1	14.809	BV	0.2691	4088.08887	237.18434	49.8731
2	15.560	VB	0.2750	4108.89160	231.59380	50.1269

Chiral HPLC spectrum of 7aa

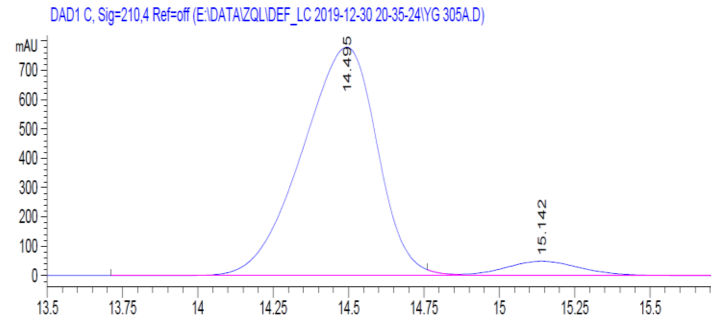

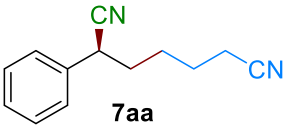

7aa

Peak #	RetTime [min]	Type	Width [min]	Area mAU *s	Height [mAU]	Area %
1	14.495	BV R	0.2703	1.35065e4	778.42566	94.0782
2	15.142	VV E	0.2718	850.18256	48.65966	5.9218

Chiral HPLC spectrum of racemic 7ba

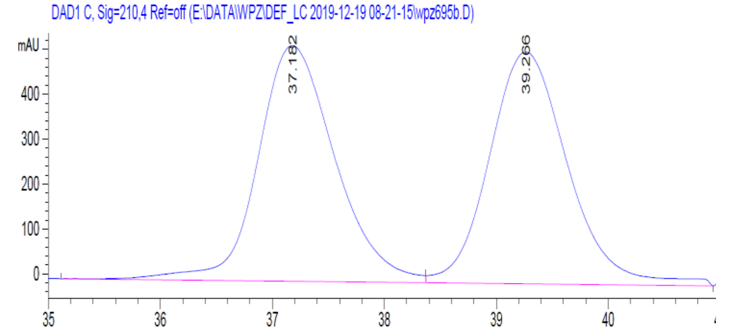

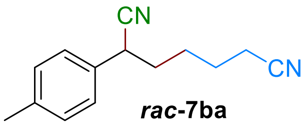

rac-7ba

Peak #	RetTime [min]	Type	Width [min]	Area mAU *s	Height [mAU]	Area %
1	37.182	BV	0.7283	2.51487e4	523.65747	50.0170
2	39.266	VB	0.7525	2.51316e4	515.71075	49.9830

Chiral HPLC spectrum of 7ba

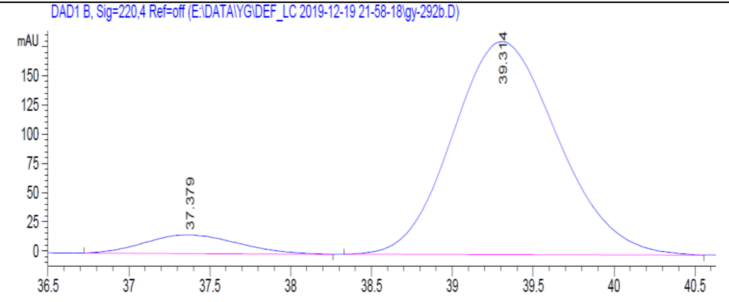

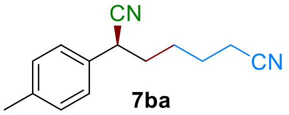

7ba

Peak #	RetTime [min]	Type	Width [min]	Area mAU *s	Height [mAU]	Area %
1	37.379	BB	0.4891	647.44360	16.10316	7.2545
2	39.314	BB	0.6953	8277.28027	182.43152	92.7455

Chiral HPLC spectrum of racemic 7ca

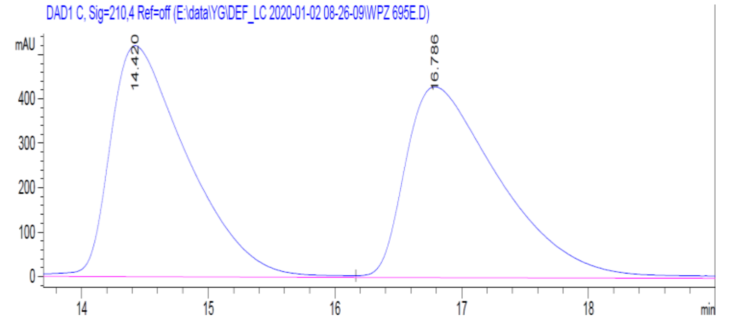

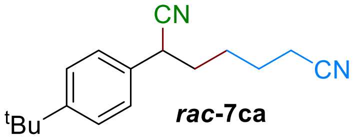

rac-7ca

Peak #	RetTime [min]	Type	Width [min]	Area mAU *s	Height [mAU]	Area %
1	14.420	VV R	0.6167	2.29561e4	520.74652	50.5506
2	16.786	VB	0.7893	2.24560e4	430.00790	49.4494

Chiral HPLC spectrum of 7ca

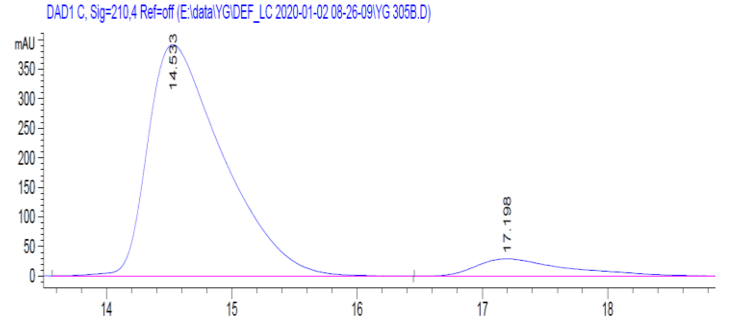

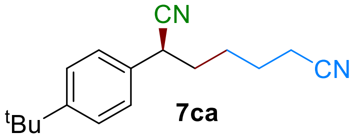

7ca

Peak #	RetTime [min]	Type	Width [min]	Area mAU *s	Height [mAU]	Area %
1	14.533	BB	0.6144	1.63003e4	391.00357	91.5522
2	17.198	BB	0.6904	1504.08850	29.32280	8.4478

Chiral HPLC spectrum of racemic 7da

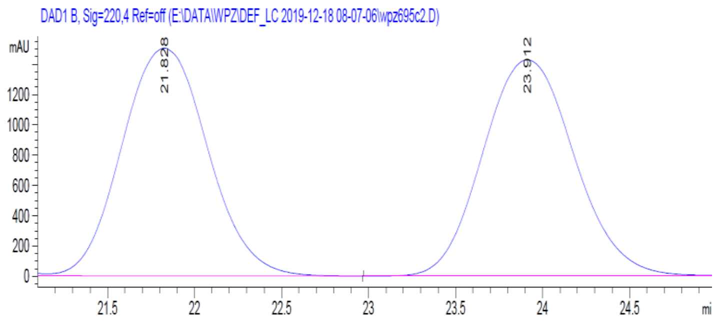

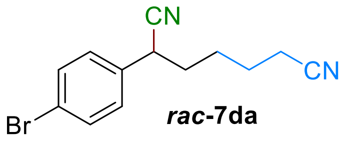

rac-**7da**

Peak #	RetTime [min]	Type	Width [min]	Area mAU *s	Height [mAU]	Area %
1	21.828	VB R	0.5416	5.40845e4	1503.25867	50.8230
2	23.912	BB	0.5755	5.23327e4	1428.33740	49.1770

Chiral HPLC spectrum of 7da

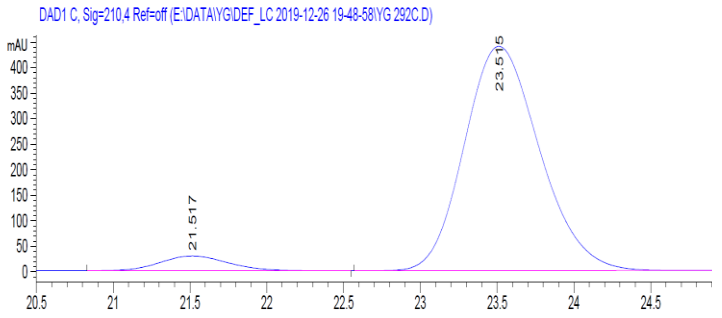

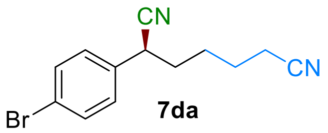

7da

Peak #	RetTime [min]	Type	Width [min]	Area mAU *s	Height [mAU]	Area %
1	21.517	BB	0.4766	889.74597	28.94557	5.6021
2	23.515	BBA	0.5303	1.49925e4	439.20050	94.3979

Chiral HPLC spectrum of racemic 7ea

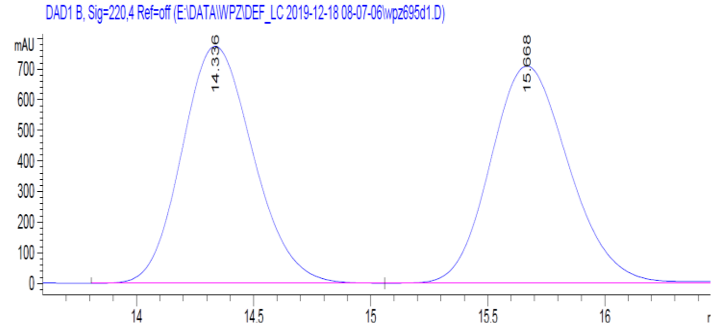

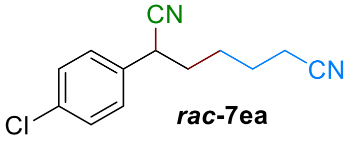

rac-**7ea**

Peak #	RetTime [min]	Type	Width [min]	Area mAU *s	Height [mAU]	Area %
1	14.336	BB	0.3270	1.63739e4	773.45380	49.6218
2	15.668	BB	0.3657	1.66235e4	708.54803	50.3782

Chiral HPLC spectrum of 7ea

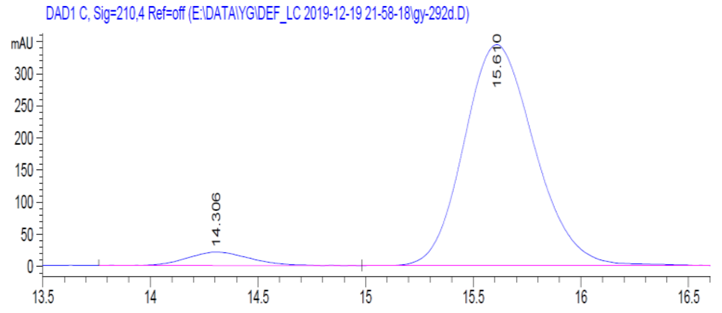

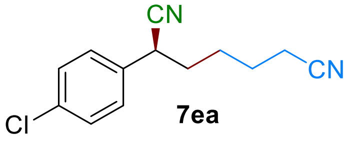

7ea

Peak #	RetTime [min]	Type	Width [min]	Area mAU *s	Height [mAU]	Area %
1	14.306	BB	0.3076	436.17953	21.23687	5.2587
2	15.610	BB	0.3543	7858.19336	344.26422	94.7413

Chiral HPLC spectrum of racemic 7fa

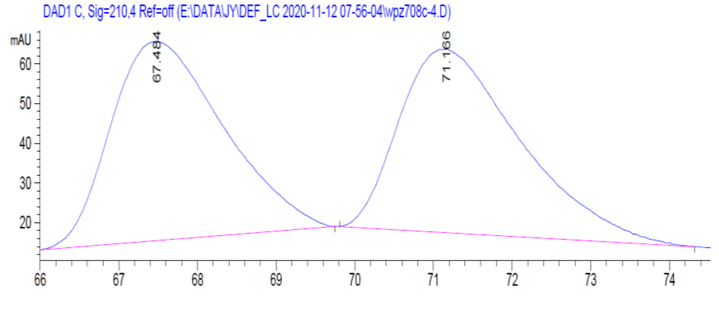

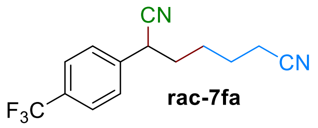

Peak #	RetTime [min]	Type	Width [min]	Area mAU *s	Height [mAU]	Area %
1	67.484	BB	1.1740	4995.87988	50.33239	50.1892
2	71.166	BB	1.2656	4958.21582	46.21582	49.8108

Chiral HPLC spectrum of 7fa

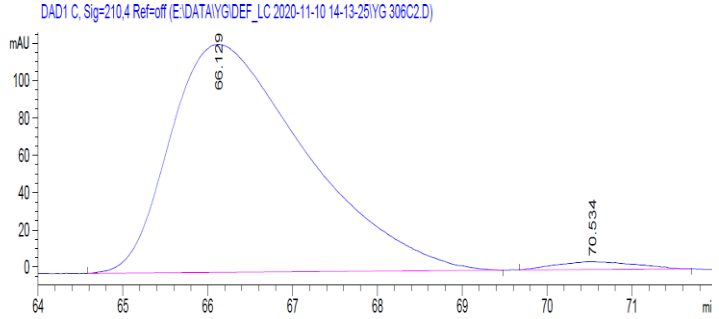

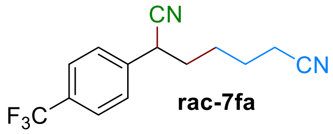

Peak #	RetTime [min]	Type	Width [min]	Area mAU *s	Height [mAU]	Area %
1	66.129	BB	1.3183	1.35730e4	122.34863	98.0793
2	70.534	BB	0.7531	265.80115	4.15101	1.9207

Chiral HPLC spectrum of racemic 7ga

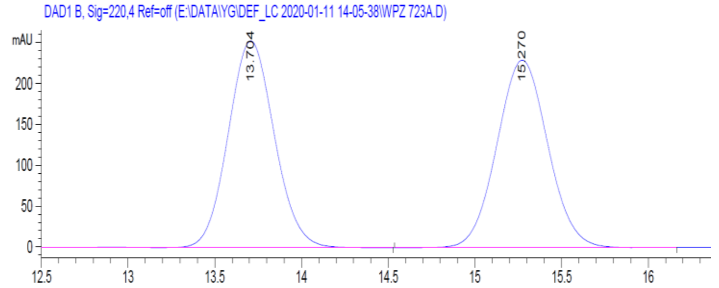

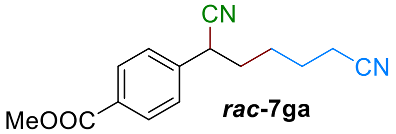

Peak #	RetTime [min]	Type	Width [min]	Area mAU *s	Height [mAU]	Area %
1	13.704	VB R	0.2874	4695.93945	252.89575	50.0786
2	15.270	BB	0.3182	4681.19385	229.32558	49.9214

Chiral HPLC spectrum of 7ga

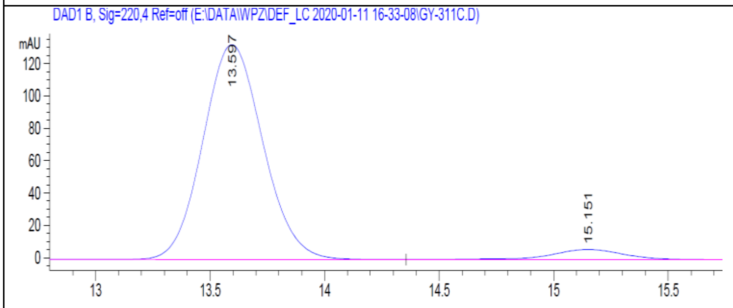

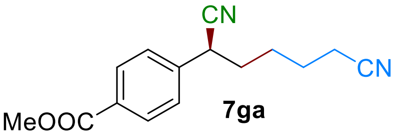

Peak #	RetTime [min]	Type	Width [min]	Area mAU *s	Height [mAU]	Area %
1	13.597	BV	0.2853	2429.32520	132.89589	94.4078
2	15.151	VB	0.3462	143.90105	6.30603	5.5922

Chiral HPLC spectrum of racemic 7ha

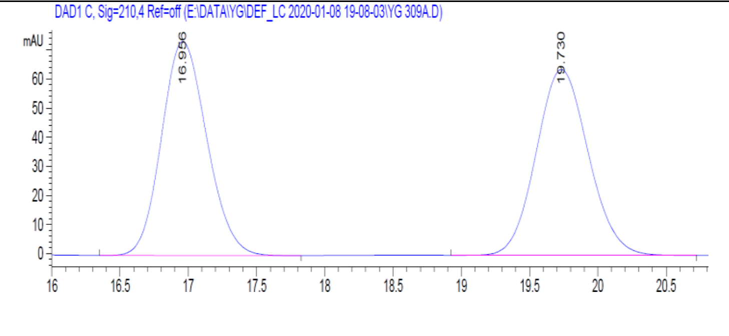

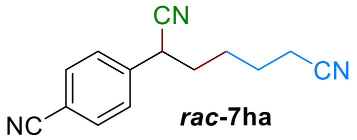

rac-7ha

Peak #	RetTime [min]	Type	Width [min]	Area mAU *s	Height [mAU]	Area %
1	16.956	BB	0.3595	1700.63672	73.61523	49.9973
2	19.730	BB	0.4146	1700.82043	63.89059	50.0027

Chiral HPLC spectrum of 7ha

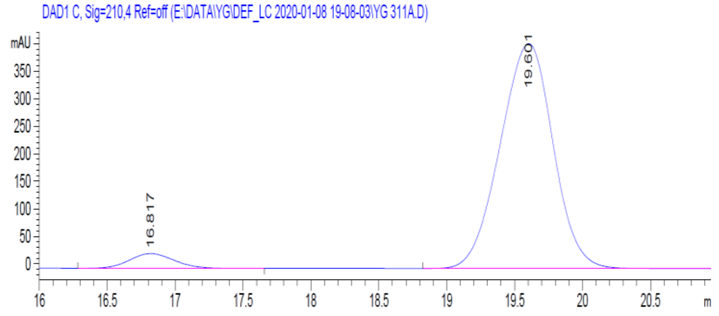

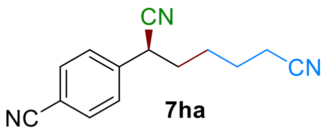

7ha

Peak #	RetTime [min]	Type	Width [min]	Area mAU *s	Height [mAU]	Area %
1	16.817	BB	0.3568	613.64069	26.83625	5.2551
2	19.601	BB	0.4259	1.10634e4	408.61282	94.7449

Chiral HPLC spectrum of racemic 7ia

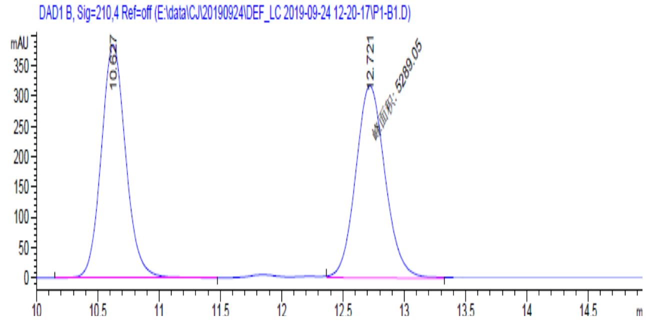

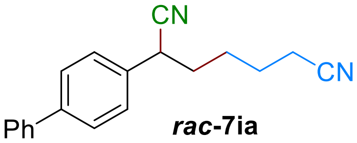

rac-7ia

Peak #	RetTime [min]	Type	Width [min]	Area mAU *s	Height [mAU]	Area %
1	10.627	BB	0.2133	5308.91748	384.04639	50.0937
2	12.721	MM	0.2784	5289.04736	316.68781	49.9063

Chiral HPLC spectrum of 7ia

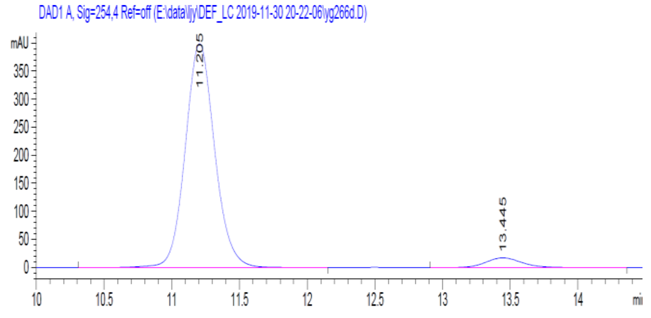

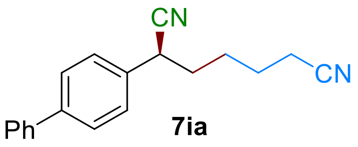

7ia

Peak #	RetTime [min]	Type	Width [min]	Area mAU *s	Height [mAU]	Area %
1	11.205	BB	0.2320	6021.04004	399.19312	95.0325
2	13.445	BB	0.2831	314.73245	17.23043	4.9675

Chiral HPLC spectrum of racemic 7ja

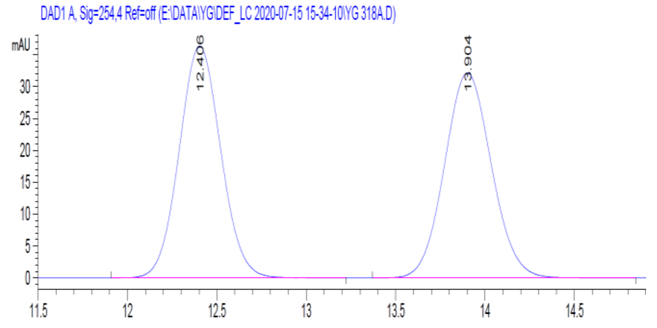

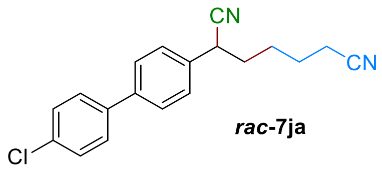

***rac*-7ja**

Peak #	RetTime [min]	Type	Width [min]	Area mAU *s	Height [mAU]	Area %
1	12.406	BB	0.2527	588.73004	36.39417	50.0861
2	13.904	BB	0.2829	586.70538	32.14546	49.9139

Chiral HPLC spectrum of 7ja

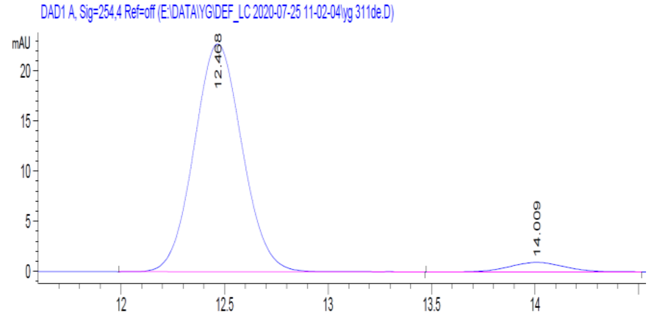

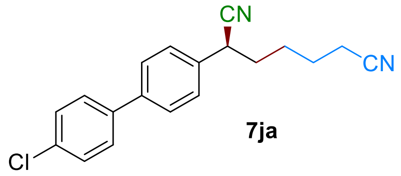

7ja

Peak #	RetTime [min]	Type	Width [min]	Area mAU *s	Height [mAU]	Area %
1	12.468	BB	0.2503	367.08051	22.73881	95.4885
2	14.009	BB	0.2917	17.34332	9.46884e-1	4.5115

Chiral HPLC spectrum of racemic 7ka

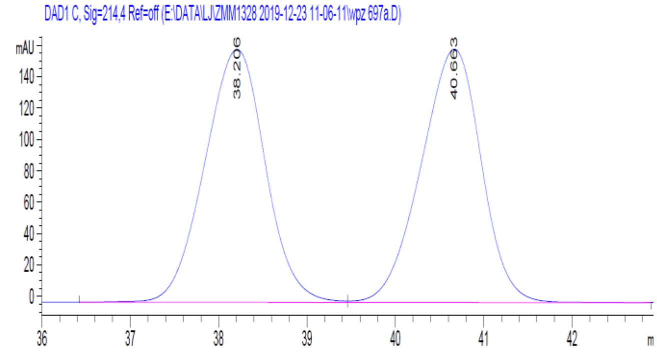

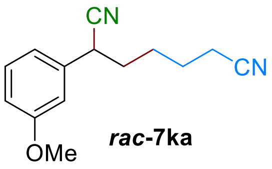

***rac*-7ka**

Peak #	RetTime [min]	Type	Width [min]	Area mAU *s	Height [mAU]	Area %
1	38.206	BV	0.7403	7686.18115	161.20288	50.1124
2	40.663	VB	0.7369	7651.69385	161.46255	49.8876

Chiral HPLC spectrum of 7ka

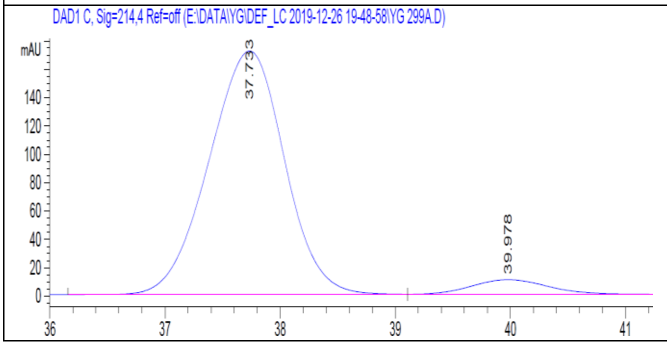

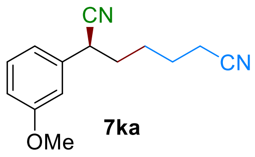

7ka

Peak #	RetTime [min]	Type	Width [min]	Area mAU *s	Height [mAU]	Area %
1	37.733	BV	0.7147	7851.08936	171.93684	93.8138
2	39.978	VB	0.7628	517.71161	10.58118	6.1862

Chiral HPLC spectrum of racemic 7la

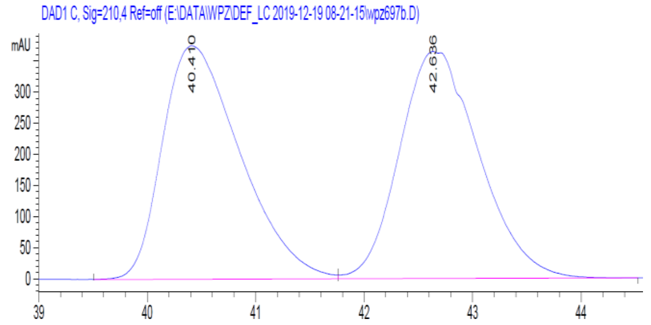

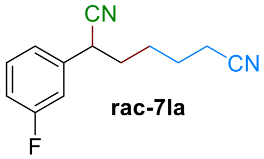

rac-7la

Peak #	RetTime [min]	Type	Width [min]	Area mAU *s	Height [mAU]	Area %
1	40.410	BV	0.7171	1.87418e4	374.97266	50.1040
2	42.636	VB	0.6571	1.86640e4	364.67343	49.8960

Chiral HPLC spectrum of 7la

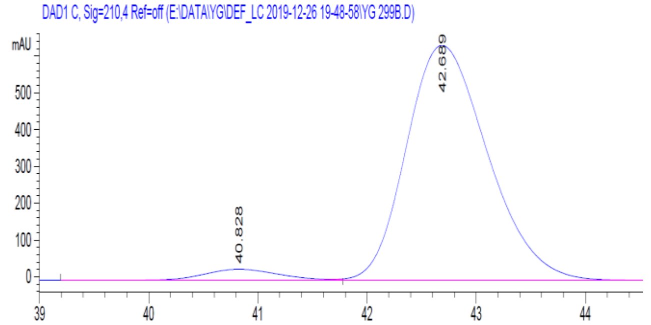

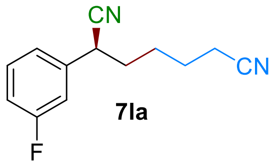

7la

Peak #	RetTime [min]	Type	Width [min]	Area mAU *s	Height [mAU]	Area %
1	40.828	BV E	0.7316	1404.49304	29.92396	4.0727
2	42.689	VB R	0.8122	3.30813e4	638.77887	95.9273

Chiral HPLC spectrum of racemic 7ma

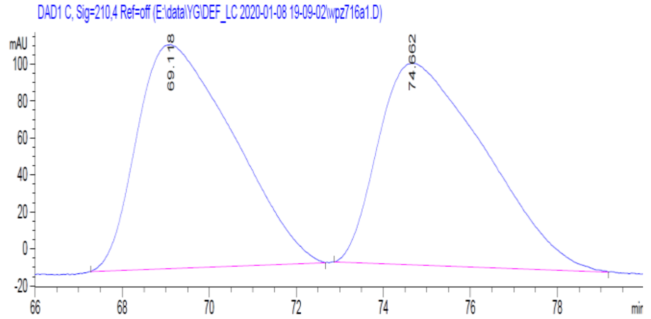

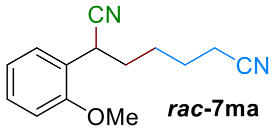

rac-7ma

Peak #	RetTime [min]	Type	Width [min]	Area mAU *s	Height [mAU]	Area %
1	69.118	BB	1.8062	1.86723e4	121.27937	50.0180
2	74.662	BB	2.0091	1.86588e4	109.22105	49.9820

Chiral HPLC spectrum of 7ma

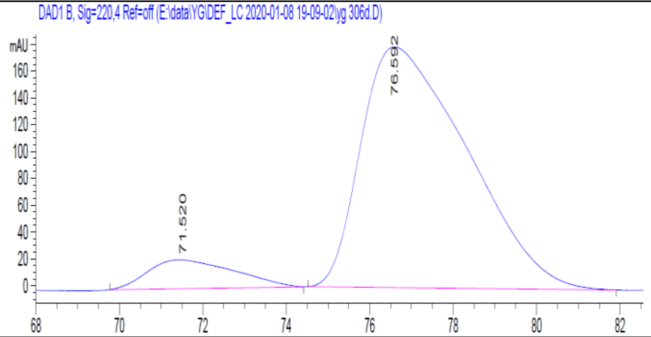

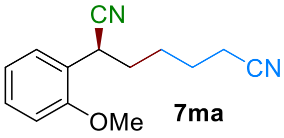

7ma

Peak #	RetTime [min]	Type	Width [min]	Area mAU *s	Height [mAU]	Area %
1	71.520	BB	1.6831	3072.51172	21.37394	8.8543
2	76.592	BB	2.0821	3.16283e4	179.28667	91.1457

Chiral HPLC spectrum of racemic 7na

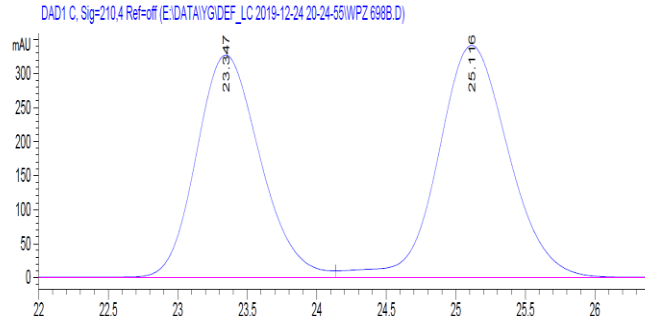

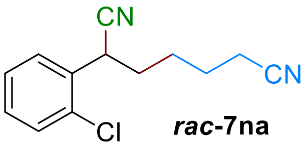

***rac*-7na**

Peak #	RetTime [min]	Type	Width [min]	Area mAU *s	Height [mAU]	Area %
1	23.347	BV	0.4961	1.04212e4	326.76123	46.0991
2	25.116	VV R	0.5398	1.21849e4	340.81155	53.9009

Chiral HPLC spectrum of 7na

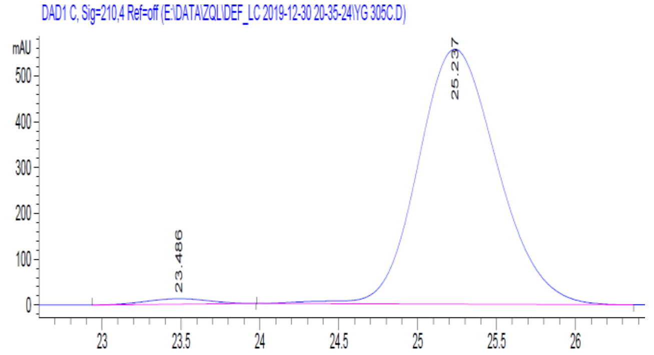

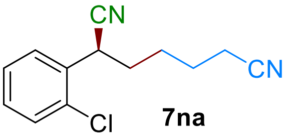

7na

Peak #	RetTime [min]	Type	Width [min]	Area mAU *s	Height [mAU]	Area %
1	23.486	BB	0.4444	332.02386	11.94119	1.6747
2	25.237	BB	0.5446	1.94939e4	556.63635	98.3253

Chiral HPLC spectrum of racemic 7oa

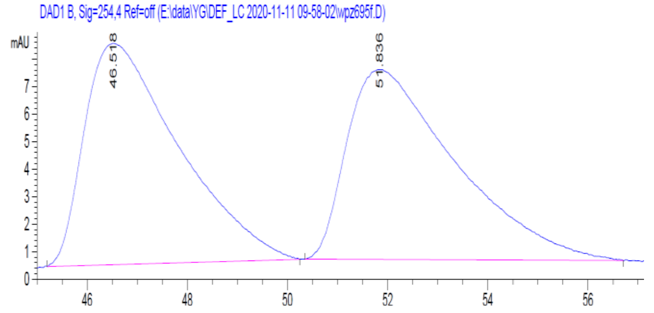

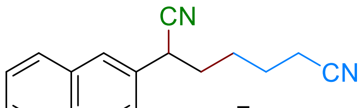

***rac*-7oa**

Peak #	RetTime [min]	Type	Width [min]	Area mAU *s	Height [mAU]	Area %
1	46.518	BB	1.5390	1051.60803	8.04523	50.2473
2	51.836	BB	1.7695	1041.25684	6.90434	49.7527

Chiral HPLC spectrum of 7oa

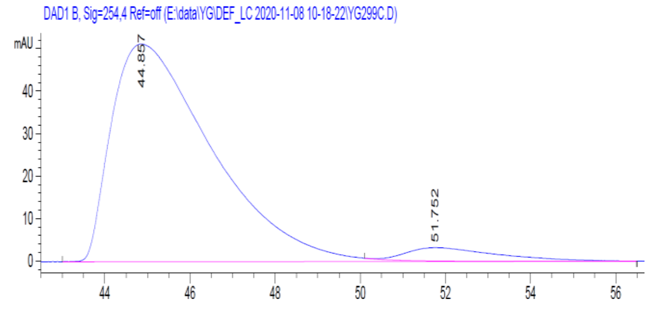

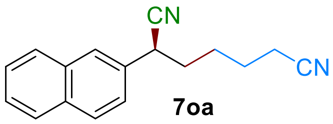

7oa

Peak #	RetTime [min]	Type	Width [min]	Area mAU *s	Height [mAU]	Area %
1	44.857	BV R	2.3492	8423.43262	51.25913	94.7261
2	51.752	VB E	1.7584	468.97684	3.15461	5.2739

Chiral HPLC spectrum of racemic 7pa

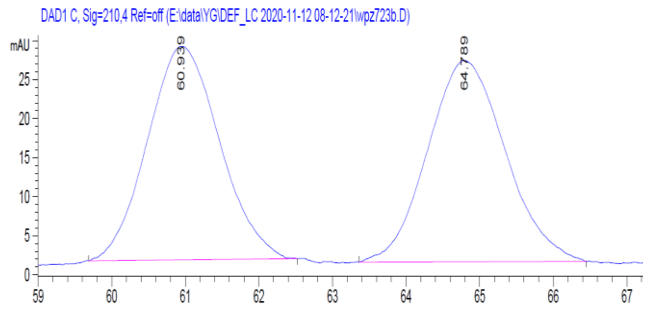

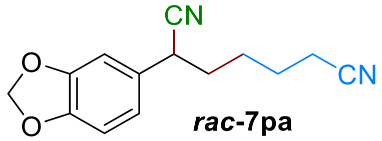

***rac*-7pa**

Peak #	RetTime [min]	Type	Width [min]	Area mAU *s	Height [mAU]	Area %
1	60.939	BB	0.8206	1867.03467	27.34484	49.6546
2	64.789	BB	0.8619	1893.01001	25.80211	50.3454

Chiral HPLC spectrum of 7pa

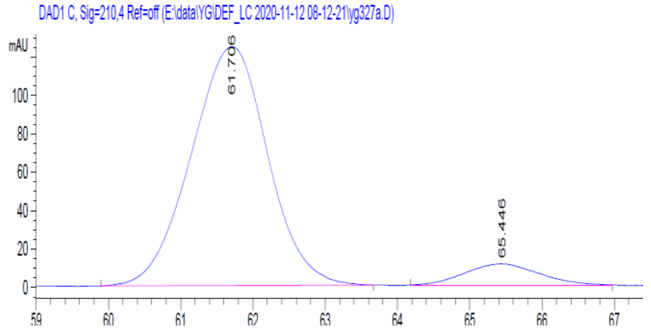

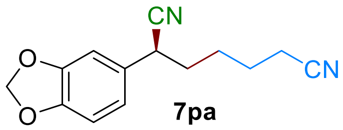

7pa

Peak #	RetTime [min]	Type	Width [min]	Area mAU *s	Height [mAU]	Area %
	61.706	BB	1.0418	9169.72754	124.42031	92.1151
	65.446	BB	0.8351	784.91534	11.04558	7.8849

Chiral HPLC spectrum of racemic 7qa

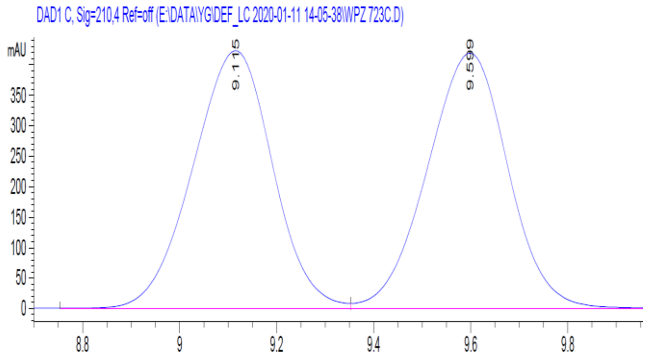

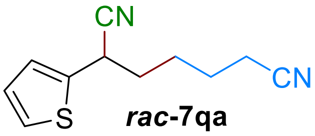

***rac*-7qa**

Peak #	RetTime [min]	Type	Width [min]	Area mAU *s	Height [mAU]	Area %
1	9.115	BV	0.1773	4826.81299	422.48315	49.9068
2	9.599	VV R	0.1808	4844.83252	418.43790	50.0932

Chiral HPLC spectrum of 7qa

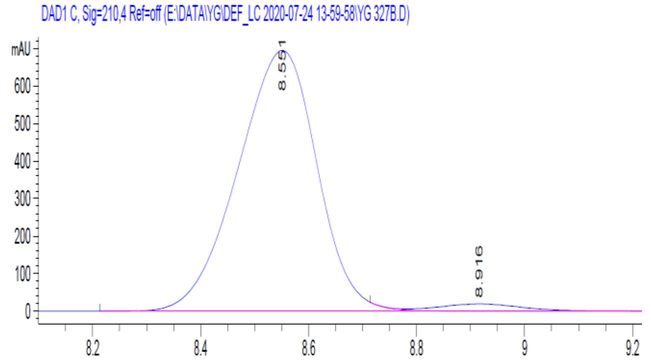

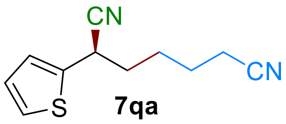

7qa

Peak #	RetTime [min]	Type	Width [min]	Area mAU *s	Height [mAU]	Area %
1	8.551	BV R	0.1589	7094.09863	695.72693	97.0706
2	8.916	VV E	0.1691	214.08406	19.34188	2.9294

Chiral HPLC spectrum of racemic 7ra

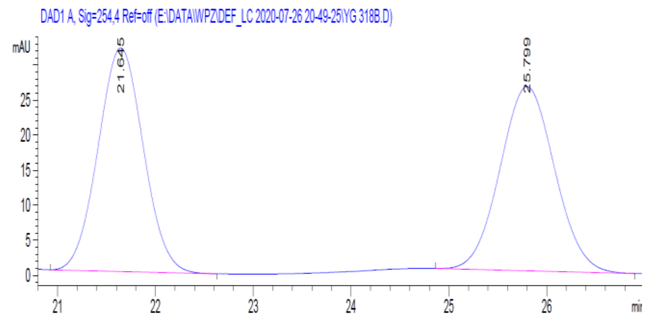

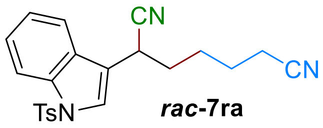

rac-**7ra**

Peak #	RetTime [min]	Type	Width [min]	Area mAU *s	Height [mAU]	Area %
1	21.645	BB	0.4978	1026.89648	31.87973	50.1559
2	25.799	BB	0.5995	1020.51337	26.25250	49.8441

Chiral HPLC spectrum of 7ra

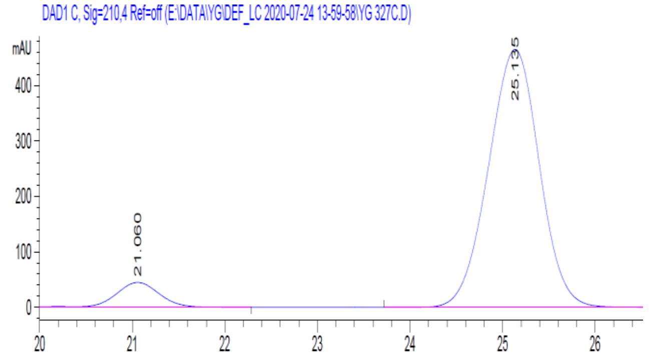

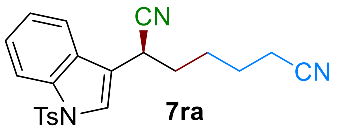

7ra

Peak #	RetTime [min]	Type	Width [min]	Area mAU *s	Height [mAU]	Area %
1	21.060	VB R	0.5052	1480.59888	44.76218	7.7077
2	25.135	BB	0.5971	1.77287e4	464.72021	92.2923

Chiral HPLC spectrum of racemic 7sa

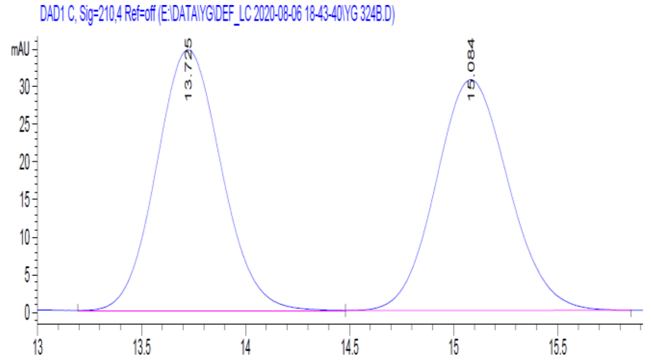

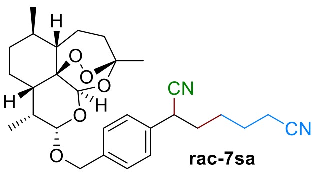

rac-7sa

Peak #	RetTime [min]	Type	Width [min]	Area mAU *s	Height [mAU]	Area %
1	13.725	BB	0.3377	753.58368	34.64970	50.2507
2	15.084	BB	0.3800	746.06592	30.64406	49.7493

Chiral HPLC spectrum of 7sa

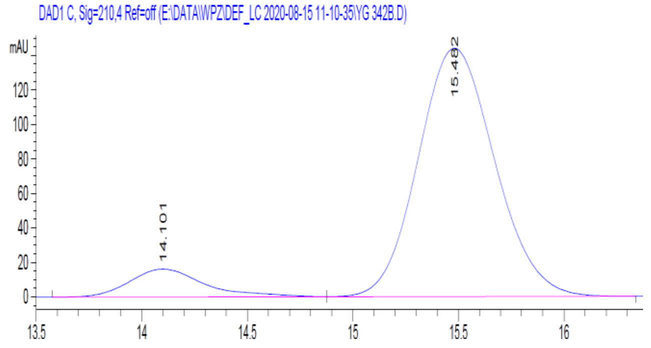

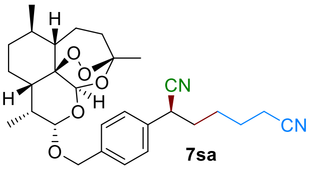

7sa

Peak #	RetTime [min]	Type	Width [min]	Area mAU *s	Height [mAU]	Area %
1	14.101	BV	0.3750	401.23770	16.19930	9.9420
2	15.482	VB	0.3927	3634.56543	143.90121	90.0580

Chiral HPLC spectrum of racemic 7ta

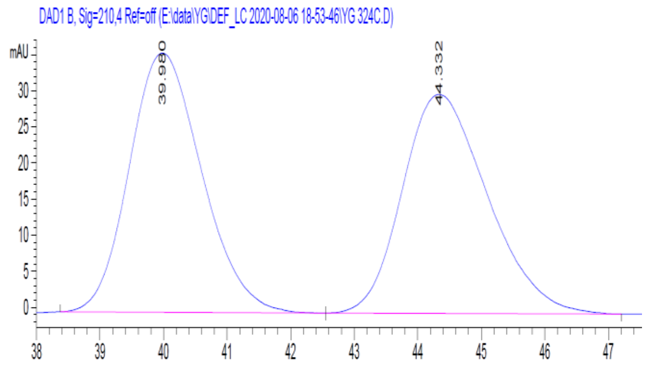

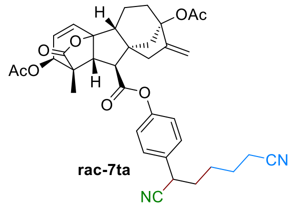

Peak #	RetTime [min]	Type	Width [min]	Area mAU *s	Height [mAU]	Area %
1	39.980	BB	1.2361	2850.92676	35.91521	50.1987
2	44.332	BB	1.3350	2828.36206	30.35714	49.8013

Chiral HPLC spectrum of 7ta

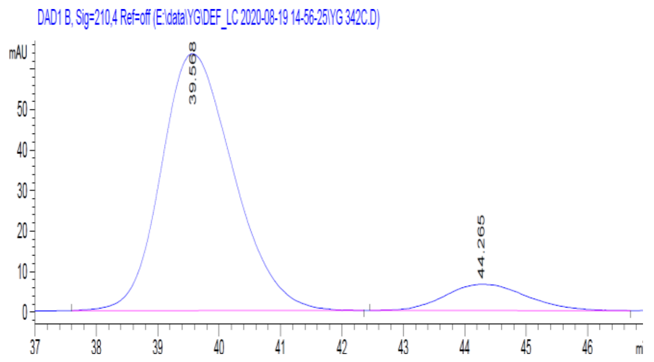

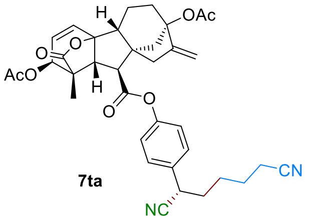

Peak #	RetTime [min]	Type	Width [min]	Area mAU *s	Height [mAU]	Area %
1	39.568	BB	1.2044	5195.65186	63.51122	89.5756
2	44.265	BB	1.1234	604.64532	6.49932	10.4244

Chiral HPLC spectrum of racemic 7ib

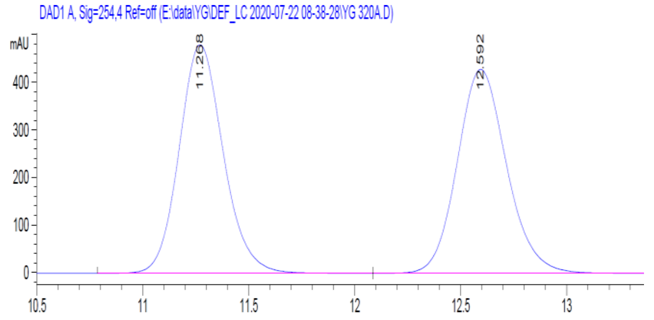

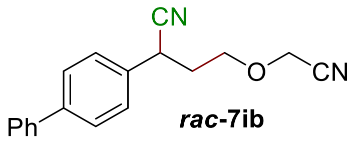

Peak #	RetTime [min]	Type	Width [min]	Area mAU *s	Height [mAU]	Area %
1	11.268	BB	0.2224	6939.21631	480.69553	49.9721
2	12.592	BV R	0.2507	6946.95605	428.98105	50.0279

Chiral HPLC spectrum of 7ib

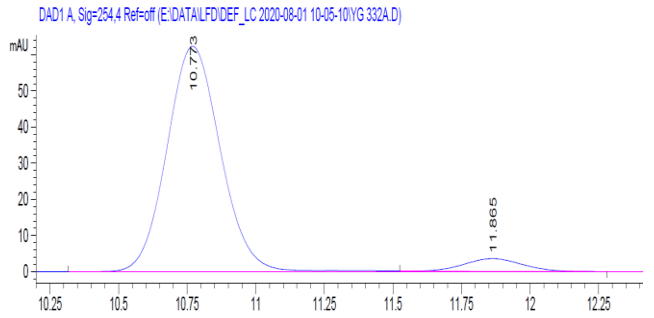

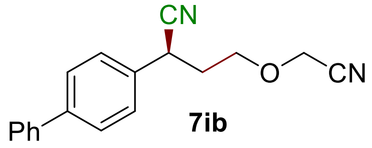

Peak #	RetTime [min]	Type	Width [min]	Area mAU *s	Height [mAU]	Area %
1	10.773	BV R	0.2154	851.28345	62.30468	94.2640
2	11.865	VB E	0.2281	51.80091	3.51141	5.7360

Chiral HPLC spectrum of racemic 7ic

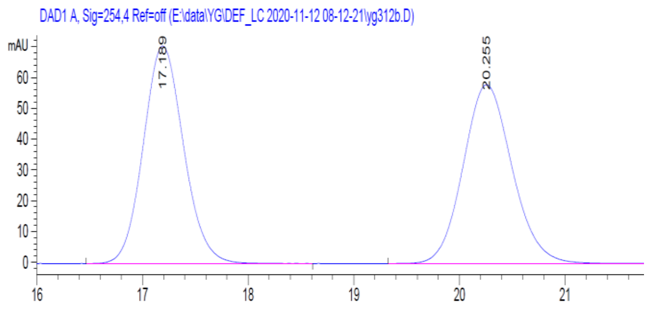

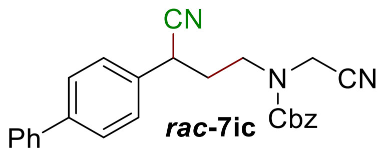

Peak #	RetTime [min]	Type	Width [min]	Area mAU *s	Height [mAU]	Area %
1	17.189	BB	0.4140	1874.86340	70.56655	50.0053
2	20.255	BB	0.4969	1874.46509	58.02673	49.9947

Chiral HPLC spectrum of 7ic

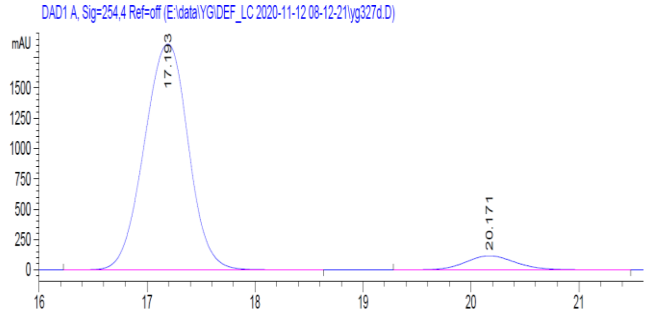

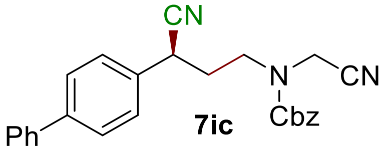

Peak #	RetTime [min]	Type	Width [min]	Area mAU *s	Height [mAU]	Area %
1	17.193	BB	0.4490	5.28224e4	1861.72534	93.4634
2	20.171	BB	0.4948	3694.28003	115.60804	6.5366

Chiral HPLC spectrum of racemic 7id

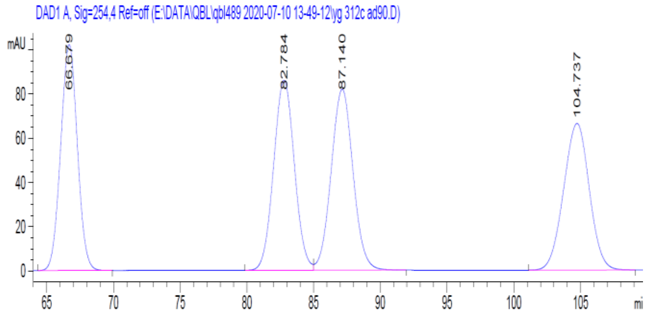

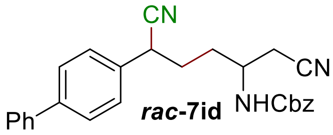

Peak #	RetTime [min]	Type	Width [min]	Area mAU *s	Height [mAU]	Area %
1	66.679	BB	1.3657	8865.48926	102.02963	24.3906
2	82.784	BV	1.6830	9289.91699	85.81342	25.5583
3	87.140	VB	1.7594	9333.32813	81.78730	25.6777
4	104.737	BB	2.0596	8859.22266	66.27247	24.3734

Chiral HPLC spectrum of 7id

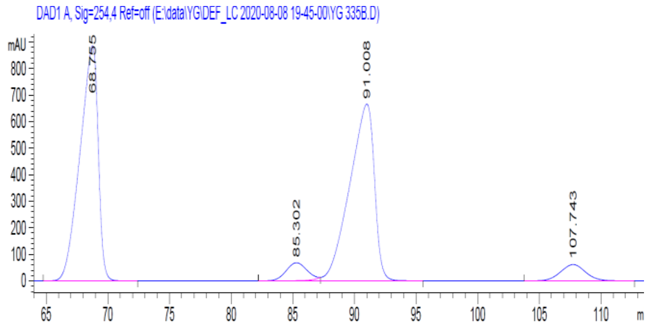

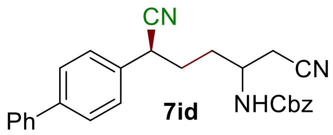

Peak #	RetTime [min]	Type	Width [min]	Area mAU *s	Height [mAU]	Area %
1	68.755	BB	1.5299	9.08987e4	885.28345	45.3752
2	85.302	BV E	1.8144	7812.13916	67.49421	3.8997
3	91.008	VB R	2.0385	9.30311e4	665.66479	46.4397
4	107.743	BB	2.1395	8584.69629	61.08928	4.2854

Chiral HPLC spectrum of racemic 7ie

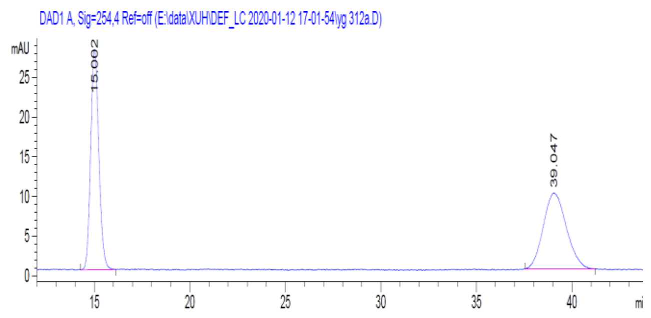

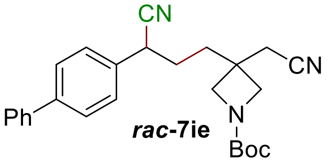

***rac*-7ie**

Peak #	RetTime [min]	Type	Width [min]	Area mAU *s	Height [mAU]	Area %
1	15.002	BB	0.4460	822.41614	27.73011	50.5838
2	39.047	BB	0.9931	803.43219	9.55434	49.4162

Chiral HPLC spectrum of 7ie

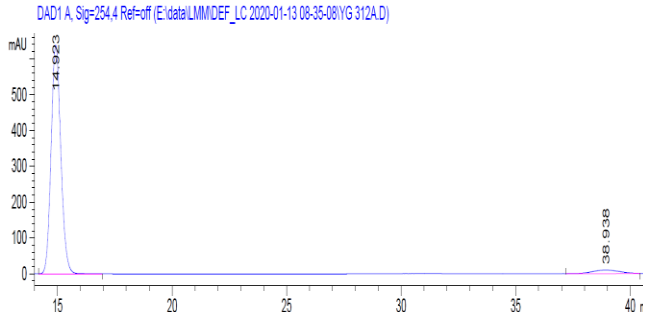

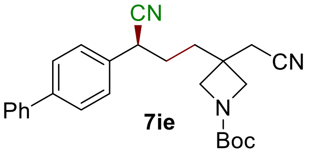

7ie

Peak #	RetTime [min]	Type	Width [min]	Area mAU *s	Height [mAU]	Area %
1	14.923	VB	0.4548	1.87748e4	638.88629	96.0692
2	38.938	BB	0.9842	768.19684	9.45184	3.9308

Chiral HPLC spectrum of racemic 7if

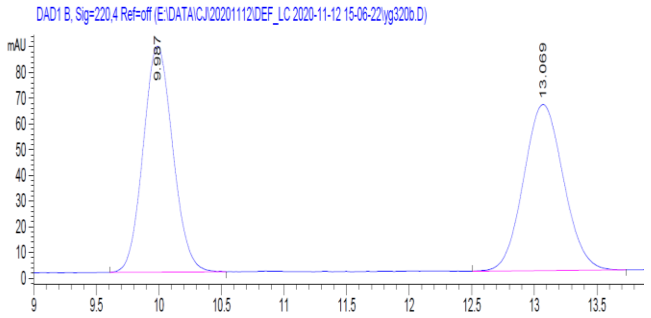

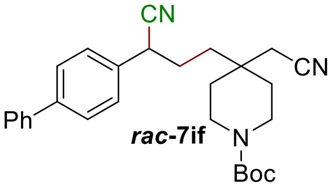

***rac*-7if**

Peak #	RetTime [min]	Type	Width [min]	Area mAU *s	Height [mAU]	Area %
1	9.987	BB	0.2561	1446.87671	87.87951	50.2184
2	13.069	BB	0.3448	1434.29370	64.64941	49.7816

Chiral HPLC spectrum of 7if

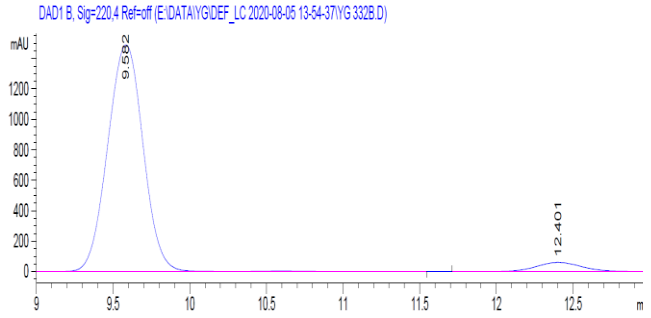

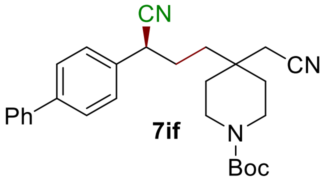

7if

Peak #	RetTime [min]	Type	Width [min]	Area mAU *s	Height [mAU]	Area %
1	9.582	BV R	0.2531	2.39643e4	1488.90857	95.0703
2	12.401	BV	0.3211	1242.63477	60.12391	4.9297

Chiral HPLC spectrum of racemic 7ig

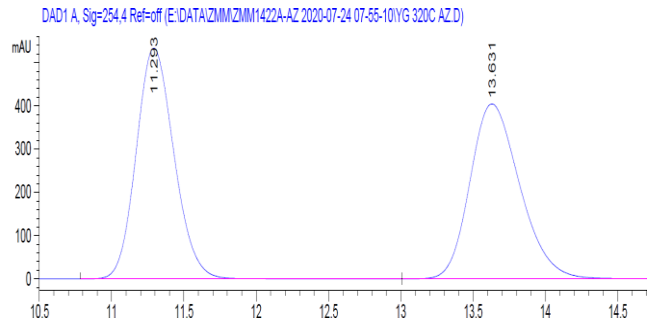

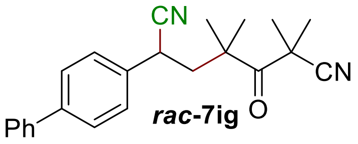

rac-**7ig**

Peak #	RetTime [min]	Type	Width [min]	Area mAU *s	Height [mAU]	Area %
1	11.293	BB	0.2838	9677.42285	533.04529	49.8002
2	13.631	BV R	0.3710	9755.08203	404.44504	50.1998

Chiral HPLC spectrum of 7ig

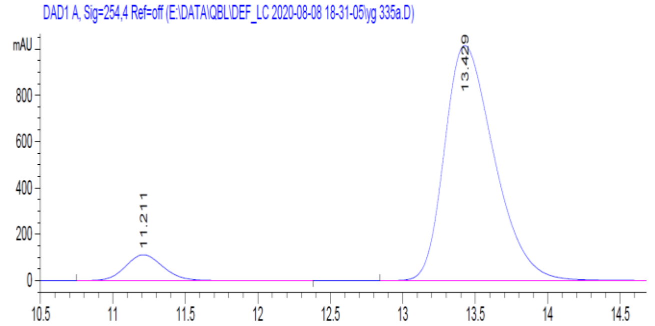

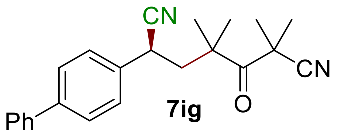

7ig

Peak #	RetTime [min]	Type	Width [min]	Area mAU *s	Height [mAU]	Area %
1	11.211	BB	0.2808	1982.54773	110.77823	7.6013
2	13.429	BB	0.3652	2.40990e4	1014.42395	92.3987

Chiral HPLC spectrum of racemic 8

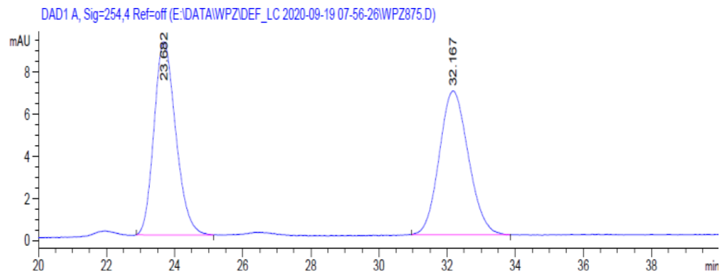

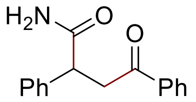

rac-**8**

Peak #	RetTime [min]	Type	Width [min]	Area mAU *s	Height [mAU]	Area %
1	23.682	BB	0.6454	406.86636	9.17482	50.0290
2	32.167	BB	0.7086	406.39420	6.82593	49.9710

Chiral HPLC spectrum of 8

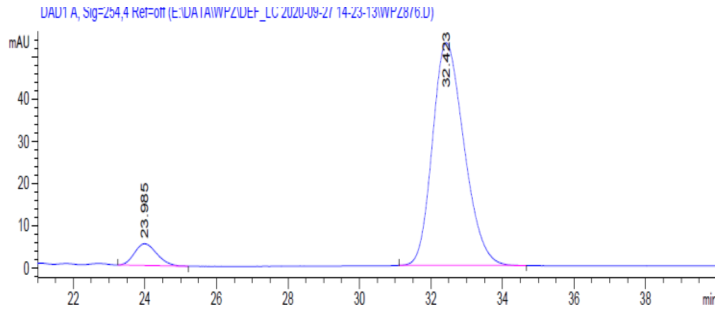

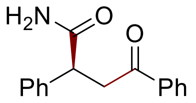

8

Peak #	RetTime [min]	Type	Width [min]	Area mAU *s	Height [mAU]	Area %
1	23.985	BB	0.5256	225.42056	5.16070	6.5104
2	32.423	BB	0.9235	3237.05811	52.77891	93.4896

Chiral HPLC spectrum of racemic 9

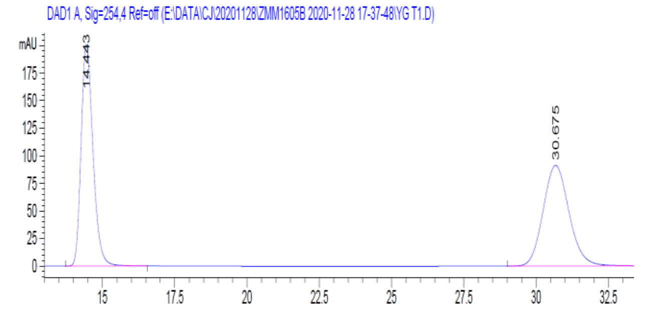

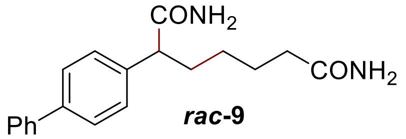

Peak #	RetTime [min]	Type	Width [min]	Area mAU *s	Height [mAU]	Area %
1	14.443	BB	0.4378	5713.20117	200.85770	50.1019
2	30.675	BB	0.9621	5689.95605	91.17242	49.8981

Chiral HPLC spectrum of 9

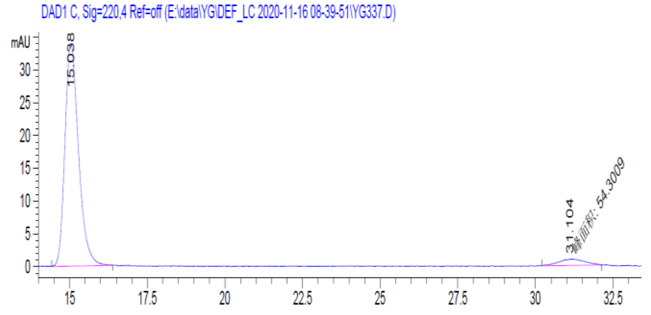

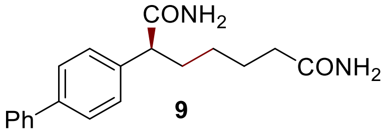

Peak #	RetTime [min]	Type	Width [min]	Area mAU *s	Height [mAU]	Area %
1	15.038	BB	0.4648	1033.59229	33.98882	95.0086
2	31.104	MM	0.9391	54.30095	9.63659e-1	4.9914

Chiral HPLC spectrum of racemic 10

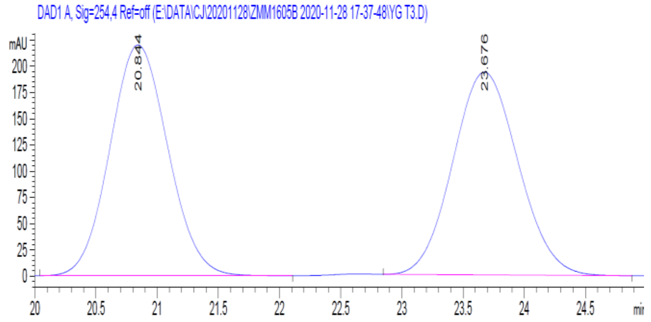

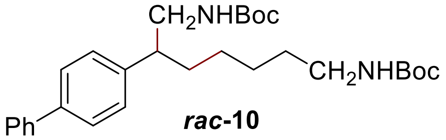

Peak #	RetTime [min]	Type	Width [min]	Area mAU *s	Height [mAU]	Area %
1	20.844	BB	0.5165	7238.64209	219.63838	50.2142
2	23.676	BB	0.5797	7176.89893	193.06720	49.7858

Chiral HPLC spectrum of 10

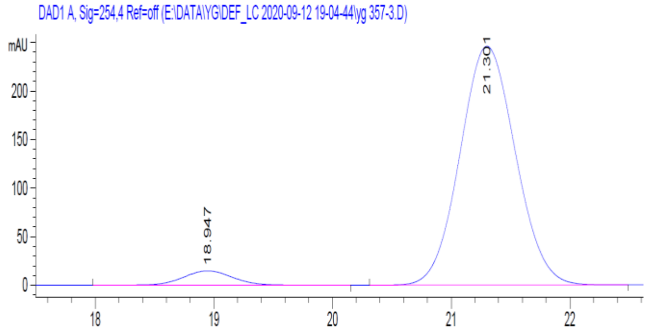

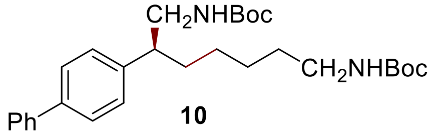

Peak #	RetTime [min]	Type	Width [min]	Area mAU *s	Height [mAU]	Area %
1	18.947	BB	0.4445	423.88858	14.69668	5.0459
2	21.301	BB	0.5067	7976.76953	245.76576	94.9541

Chiral HPLC spectrum of racemic 11

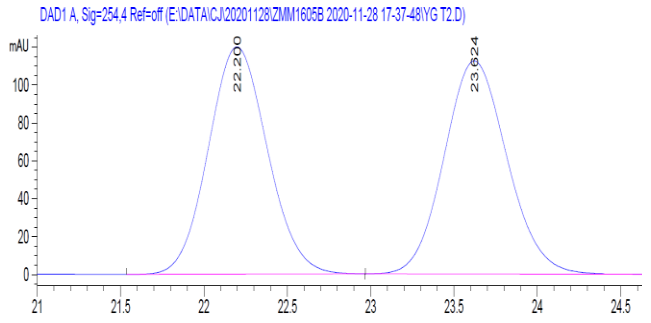

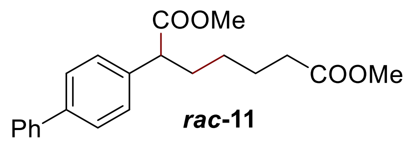

***rac*-11**

Peak #	RetTime [min]	Type	Width [min]	Area mAU *s	Height [mAU]	Area %
1	22.200	BB	0.3835	2956.68506	119.96191	50.0301
2	23.624	BB	0.4085	2953.12695	112.41398	49.9699

Chiral HPLC spectrum of 11

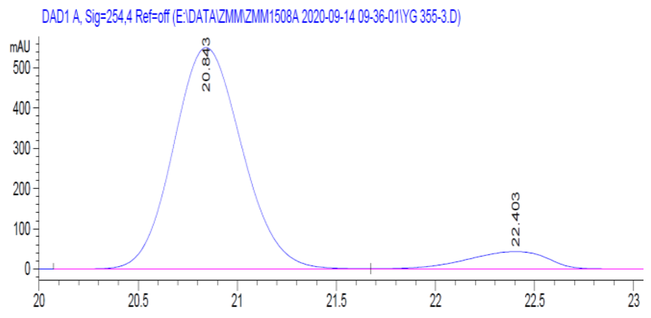

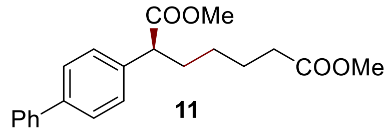

11

Peak #	RetTime [min]	Type	Width [min]	Area mAU *s	Height [mAU]	Area %
1	20.843	BV	0.3759	1.31947e4	550.05920	91.6554
2	22.403	VB	0.4470	1201.27930	42.86132	8.3446

Chiral HPLC spectrum of racemic 14

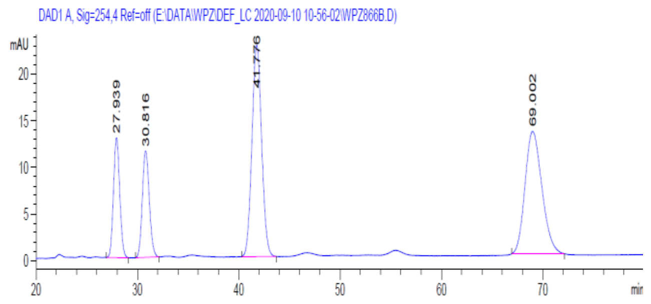

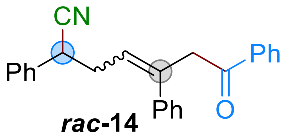

***rac*-14**

Peak #	RetTime [min]	Type	Width [min]	Area mAU *s	Height [mAU]	Area %
1	27.939	BB	0.6111	552.39545	12.86132	13.3714
2	30.816	BB	0.6967	547.20966	11.42572	13.2459
3	41.776	BB	0.9322	1506.79492	22.70724	36.4738
4	69.002	BB	1.3670	1524.76782	13.16677	36.9089

Chiral HPLC spectrum of 14

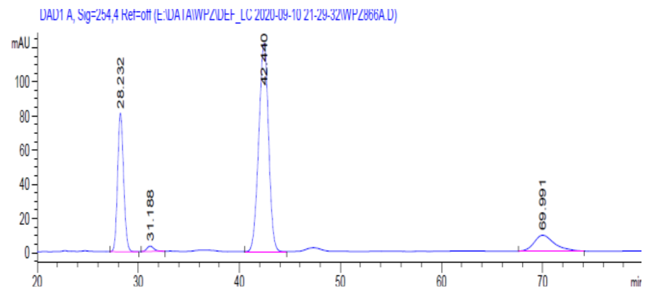

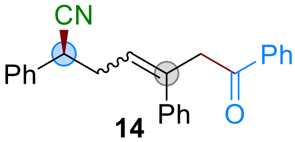

14

Peak #	RetTime [min]	Type	Width [min]	Area mAU *s	Height [mAU]	Area %
1	28.232	BB	0.6441	3366.54810	81.13069	25.8441
2	31.188	BB	0.6211	155.06454	3.15688	1.1904
3	42.440	BB	1.0384	8218.65234	121.92053	63.0924
4	69.991	BB	1.6206	1286.11780	9.30486	9.8732

Chiral HPLC spectrum of racemic 15

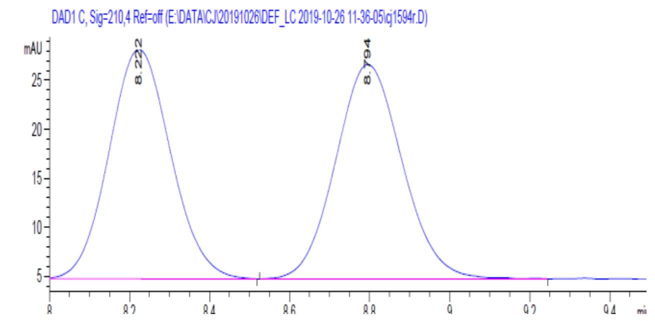

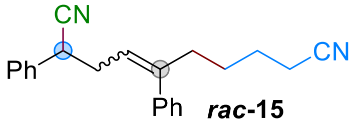

rac-**15**

Peak #	RetTime [min]	Type	Width [min]	Area mAU *s	Height [mAU]	Area %
1	8.222	BB	0.1650	250.92964	23.42686	49.7552
2	8.794	BB	0.1771	253.39909	21.88677	50.2448

Chiral HPLC spectrum of 15

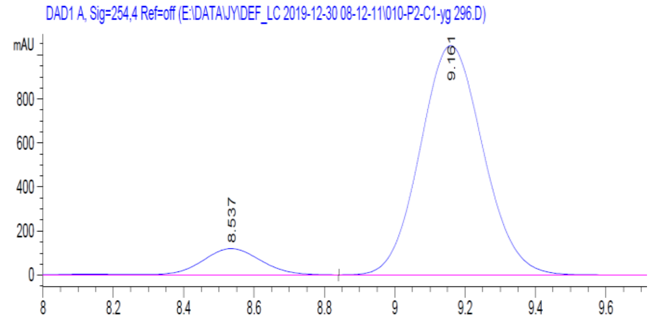

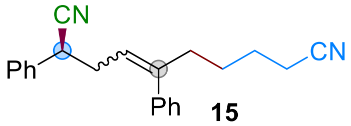

15

Peak #	RetTime [min]	Type	Width [min]	Area mAU *s	Height [mAU]	Area %
1	8.537	VB R	0.1792	1400.67468	119.27027	9.7694
2	9.161	BV R	0.1948	1.29367e4	1041.92419	90.2306
